# The Hippo tumor suppressor pathway triggers non-cell autonomous tumorigenesis in *Drosophila*

**DOI:** 10.1038/s44319-026-00778-5

**Published:** 2026-05-01

**Authors:** Daichi Honda, Misako Okumura, Ayano Oi, Chisako Sakuma, Fumiaki Obata, Toshinori Ando, Masayuki Miura, Takahiro Chihara

**Affiliations:** 1 Program of Biomedical Science, Graduate School of Integrated Sciences for Life, Hiroshima University, Higashi-Hiroshima, Japan; 2 Program of Basic Biology, Graduate School of Integrated Sciences for Life, Hiroshima University, Higashi-Hiroshima, Japan; 3 Laboratory of Environmental Genetics, Graduate School of Life Sciences, Tohoku University, Sendai, Japan; 4 Laboratory for Nutritional Biology, RIKEN Center for Biosystems Dynamics Research, Kobe, Japan; 5 Metabolic and Behavioral Physiology RIKEN ECL Research Team, RIKEN Pioneering Research Institute, Kobe, Japan; 6 Laboratory of Molecular Cell Biology and Development, Graduate School of Biostudies, Kyoto University, Kyoto, Japan; 7 Department of Oral and Maxillofacial Pathobiology, Graduate School of Biomedical and Health Sciences, Hiroshima University, Hiroshima, Japan; 8 Laboratory for Cell Vigor Regulation, National Institute for Basic Biology, Okazaki, Japan

**Keywords:** Cancer, Signal Transduction

## Abstract

The Hippo pathway is a tumor suppressor pathway, and most related studies have indicated that its inhibition leads to tumorigenesis. However, recent studies have suggested that the activated Hippo pathway can promote tumorigenesis in certain contexts. Here, we demonstrate that the activated Hippo pathway induces non-cell-autonomous tumorigenesis, characterized by tumor markers in the *Drosophila* wing epithelium. This suggests that Hippo-activated cells behave similarly to “oncogenic niche cells.” We find that Hippo-activated cells induce Dronc-Wingless/Spitz signaling in the hinge/ventral notum region, which causes tumorigenesis. Moreover, we identify the amino acid transporters Sat1/2, which are implicated in amino acid incorporation and function redundantly with the growth factors Wingless and Spitz to facilitate non-cell-autonomous tumorigenesis.

## Synopsis

Hippo signaling in Drosophila wing discs promotes the growth of neighboring tumors. This non-cell-autonomous effect depends on growth factor signaling and amino acid transporters.

- Hippo signaling induces tumorigenesis in neighboring cells.
- Growth factors Wg and Spitz mediate this non-cell-autonomous effect.
- The amino acid transporters Sat1 and Sat2 enhance amino acid uptake to enable tumor initiation.
- Sat1 and Sat2 cooperate with Wg and Spitz to drive tumorigenesis.

## Abstract (web summary)

Hippo signaling in Drosophila wing discs promotes the growth of neighboring tumors. This non-cell-autonomous effect depends on growth factor signaling and amino acid transporters.

## Graphical abstract

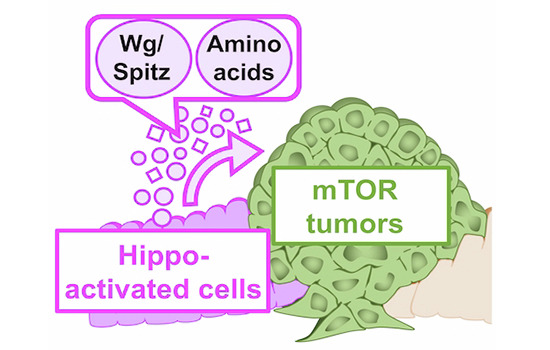

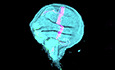

## Introduction

The Hippo pathway is a highly conserved tumor suppressor pathway that negatively regulates organ growth and tumorigenesis (Moroishi et al, 2015; Yu et al, 2015). The *Drosophila* Hippo pathway consists of two main components, Hippo (Hpo) and Warts (Wts), which target the transcriptional coactivator Yorkie (Yki: Yes-associated protein, YAP in mammals) (Moroishi et al, 2015; Yu et al, 2015). Because activation of the Hippo pathway suppresses Yki through its sequestration and degradation in the cytosol, the inactivation of the pathway translocates Yki to the nucleus and initiates tumorigenesis by promoting cell proliferation, cell competition, and migration (Ziosi et al, 2010; Thompson and Cohen, 2006; Ding et al, 2021; Nagata et al, 2022; Udan et al, 2003).

In contrast to this classic model in which the Hippo pathway functions as a tumor suppressor, recent reports have suggested Hippo pathway functions as a tumor promoter. For instance, LATS1/2 (human orthologs of Wts), which are components of the Hippo pathway, suppress antitumor responses by inhibiting the secretion of signal molecules to activate immune cells in murine cancer cells (Moroishi et al, 2016). Interestingly, LATS1/2 behave as a tumor promoter in vivo but not in vitro (Moroishi et al, 2016), questioning the role of the Hippo pathway in the body. YAP is frequently silenced in multiple cancers (Carter et al, 1994; Hampton et al, 1994; Cottini et al, 2014; Yuan et al, 2008; Pearson et al, 2021). For instance, loss of YAP promotes tumor growth in breast cancers and migration in hematological cancers (Carter et al, 1994; Hampton et al, 1994; Cottini et al, 2014; Yuan et al, 2008). These recent studies suggest that in certain contexts, Hippo activation/YAP silencing functions as a tumor promoter. However, the mechanisms underlying tumorigenesis induced by Hippo activation remain poorly understood.

We investigated the tumor-promoting role of the Hippo pathway using a *Drosophila* wing disc, in which tumorigenesis can be analyzed using genetic manipulations (Martínez-Abarca Millán et al, 2023; Gandille et al, 2010). We found that Hippo activation can induce tumors characterized by cell proliferation markers, including activated mTOR pathway, phospho-Histone H3 (PH3), 5-ethynyl-2′-deoxyuridine (EdU) staining, and invasive markers, including c-Jun N-terminal kinase (JNK) pathway activation, matrix metalloproteinase 1 (MMP1) expression, and cell polarity defects. These tumors were induced by Hippo-activated cells in a non-cell-autonomous manner. Moreover, the tumorigenic effect, including mTOR activation, occurs through Hippo target genes, including *rpr*, *hid, grim* (an apoptotic inducer), *atg8a* (an autophagy-related gene), *cyclin E* (a proliferative activator), and the microRNA *bantam*. We found that the signaling pathway of Apoptosis-induced Proliferation (AiP), Dronc–Wingless (Wg)/Spitz signaling, acting downstream of Rpr, Hid, and Grim (Fan et al, 2014; Bergmann and Fan, 2025), was required for mTOR activation caused by Hippo activation. Moreover, we found that the evolutionarily conserved amino acid transporters, Strip-interacting amino acid transporters 1/2 (Sat1/2), were implicated in amino acid incorporation and were required for and redundantly worked with growth factors for mTOR activation caused by Hippo activation. Therefore, we suggest that Hippo-activated cells cause non-cell-autonomous tumorigenesis via growth factors Wg/Spitz and Sat1/2. Our findings provide novel insights into the Hippo pathway and may be useful for the development of cancer treatments targeting this pathway.

## Results

### The activated Hippo pathway induces tumorigenesis in a non-cell autonomous manner

**Figure 1:**
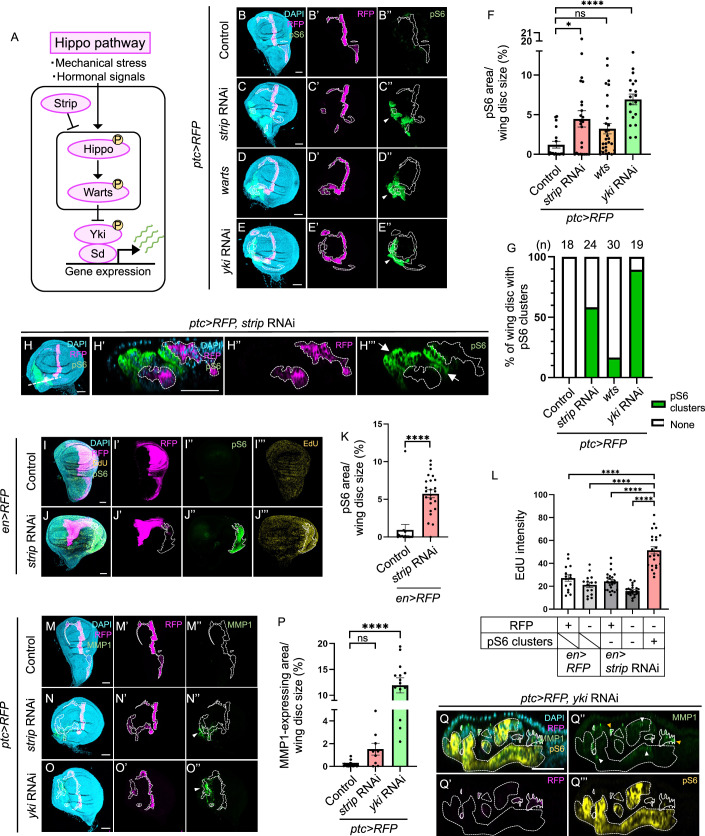
The activated Hippo pathway upregulates the mTOR pathway, EdU incorporation, and MMP1 expression in a non-cell-autonomous manner. (**A**) The schematic depiction indicates the Hippo pathway and its function. (**B**–**E**) Confocal images showing wing discs bearing wild-type, *strip-*, *yki-*knocked down, or *wts*-overexpressed cells marked with RFP expression (magenta) and stained with anti-phospho-S6 (green). The white arrowhead shows the pS6 cell clusters. (**F**) Quantification of the size of the phospho-S6 positive region (% of phospho-S6 positive area/disc area) in wild-type, *strip-, yki*-knocked down, or *wts-*overexpressed wing disc. ns, not significant; ^∗^*P* < 0.05; ^∗∗∗∗^*P* < 0.0001; one-way ANOVA with Dunnett’s multiple comparison test. Sample size and *P* value: *n* = 18 (*ptc* > *RFP*), *n* = 24, *P* = 1.50 × 10^−2^ (*ptc>strip* RNAi), *n* = 30, *P* = 1.57 × 10^−1^ (*ptc>wts*), *n* = 19, *P* = 2.54 × 10^−5^ (*ptc>yki* RNAi). (**G**) Incidence of mTOR (phospho-S6)-activated tumors. Sample size: *n* = 18 (*ptc* > *RFP*), *n* = 24 (*ptc > strip* RNAi), *n* = 30 (*ptc > wts*), *n* = 19 (*ptc > yki* RNAi). (**H**) Confocal images showing wing discs bearing *strip-*knockdown cells marked with RFP expression (magenta) and stained with anti-phospho-S6 (green). (**H’**–**H”’**) Vertical sections at a site indicated by a dashed line in (**H**). The white arrow indicates non-cell-autonomous activation of the mTOR pathway. RFP (magenta) outlined by white dashed lines marks the expression pattern of *ptc-Gal4* in the wing disc (**B**–**E**, **H**). (**I**, **J**) Confocal images showing the wing discs bearing wild-type or *strip-*knockdown cells marked with RFP expression (magenta) and stained with anti-phospho-S6 (green) and EdU (yellow). RFP (magenta) marks the expression pattern of *en-Gal4* in the wing disc. pS6 cell clusters are outlined by white dashed lines. (**K**) Quantification of the size of the phospho-S6 positive region (% of phospho-S6 positive area/disc area) in wild-type or *strip-*knockdown wing disc. ^∗∗∗∗^*P* < 0.0001; Welch’s *t* test. Sample size and *P* value: *n* = 15, (*en* > *RFP*), *n* = 24, *P* = 4.09 × 10^−6^ (*en>strip* RNAi). (**L**) Quantification of the EdU intensity (EdU intensity/RFP-positive, RFP-negative, or pS6-cell cluster area [pixels]) in the wing disc bearing wild-type or *strip*-knockdown cells. ^∗∗∗∗^*P* < 0.0001; one-way ANOVA with Dunnett’s multiple comparison test. Sample size and *P* value: *n* = 15, *P* = 9.02 × 10^−10^ (RFP^+^ area in *en* > *RFP*), *n* = 15, *P* = 2.05 × 10^−13^ (RFP^−^ area in *en* > *RFP*), *n* = 23, *P* = 1.09 × 10^−13^ (RFP^+^ area in *en>strip* RNAi), *n* = 23, *P* = 7 × 10^−15^ (RFP^−^ area in *en>strip* RNAi), *n* = 23 (pS6^+^ area in *en>strip* RNAi). (**M**–**O**) Confocal images showing the wing discs bearing wild-type, *strip-*, or *yki-*knocked down cells marked with RFP expression (magenta) and stained with anti-MMP1 (green). The white arrowhead shows the MMP1-expressing cells. RFP (magenta) outlined by white dashed lines marks the expression pattern of *ptc-Gal4* in the wing disc. (**P**) Quantification of the MMP1-expressing area (% of MMP1-expressing area/disc area) in wild-type, *strip*-, or *yki*-knockdown wing disc. ns, not significant; ^∗∗∗∗^*P* < 0.0001; one-way ANOVA with Dunnett’s multiple comparison test. Sample size and *P* value: *n* = 22 (*ptc* > *RFP*), *n* = 9, *P* = 5.86 × 10^−1^ (*ptc>strip* RNAi), *n* = 18, *P* = 2.07 × 10^−12^ (*ptc>yki* RNAi). (**Q**) Confocal images showing wing discs bearing *yki-*knockdown cells marked with RFP expression (magenta) and stained with anti-MMP1 (green) and anti-pS6 (yellow). Vertical sections at a site indicated by a dashed line in Fig. EV2B. The white arrowheads indicate non-cell-autonomous MMP1 expression in pS6 cell clusters, outlined by white dashed lines. The yellow arrowheads indicate cell-autonomous MMP1 expression in the Hippo-activated cells, outlined by solid lines. Scale bars represent 50 μm in (**B**–**E**, **H**–**J**, **M**–**O**, **Q**). Dots represent biological replicates (**F**, **K**, **L**, **P**); numbers indicate the number of biological replicates (**G**); error bars indicate SEM. Source data are available online for this figure.

First, we genetically induced Hippo-activated cells in the *Drosophila* epithelial tissue, the wing disc, using the *ptc-Gal4* driver expressed at the anterior/posterior (A/P) compartment boundary. Because the Hippo pathway is suppressed by the Striatin-interacting phosphatase and kinase (STRIPAK) complex, the knockdown of the STRIPAK component *strip* can effectively activate the Hippo pathway (Fig. 1A; Chen et al, 2019). In addition, *wts* overexpression or *yki* knockdown induces Hippo-activated phenotypes (Fig. 1A; Huang et al, 2005; Oh and Irvine, 2008). We examined the effects of Hippo activation on tumorigenesis using the following markers: tissue growth/proliferation markers (mTOR pathway activation, EdU incorporation, and PH3), invasion markers (JNK pathway activation, MMP1 expression, and cell polarity defects), which are associated with the tumorigenesis of *Drosophila* and mammals (Nagata et al, 2022; Zhang et al, 2022; Zou et al, 2020; Parker and Struhl, 2020; Sun et al, 2019; Montemurro et al, 2025; Parra and Johnston, 2020; Uhlirova and Bohmann, 2006).

**Figure EV1:**
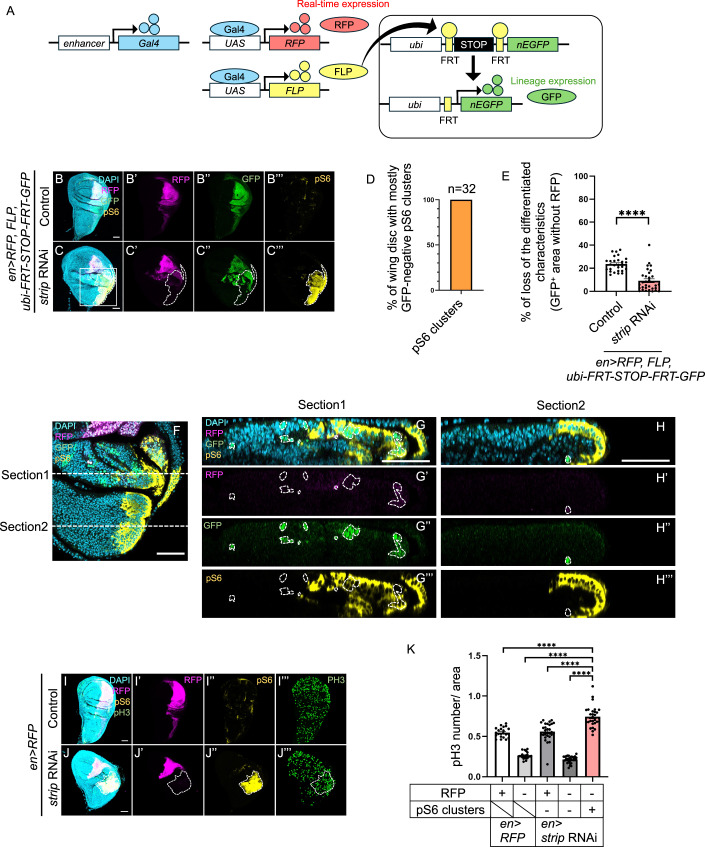
The mTOR pathway is activated in a non-cell-autonomous manner accompanied by PH3 upregulation. (**A**) The schematic representation illustrates the G-TRACE technique. Enhancer activity, indicated by real-time expression, is visualized through RFP expression facilitated by Gal4 binding to UAS. Conversely, upon *Gal4* expression, a ubiquitous promoter persistently induces GFP (lineage expression) in these cells via flippase-dependent excision of a STOP cassette. (**B**, **C**) Confocal images depict wing discs containing either wild-type or *strip*-knockdown cells, marked with G-TRACE, demonstrating the lineage of Gal4 (green) through GFP expression and active Gal4 through RFP expression (magenta), and stained with anti-phospho-S6 (yellow). RFP (magenta) delineates the current expression pattern, whereas GFP indicates the lineage of *en-Gal4* in the wing disc. pS6 cell clusters are outlined by white dashed lines. (**D**) Incidence of predominantly GFP-negative pS6 clusters. Sample size: *n* = 32 (*en* > *RFP, strip RNAi, FLP, ubi-FRT-STOP-FRT-GFP*). (**E**) Quantification of the area of loss of differentiated characteristics (% of GFP^+^ area without RFP/entire GFP-positive area) in wing discs containing either wild-type or *strip*-knockdown cells with G-TRACE. ^∗∗∗∗^*P* < 0.0001; Welch’s *t* test. Sample size and *P* value: *n* = 28 (*en* > *RFP, FLP, ubi-FRT-STOP-FRT-GFP*), *n* = 31, *P* = 4.57 × 10^−9^ (*en* > *RFP, strip* RNAi*, FLP, ubi-FRT-STOP-FRT-GFP*). (**F**–**H**) High-magnification image (**F**) indicated in the white box in (**C**). (**G**, **H**) Vertical sections at a site indicated by a dashed line in (**F**). GFP (green) is outlined by white dashed lines. (**I**, **J**) Confocal images showing wing discs containing either wild-type or *strip*-knockdown cells marked with RFP expression (magenta) and stained with anti-phospho-S6 (yellow) and anti-PH3 (green). RFP (magenta) delineates the expression pattern of *en-Gal4* in the wing disc. pS6 cell clusters are outlined by white dashed lines. (**K**) Quantification of the number of PH3 (the number of PH3/RFP-positive, RFP-negative, or pS6 cell-cluster area [pixels]) in wing discs containing either wild-type or *strip*-knockdown cells. Normalized values were scaled by a factor of 100. ∗∗∗∗*P* < 0.0001; one-way ANOVA with Dunnett’s multiple comparison test. Sample size and *P* value: *n* = 22, *P* = 6.96 × 10^−12^ (RFP^+^ area in *en* > *RFP*), *n* = 22, *P* < 1 × 10^−15^ (RFP^−^ area in *en* > *RFP*), *n* = 29, *P* = 3.93 × 10^−12^ (RFP^+^ area in *en>strip* RNAi), *n* = 29, *P* < 1 × 10^−15^ (RFP^−^ area in *en>strip* RNAi), *n* = 29 (pS6^+^ area in *en>strip* RNAi). Numbers indicate the number of biological replicates (**D**); dots represent biological replicates (**E**, **K**); error bars indicate SEM.

The mTOR signaling was sparsely and weakly activated in the wild-type wing disc, as indicated by scattered staining with an antibody against phosphorylated ribosomal protein S6 (pS6) (Fig. 1B). In contrast, Hippo-activated cells induced strong activation of mTOR signaling (pS6 staining in Fig. 1B–E, quantified in F and G). All three types of Hippo-activated/Yki-suppressed cells, *strip* RNAi, *wt*s overexpression, and *yki* RNAi, activated mTOR signaling, although the induction efficiency of mTOR-activated cell clusters differed among the three types (Fig. 1F,G). Interestingly, Hippo-activated cells rarely overlapped with cells with mTOR activation, and mTOR activation was induced in the cells surrounding the Hippo-activated cells (Fig. 1H). In addition to *ptc-Gal4*, when using the *en-Gal4* driver expressed in the posterior compartment, mTOR-activated cell clusters emerged around Hippo-activated cells (Fig. 1I,J, quantified in K), suggesting that non-cell-autonomous mTOR activation is induced by Hippo activation. To further support this hypothesis, we performed a lineage analysis of Hippo-activated cells using G-TRACE (Evans et al. 2009) and found that mTOR-activated cells were not actively formed by dedifferentiated/transformed Hippo-activated cells (Fig. EV1A–C). Because the majority of mTOR-activated cells were neither currently nor previously activated by the Hippo pathway (Fig. EV1B,C,F–H, quantified in D), and activation of the Hippo pathway did not lead to the loss of differentiation/transformation characteristics (Fig. EV1B,C, quantified in E), the mTOR-activated cell cluster was not a consequence of the loss of differentiation/transformation characteristics in Hippo-activated cells. Thus, Hippo-activated cells drive mTOR signaling in surrounding cells. Moreover, the proliferation of mTOR-activated cell clusters was strongly upregulated, as indicated by the increased EdU incorporation (Fig. 1I,J, quantified in L) and PH3 levels (Fig. EV1I,J, quantified in K). Thus, the mTOR-activated cell cluster is hyperplastic characterized by excessive proliferation.

**Figure EV2:**
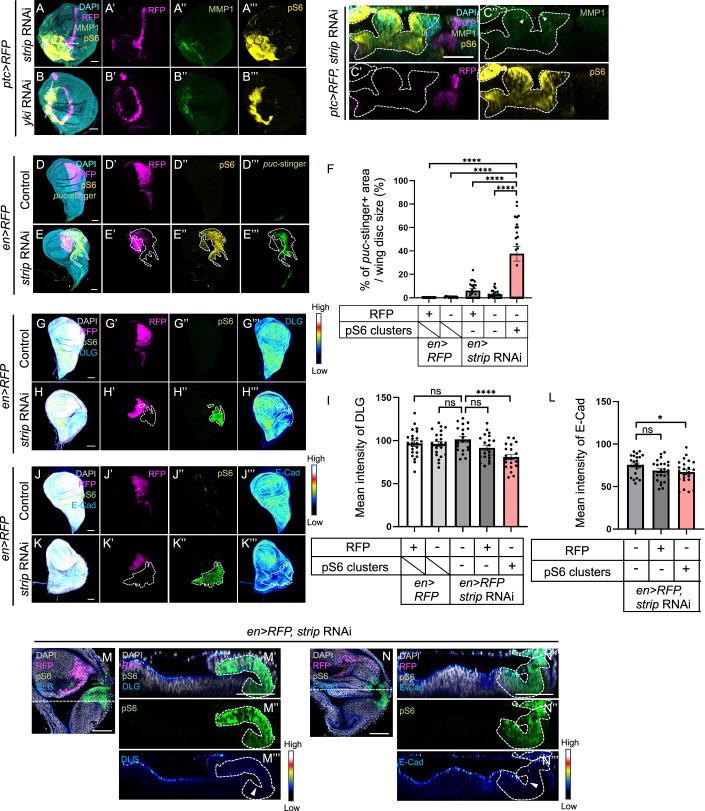
MMP1 and the JNK pathway are upregulated and cell polarity proteins are downregulated in mTOR-activated cell clusters. (**A**–**C**) Confocal microscopy images depicting wing discs containing either *strip* or *yki*-knocked down cells, marked by RFP expression (magenta), and stained with anti-phospho-S6 (yellow) and anti-MMP1 (green). (**C**) Vertical sections at the site indicated by the dashed line in (**A**). The white arrowheads indicate MMP1-expressing cells within the pS6 cell clusters, which are delineated by white dashed lines. RFP (magenta) highlights the expression pattern of *ptc-Gal4* in wing discs. (**D**, **E**) Confocal images illustrating wing discs with either wild-type or *strip*-knockdown cells marked by RFP expression (magenta), stained with anti-phospho-S6 (yellow), and labeled with *puc-stinger* to indicate JNK pathway activity (green). RFP (magenta) indicates the expression pattern of *en-Gal4* on wing discs. The pS6 cell clusters are outlined by white dashed lines. (**F**) Quantification of the *puc-stinger-*labeled area (% of *puc-stinger*-labeled area/RFP-positive, RFP-negative, or pS6 cell-cluster area) in wing discs containing either wild-type or *strip*-knockdown cells. ∗∗∗∗*P* < 0.0001; one-way ANOVA with Dunnett’s multiple comparison test. Sample in and strip value: *n* = 13, *P* = 1.88 × 10^−9^ (RFP^+^ area in *en* > *RFP*), *n* = 13, *P* = 2.82  × 10^−9^ (RFP^−^ area in *en* > *RFP*), *n* = 23, *P* = 3.69 × 10^−9^ (RFP^+^ area in *en>strip* RNAi), *n* = 23, *P* = 1.30 × 10^−10^ (RFP^−^ area in *en>strip* RNAi), *n* = 23 (pS6^+^ area in *en>strip* RNAi). (**G**, **H**) Confocal images showing wing discs with either wild-type or *strip*-knockdown cells, marked by RFP expression (magenta), and stained with anti-phospho-S6 (green) and anti-DLG (rainbow LUT). RFP (magenta) indicates the expression pattern of *en-Gal4* on wing discs. The pS6 cell clusters are outlined by white dashed lines. (**I**) Quantification of DLG intensity (DLG intensity/disc area [pixels]) in wing discs bearing wild-type or *strip*-knockdown cells. ns, not significant; ^∗∗∗∗^*P* < 0.0001; one-way ANOVA with Dunnett’s multiple comparison test. Sample size and *P* value: *n* = 23, *P* = 6.49 × 10^−1^ (RFP^+^ area in *en* > *RFP*), *n* = 23, *P* = 5.71 × 10^−1^ (RFP^−^ area in *en* > *RFP*), *n* = 21 (RFP^−^ area in *en>strip* RNAi), *n* = 21, *P* = 1.00 × 10^−1^ (RFP^+^ area in *en>strip* RNAi), *n* = 21, *P* = 4.60 × 10^−5^ (pS6^+^ area in *en>strip* RNAi). (**J**, **K**) Confocal images showing wing discs with either wild-type or *strip*-knockdown cells, marked by RFP expression (magenta), and stained with anti-phospho-S6 (green) and anti-E-Cad (rainbow LUT). RFP (magenta) indicates the expression pattern of *en-Gal4* on wing discs. The pS6 cell clusters are outlined by white dashed lines. (**L**) Quantification of E-Cad intensity (E-Cad intensity/disc area [pixels]) in wing discs bearing wild-type or *strip*-knockdown cells. ns, not significant; ^∗^*P* < 0.05; one-way ANOVA with Dunnett’s multiple comparison test. Sample size and *P* value: *n* = 24 (RFP^−^ area in *en>strip* RNAi), *n* = 24, *P* = 1.13 × 10^−1^ (RFP^+^ area in *en>strip* RNAi), *n* = 24, *P* = 3.83 × 10^−2^ (pS6^+^ area in *en>strip* RNAi). (**M**, **N**) High-magnification images showing wing discs with *strip*-knocked down cells stained with anti-phospho-S6 (green), DLG (rainbow LUT), or E-Cad (rainbow LUT). (**M’**–**M”’**, **N**–**N”’**) Vertical sections at a site indicated by a dashed line in (**M**, **N**). White arrowheads indicate DLG- or E-Cad-depleted spots. Scale bars represent 50 μm in (**A**–**E**, **G**, **H**, **J**, **K**, **M**, **N**). Dots represent biological replicates (**F**, **I**, **L**); error bars indicate SEM.

In addition to proliferation markers, Hippo-activated cells induced MMP1 expression (Fig. 1M–O, quantified in P). MMP1 upregulation occurred in both *strip*/*yki* RNAi lines, although the MMP1 upregulation by *yki* RNAi was stronger than that by *strip* RNAi (Fig. 1P). MMP1 upregulation was induced by Hippo activation in both cell-autonomous and non-cell-autonomous manners (Figs. 1Q and EV2A–C). The MMP1 expression was frequently observed in mTOR-activated cells in a non-cell-autonomous manner (Figs. 1Q and  EV2C). Subsequently, we investigated the JNK pathway, which enhances MMP1 expression (Sun et al, 2019; Uhlirova and Bohmann, 2006). We used *puc-stringer*, a JNK reporter, and observed a significant increase in the *puc-stringer* signal within mTOR-activated cell clusters (Fig. EV2D,E, quantified in F). These findings suggest that mTOR-activated cell clusters upregulate MMP1 expression via JNK activation. Furthermore, we examined the loss of cell polarity, which is associated with the acquisition of invasive capabilities (Montemurro et al, 2025). mTOR-activated cell clusters tended to exhibit reduced expression of the tight junction protein Discs large (DLG) and adherens junction protein E-cadherin (E-Cad) (Fig. EV2G,H,J,K,M,N, quantified in I and L). These findings imply that Hippo-activated cells behave like “oncogenic niche cells,” potentially inducing dysplastic (mTOR-activated) tumors in a non-cell-autonomous manner.

### Hippo activation in the hinge/ventral notum regions induces mTOR-activated cell clusters

**Figure 2:**
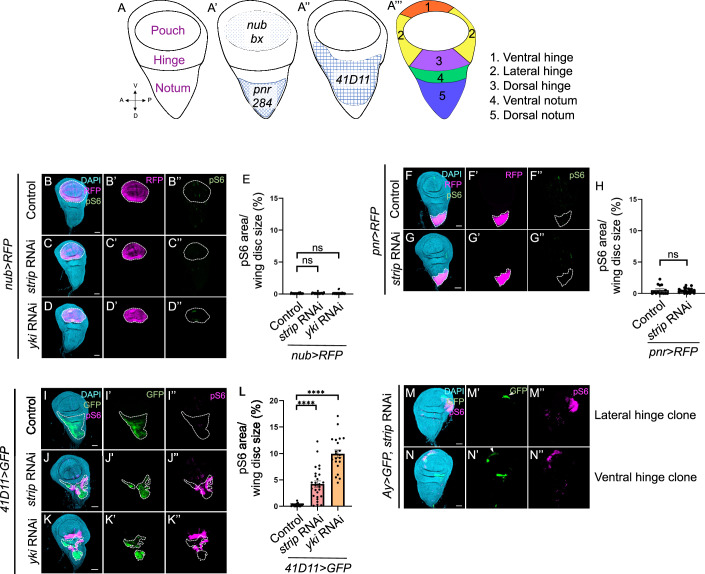
Hippo activation in the hinge/ventral notum region induces mTOR-activated tumors. (**A**) The schematic depictions indicate the compartments (A), the active area of *nub-Gal4*, *bx-Gal4*, *pnr-Gal4, 284-Gal4* (**A’**), and *41D11-Gal4* (**A”**), and subcompartments (**A”’**) in the wing disc. (**B**–**D**) Confocal images showing the wing discs bearing wild-type, *strip-*, or *yki*-knockdown cells marked with RFP expression (magenta) and stained with anti-phospho-S6 (green). RFP (magenta) outlined by white dashed lines marks the expression pattern of *nub-Gal4* in the wing disc. (**E**) Quantification of the size of the phospho-S6 positive region (% of phospho-S6 positive area/disc area) in the wild-type, *strip-*, or *yki*-knockdown wing disc. ns, not significant; one-way ANOVA with Dunnett’s multiple comparison test. Sample size and *P* value: *n* = 24 (*nub* > *RFP*), *n* = 20, *P* = 5.46 × 10^−1^ (*nub>strip* RNAi), *n* = 26, *P* = 5.95 × 10^−1^ (*nub>yki* RNAi). (**F**, **G**) Confocal images showing the wing discs bearing wild-type or *strip*-knockdown cells marked with RFP expression (magenta) and stained with anti-phospho-S6 (green). RFP (magenta) outlined by white dashed lines marks the expression pattern of *pnr-Gal4* in the wing disc. (**H**) Quantification of the size of the phospho-S6 positive region (% of phospho-S6 positive area/disc area) in the wild-type or *strip*-knockdown wing disc. ns, not significant; Welch’s *t* test. Sample size and *P* value: *n* = 14 (*pnr* > *RFP*), *n* = 22, *P* = 8.15 × 10^−1^ (*pnr>strip* RNAi). (**I**–**K**) Confocal images showing the wing discs bearing wild-type, *strip-*, or *yki*-knockdown cells marked with GFP expression (green) and stained with anti-phospho-S6 (magenta). GFP (green) outlined by white dashed lines marks the expression pattern of *41D11-Gal4* in the wing disc. (**L**) Quantification of the size of the phospho-S6 positive region (% of phospho-S6 positive area/disc area) in the wild-type, *strip-*, or *yki*-knockdown wing disc. ∗∗∗∗*P* < 0.0001; one-way ANOVA with Dunnett’s multiple comparison test. Sample size and *P* value: *n* = 20 (*41D11* > *GFP*), *n* = 27, *P* = 6.19 × 10^−6^ (*41D11>strip* RNAi), *n* = 21, *P* < 1 × 10^−15^ (*41D11>yki* RNAi). (**M**, **N**) Confocal images showing wing discs bearing *strip-*knocked down clones marked with GFP expression (green) and stained with anti-phospho-S6 (magenta). GFP (green) marks the expression pattern of *Ay-Gal4* in the wing disc. The white arrowheads indicate the *strip-*knocked down clones in the lateral/ventral hinge. Scale bars represent 50 μm in (**B**–**D**, **F**, **G**, **I**–**K**, **M**, **N**). Dots represent biological replicates (**E**, **H**, **L**); error bars indicate SEM. Source data are available online for this figure.

**Figure EV3:**
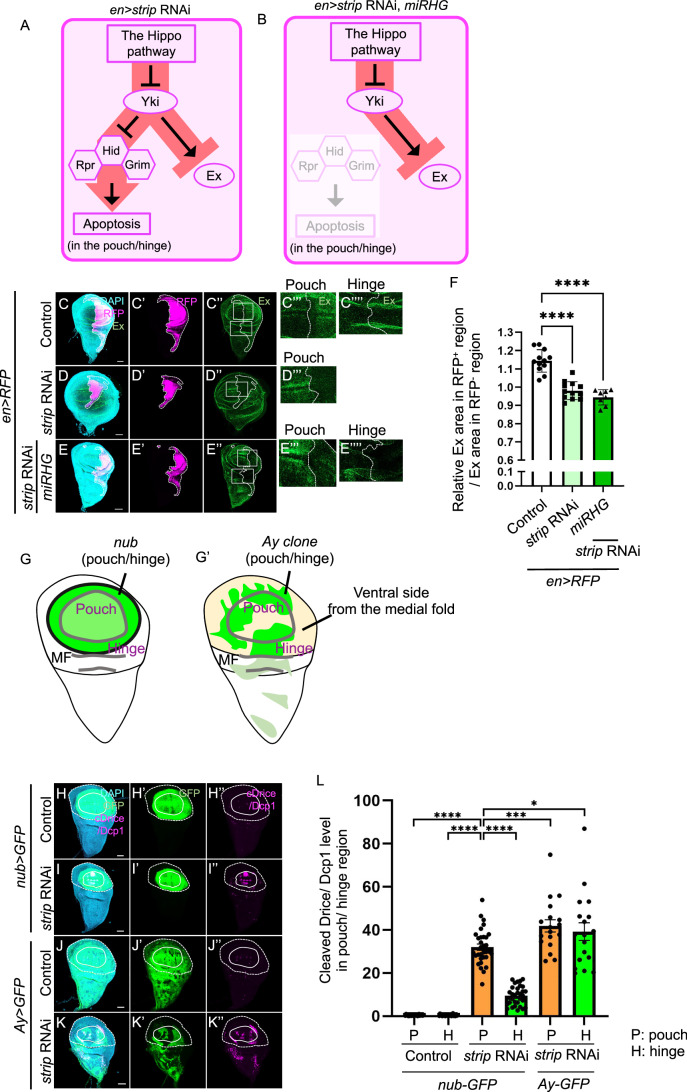
The Hippo signaling pathway is activated by *strip* RNAi in the pouch/hinge region. (**A**, **B**) The schematic representation illustrates a signaling pathway activated by Hippo through *strip* RNAi, with or without the knockdown of *rpr, hid, and grim* (*miRHG*). *miRHG* inhibits apoptosis in the pouch and hinge regions, facilitating the analysis of these areas under Hippo activation. (**C**–**E**) Confocal images displaying wing discs containing either wild-type or *strip*-knockdown cells, with or without *miRHG*, marked by RFP expression (magenta) and stained with anti-Expanded (green). RFP (magenta), delineated by white dashed lines, indicates the expression pattern of *en-Gal4* in the wing disc. High-magnification images (C’’’–E’’’’) indicated in the white boxes in (C’’–E’’). (**F**) Quantification of the relative Expanded level (Expanded positive area in RFP^+^ region/Expanded positive area in RFP^−^ region) in the wild-type or *strip*-knockdown wing disc. ∗∗∗∗*P* < 0.0001; one-way ANOVA with Dunnett’s multiple comparison test. Sample size and *P* value: *n* = 12 (*en* > *RFP*), *n* = 12, *P* = 4.63 × 10^−8^ (*en>strip* RNAi), *n* = 8, *P* = 7.28 × 10^−9^ (*en>strip* RNAi, *miRHG*). (**G**) The schematic depictions indicate the *nub-Gal4* active area in (**G**, green) and *Ay-Gal4* active clone in (**G’**, green). The hinge region on the ventral side from the medial fold (MF) in (**G’**, yellow). (**H**–**K**) Confocal images showing wing discs containing either wild-type or *strip*-knockdown cells marked with GFP expression and stained with anti-cleaved Drice/Dcp1 (magenta). GFP (green) indicates the expression pattern of *nub-Gal4* or *Ay-Gal4* in the wing disc. Solid lines and dashed lines outline the pouch region and hinge region on the ventral side of the medial fold (green clone in yellow area in **G’**), respectively. The female animals of these strains were selected for the experiment. (**L**) Quantification of the size of the cleaved Drice/Dcp1-positive region (% of anti-cleaved Drice/Dcp1-positive area/GFP-positive area) in the wing disc containing either wild-type or *strip*-knockdown cells. Quantification was conducted in the GFP-positive area in the pouch (P) or hinge region on the ventral side from the medial fold (H). ∗*P* < 0.05; ^∗∗∗^*P* < 0.001; ^∗∗∗∗^*P* < 0.0001; one-way ANOVA with Dunnett’s multiple comparison test. Sample size and *P* value: *n* = 25, *P* < 1 × 10^−15^ (pouch in *nub* > *GFP*), *n* = 25, *P* < 1 × 10^−15^ (hinge in *nub* > *GFP*), *n* = 32 (pouch in *nub>strip* RNAi), *n* = 32, *P* < 1 × 10^−15^ (hinge in *nub>strip* RNAi), *n* = 18, *P* = 5.26 × 10^−4^ (pouch in *Ay* > *GFP*), *n* = 18, *P* = 1.93 × 10^−2^ (hinge in *Ay>strip* RNAi). Scale bars represent 50  μm in (**C**–**E**, **H**–**K**). Dots represent biological replicates (**F**, **L**); error bars indicate SEM.

We generated Hippo-activated cells in the wing disc using *ptc-Gal4*, *ci-Gal4*, and *en-Gal4* drivers, which were expressed in the A/P compartment boundary, anterior compartment, and posterior compartment, respectively. mTOR-activated cell clusters tended to emerge in the putative hinge and notum regions, but not in the pouch region (Figs. 2A and 1B–E,I,J; Appendix Fig. S1A–C), although *strip* RNAi activated the Hippo pathway not only in the hinge region but also in the pouch region, as indicated by the suppression of Expanded, a *yki*-target gene (Fig. EV3A–E, quantified in F). This suggests that there may be cell type/region specificity for Hippo-activated cells to behave as oncogenic niche cells. To examine this possibility, we activated the Hippo pathway in the specific region of the wing disc, including the pouch, hinge, or notum region, using *nub-Gal4*, *bx-Gal4*, *pnr-Gal4*, *284-Gal4*, or *41D11-Gal4* (the expression pattern of each *Gal4* line is summarized in Fig. 2A). Hippo activation in the pouch or dorsal notum region using *nub-Gal4*, *bx-Gal4*, *pnr-Gal4*, or *284-Gal4* did not induce mTOR-activated cell clusters (Fig. 2B–D,F,G, quantified in E, H; Appendix Fig. S1D,E,G,H, quantified in F, I). However, apoptosis, which is a characteristic of Hippo activation, was observed in the pouch region (Fig. EV3H,I, quantified L). In contrast, when the Hippo pathway was activated in the dorsal hinge and ventral notum regions using *41D11-Gal4*, mTOR-activated cell clusters were observed (Fig. 2I–K, quantified in L). In addition, we used an *Ay-Gal4* driver (*actin promoter-FRT-y*^*+*^*-STOP-FRT-Gal4*) to randomly generate Hippo-activated clones in wing discs (Fig. 2M,N). This activation induced apoptosis in the hinge region on the ventral side of the medial fold (Fig. EV3G,J,K, quantified in L), which was not effectively elicited by Hippo activation using *nub-Gal4* (Fig. EV3H,I, quantified L). When Hippo-activated clones were induced in the ventral/lateral hinge region, mTOR-activated cell clusters emerged in adjacent areas (Fig. 2M,N). Therefore, we conclude that hinge/ventral notum cells could serve as oncogenic niche cells upon activation of the Hippo pathway.

### *rpr*, *hid*, *grim*, *bantam*, *cyclin E*, and *atg8a* are involved in non-cell-autonomous mTOR activation

**Figure 3:**
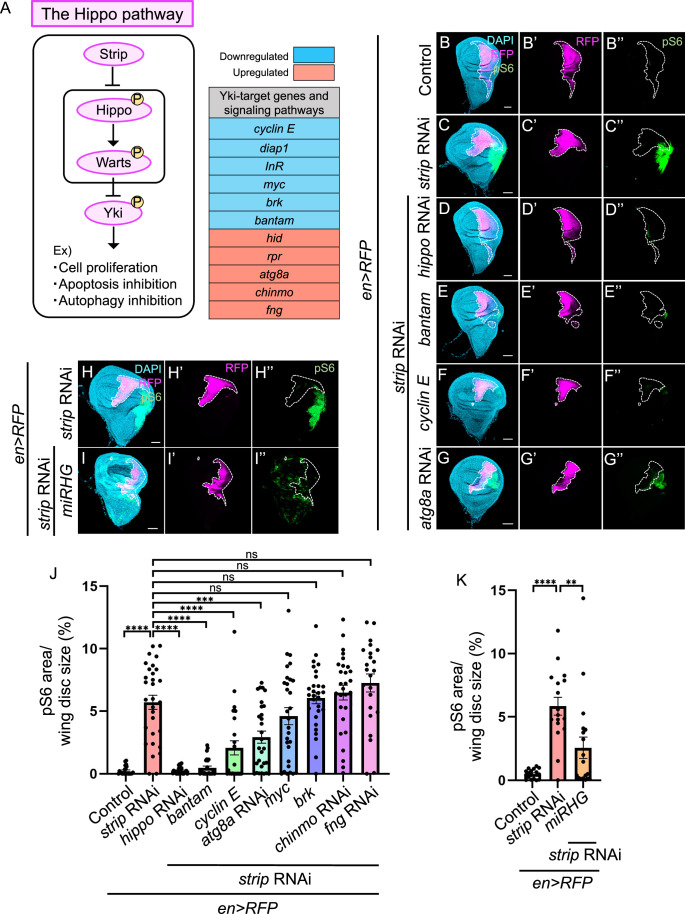
Hippo activation induces non-cell-autonomous mTOR activation by regulating several Hippo-target genes. (**A**) The left schematic depiction indicates the Hippo pathway and its function. The right table shows the Hippo/Yki-target genes and signaling pathways. The blue- and red-colored genes are downregulated and upregulated, respectively, when the Hippo pathway is activated. (**B**–**I**) Confocal images showing the wing discs bearing wild-type or *strip-*knockdown cells with knockdown of *hippo, atg8a*, *rpr, hid, grim*, or overexpression of *bantam*, or *cyclin E*, marked with RFP expression (magenta) and stained with anti-phospho-S6 (green). RFP (magenta) outlined by white dashed lines marks the expression pattern of *en-Gal4* in the wing disc. Knockdown of *rpr, hid*, and *grim* was conducted by expressing microRNAs for *rpr*, *hid*, and *grim* (*miRHG*). (**J**, **K**) Quantification of the phospho-S6 positive region size (% of phospho-S6 positive area/disc area) in the wing disc-bearing wild-type or *strip-*knockdown cells with the knockdown of *hippo, atg8a, rpr, hid, grim*, *chinmo*, and *fng*, or overexpression of *bantam*, *cyclin E*, *myc*, or *brk*. ns, not significant; ^∗∗^*P* < 0.01; ^∗∗∗^*P* < 0.001; ^∗∗∗∗^*P* < 0.0001; one-way ANOVA with Dunnett’s multiple comparison test. Sample size and *P* value in (**J**): *n* = 23, *P* = 7.76 × 10^−12^ (*en* > *RFP*), *n* = 30 (*en>strip* RNAi), *n* = 25, *P* = 2.87 × 10^−12^ (*en>strip* RNAi, *hippo* RNAi), *n* = 25, *P* = 2.18 × 10^−11^ (*en>strip* RNAi, *bantam*), *n* = 26, *P* = 4.10 × 10^−6^ (*en>strip* RNAi, *cyclin E*), *n* = 29, *P* = 5.34 × 10^−4^ (*en>strip* RNAi, *atg8a* RNAi), *n* = 29, *P* = 4.96 × 10^−1^ (*en>strip* RNAi, *myc*), *n* = 29, *P* = 9.99 × 10^−1^ (*en>strip* RNAi, *brk*), *n* = 28, *P* = 8.55 × 10^−1^ (*en>strip* RNAi, *chinmo* RNAi), *n* = 23, *P* = 2.05 ×  10^−1^ (*en>strip* RNAi, *fng* RNAi). Sample size and *P* value in (**K**): *n* = 17, *P* = 1.56 × 10^−6^ (*en-RFP*), *n* = 17 (*en>strip* RNAi), *n* = 19, *P* = 1.64 × 10^−3^ (*en>strip* RNAi, *miRHG*). Scale bars represent 50 μm in (**B**–**I**). Dots represent biological replicates (**J**, **K**); error bars indicate SEM. Source data are available online for this figure.

We investigated the mechanism by which the activated Hippo pathway induced non-cell-autonomous tumorigenesis. Given that the Hippo pathway regulates the expression of multiple genes (Udan et al, 2003; Ziosi et al, 2010; Thompson and Cohen, 2006), we investigated which target genes were crucial for mTOR activation mediated by the Hippo pathway. To this end, we overexpressed or knocked down previously reported Hippo/Yki target genes (Fig. 3A; Udan et al, 2003; Ziosi et al, 2010; Thompson and Cohen, 2006; Kowalczyk et al, 2022; Wang et al, 2020; Seo et al, 2023). We found that *bantam* overexpression, *cyclin E* overexpression, *atg8a* knockdown, or knockdown of *rpr*, *hid*, and *grim* suppressed the non-cell-autonomous mTOR activation caused by Hippo activation (Fig. 3B–I, quantified in J and K). In contrast, the expression of other Hippo-target genes, *myc, brk*, *chinmo*, *fng*, *diap1*, and *InR*, was not involved in non-cell-autonomous mTOR activation (Appendix Fig. S2A–G, quantified in Fig. 3J and Appendix Fig. S2H). These results indicate that Cyclin E (a proliferative activator), Atg8a (an autophagy-related gene), Rpr, Hid, and Grim (apoptosis inducers), and *bantam* (a regulator of cell proliferation, autophagy, and apoptosis) (Udan et al, 2003; Ziosi et al, 2010; Thompson and Cohen, 2006; Texada et al, 2019; Brennecke et al, 2003) are involved in non-cell-autonomous mTOR activation caused by Hippo-activated cells.

### Hippo activation induces non-cell-autonomous mTOR activation through Dronc–Wg/Spitz signaling

**Figure 4:**
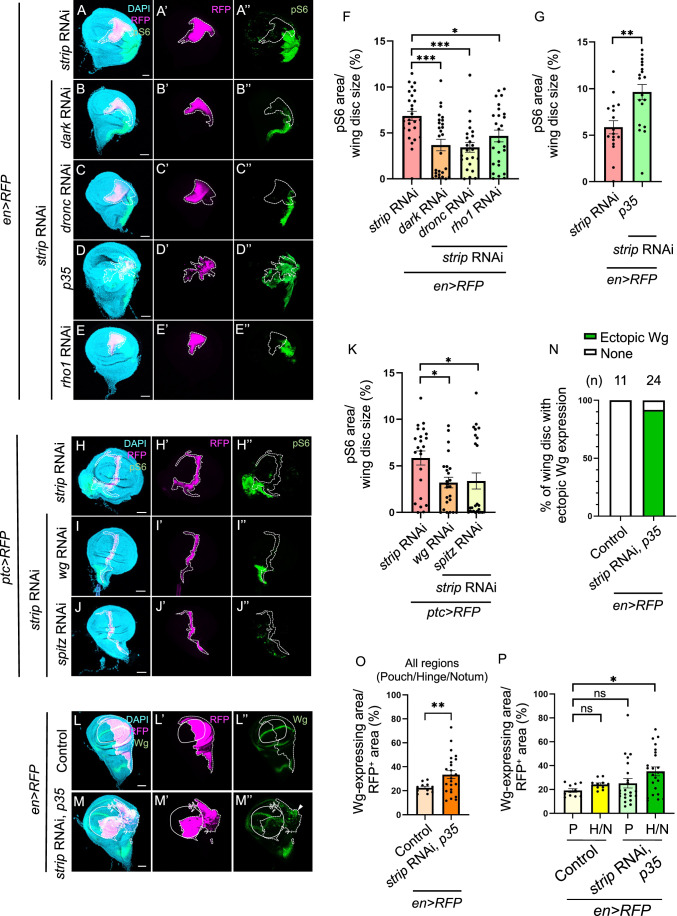
Hippo activation induces mTOR-activated tumors through Dronc–Wg/Spitz signaling. (**A**–**E**) Confocal images showing wing discs bearing *strip-*knockdown cells with or without *dark*, *dronc*, *rho1* knockdown, or *p35* overexpression, marked with RFP expression (magenta) and stained with anti-phospho-S6 (green). RFP (magenta), outlined by white dashed lines, marks the expression pattern of *en-Gal4* in the wing disc. (**F**, **G**) Quantification of the size of the phospho-S6 positive region (% of phospho-S6 positive area/disc area) in wing discs bearing wild-type, *strip-*knockdown cells with or without *dark*, *dronc*, *rho1* knockdown, or *p35* overexpression. ^∗^*P* < 0.05; ^∗∗∗^*P* < 0.001; one-way ANOVA with Dunnett’s multiple comparison test for (**F**). **P < 0.01; unpaired *t* test for (**G**). Sample size and *P* value in (**F**): *n* = 27 (*en>strip* RNAi), *n* = 27, *P* = 4.26 × 10^−4^ (*en>strip* RNAi, *dark* RNAi), *n* = 24, *P* = 2.13 × 10^−4^ (*en>strip* RNAi, *dronc* RNAi), *n* = 26, *P* = 2.24 × 10^−2^ (*en>strip* RNAi, *rho1* RNAi). Sample size and *P* value in (**G**): *n* = 17 (*en>strip* RNAi), *n* = 19, *P* = 1.41 × 10^−3^ (*en>strip* RNAi, *p35*). (**H**–**J**) Confocal images showing wing discs bearing *strip-*knockdown cells with or without *wg* or *spitz* knockdown, marked with RFP expression (magenta) and stained with anti-phospho-S6 (green). RFP (magenta), outlined by white dashed lines, marks the expression pattern of *ptc-Gal4* on wing discs. Because the *spitz* knockdown demonstrated a bimodal distribution in the pS6 positive region quantified in (**K**), the representative image (**J**) was selected from the low-value group. (**K**) Quantification of the size of the phospho-S6 positive region (% of phospho-S6 positive area/disc area) in wing discs bearing *strip-*knockdown cells with or without *wg* or *spitz* knockdown. ^*^P < 0.05; one-way ANOVA with Dunnett’s multiple comparison test. Sample size and *P* value: *n* = 23 (*ptc>strip* RNAi), *n* = 24, *P* = 2.97 × 10^−2^ (*ptc>strip* RNAi, *wg* RNAi), *n* = 25, *P* = 4.13 × 10^−2^ (*ptc>strip* RNAi, *spitz* RNAi). (**L**, **M**) Confocal images showing wing discs bearing wild-type or *strip-*knockdown cells with *p35* overexpression marked with RFP (magenta) and stained with anti-Wg (green). RFP (magenta), outlined by white dashed lines, marks the expression pattern of *en-Gal4* in the wing disc. Solid lines outline the pouch region. White arrowheads indicate ectopic expression of Wg. (**N**) Incidence of ectopic Wg expression. *n* = 11 (*en* > *RFP*), *n* = 24 (*en > strip* RNAi, *p35*). (**O**, **P**) Quantification of the Wg-expressing area (% of Wg-expressing area/RFP-positive area) in wing discs bearing wild-type or *strip-*knockdown cells overexpressing p35. As *en-Gal4* driver was active in the pouch, hinge, and ventral notum, the RFP-positive area could be separated into two areas: the pouch and hinge/ventral notum regions. Quantification was conducted in the RFP-positive areas of all regions (pouch/hinge/ventral notum region), pouch (P), or hinge/ventral notum region (H/N). ^∗∗^*P* < 0.01; Welch’s *t* test for (**O**). ns, not significant; ^*^P < 0.05; one-way ANOVA with Dunnett’s multiple comparison test for (**P**). Sample size and *P* value in (**O**): *n* = 11 (*en* > *RFP*), *n* = 24, *P* = 4.07 × 10^−3^ (*en>strip* RNAi, *p35*). Sample size and *P* value in (**P**): *n* = 11 (pouch in *en* > *RFP*), *n* = 11, *P* = 7.51 × 10^−1^ (hinge/notum in *en* > *RFP*), *n* = 22, *P* = 5.49 × 10^−1^ (pouch in *en> strip* RNAi, *p35*), *n* = 24, *P* = 1.49 × 10^−2^ (hinge/notum in *en> strip* RNAi, *p35*). Scale bars represent 50 μm in (**A**–**E**, **H**–**J**, **L**, **M**). Dots represent biological replicates (**F**, **G**, **K**, **O**, **P**); numbers indicate the number of biological replicates (*N*); error bars indicate SEM. Source data are available online for this figure.

The Hippo pathway drives apoptotic signaling through *rpr* and *hid* expression (Appendix Fig. S3A; Udan et al, 2003; Zhang and Cohen, 2013). Because these apoptotic signaling components are crucial for mTOR activation (Fig. 3H,I, quantified in K), we examined how apoptotic signaling is involved in mTOR activation. Rpr, Hid, and Grim activate the effector caspase Drice/Dcp-1 for apoptosis via Diap1, and the initiator caspase Dronc, which forms an apoptosome with Dark for activation. (Appendix Fig. S3A, Shinoda and Miura 2024). We confirmed that Hippo activation by *strip* RNAi drives Drice/Dcp-1 activation through Rpr, Hid, and Grim (Appendix Fig. S3B–D, quantified in F). Next, we investigated the involvement of Dark, Dronc, and Drice/Dcp1 as downstream components of Rpr, Hid, and Grim, respectively. The knockdown of *dark* or *dronc* significantly suppressed non-cell-autonomous mTOR activation (Fig. 4A–C, quantified in F). In contrast, the expression of *p35*, a Drice/Dcp1 inhibitor, promoted mTOR activation (Fig. 4D, quantified in G), although *p35* expression significantly suppressed activated Drice/Dcp1 levels (Appendix Fig. S3E, quantified in F). Therefore, we expected that the Hippo pathway would activate Dronc, which induces non-cell-autonomous mTOR activation, but not through Drice/Dcp1. A previous study demonstrated that Dronc regulates not only Drice/Dcp1 for apoptosis but also Rho1–Wg/Spitz signaling for AiP, a phenomenon in which undead cells promote cell proliferation in the live surrounding cells (Fan et al, 2014; Bergmann and Fan, 2025). We knocked down *rho1*, *wg*, or *spitz* in Hippo-activated cells to investigate the involvement of Rho1–Wg/Spitz signaling. We found that the non-cell-autonomous mTOR activation was significantly suppressed (Fig. 4E,H–J, quantified in F and K). Moreover, Wg was expressed in different patterns and was ectopically expressed following Hippo activation compared with the control (Fig. 4L,M, quantified in N, O). Wg expression was significantly increased by Hippo activation in the hinge/ventral notum but not in the pouch region (Fig. 4P). These data indicated that Hippo-activated cells would drive Dronc-Rho1 signaling to promote the expression of *wg*/*spitz*, especially in the hinge/ventral notum region, for non-cell-autonomous mTOR activation.

**Figure 5:**
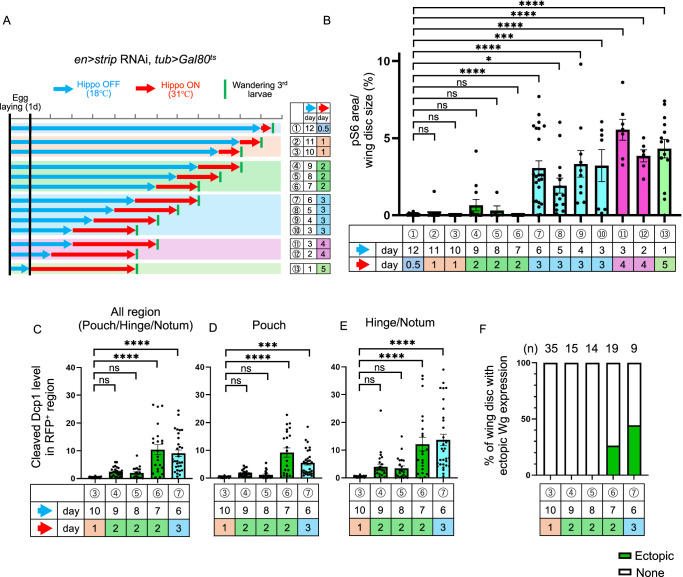
Dronc activation and ectopic Wg expression occur before emerging mTOR-activated tumors. (**A**) Schematic representation of the heat shock schedule used for *strip* knockdown using *en-Gal4*. Blue and red arrows denote the time post-egg laying at 18 °C (Hippo OFF) and the day of heat shock at 31 °C (Hippo ON), respectively. (**B**) Quantification of the phospho-S6-positive area in the wing disc bearing *strip*-knockdown cells induced under *Gal80*^*ts*^ control. The phospho-S6-positive region is shown as a percentage of the total disc area. ns, not significant; ∗*P* < 0.05; ∗∗∗*P* < 0.001; ∗∗∗∗*P* < 0.0001; one-way ANOVA with Dunnett’s multiple comparison test. Sample size and *P* value: *n* = 12 (①AEL: HS = 12:0.5), *n* = 17, *P* = 1.00 (②AEL: HS = 11:1), *n* = 23, *P* = 1.00 (③AEL: HS = 10:1), *n* = 12, *P* = 9.59 × 10^−1^ (④AEL: HS = 9:2), *n* = 5, *P* = 1.00 (⑤AEL: HS = 8:2), *n* = 22, *P* = 1.00 (⑥AEL: HS = 7:2), *n* = 24, *P* = 1.89 × 10^−6^ (⑦AEL: HS = 6:3), *n* = 14, *P* = 2.41 × 10^−2^ (⑧AEL: HS = 5:3), *n* = 10, *P* = 2.42 × 10^−5^ (⑨AEL: HS = 4:3), *n* = 7, *P* = 3.39 × 10^−4^ (⑩AEL: HS = 3:3), *n* = 7, *P* = 7.63 × 10^−11^ (⑪AEL: HS = 3:4), *n* = 6, *P* = 2.84 × 10^−5^ (⑫AEL: HS = 2:4), *n* = 14, *P* = 9.08 × 10^−10^ (⑬AEL: HS = 1:5). (**C**–**E**) Quantification of cleaved Drice/Dcp1-positive region in *strip-*knockdown cells marked by RFP, with temporal control via Gal80^ts^. The *en-Gal4* driver drives expression in the pouch, hinge, and ventral notum regions, allowing separation of the RFP-positive area into two subregions: pouch and hinge/ventral notum. Quantification was performed for the entire RFP-positive region (pouch/hinge/ventral notum), as well as each subregion individually. ns, not, significant; ∗∗∗*P* < 0.001; ∗∗∗∗*P* < 0.0001; one-way ANOVA with Dunnett’s multiple comparison test. Sample size and *P* value in (**C**): *n* = 20 (③AEL: HS = 10:1), *n* = 26, *P* = 4.18 × 10^−1^ (④AEL: HS = 9:2), *n* = 21, *P* = 6.57 × 10^−1^ (⑤AEL: HS = 8:2), *n* = 22, *P* = 1.20 × 10^−8^ (⑥AEL: HS = 7:2), *n* = 31, *P* = 8.37 × 10^−8^ (⑦AEL: HS = 6:3). Sample size and *P* value in (**D**): *n* = 20 (③AEL: HS = 10:1), *n* = 26, *P* = 6.27 × 10^−1^ (④AEL: HS = 9:2), *n* = 21, *P* = 9.23 × 10^−1^ (⑤AEL: HS = 8:2), *n* = 22, *P* = 1.15 × 10^−9^ (⑥AEL: HS = 7:2), *n* = 31, *P* = 1.12 × 10^−4^ (⑦AEL: HS = 6:3). Sample size and *P* value in (**E**): *n* = 20 (③AEL: HS = 10:1), *n* = 26, *P* = 3.47 × 10^−1^ (④AEL: HS = 9:2), *n* = 21, *P* = 5.28 × 10^−1^ (⑤AEL: HS = 8:2), *n* = 22, *P* = 2.63 × 10^−5^ (⑥AEL: HS = 7:2), *n* = 31, *P* = 3.16 × 10^−7^ (⑦AEL: HS = 6:3). (**F**) Frequency of ectopic Wg expression at different heat shock timings. Sample size: *n* = 35 (③AEL: HS = 10:1), *n* = 15 (④AEL: HS = 9:2), *n* = 14 (⑤AEL: HS = 8:2), *n* = 19 (⑥AEL: HS = 7:2), *n* = 9 (⑦AEL: HS = 6:3). *en-Gal4* was used for *strip* knockdown. Dots represent biological replicates (**B**–**E**); numbers indicate the number of biological replicates (**F**). Source data are available online for this figure.

**Figure EV4:**
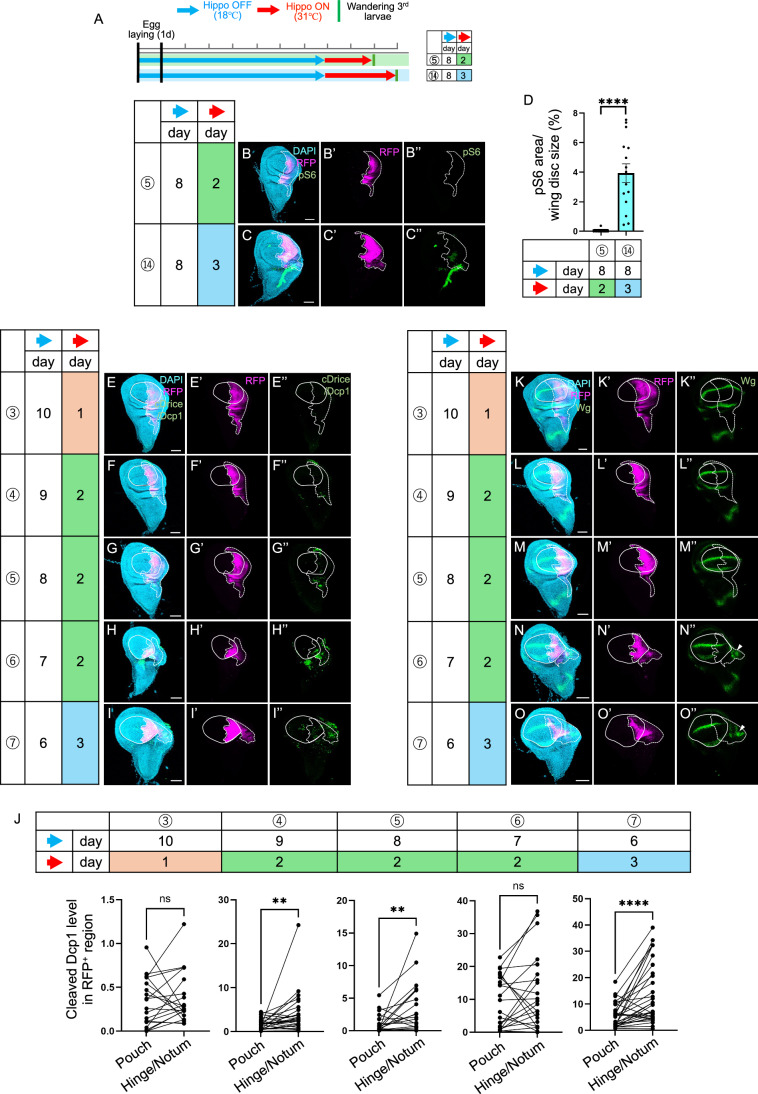
The activation of Dronc and ectopic expression of Wg are predominantly observed in the hinge and ventral notum regions. (**A**) The schematic representation illustrates the temporal schedule for heat shock in the context of *strip* knockdown. Blue and red arrows denote the time post-egg laying at 18 °C (Hippo OFF) and the day of heat shock at 31 °C (Hippo ON), respectively. (**B**, **C**) Confocal images displaying wing discs containing *strip*-knockdown cells, under *Gal80*^*ts*^ control, marked by RFP expression (magenta) and stained with anti-phospho-S6 (green). The RFP (magenta), delineated by white dashed lines, indicates the expression pattern of *en-Gal4* within the wing disc. (**D**) Quantification of the phospho-S6 positive region size (% of phospho-S6 positive area/disc area) in wing discs with *strip-*knockdown cells, whose induction was controlled by *Gal80ts*. ∗∗∗∗*P* < 0.0001; Welch’s *t* test. Sample size and *P* value: *n* = 14 (⑤AEL: HS = 8:2), *n* = 15, *P* = 2.10 × 10^−5^ (⑭AEL: HS = 8:3). (**E**–**I**) Confocal images depicting wing discs with *strip*-knockdown cells, regulated by *Gal80*^*ts*^, marked by RFP expression (magenta) and stained with anti-cleaved Drice/Dcp1 (green). The RFP (magenta), outlined by white dashed lines, marks the *en-Gal4* expression pattern in the wing disc. Solid lines demarcate the pouch region. (**J**) A comparison of quantification data for the cleaved Drice/Dcp1-positive region (% of anti-cleaved Drice/Dcp1-positive area relative to RFP-positive area) between the pouch and hinge/ventral notum regions in wing discs containing *strip*-knockdown cells under *Gal80*^*ts*^ control. ∗∗*P* < 0.01; ^∗∗∗∗^*P* < 0.0001; Wilcoxon matched-pairs signed rank test. Sample size and *P* value: *n* = 20, *P* = 3.49 × 10^−1^ (③AEL: HS = 10:1), *n* = 26, *P* = 5.1 × 10^−3^ (④AEL: HS = 9:2), *n* = 21, *P* = 4.88 × 10^−3^ (⑤AEL: HS = 8:2), *n* = 22, *P* = 1.29 × 10^−1^ (⑥AEL: HS = 7:2), *n* = 31, *P* = 5.61 × 10^−5^ (⑦AEL: HS = 6:3). (**K**–**O**) Confocal images illustrating wing discs with *strip-*knockdown cells, regulated by *Gal80*^*ts*^, marked by RFP expression (magenta) and stained with anti-Wg (green). The RFP (magenta), outlined by white dashed lines, indicates the *en-Gal4* expression pattern in the wing disc. Solid lines demarcate the pouch region. Scale bars represent 50 μm in (**B**, **C**, **E**–**I**, **K**–**O**). Dots represent biological replicates (**D**, **J**); error bars indicate SEM.

Because Dronc–Wg/Spitz signaling is required for non-cell-autonomous mTOR activation, we investigated whether Dronc activation and Wg expression were upregulated before the emergence of mTOR-activated cell clusters. We temporally varied the duration of Hippo activation using *Gal80*^*ts*^ and assessed the temporal progression of mTOR activation (Fig. 5A). Following a 3-day induction of *strip* RNAi, mTOR activation was observed in adjacent cells, whereas no such activation was detected after 2 days (Fig. 5A, quantified in B; Fig. EV4A–C, quantified in D). The activation of Dronc, indicated by active Drice/Dcp1 (cleaved Dcp1), was observed in both the hinge/notum and pouch regions, with a more pronounced presence in the hinge/notum area 2 days after the induction of *strip*-RNAi (Fig. EV4E–I, quantified in Figs. 5C–E and  EV4J). In addition, increased Wg expression was noted in the hinge/notum region, but not in the pouch area 2 days after *strip* RNAi induction (Fig. EV4K–O, quantified in Fig. 5F). Therefore, the activation of Dronc and the elevated expression of Wg occurred 2 days after Hippo activation and may subsequently activate the mTOR pathway in adjacent cells 3 days later.

### Dronc–Wg/Spitz signaling activation alone is insufficient to induce non-cell-autonomous mTOR activation

**Figure 6:**
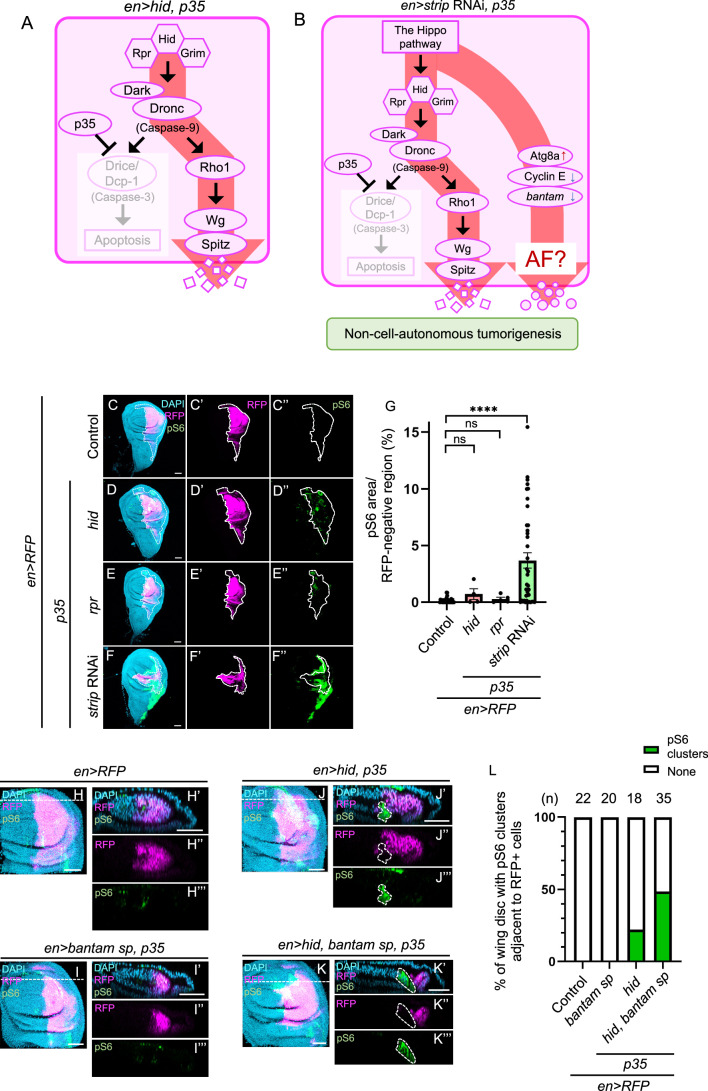
Activation of Dronc–Wg/Spitz signaling alone could not significantly induce non-cell-autonomous mTOR activation. (**A**) The schematic depiction indicates a driven signaling pathway in *hid*-overexpressing cells with *p35* overexpression. (**B**) The schematic depiction indicates a driven signaling pathway in *strip*-knockdown cells with *p35* overexpression. AF: additional factors. (**C**–**F**) Confocal images showing the wing discs bearing wild-type, *strip-*knockdown, *hid*-, or *rpr*-overexpressed cells with *p35* overexpression marked with RFP expression (magenta) and stained with anti-phospho-S6 (green). RFP (magenta) outlined by white dashed lines marks the expression pattern of *en-Gal4* in the wing disc. (**G**) Quantification of the size of the phospho-S6 positive region (% of phospho-S6 positive area/RFP-positive area) in RFP-positive region bearing wild-type, *strip-*knockdown, *hid-*, or *rpr-*overexpressed cells with *p35* overexpression. ns not significant; ∗∗∗∗*P* < 0.0001; one-way ANOVA with Dunnett’s multiple comparison test. Sample size and *P* value: *n* = 27 (*en* > *RFP*), *n* = 4, *P* = 9.78 × 10^−1^ (*en>hid, p35*), *n* = 5, *P* = 9.99 × 10^−1^ (*en>rpr, p35*), *n* = 39, *P* = 6.40 × 10^−5^ (*en>strip* RNAi, *p35*). (**H**–**K**) Confocal images showing the wing discs bearing wild-type, *bantam sp-*, *hid*-overexpressed cells, or concurrently *bantam sp* and *hid*-expressed cells with *p35* overexpression marked with RFP expression (magenta) and stained with anti-phospho-S6 (green). pS6-cell clusters are outlined by white dashed lines. (**L**) Incidence of wing discs with mTOR (phospho-S6)-activated cell clusters adjacent to genetically manipulated cells in the pouch region. *n* = 22 (*en* > *RFP*), *n* = 20 (*en > bantam sp, p35*), *n* = 18 (*en > hid, p35*), *n* = 35 (*en > hid, bantam sp, p35*). Scale bars represent 50 μm in (**C**–**F**, **H**–**K**). Dots represent biological replicates (**G**); numbers indicate the number of biological replicates (**L**); error bars indicate SEM. Source data are available online for this figure.

Because Dronc–Wg/Spitz signaling is used in AiP, we examined whether non-cell-autonomous mTOR activation caused by Hippo activation was simply driven by AiP. Thus, we investigated whether activation of the key signaling pathway for AiP, Dronc–Wg/Spitz signaling, alone was sufficient to induce non-cell-autonomous mTOR activation. To this end, we expressed *hid* or *rpr* with *p35*, which can activate Dronc–Wg/Spitz signaling without inducing apoptosis (Fig. 6A; Ryoo et al, 2004; Fan et al, 2014). These cells unexpectedly induced cell-autonomous mTOR activation, but they did not significantly induce non-cell-autonomous mTOR activation compared to that shown by the Hippo-activated cells (Fig. 6C–F, quantified in G), although *hid*-overexpression with *p35* infrequently induced small mTOR-activated clusters in the pouch region (Fig. 6H,J, quantified in L). These data suggest that the non-cell-autonomous mTOR activation caused by Hippo activation is not simply caused by AiP. Given that additional factors, including *atg8a*, *cyclin E*, and *bantam*, are required for Hippo-mediated mTOR activation (Fig. 3E–G), unknown pathways may be responsible for the secretion of different molecules in addition to Wg/Spitz (Fig. 6B). To support this idea, we concurrently overexpressed *hid* and *bantam sponge (bantam sp: bantam suppressor)*, in conjunction with *p35*, and demonstrated that this combination more frequently induced small mTOR-activated clusters within the pouch region compared to the overexpression of *hid* or *bantam sponge* individually (Fig. 6H–K, quantified in L).

### Amino acid transporters Sat1/2 and Wg/Spitz redundantly function to achieve non-cell-autonomous mTOR activation

**Figure EV5:**
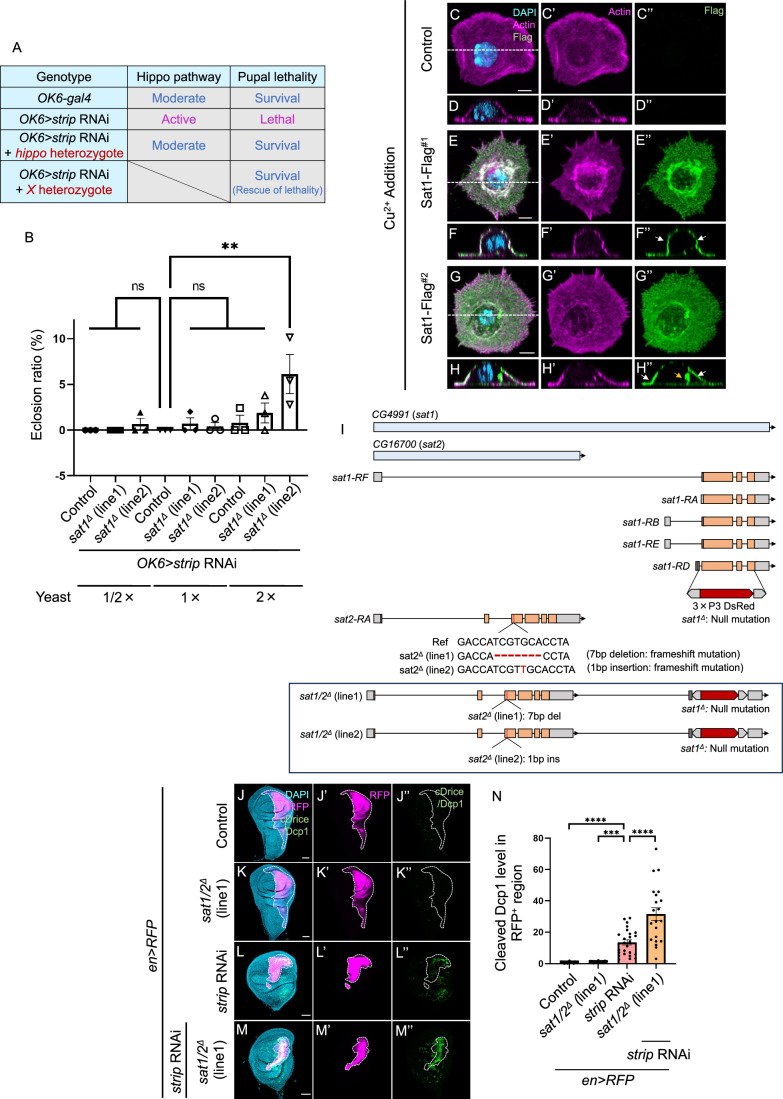
Genomic deficiency screening identifies *sat1/2* and Sat1/2 functions to suppress apoptosis in cells with an activated Hippo pathway. (**A**) The table presents data on Hippo activity and pupal lethality in specimens possessing either wild-type or *strip*-knockdown cells, with or without different gene background mutations. The *strip* knockdown was executed using the *OK6-Gal4* driver for genomic deficiency screening. *X* genes are hypothesized to be genes whose deletion can mitigate pupal lethality induced by Hippo activation. (**B**) The eclosion ratio (percentage of adult flies relative to the number of pupae) is quantified in individuals with wild-type or *strip*-knockdown cells, with or without the *sat1* background mutation. These individuals were provided with diets containing different yeast concentrations (normal, 50%, or 200% yeast concentration). ns, not significant; ^∗∗^*P* < 0.01; one-way ANOVA with Dunnett’s multiple comparison test. Sample size and *P* value: *n* = 3, *P* = 1.00 (*OK6>strip* RNAi in 50% yeast), *n* = 3, *P* = 1.00 (*sat1* mutant line1; *OK6>strip* RNAi in 50% yeast), *n* = 3, *P* = 9.97 ×  10^−1^ (*sat1* mutant line2; *OK6>strip* RNAi in 50% yeast), *n* = 3 (*OK6>strip* RNAi in 100% yeast), *n* = 3, *P* = 9.96 × 10^−1^ (*sat1* mutant line1; *OK6>strip* RNAi in 100% yeast), *n* = 3, *P* = 1.00 (*sat1* mutant line2; *OK6>strip* RNAi in 100% yeast), *n* = 3, *P* = 9.89 × 10^−1^ (*OK6>strip* RNAi in 200% yeast), *n* = 3, *P* = 5.95 ×  10^−1^ (*sat1* mutant line1; *OK6>strip* RNAi in 200% yeast), *n* = 3, *P* = 1.07 × 10^−3^ (*sat1* mutant line2; *OK6>strip* RNAi in 200% yeast). (**C**–**H**) Confocal microscopy images depict S2 cells expressing a Flag-tagged Sat1 with a copper-inducible promoter. The addition of Cu^2+^ activates the copper-inducible promoter, leading to the overexpression of Flag-tagged Sat1. Sat1-Flag#1 and Sat1-Flag#2 refer to S2 cells (line1) and (line2) containing *pMT-Flag-sat1-puro*, respectively. Cell outlines are stained with TRITC-phalloidin, and Sat1 expression is indicated by anti-Flag staining (green). (**D**, **F**, **H**) Vertical sections at a site indicated by a dashed line in (**C**, **E**, **G**). The white arrows indicate Sat1 localization at the plasma membrane, while the yellow arrow indicates intracellular localization of Sat1. Scale bars represent 5 μm in (**C**, **E**, **G**). (**I**) Schematic illustrations depict the *CG4991* (*sat1*) and *CG16700* (*sat2*) genes and the mutated site of *sat1/2*. The coding region of the *sat1* gene was replaced by the marker gene *3xP3 DsRed*. The coding region of the *sat2* gene was altered with a deletion (line 1) or an insertion (line 2). *sat1/2* double mutant bears a *sat1* null mutation combined with a deletion or insertion mutation in *sat2*. (**J**–**M**) Confocal images displaying wing discs containing wild-type or *strip*-knockdown cells, with or without the *sat1/2* background mutations, marked with RFP expression (magenta) and stained with anti-Drice/Dcp1 (green). RFP (magenta) marks the expression pattern of *en-Gal4* in the wing disc. The male animals of these strains were selected for the experiment, and the *sat1/2* background mutations were hemizygous. (**N**) The size of the cleaved Drice/Dcp1-positive region is quantified (% of anti-cleaved Drice/Dcp1-positive area/RFP-positive area) in the wing disc containing wild-type or *strip*-knockdown cells, with or without the *sat1/2* background mutations. ^∗∗∗^*P* < 0.001; ^∗∗∗∗^*P* < 0.0001; one-way ANOVA with Dunnett’s multiple comparison test. Sample size and *P* value: *n* = 23, *P* = 8.43 × 10^−5^ (*en* > *RFP*), *n* = 22, *P* = 1.40 × 10^−4^ (*sat1/2* mutant line1, *en* > *RFP*), *n* = 25 (*en>strip* RNAi), *n* = 21, *P* = 6.04 × 10^−8^ (*sat1/2* mutant line1, *en>strip* RNAi). Scale bars represent 50 μm in (**J**–**M**). Dots represent technical (**B**) or biological (**N**) replicates; error bars indicate SEM.

To identify additional or previously unknown factors involved in the regulation of mTOR activation by Hippo activation, we conducted a genetic modifier screen using chromosomal deficiencies to identify genes that interact with the Hippo pathway. We utilized the pupal lethality phenotype caused by Hippo activation in motor neurons (*OK6-Gal4>strip* RNAi) as a readout (Fig. EV5A). This screen identified a previously uncharacterized putative amino acid transporter, *CG4991*, hereafter referred to as *strip-interacting amino acid transporter 1* (*sat1*), whose deficiency partially rescued lethality, depending on the yeast concentration (Fig. EV5A, quantified in B).

**Figure 7:**
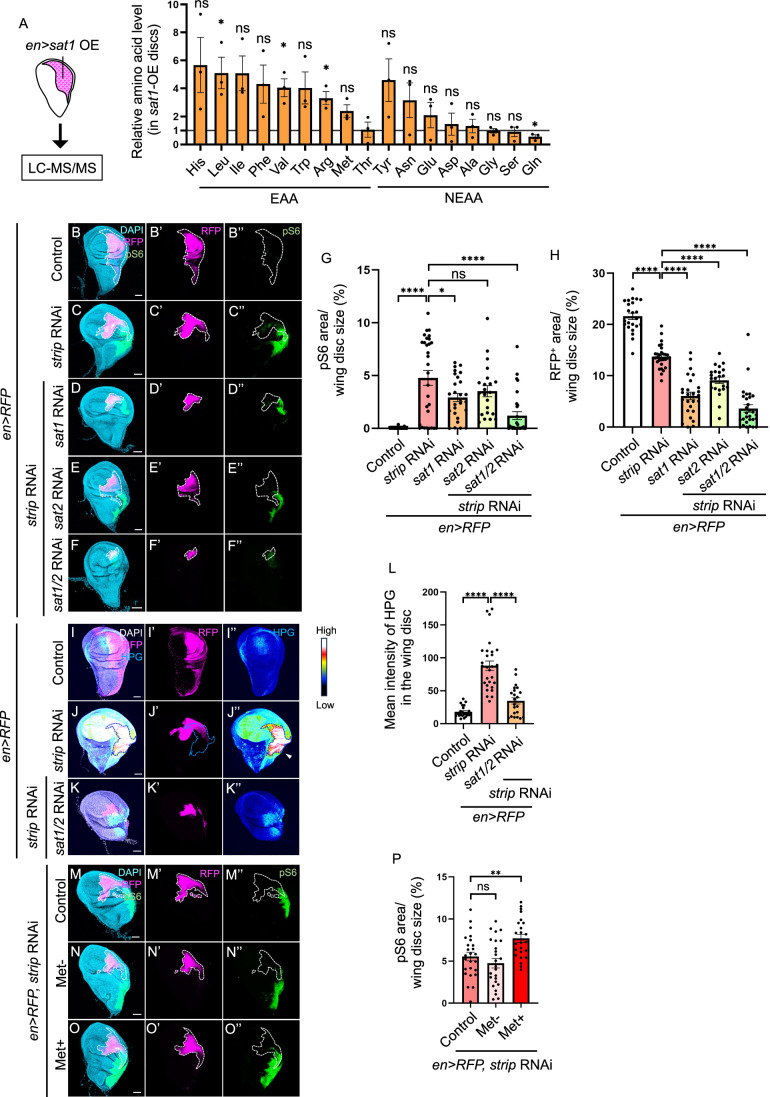
Hippo activation induces mTOR-activated tumors through the amino acid transporter *sat1/2.* (**A**) Quantification of amino acid levels in the wing discs bearing wild-type or *sat1*-overexpressed cells using *en-Gal4*. Amino acid levels of *sat1* overexpression are presented as fold changes relative to the control group. EAA: essential amino acids. NEAA: non-essential amino acids. Certain amino acids below the detection limit are not listed. ns, not significant; ∗*P* < 0.05; Welch’s *t* test or unpaired *t* test. Sample size and *P* value: *n* = 3, *P* = 1.40 × 10^−1^ (His), *n* = 3, *P* = 2.36 × 10^−2^ (Leu), *n* = 3, *P* = 7.83 × 10^−2^ (Ile), *n* = 3, *P* = 7.48 × 10^−2^ (Phe), *n* = 3, *P* = 1.21  × 10^−2^ (Val), *n* = 3, *P* = 6.30 × 10^−2^ (Trp), *n* = 3, *P* = 1.30 × 10^−2^ (Arg), *n* = 3, *P* = 7.15 × 10^−2^ (Met), *n* = 3, *P* = 9.23 × 10^−1^ (Thr), *n* = 3, *P* = 8.29 × 10^−2^ (Tyr), *n* = 3, *P* = 1.64 × 10^−1^ (Asn), *n* = 3, *P* = 2.96 × 10^−1^ (Glu), *n* = 3, *P* = 6.05 × 10^−1^ (Asp), *n* = 3, *P* = 5.98  × 10^-1^ (Ala), *n* = 3, *P* = 9.51 × 10^−1^ (Gly), *n* = 3, *P* = 8.54 × 10^−1^ (Ser), *n* = 3, *P* = 4.66 × 10^−2^ (Gln). (**B**–**F**) Confocal images showing the wing discs bearing wild-type or *strip-*knockdown cells with or without *sat1*, *sat2*, or *sat1/2* knockdown marked with RFP expression (magenta) and stained with anti-phospho-S6 (green). RFP (magenta) outlined by white dashed lines marks the expression pattern of *en-Gal4* in the wing disc. (**G**) Quantification of the size of the phospho-S6 positive region (% of phospho-S6 positive area/disc area) in the wing disc bearing wild-type or *strip-*knockdown cells with or without *sat1*, *sat12*, or *sat1/2* knockdown. ns, not significant; ^∗^*P* < 0.05; ^∗∗∗∗^*P* < 0.0001; one-way ANOVA with Dunnett’s multiple comparison test. Sample size and *P* value: *n* = 23, *P* = 9.31 × 10^−10^ (*en* > *RFP*), *n* = 30 (*en>strip* RNAi), *n* = 27, *P* = 2.03 × 10^−2^ (*en>strip* RNAi, *sat1* RNAi), *n* = 22, *P* = 2.27 × 10^−1^ (*en>strip* RNAi, *sat*2 RNAi), *n* = 29, *P* = 7.33 × 10^−7^ (*en>strip* RNAi, *sat1/2* RNAi). (**H**) Quantification of the RFP-positive region (expressed as % of total disc area) in wing disc bearing the wild-type or *strip*-knockdown cells, with or without additional knockdown of *sat1*, *sat2*, or *sat1/2* knockdown. ns, not significant; ^∗∗∗∗^*P* < 0.0001; one-way ANOVA with Dunnett’s multiple comparison test. Sample size and *P* value: *n* = 23, *P* = 3.5 × 10^−14^ (*en* > *RFP*), *n* = 30 (*en>strip* RNAi), *n* = 27, *P* = 1.8 × 10^−14^ (*en>strip* RNAi, *sat1* RNAi), *n* = 22, *P* = 6.20 × 10^−6^ (*en>strip* RNAi, *sat2* RNAi), *n* = 29, *P* < 1 × 10^−15^ (*en>strip* RNAi, *sat1/*2 RNAi). (**I**–**K**) Confocal images showing the wing discs bearing wild-type or *strip-*knockdown cells with or without *sat1/2* knockdown marked with RFP expression (magenta) and cultured with a methionine analog homopropargylglycine (HPG) for visualizing methionine incorporation. The blue dashed line marks the high HPG incorporation area, represented by a rainbow LUT. The white arrowhead indicates a high-intensity area of non-cell-autonomous HPG incorporation. (**L**) Quantification of the HPG intensity (HPG intensity/disc area [pixels]) in the wing disc bearing wild-type or *strip-*knockdown cells with or without *sat1/2* knockdown. ^∗∗∗∗^*P* < 0.0001; one-way ANOVA with Dunnett’s multiple comparison test. Sample size and *P* value: *n* = 24, *P* = 9.1 × 10^−14^ (*en* > *RFP*), *n* = 28 (*en>strip* RNAi), *n* = 22, *P* = 2.41 × 10^−9^ (*en>strip* RNAi, *sat1/*2 RNAi). (**M**–**O**) Confocal images showing the wing discs bearing *strip-*knocked down cells marked with RFP expression (magenta) and stained with anti-phospho-S6 (green). These individuals for analysis were fed a holidic diet with varying concentrations of methionine (normal, 50% met, or 200% met concentration). (**P**) Quantification of the size of the phospho-S6 positive region (% of phospho-S6 positive area/disc area) in the wing disc bearing *strip-*knocked down cells in individuals fed a holidic diet with varying concentrations of methionine. ns, not significant; ^∗∗^*P* < 0.01; one-way ANOVA with Dunnett’s multiple comparison test. Sample size and *P* value: *n* = 25 (Control), *n* = 25, *P* = 4.48 × 10^−1^ (Met − ), *n* = 24, *P* = 8.85 × 10^−3^ (Met + ). Scale bars represent 50 μm in (**B**–**F**, **I**–**K**, **M**–**O**). Dots represent technical (**A**) or biological (**G**, **H**, **L**, **P**) replicates; error bars indicate SEM. Source data are available online for this figure.

Based on sequence similarity, Sat1 can be classified as a member of the solute carrier (SLC) 36 A family, a proton-coupled amino acid transporter. The SLC36A family has been reported to transport certain amino acids, such as proline and alanine; however, their detailed functions are not fully understood (Thwaites and Anderson, 2011). The substrates and localization of SLC36A transporters are distinct among family members and vary among tissues. We investigated the subcellular localization of Sat1 in *Drosophila* S2 cells to elucidate its functions. We overexpressed FLAG-tagged Sat1 in these cells and observed that Sat1-FLAG was predominantly localized to the plasma membrane with some intracellular distribution (Fig. EV5C–H). Furthermore, we observed an increase in the number of amino acids within the wing disc upon Sat1 overexpression in the wing disc (Fig. 7A). These findings indicated that Sat1 regulates amino acid levels in wing disc cells. Moreover, within the *sat1* genomic locus, we identified another uncharacterized putative amino acid transporter, *CG16700*, which we designated *sat2* (Fig. EV5I). *Drosophila sat1* and *sat2* are homologous to members of the human SLC36A1–4 family. As the two genes share a common 5′ UTR and encode proteins with high sequence similarity (Fig. EV5I; Appendix Fig. S4A), they were likely to function redundantly.

We knocked down *sat1/2* in Hippo-activated cells to investigate the association between *sat1/2* and mTOR activation caused by Hippo activation. Knockdown of *sat1* in Hippo-activated cells significantly suppressed non-cell-autonomous mTOR activation, whereas *sat2* knockdown exhibited only a mild effect (Fig. 7B–E, quantified in G). The combined knockdown of *sat1* and *sat2* led to greater suppression of mTOR activation than either single knockdown (Fig. 7B–F, quantified in G), suggesting *sat1/2* redundantly regulates mTOR activation in a non-cell-autonomous manner.

Because amino acids are established activators of the mTOR pathway (Laplante and Sabatini, 2012), it is plausible that mTOR-activated tumors arise due to amino acid stimulation. To investigate the association between mTOR activation and amino acids, we employed the methionine analog, homopropargylglycine (HPG), incorporation, which was previously utilized as an indicator of methionine uptake (Nishida et al, 2021; Obata et al, 2018). HPG incorporation was non-cell autonomously enhanced by Hippo activation (Fig. 7I,J, quantified in L). Moreover, a methionine-enriched diet caused the enlargement of mTOR-activated cell clusters, although methionine depletion did not reduce the mTOR-activated cell clusters (Fig. 7M–O, quantified in P). These data suggest that Hippo-activated cells can regulate methionine uptake and metabolism to non-autonomously activate the mTOR pathway. Because HPG incorporation promoted by Hippo activation was inhibited by *sat1/2* knockdown (Fig. 7I–K, quantified in L), *sat1/2* non-cell-autonomously regulates methionine uptake/metabolism for mTOR activation.

Single or double knockdown of *sat1* and *sat2* reduced the autonomous growth of Hippo-activated cells, with double knockdown producing a more pronounced effect (Fig. 7H). The magnitude of cell-autonomous suppression was correlated with the extent of non-cell-autonomous mTOR inhibition (Fig. 7G,H). Because *sat1/2* double mutants (Fig. EV5I) enhance Drice/Dcp-1 activation in Hippo-activated cells (Fig. EV5J–M, quantified in N), Sat1/2 might contribute to the survival of Hippo-activated cells. Based on these findings, we propose two mechanisms. First, Sat1/2 may promote the survival of Hippo-activated cells by facilitating the import of amino acids, thereby increasing the number of Wg/Spitz-secreting cells, which, consequently, promotes non-cell-autonomous methionine incorporation and mTOR activation. Alternatively, Sat1/2 may import amino acids, which Hippo-activated cells release into the microenvironment, thereby stimulating both cell-autonomous and non-cell-autonomous growth. In both scenarios, Sat1/2 appear to facilitate the production or release of tumorigenic signals, acting as novel regulators of the Hippo-tumor axis.

**Figure 8:**
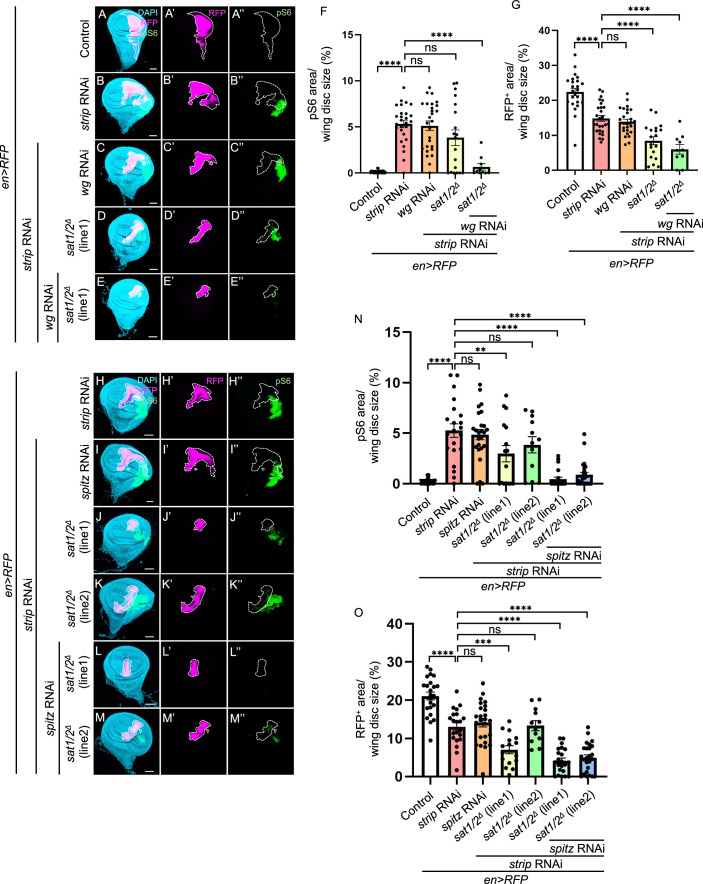
Sat1/2 redundantly work with growth factors Wg and Spitz. (**A**–**E**) Confocal images showing wing discs bearing wild-type or *strip-*knockdown cells with or without *wg* knockdown alone, or *wg* knockdown with *sat1/2* background mutations marked with RFP expression (magenta) and stained with anti-phospho-S6 (green). Male animals of these strains were selected for the experiment, and the *sat1/2* background mutations were hemizygous. RFP (magenta), outlined by white dashed lines, marks the expression pattern of *en-Gal4* in the wing disc. (**F**) Quantification of the size of the phospho-S6 positive region (% of phospho-S6 positive area/disc area) in wing discs bearing wild-type, *strip-*knocked down cells with or without *wg* knockdown alone, or *wg* knockdown with the *sat1/2* background mutations. ns, not significant; ^∗∗∗∗^*P* < 0.0001; one-way ANOVA with Dunnett’s multiple comparison test. Sample size and *P* value: *n* = 24, *P* = 7.92 × 10^−12^ (*en* > *RFP*), *n* = 27 (*en>strip* RNAi), *n* = 27, *P* = 9.94 × 10^−1^ (*en>strip* RNAi, *wg* RNAi), *n* = 19, *P* = 1.18 × 10^−1^ (*en>strip* RNAi, *sat1/2* mutant line1), *n* = 10, *P* = 1.62 × 10^−6^ (*en>strip* RNAi, *wg* RNAi, *sat1/2* mutant line1). (**G**) Quantification of the RFP-positive area (expressed as % of total disc area) in wing discs bearing wild-type or *strip*-knocked down cells, with or without *wg* knockdown, alone, or in combination with *sat1/2* background mutations. ns, not significant; ^∗∗∗∗^*P* < 0.0001; one-way ANOVA with Dunnett’s multiple comparison test. Sample size and *P* value: *n* = 24, *P* = 1.30 × 10^−7^ (*en* > *RFP*), *n* = 27 (*en>strip* RNAi), *n* = 27, *P* = 8.76 × 10^−1^ (*en>strip* RNAi, *wg* RNAi), *n* = 19, *P* = 3.28 × 10^−5^ (*en>strip* RNAi, *sat1/2* mutant line1), *n* = 10, *P* = 2.77 × 10^−6^ (*en>strip* RNAi, *wg* RNAi, *sat1/2* mutant line1). (**H**–**M**) Confocal images showing wing discs bearing wild-type or *strip-*knocked down cells with or without *spitz* knockdown alone, or *spitz* knockdown with *sat1/2* background mutations marked with RFP expression (magenta) and stained with anti-phospho-S6 (green). Male animals of these strains were selected for the experiment, and the *sat1/2* background mutations were hemizygous. RFP (magenta), outlined by white dashed lines, marks the expression pattern of *en-Gal4* in the wing disc. (**N**) Quantification of the size of the phospho-S6 positive region (% of phospho-S6 positive area/disc area) in wing discs bearing wild-type, *strip-*knockdown cells with or without *spitz* knockdown alone, or *spitz* knockdown with *sat1/2* background mutations. ns, not significant; ^∗∗∗∗^*P* < 0.0001; one-way ANOVA with Dunnett’s multiple comparison test. Sample size and *P* value: *n* = 23, *P* = 9.94 × 10^−12^ (*en* > *RFP*), *n* = 21 (*en>strip* RNAi), *n* = 25, *P* = 9.76 × 10^−1^ (*en>strip* RNAi, s*pitz* RNAi), *n* = 16, *P* = 9.71 × 10^−3^ (*en>strip* RNAi, *sat1/2* mutant line1), *n* = 12, *P* = 2.94 × 10^−1^ (*en>strip* RNAi, *sat1/2* mutant line2), *n* = 22, *P* = 1.39 × 10^−10^ (*en>strip* RNAi, *spitz* RNAi, *sat1/2* mutant line1), *n* = 25, *P* = 1.46 × 10^−9^ (*en>strip* RNAi, *spitz* RNAi, *sat1/2* mutant line2). (**O**) Quantification of the RFP-positive area (expressed as % of the total disc area) in wing discs bearing wild-type or *strip*-knocked down cells, with or without *spitz* knockdown, alone, or in combination with *sat1/2* background mutations. ns, not significant; ^∗∗∗^*P* < 0.001; ^∗∗∗∗^*P* < 0.0001; one-way ANOVA with Dunnett’s multiple comparison test. Sample size and *P* value: *n* = 23, *P* = 1.35 × 10^−7^ (*en* > *RFP*), *n* = 21 (*en>strip* RNAi), *n* = 25, *P* = 9.30 × 10^−1^ (*en>stri*p RNAi, *spitz* RNAi), *n* = 16, *P* = 5.05 × 10^−4^ (*en>strip* RNAi, *sat1/2* mutant line1), *n* = 12, *P* = 1.00 (*en>strip* RNAi, *sat1/2* mutant line2), *n* = 22, *P* = 7.24 × 10^−9^ (*en>strip* RNAi, *spitz* RNAi, *sat1/2* mutant line1), *n* = 25, *P* = 5.63 × 10^−8^ (*en>strip* RNAi, *spitz* RNAi, *sat1/2* mutant line2). Scale bars represent 50  μm in (**A**–**E**, **H**–**M**). Dots represent biological replicates (**F**, **G**, **N**, **O**); error bars indicate SEM. Source data are available online for this figure.

Because *wg* or *spitz* knockdown alone demonstrated only modest effects (Figs. 4K and 8A–C,H, and I, quantified in F and N), we speculated that other factors, such as Sat1/2, may function redundantly. To test this, we knocked down *wg* or *spitz* in a *sat1/2* double mutant background (Fig. EV5I). Although neither *sat1/2* mutations nor *wg* or *spitz* knockdown alone significantly suppressed non-cell-autonomous mTOR activation (Fig. 8A–D,H–K, quantified in F and N), the combined perturbation substantially reduced mTOR signaling (Fig. 8E,L,M, quantified in F and N). These results indicate that Wg/Spitz and Sat1/2 function redundantly in supporting mTOR activation, and that the simultaneous inhibition of all three components exerts a synergistic suppressive effect.

Consistently, the cell-autonomous growth of Hippo-activated cells was modestly suppressed by *sat1/2* mutations, but not by *wg* or *spitz* knockdown alone (Fig. 8A–D,H–K, quantified in G and O). In contrast, the triple perturbation significantly reduced cell-autonomous growth (Fig. 8E,L,M, quantified in G and O), paralleling the effects observed for mTOR suppression (Fig. 8F,G,N,O) and mirroring those observed with *sat1/2* knockdown alone (Fig. 7G,H). Because Wg promotes the survival and tumorigenesis of Wg-secreting cells (Waghmare et al, 2024), Wg/Spitz cooperates with Sat1/2 to shape a tumor-promoting microenvironment that enhances both cell-autonomous and non-cell-autonomous tumor growth.

## Discussion

**Figure 9:**
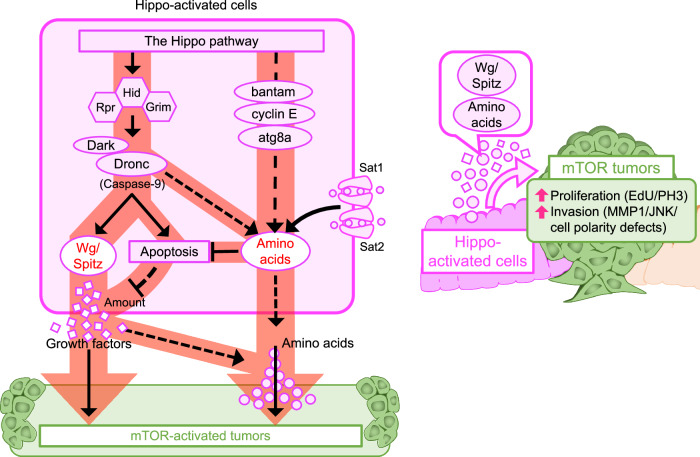
Hippo-activated cells induce non-cell-autonomous tumorigenesis through Wg, Spitz, and Sat1/2. Schematic depiction of the mechanistic hypothesis underlying non-cell-autonomous tumorigenesis caused by Hippo-activated cells. Hippo activation alters the expression of several genes, including *rpr*, *hid*, *bantam*, *atg8a*, and *cyclin E*. Hippo activates Dronc, which subsequently enhances Wg/Spitz expression and secretion. Furthermore, Sat1/2 were identified as a collaborator of Wg/Spitz. Sat1/2 can promote the survival of cells secreting Wg/Spitz through amino acid import, thereby increasing Wg/Spitz secretion. Alternatively, Sat1/2 can facilitate amino acid import, which is utilized to elevate amino acid levels in the microenvironment. Those signal molecules, including Wg/Spitz and amino acids, in the microenvironment would cause non-cell-autonomous mTOR activation and subsequent tumorigenesis.

Several studies have asserted that the Hippo pathway functions as a tumor suppressor in *Drosophila* and mammals, because mutations in the Hippo pathway promote tumorigenesis (Moroishi et al, 2015; Yu et al, 2015). However, recent studies have suggested that the Hippo pathway functions as a tumor promoter in certain contexts; it promotes tumor growth and invasive behavior in a cell-autonomous manner (Moroishi et al, 2016; Cottini et al, 2014; Yuan et al, 2008; Pearson et al, 2021). Here, we provided genetic evidence that Hippo-activated cells act as oncogenic niche cells that promote tumorigenesis in a non-cell-autonomous manner (Fig. 9). Our study demonstrated that Hippo pathway activation triggers Dronc–Wg/Spitz signaling, specifically in the hinge and ventral notum regions, leading to mTOR activation, in neighboring non-autonomous cells. However, Dronc–Wg/Spitz signaling alone is insufficient to fully account for the tumorigenic effects of Hippo activation. To identify additional contributors, we performed a genetic screen for *strip/hippo*-interacting factors and identified two previously uncharacterized amino acid transporters, Sat1 and Sat2. These transporters cooperate with Wg/Spitz to promote non-cell autonomous mTOR activation in the context of Hippo pathway activation.

Previous studies have found that Src-activated cells act as oncogenic niche cells that induce non-cell-autonomous tumorigenesis through activation of the JNK pathway (Enomoto and Igaki, 2013; Enomoto et al, 2015). Similarly, we found that Rho1, a JNK pathway regulator, was required for non-cell-autonomous mTOR activation in Hippo-activated cells (Fig. 4E, quantified in F). This suggests that Hippo- and Src-driven oncogenic niches may share a common underlying mechanism. Moreover, Src activation in cooperation with *Ras*^*V12*^ expression promotes Wg expression via the JNK pathway (Hirabayashi et al, 2013). Consistently, our findings demonstrated that Hippo-activated cells upregulated Wg expression to drive non-cell-autonomous mTOR activation (Fig. 4M, quantified in N–P), further supporting the possibility of a convergent mechanism involving Wg expression in both contexts. However, Src activation inhibits the Hippo pathway (Enomoto and Igaki, 2013), meaning that Src-activated cells are not classified as Hippo-activated cells. This indicates that these two oncogenic niche types differ in the upstream driving pathway. Altogether, these findings suggest that Hippo- and Src-activated cells utilize distinct signaling cascades, but converge on a common downstream outcome, Wg expression, to promote non-cell-autonomous tumorigenesis.

Hippo-activated cells undergo activation of caspases and Wg upregulation and subsequently stimulate cell proliferation in adjacent cells (Fig. 9). These mechanisms of tumorigenesis resemble those involved in the regeneration/morphogenesis systems in hydra, where Wg/WNT is upregulated in apoptotic cells for regeneration (Chera et al, 2009; Brooun et al, 2022). Previous research has suggested that Src activation, which induces non-cell-autonomous tumorigenesis, utilizes a pathway analogous to regeneration (Enomoto and Igaki, 2013), non-cell-autonomous tumorigenesis may be linked to regenerative processes. Moreover, certain components involved in Hippo activation-mediated tumorigenesis are shared with AiP processes in imaginal discs (Fan et al, 2014; Smith-Bolton, 2016). Specifically, Wg/Spitz are secreted by caspase-activated cells, and JNK activation is induced in neighboring cells during AiP processes (Smith-Bolton, 2016; Fan et al, 2014). Because Hippo activation employs Wg/Spitz to induce mTOR-activated tumors (Fig. 4I,J, quantified in K), where the JNK pathway is activated (Fig. EV2D,E, quantified in F), it is plausible that the AiP pathway contributes to tumorigenesis associated with Hippo activation.

Our findings revealed that the spatial context in which Hippo signaling is activated critically influences tumor induction. Specifically, Hippo activation in the hinge and ventral notum, but not in the pouch region, was sufficient to induce mTOR-activated tumors (Fig. 2). This regional difference can be explained by the observation that ectopic Wg expression is induced by Hippo activation in the hinge/ventral notum but not in the pouch (Fig. 4L,M, quantified in N–P; Fig. EV4N,O, quantified in Fig. 5F). Although Hippo activation triggered Dronc activation in both regions, as shown by cleaved Drice/Dcp1 staining (Fig. EV4H,I, quantified in Fig. 5D,E), Wg expression was absent in the pouch. Dronc activity was more robust in the hinge and the ventral notum (Fig. EV4J), suggesting that a threshold level of caspase activity is required to induce Wg expression. Interestingly, our observations contrast with previous findings in eye discs, where Hippo signaling was demonstrated to suppress Wg expression (Wittkorn et al, 2015). This discrepancy implies that the effect of Hippo signaling on Wg expression may be tissue- or compartment-specific.

The suppressive effect of *wg*/*spitz* RNAi on mTOR activity was observed with *ptc-Gal4* driver but not with *en-Gal4* (Figs. 4K, and  8F,N). The mechanism underlying this regional difference is currently unresolved. One possible explanation is that Hippo-activated cells produce distinct combinations or levels of growth factors in different regions of the wing disc, resulting in region-specific sensitivity to the depletion of individual ligands.

Although Dronc–Wg/Spitz signaling was necessary for non-cell-autonomous mTOR activation, it is not sufficient to fully recapitulate Hippo-induced mTOR activation. This led us to hypothesize the involvement of additional factors downstream of Hippo activation. We found that Hippo-activated cells exhibited altered expression of *bantam*, *cyclin E*, and *atg8a*, which contribute to non-cell-autonomous mTOR activation. Furthermore, genetic screening identified Sat1 and Sat2—putative amino acid transporters—as potential modulators of non-cell-autonomous mTOR activation. Because *bantam* and *atg8a* regulate autophagy, which produces amino acids via protein degradation, amino acids may be a common key factor. However, the precise mechanism through which Sat1/2 and Yki target genes, including *bantam* and *atg8a*, regulate non-cell-autonomous tumorigenesis remains unclear. One possibility is that Sat1/2 and Yki target genes, including *bantam* and *atg8a*, regulate autophagy for amino acid secretion. Recent studies have demonstrated that autophagy in neighboring benign cells induces amino acid secretion, which supports tumor growth (Katheder et al, 2017; Sousa et al, 2016). Because autophagy is controlled by *bantam* and *atg8a* but also certain amino acid transporters (Texada et al, 2019; Xu et al, 2024; Matsui and Fukuda, 2013; Villagomez et al, 2024), Sat1/2 may cooperate with *bantam* and Atg8a to promote amino acid secretion by modulating autophagy-dependent protein degradation. Moreover, the alternative possibility is that Sat1/2 may promote the survival of Hippo-activated cells, maintaining secretion of growth factors. Because *sat1/2* double mutants enhanced Drice/Dcp-1 activation in Hippo-activated cells (Fig. EV5J–M, quantified in N), Sat1/2 enhanced cell survival and maintained the population of Hippo-activated cells. This may potentially promote the secretion of tumor-promoting factors, such as Wg/Spitz, which indirectly promotes non-cell-autonomous amino acid incorporation/mTOR activation (Fig. 9).

Our study collectively uncovered a previously unrecognized role for the Hippo pathway in promoting tumor development. This finding not only enhances our understanding of the function of the Hippo pathway but also has potential implications for the development of therapeutic strategies targeting the Hippo pathway in human cancers.

## Methods

**Table:** Reagents and tools table

Reagent/resource	Reference or source	Identifier or catalog number
**Experimental models**		
*Drosophila melanogaster*	Bloomington Drosophila Stock Center (BDSC), Vienna Drosophila Resource Center (VDRC)	N/A
*ptc-Gal4*	BDSC	#2017
*en-Gal4, UAS-RFP*	BDSC	#30557
*ci-Gal4, UAS-GFP*	Shizue Ohsawa	
*pnr-Gal4*	BDSC	#3039
*nub-Gal4*	BDSC	#86108
*41D11-Gal4*	Gines Morata	
*bx-Gal4*	BDSC	#8696
*284-Gal4*	Gines Morata	
*OK6-Gal4*	Corey S Goodman	
*hs-FLP; Ay-Gal4*	Tatsushi Igaki	
*UAS-strip RNAi* ^*#6+9*^	Takahiro Chihara	
*UAS-strip RNAi* ^*#9-1-5*^	Takahiro Chihara	
*UAS-wts*	Georg Halder	
*UAS-yki RNAi*	BDSC	#34067
*UAS-FLP, Ubi-FRT-STOP-FRT-nEGFP*	BDSC	#28282
*UAS-hippo RNAi*	BDSC	#33614
*UAS-bantam*	BDSC	#60671
*UAS-cyclin E*	BDSC	#4781
*UAS-atg8a RNAi*	BDSC	#34340
*UAS-miRHG*	Chun-Hong Chen	
*UAS-myc*	BDSC	#64759
*UAS-brk*	BDSC	#93081
*UAS-chinmo RNAi*	BDSC	#26777
*UAS-fng RNAi*	BDSC	#25947
*UAS-diap1*	BDSC	#63820
*UAS-InR*	BDSC	#8248
*UAS-InR*	BDSC	#8250
*UAS-dark RNAi*	BDSC	#33924
*UAS-dronc RNAi*	BDSC	#32963
*UAS-rho1 RNAi*	BDSC	#27727
*UAS-p35*	BDSC	#5073
*UAS-p35*	Bruce Hay	
*UAS-hid*	BDSC	#65403
*UAS-hid*	Tatsushi Igaki	
*UAS-bantam sponge*	Tatsushi Igaki	
*puc-stinger*	Tatsushi Igaki	
*UAS-rpr*	BDSC	#5824
*UAS-spitz RNAi*	BDSC	#28387
*UAS-wg RNAi*	BDSC	#31249
*UAS-CG4991 RNAi*	VDRC	#30264
*UAS-CG16700 RNAi*	BDSC	#61217
*sgRNA for CG16700*	BDSC	#82722
*nos-Cas9*	NIG-FLY	#CAS-0001
*tubP-Gal80*	BDSC	#9491
*UAS-mCD8-RFP*	BDSC	#32218
*UAS-mCD8-GFP*	Liqun Luo	
*Drosophila* deficiency lines	Bloomington *Drosophila* Stock Center and Kyoto *Drosophila* Stock Center	
**Recombinant DNA**		
CG4991 sequence	DGRC	# LD23664
CG4991 sequence	DGRC	#UFO02039
**Antibodies**		
Rabbit anti-cleaved *Drosophila* Drice/Dcp-1 (Asp216)	Cell Signaling Technology	#9578
Rabbit anti-phospho-S6	Jongkyeong Chung and Aurelio Teleman	
Mouse anti-MMP1	DSHB	#5H7B11 #3A6B4 #3B8D12
Mouse anti-DLG	DSHB	#4F3
Rat anti-phospho-histone H3	Abcam	#ab10543
Rat anti-E-Cad	DSHB	#DCAD2
Mouse anti-Wg	DSHB	#4D4
Guinea pig anti-Expanded	Rick Fehon	
Mouse anti-Flag	Sigma-Aldrich	#F1804-200UG
Goat anti-rabbit Alexa Fluor™ 647	Invitrogen	#A-21245
Goat anti-rabbit Alexa 568	Invitrogen	#A-11036
Goat anti-rabbit Alexa 488	Invitrogen	#A-11034
Goat anti-mouse Alexa 488	Invitrogen	#R37120
Goat anti-rat Alexa 488	Invitrogen	#A-11006
Goat anti-guinea pig Alexa 488	Invitrogen	#A-11073
**Oligonucleotides and other sequence-based reagents**		
Oligonucleotides for guide RNAs (*sat1* knockout)	F-5′-CTTCGTACTTCATCGCTACTTCTC-3’ R-5′- AAACGAGAAGTAGCGATGAAGTAC-3’	This study
Oligonucleotides for guide RNAs (*sat1* knockout)	F-5’- CTTCGCCGTGCTGGGCATCGTCAC-3′ R-5′-AAACGTGACGATGCCCAGCACGGC-3'	This study
Cloning primers (*sat1* 5’ homology arm)	F-5′-CCCTTCGCTGAAGCAGGTGGctcacagcgagcaagaggtgatcatcg-3′ R-5′-GCAGGTGTGCATATGTCCGCaagtagcgatgaagtacagttaggtctc-3′	This study
Cloning primers (*sat1* 3’ homology arm)	F-5′-TATAGAAGAGCACTAGTAAAcactggcacctaccagagcatcgtgg-3′ R-5′-ACTCGATTGACGGAAGAGCCtactttttaaaaaacccctattaccc-3′	This study
*sat1* cloning primers (*UAS-sat1*)	F-5’-AGGGAATTGGGAATTATGGGAAGAACACTGGAAATAACCG-3’ R-5’-ATCTGTTAACGAATTCCTACTTAAACTCCTTGACGATCTCCACG-3’	This study
**Chemicals, enzymes and other reagents**		
VECTASHIELD Mounting Medium	Funakoshi Corporation	#H-1000
DAPI	Nacalai Tesque	#11034-56
TRITC-phalloidin	Sigma-Aldrich	#P1951
Click-iT™ EdU Cell Proliferation Kit for Imaging, Alexa Fluor™ 647 dye	Invitrogen	#C10340
Click-IT™ HPG Alexa Fluor™ 488 Protein Synthesis Assay Kit	Invitrogen	#C10428
BbsI-HF	Biolabs	# R3539L
FastDigest EcoRⅠ	Thermo Fisher	#FD0274
FastDigest NotI	Thermo Fisher	#FD0594
FastDigest BglII	Thermo Fisher	#FD0083
FastDigest XhoⅠ	Thermo Fisher	#FD0694
**Software**		
GraphPad Prism 9 or 10	http://www.graphpad.com	
ImageJ	https://imagej.net/ij/	
Fiji	https://imagej.net/software/fiji/	
**Cell materials**		
S2 cell (S2-DRSC)	Drosophila Genomics Resource Center	#181
**Other**		

### Fly strains

The flies were raised in standard fly food at 25 °C. At 48–72 h after laying eggs, heat shock was applied to *hs-FLP*; *Ay-Gal4* for 30 min at 37 °C. After egg laying for 1 day, heat shock was applied to inactivate Gal80^ts^ by shifting the temperature to 31 °C at different developmental stages and maintained until the wandering third-instar larval stage. Unless otherwise stated, the sexes of the dissected larvae was not determined. The following flies were used in this study: *ptc-Gal4* (BL2017), *en-Gal4*, *UAS-RFP* (BL30557), *ci-Gal4*, *UAS-GFP* (from Shizue Ohsawa), *pnr-Gal4* (BL3039), *nub-Gal4* (BL86108), *41D11-Gal4* (Medina et al, 2021), *bx-Gal4* (BL8696), *284-Gal4* (Medina et al, 2021), *OK6-Gal4* (Aberle et al, 2002), *hs-FLP*; *Ay-Gal4* (from Tatsushi Igaki), *UAS-strip* RNAi^#6+9^ (Sakuma et al, 2016), *UAS-strip* RNAi^#9-1-5^ (Sakuma et al, 2016), *UAS-wts* (Sansores-Garcia et al, 2011), *UAS-yki* RNAi (BL34067), *UAS-FLP, Ubi-FRT-STOP-FRT-nEGFP* (BL28282), *UAS-hippo* RNAi (BL33614), *UAS-bantam* (BL60671), *UAS-cyclin E* (BL4781), *UAS-atg8a* RNAi (BL34340), *UAS-miRHG* (From Chun-Hong Chen), *UAS-myc* (BL64759), *UAS-brk* (BL93081), *UAS-chinmo* RNAi (BL26777), *UAS-fng* RNAi (BL25947), *UAS-diap1* (BL63820), *UAS-InR*^#1^ (BL8248), *UAS-InR*^#2^ (BL8250), *UAS-dark* RNAi (BL33924), *UAS-dronc* RNAi (BL32963), *UAS-rho1* RNAi (BL27727), *UAS-p35* (BL5073), *UAS-p35* (from Bruce Hay), *UAS-hid* (BL65403 or from Tatsushi Igaki), UAS-*bantam sponge*, *puc-stinger* (from Tatsushi Igaki), *UAS-rpr* (BL5824), *UAS-spitz* RNAi (BL28387), *UAS-wg* RNAi (BL31249), *UAS-CG4991* RNAi (VDRC30264), *UAS-CG16700* RNAi (BL61217), *sgRNA* for *CG16700* (*sat2)* (BL82722), *nos-Cas9* (CAS-0001), *w; Sco/CyO, tubP-Gal80* (BL9491), *UAS-mCD8-RFP* (BL32218), *UAS-mCD8-GFP* (from Liqun Luo), and *Drosophila* deficiency lines (Bloomington *Drosophila* Stock Center and Kyoto *Drosophila* Stock Center). All the genotypes are listed in Appendix Table S1.

The *sat1/2* mutant was generated in this study. As shown in Fig. EV5I, the *sat1* gene region was replaced by *3 × P3 DsRed* to generate a *sat1* null mutant using CRISPR-Cas9-triggered homologous recombination. Two guide RNA vectors and one donor vector were injected into the *vasa-Cas9* fly line (GenetiVision). The DNA fragments of the guide RNAs were subcloned into a *Bbs*I-digested U6b-*sgRNA* vector. Oligonucleotide sequences for the guide RNAs are listed in the Reagents and Tools Table. The 5’ homology arm (1 kbp-sequences upstream from 78 bp after the start codon) was amplified by PCR using the primers listed in the Reagents and Tools Table. The 3′ homology arm (1 kbp-sequences downstream from 46 bp before the stop codon) was amplified by PCR using the primers listed in the Reagents and Tools Table. The 5’ homology arm was subcloned into the cut site of the *pHD-DsRed-attP* vector using *EcoR*Ⅰ and *Not*I. The 3’ homology arm was subcloned into the cut site of the *pHD-DsRed-attP* vector using *Bgl*II and *Xho*Ⅰ. We screened for DsRed-positive transformants, and the *sat1* depletion was confirmed by genomic sequencing. Next, we crossed the *sat2 sgRNA* line (BL82722) with flies expressing *Cas9* in germ cells (CAS-0001) to induce *sat2* mutations in the *sat1* mutant. A heteroduplex mobility assay was conducted in the F1 generation using microchip electrophoresis on a MultiNA (Shimazu, MCE-202). Finally, we confirmed the *sat2* mutations by genomic sequencing.

*UAS-sat1* was generated in the present study, *sat1* sequence (DGRC# LD23664) was amplified by PCR using the primers listed in the Reagents and Tools Table. The *sat1* sequence was subcloned into the *EcoR*I-digested *pUAST*. The vector was integrated into the fly genome using the *piggyBac* system. The *UAS-sat1 *strain was generated by Toshiyuki Fujii (Hiroshima University, Japan).

### Immunostaining

Wandering third-instar larvae were dissected in phosphate-buffered saline (PBS) containing 0.3% Triton X-100 (0.3% PBST), fixed with 4% paraformaldehyde (PFA) for 20 min at room temperature, and washed three times with 0.3% PBST for 20 min. The samples were blocked with 5% normal goat serum (NGS) in 0.3% PBST for 1 h. The blocked samples were incubated at 4 °C overnight with primary antibodies in 0.3% PBST, washed three times with 0.3% PBST for 20 min, and incubated at 4 °C overnight with secondary antibodies in 0.3% PBST. After washing three times with 0.3% PBST, the samples were incubated at 4 °C overnight or at room temperature for 1 h with a VECTASHIELD Mounting Medium (Funakoshi Corporation #H-1000) containing DAPI (1:100, Nacalai Tesque #11034-56, 0.15 μg/ml), and were mounted with the same mounting medium. The primary antibodies used were rabbit anti-cleaved *Drosophila* Drice/Dcp-1 (Asp216) (1:100 or 1:400, Cell Signaling Technology #9578), rabbit anti-phospho-S6 (1:500 or 1:1000, gifts from Jongkyeong Chung and Aurelio Teleman), mouse anti-MMP1 (1:1:1 mixture of 5H7B11, 3A6B4, and 3B8D12 were diluted 1:10, DSHB), mouse anti-DLG (1:200, DSHB #4F3), rat anti-phospho-histone H3 (1:500, Abcam #ab10543), rat anti-E-Cad (1:100, DSHB #DCAD2), mouse anti-Wg (1:100 DSHB #4D4), guinea pig anti-Expanded (1:5000, gifts from Rick Fehon), and mouse anti-Flag (1:200, Sigma-Aldrich #F1804-200UG). The secondary antibodies used were goat anti-rabbit Alexa Fluor™ 647 (1:250 or 1:1000, Invitrogen #A-21245), goat anti-rabbit Alexa 568 (1:250, Invitrogen #A-11036), goat anti-rabbit Alexa 488 (1:250, Invitrogen #A-11034), goat anti-mouse Alexa 488 (1:250, Invitrogen #R37120), goat anti-rat Alexa 488 (1:250, Invitrogen #A-11006), and goat anti-guinea pig Alexa 488 (1:1000, Invitrogen #A-11073). Actin was stained using TRITC-phalloidin (1:200, Sigma-Aldrich #P1951). The images were captured using a Zeiss LSM900 confocal microscope.

### EdU staining

We followed the protocol for Click-iT™ EdU Cell Proliferation Kit for Imaging, Alexa Fluor™ 647 dye (Invitrogen #C10340). The wing discs were incubated in 1× PBS supplemented with EdU (1:1000) for 20 min at room temperature and washed once with 1× PBS. The discs were fixed with 4% PFA for 30 min, washed three times with 0.3% PBST for 20 min, and blocked with 5% NGS in 0.3% PBST for 1 h at room temperature. To visualize EdU incorporation, the discs were incubated in the Click-iT^®^ reaction cocktail for 30 min and washed three times with 0.3% PBST for 20 min at room temperature. After EdU staining, the discs were stained with antibodies following the protocol described above.

### HPG incorporation assay

We followed the protocol for Click-IT™ HPG Alexa Fluor™ 488 Protein Synthesis Assay Kit (Invitrogen # C10428). The wing discs were dissected, washed once with 1× PBS, and supplemented with HPG (1:1000) for 20 min at room temperature. After washing once with 1× PBS, the discs were fixed in 4% PFA for 20 min. Next, the discs were washed twice with 1× PBS and incubated with 0.3% PBST for 20 min. After washing twice with 1× PBS, the discs were incubated in the Click-iT reaction cocktail for 30 min, washed once with reaction rinse buffer, and four times with 1× PBS. The discs were incubated at room temperature for 1 h with the VECTASHIELD Mounting Medium containing DAPI and mounted with the same mounting medium.

### Dietary manipulations

Standard fly food contained the following ingredients: 50 mL water, 0.35 g agar, 2 g yeast, 2.5 g cornflower, 5 g glucose, 1.5 g rice bran, 200 μL propionic acid, and 200 μL Bokinin. A 50% or 200% yeast diet was prepared by decreasing or increasing the yeast concentration in the standard fly food. A synthetic diet, HolFast (Sorge et al, 2025), was used to control specific amino acid levels in the diet. As complete depletion of essential amino acids, such as methionine, disturbs normal development, we decreased methionine to half of the control diet (50% Met).

### Measurement of amino acids

Ten wing discs were homogenized in 115 µL of ultra-pure water (UPW) and flash-frozen in liquid nitrogen. Next, 100% methanol containing 20 µM internal standards (methionine sulfone and 2-morpholinoethanesulfonic acid) was added, following deproteinization with chloroform and acetonitrile (Kosakamoto et al, 2024). The samples were placed into a pre-washed 10 kDa centrifugal device (Pall #OD010C35). After centrifugation, the samples were evaporated using a centrifugal concentrator (TAITEC #VC-96R), resolubilized with ultrapure water, and subjected to LC-MS/MS (LC-MS-8060NX, Shimadzu) with a PFPP column (Discovery HS F5 (2.1 mm×150 mm, 3 μm; Sigma-Aldrich) in the column oven at 40 °C. A gradient of solvent A (0.1% formic acid in water) to solvent B (0.1% formic acid, acetonitrile) was applied for 20 min to separate the solutes. The MRM parameters were optimized using the LabSolutions LC-MS software (Shimadzu) (Kosakamoto et al, 2024). The metabolite concentrations were normalized to methionine sulfone.

### Processing, calculation, and statistical analyses for image quantification

The images were processed in the ImageJ/Fiji software, applying a threshold to eliminate the background/low signal. We defined the region of the wing disc, RFP^+^, GFP^+^ or pS6 cell clusters as regions of interest (ROIs) using particle analysis in the ImageJ/Fiji software. We measured the size of the entire wing disc, RFP^+^ region, RFP^−^ region, or pS6 cell cluster. Furthermore, we measured (i) the positive areas of anti-phospho-S6, anti-MMP1, anti-Drice/Dcp1, anti-Expanded, anti-Wg staining; (ii) the mean intensity of EdU, anti-DLG, anti-E-Cad, HPG; or (iii) the number of PH3-positive dots in specific regions (whole wing disc, RFP^+^, RFP^−^, GFP^+^ region, or pS6 cell clusters). The measured values (i–iii) were normalized to the corresponding area of each region (whole wing disc, RFP^+^, RFP^−^, GFP^+^ region, or pS6 cell clusters). All statistical analyses were performed using GraphPad Prism version 9 or 10. Quantitative data were statistically analyzed using Welch’s *t* test or unpaired *t* test for single comparison, or one-way analysis of variance (ANOVA) with Dunnett’s multiple comparison test for multiple comparisons. All data shown within the same graph were obtained from experiments conducted simultaneously. Each condition was analyzed using at least three biological or technical replicates. The error bars represent the standard error of the mean (SEM).

### Genetic modifier screen to identify the *strip*-interacting genes

*strip* knockdown in motor neurons using *OK6-Gal4* resulted in pupal lethality. We screened for genes whose deletion could rescue the pupal lethality caused by *strip* knockdown. *UAS-strip RNAi; OK6-Gal4/ tubP-Gal80, CyO* was crossed with the genomic deletion strains. *Strip* knockdown was performed with the heterozygous or hemizygous genomic deletion background in the F1 generation. We marked the pupae of the F1 generation and counted the number of adults enclosed within 120 h after pupation. Individuals that did not emerge within 120 h of pupation were defined as dead pupae. Deletion strains with a survival rate > 10% were identified as strains that could rescue pupal lethality caused by *strip* knockdown. Nutrient-rich foods were used to facilitate larval growth. Further details on food conditions will be sent upon request. The initial screening was performed by Tomoki Umehara (University of Tokyo).

### Establishment of S2 cells bearing *pMT-Flag-sat1-puro* and analysis of Sat1 localization

The *Flag* sequence was attached to the *sat1* sequence (DGRC#UFO02039), which was subcloned into the *pMT-puro* plasmid (#17923; Addgene). *pMT-Flag-sat1-puro* plasmid or empty plasmid (*pMT-puro*) as a control was transfected into S2 cells and inserted into the genome of S2 cells. Transfected S2 cells were selected using puromycin, and S2 cells bearing *pMT-Flag-sat1-puro* or *pMT-puro* were established. S2 cells (2.0 × 10^6^ cells/well) were seeded on a 6-well plate and incubated with 0.5 mM CuSO_4_(II) for 24 h to activate the copper-inducible promoter (*metallothionein* gene promoter) and induce Flag-Sat1 overexpression. Sat1 localization was detected by staining with a FLAG-tag.

## Supplementary information

Appendix

Peer Review File

Source data Fig. 1

Source data Fig. 2

Source data Fig. 3

Source data Fig. 4

Source data Fig. 5

Source data Fig. 6

Source data Fig. 7

Source data Fig. 8

Expanded View Figures

## Data Availability

This study includes no data deposited in external repositories. The source data of this paper are collected in the following database record: biostudies:S-SCDT-10_1038-S44319-026-00778-5.
