# Supplementary material for: The Hippo tumor suppressor pathway triggers non-cell autonomous tumorigenesis in Drosophila
Source: EMBO Rep. 2026 May 1;27(11):2915–46. doi: 10.1038/s44319-026-00778-5 (PMC13261145; doi:10.1038/s44319-026-00778-5)
Supplement: Supplementary file 1 — Appendix [file 44319_2026_778_MOESM1_ESM.pdf]

Appendix

Appendix Figure S1 2
Appendix Figure S2 3
Appendix Figure S3 4
Appendix Figure S4 5
Appendix Table S1 6–8

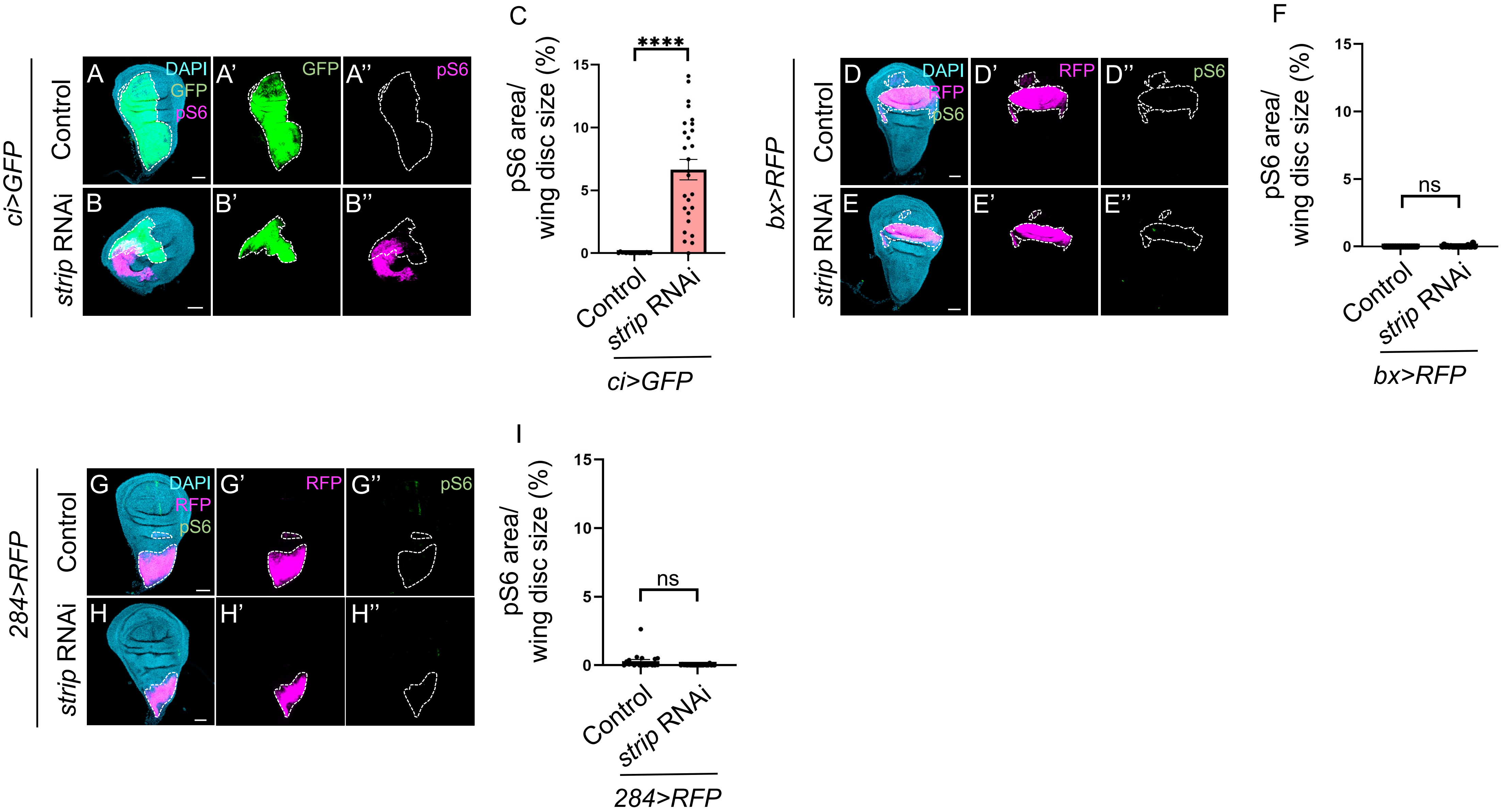

Appendix Figure S1. Hippo activation in the pouch/dorsal notum region cannot induce mTOR activation

(A and B) Confocal images show the wing discs bearing wild-type or *strip*-knocked down cells marked with GFP expression (green) and stained with anti-phospho-S6 (magenta). GFP (green), outlined by white dashed lines, marks the expression pattern of *ci-Gal4* in the wing disc. (C) Quantification of the size of the phospho-S6 positive region (% of phospho-S6 positive area/disc area) in the wild-type or *strip*-knocked down wing disc. ****$p < 0.0001$; Welch's t-test. Sample size and p value: n=18 (*ci>GFP*), n=27, $p=1.13\times10^{-8}$ (*ci>strip* RNAi).

(D and E) Confocal images show the wing discs bearing wild-type or *strip*-knockdown cells marked with RFP expression (magenta) and stained with anti-phospho-S6 (green). RFP (magenta) outlined by white dashed lines marks the expression pattern of *bx-Gal4* in the wing disc. (F) Quantification of the size of the phospho-S6 positive region (% of phospho-S6 positive area/disc area) in the wild-type or *strip*-knocked down wing disc. ns, not significant; Welch's t-test. Sample size and p value: n=18 (*bx>RFP*), n=20, $p=5.39\times10^{-2}$ (*bx>strip* RNAi).

(G and H) Confocal images show the wing discs bearing wild-type or *strip*-knockdown cells marked with RFP expression (magenta) and stained with anti-phospho-S6 (green). RFP (magenta) outlined by white dashed lines marks the expression pattern of *284-Gal4* in the wing disc. (I) Quantification of the size of the phospho-S6 positive region (% of phospho-S6 positive area/disc area) in the wild-type or *strip*-knocked down wing disc. ns, not significant; Welch's t-test. Sample size and p value: n=20 (*284>RFP)*, n=22, $p=5.17\times10^{-2}$ (*284>strip* RNAi). Scale bars represent 50 μm in (A, B, D, E, G, H). Dots represent biological replicates (C, F, I); error bars indicate SEM.

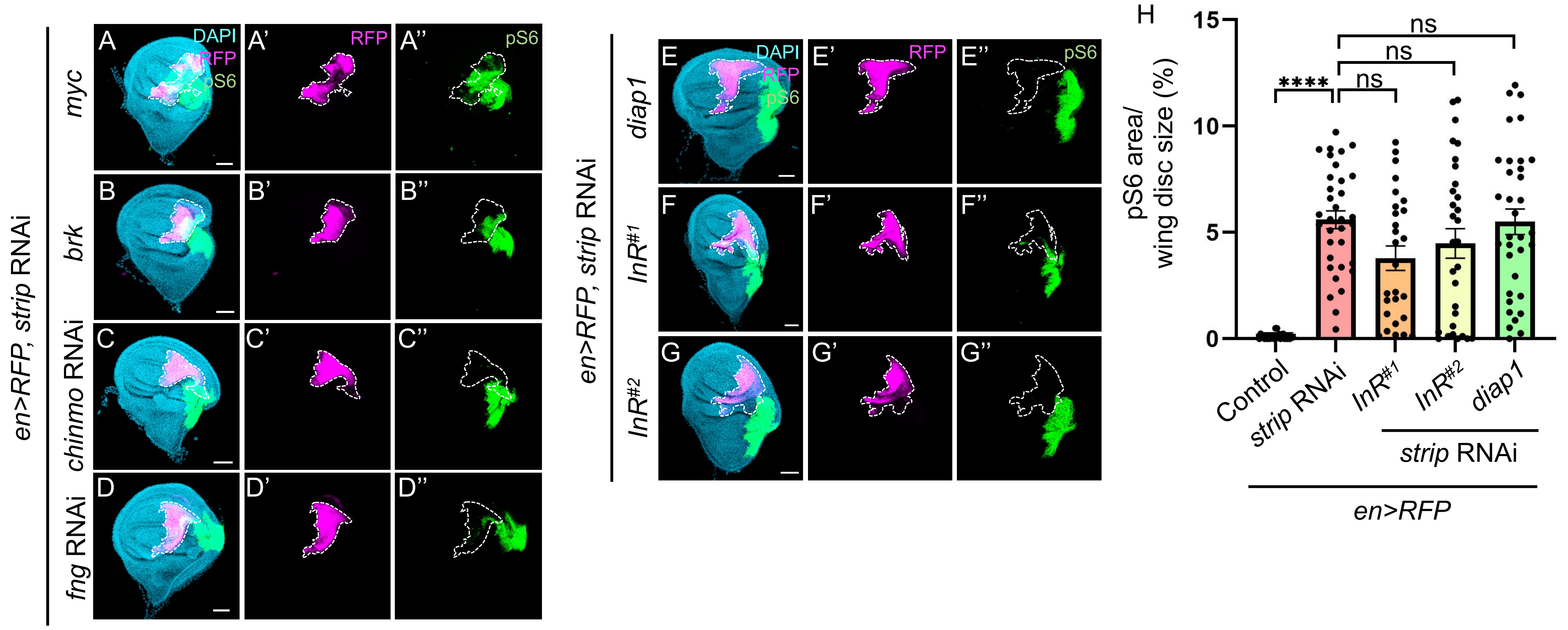

Appendix Figure S2. Hippo activation, rather than by controlling the expression levels of *myc*, *brk*, *chinmo*, *fng*, *diap1*, and *InR,* induces the mTOR activation

(A–G) Confocal images show the wing discs bearing wild-type or *strip*-knockdown cells with or without overexpression of *myc*, *brk*, *diap1*, *InR*, or knockdown of *chinmo*, or *fng* marked with RFP expression (magenta) and stained with anti-phospho-S6 (green). RFP (magenta) outlined by white dashed lines marks the expression pattern of *en-Gal4* in the wing disc. (H) Quantification of the size of the phospho-S6 positive region (% of phospho-S6 positive area/disc area) in the wing disc bearing wild-type, *strip*-knockdown cells with or without overexpression of *diap1* or *InR*. ns, not significant; ****$p < 0.0001$; one-way ANOVA with Dunnett's multiple comparison test. Sample size and p value: n=28, p=3.58×10^{-11} (*en>RFP*), n=34 (*en>strip* RNAi), n=27, p=5.99×10^{-2} (*en>strip* RNAi, *InR*$^{\#1}$), n=30, p=3.64×10^{-1} (*en>strip* RNAi, *InR*$^{\#2}$), n=34, p=1.00 (*en>strip* RNAi, *diap1*). Scale bars represent 50 µm in (A–G). Dots represent biological replicates (H); error bars indicate SEM.

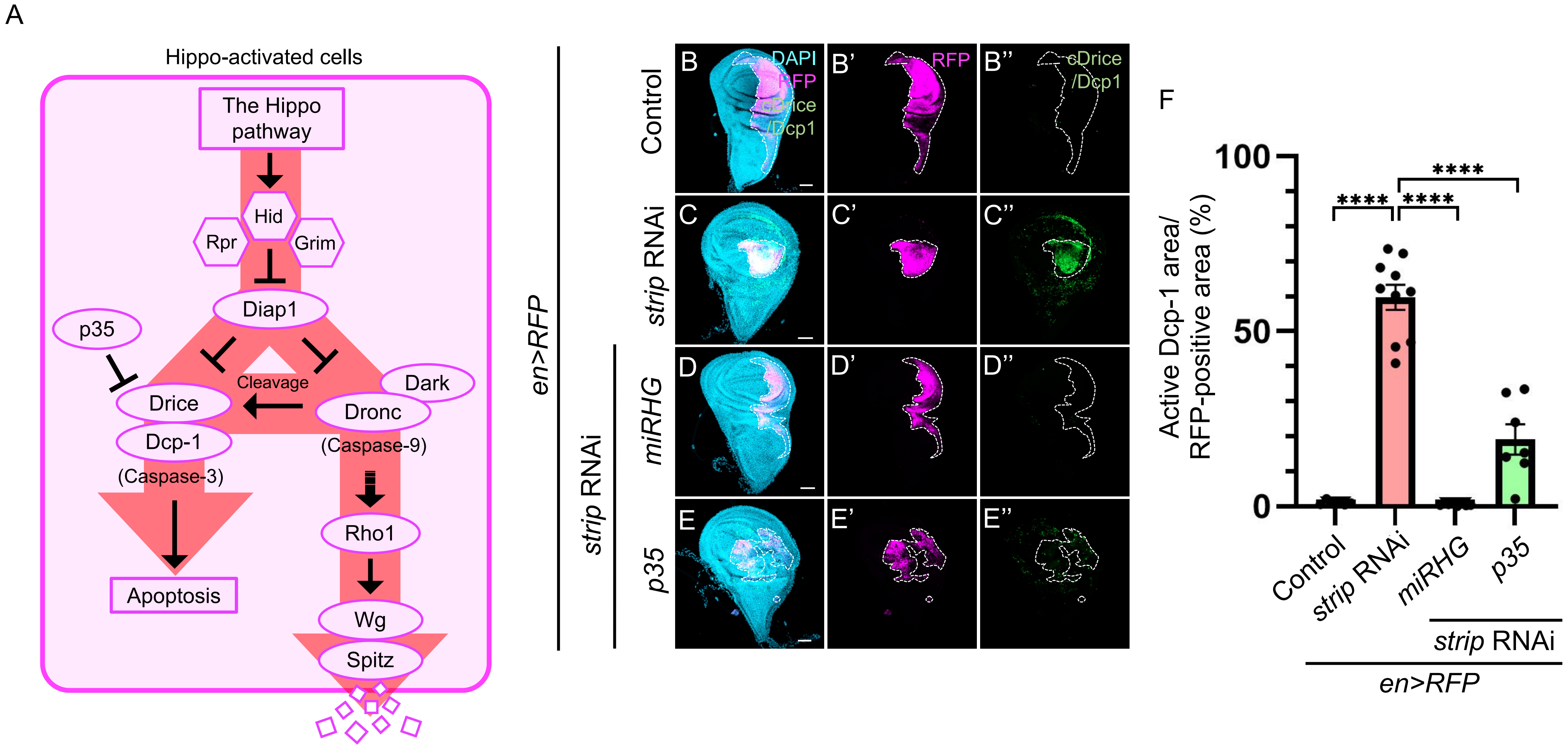

Appendix Figure S3. Hippo activation by *strip* RNAi drives apoptosis signaling

(A) The schematic depiction indicates the signaling pathway of apoptosis and apoptosis-induced proliferation. (B–E) Confocal images show the wing discs bearing wild-type or *strip*-knockdown cells with or without *rpr*, *hid*, and *grim* knockdown or *p35* overexpression marked with RFP expression (magenta) and stained with anti-cleaved Drice/Dcp1 (green). RFP (magenta) outlined by white dashed lines marks the expression pattern of *en-Gal4* in the wing disc. Knockdown of *rpr, hid,* and *grim* was conducted by the expression of microRNAs for *rpr*, *hid*, and *grim* (*miRHG*). (F) Quantification of the size of the cleaved Drice/Dcp1-positive region (% of anti-cleaved Drice/Dcp1-positive area/RFP-positive area) in the wing disc bearing wild-type or *strip*-knockdown cells with or without *rpr*, *hid*, and *grim* knockdown or *p35* overexpression. ns, not significant; ****$p < 0.0001$; one-way ANOVA with Dunnett's multiple comparison test. Sample size and p value: n=6, $p=6.63\times10^{-13}$ (*en>RFP*), n=10 (*en>strip* RNAi), n=8, $p=7.50\times10^{-14}$ (*en>strip* RNAi*, miRHG*), n=7, $p=9.12\times10^{-10}$ (*en>strip* RNAi*, p35*). Scale bars represent 50 μm in (B–E). Dots represent biological replicates (F); error bars indicate SEM.

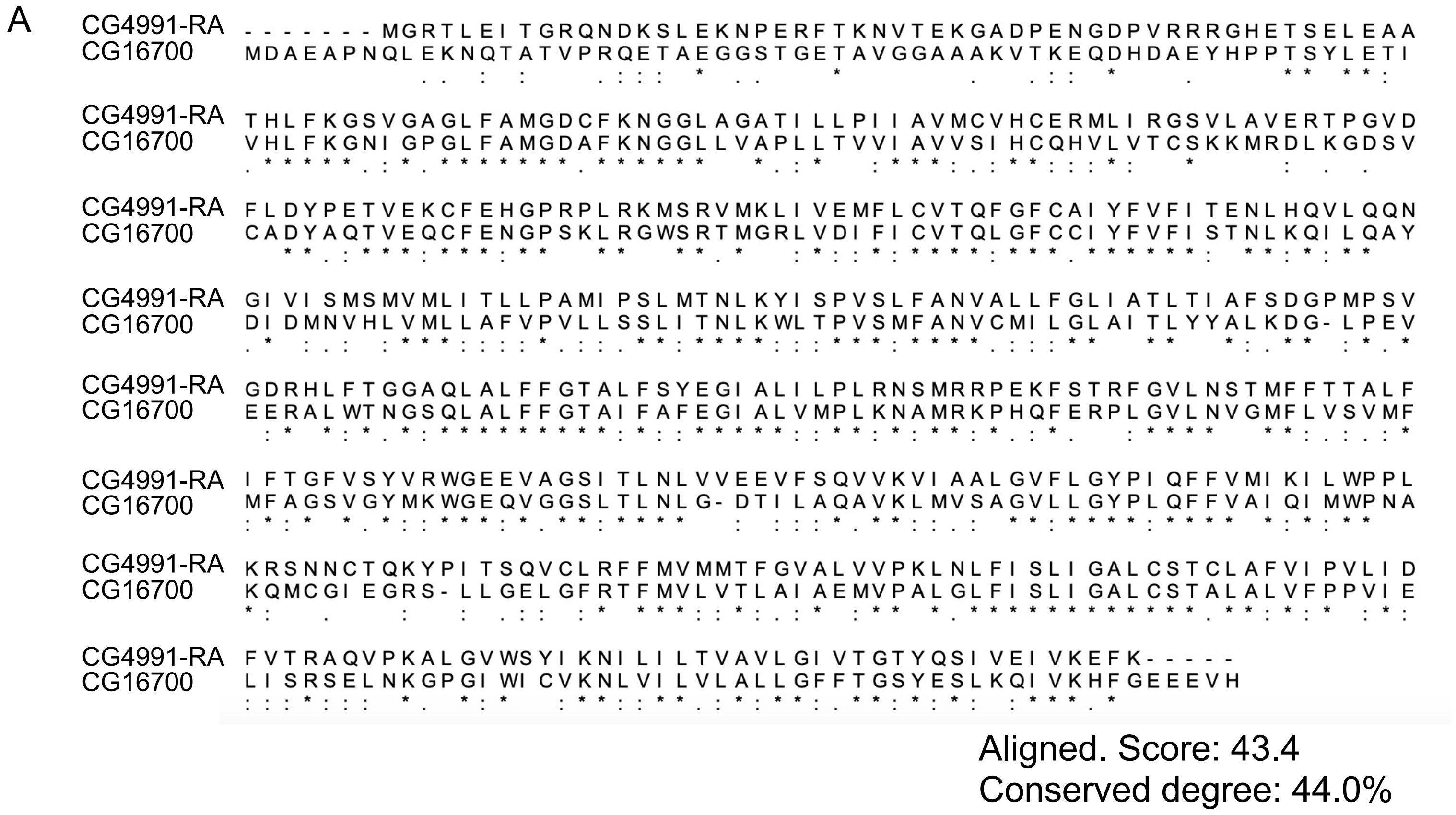

Appendix Figure S4. High similarity of Sat1 and Sat2

(A) The similarity of the amino acid sequence between Sat1 and Sat2. Sat1 and Sat2 were aligned using ClustalW. Asterisk (*): sequence identity; colon (:): strongly similar properties; period (.): weakly similar properties.

Appendix Table S1: Genotype list

Figure 1	
(B, F, G, M, P)	ptc-Gal4/+; UAS-mCD8-RFP/ +
(C, F, G, H, N, P)	ptc-Gal4/ UAS-strip RNAi; UAS-mCD8-RFP/ +
(D, F, G)	ptc-Gal4/ UAS-wts; UAS-mCD8-RFP/ +
(E, F, G, O, P, Q)	ptc-Gal4/ +; UAS-mCD8-RFP/ UAS-yki RNAi
(I, K, L)	en-Gal4, UAS-RFP/ +
(J, K, L)	en-Gal4, UAS-RFP/ UAS-strip RNAi

Figure 2	
(B, E)	nub-Gal4/ +; UAS-mCD8-RFP/ +
(C, E)	nub-Gal4/ UAS-strip RNAi ; UAS-mCD8-RFP/ +
(D, E)	nub-Gal4/ +; UAS-mCD8-RFP/ UAS-yki RNAi
(F, H)	pnr-Gal4/ UAS-mCD8-RFP
(G, H)	UAS-strip RNAi/ + ; pnr-Gal4/ UAS-mCD8-RFP
(I, L)	41D11-Gal4, UAS-GFP/ +
(J, L)	UAS-strip RNAi/ +; 41D11-Gal4, UAS-GFP/ +
(K, L)	41D11-Gal4, UAS-GFP/ UAS-yki RNAi
(M, N)	hs-FLP/ + or y; Act>y>Gal4, UAS-GFP/ UAS-strip RNAi

Figure 3	
(B, J, K)	en-Gal4, UAS-RFP/ +
(C, J)	en-Gal4, UAS-RFP/ UAS-strip RNAi
(D, J)	en-Gal4, UAS-RFP/ UAS-strip RNAi; UAS-hippo RNAi/ +
(E, J)	en-Gal4, UAS-RFP/ UAS-strip RNAi; UAS-bantam/ +
(F, J)	en-Gal4, UAS-RFP/ UAS-strip RNAi; UAS-cyclinE/ +
(G, J)	en-Gal4, UAS-RFP/ UAS-strip RNAi; UAS-atg8a RNAi/ +
(H, K)	UAS-strip RNAi/ + or y; en-Gal4, UAS-RFP/ +
(I, K)	UAS-strip RNAi/ + or y; en-Gal4, UAS-RFP/ UAS-miRHG
(J)	en-Gal4, UAS-RFP/ UAS-strip RNAi; UAS-myc/ +
(J)	en-Gal4, UAS-RFP/ UAS-strip RNAi; UAS-brk/ +
(J)	en-Gal4, UAS-RFP/ UAS-strip RNAi; UAS-chinmo RNAi/ +
(J)	en-Gal4, UAS-RFP/ UAS-strip RNAi; UAS-fng RNAi/ +

Figure 4	
(A, F)	en-Gal4, UAS-RFP/ UAS-strip RNAi
(B, F)	en-Gal4, UAS-RFP/ UAS-strip RNAi; UAS-dark RNAi/ +
(C, F)	en-Gal4, UAS-RFP/ UAS-strip RNAi; UAS-dronc RNAi/ +
(D, G)	UAS-strip RNAi/ + or y ;en-Gal4, UAS-RFP/ UAS-p35
(E, F)	en-Gal4, UAS-RFP/ UAS-strip RNAi; UAS-rho1 RNAi/ +
(G)	UAS-strip RNAi/ + or y ;en-Gal4, UAS-RFP/ +
(H, K)	ptc-Gal4/ UAS-strip RNAi; UAS-mCD8-RFP/ +
(I, K)	ptc-Gal4/ UAS-strip RNAi; UAS-mCD8-RFP/ UAS-wg RNAi
(J, K)	ptc-Gal4/ UAS-strip RNAi; UAS-mCD8-RFP/ UAS-spitz RNAi
(L, N–P)	en-Gal4, UAS-RFP/ +
(M, N–P)	UAS-strip RNAi/ + or y ;en-Gal4, UAS-RFP/ UAS-p35

Appendix Table S1: Genotype list (continued)

Figure 5	
(B–F)	en-Gal4, UAS-RFP/ UAS-strip RNAi; tub>Gal80ts/ +

Figure 6	
(C, G, H, L)	en-Gal4, UAS-RFP/ +
(D, G)	en-Gal4, UAS-RFP/ UAS-hid; UAS-p35/ +
(E, G)	en-Gal4, UAS-RFP/ UAS-rpr, UAS-p35
(F, G)	UAS-strip RNAi/ + or y ;en-Gal4, UAS-RFP/ UAS-p35
(I, L)	en-Gal4, UAS-RFP/ UAS-p35; UAS-bantam sp/ +
(J, L)	UAS-hid/ +; en-Gal4, UAS-RFP/ UAS-p35
(K, L)	UAS-hid/ +; en-Gal4, UAS-RFP/ UAS-p35; UAS-bantam sp/ +

Figure 7	
(A, B, G, H, I, L)	en-Gal4, UAS-RFP/ +
(A)	en-Gal4, UAS-RFP/ +; UAS-CG4991/ +
(C, G, H, J, L, M–P)	UAS-strip RNAi/ + or y; en-Gal4, UAS-RFP/ +
(D, G, H)	UAS-strip RNAi/ + or y; en-Gal4, UAS-RFP/ +; UAS-CG4991 RNAi/ +
(E, G, H)	UAS-strip RNAi/ + or y; en-Gal4, UAS-RFP/ UAS-CG16700 RNAi
(F, G, H, K, L)	UAS-strip RNAi/ + or y; en-Gal4,UAS-RFP/ UAS-CG16700 RNAi; UAS-CG4991RNAi

Figure 8	
(A, F, G, N, O)	+/ y; en-Gal4, UAS-RFP/ +
(B, F, G, H, N, O)	+/ y; en-Gal4, UAS-RFP/ UAS-strip RNAi
(C, F, G)	+/ y; en-Gal4, UAS-RFP/ UAS-strip RNAi; UAS-wg RNAi/ +
(D, F, G, J, N, O)	sat1Δ/2Δ (line1)/ y; en-Gal4, UAS-RFP/ UAS-strip RNAi
(E, F, G)	sat1Δ/2Δ (line1)/ y; en-Gal4, UAS-RFP/ UAS-strip RNAi; UAS-wg RNAi/ +
(I, N, O)	+/ y; en-Gal4, UAS-RFP/ UAS-strip RNAi; UAS-spitz RNAi/ +
(K, N, O)	sat1Δ/2Δ (line2)/ y; en-Gal4, UAS-RFP/ UAS-strip RNAi
(L, N, O)	sat1Δ/2Δ (line1)/ y; en-Gal4, UAS-RFP/ UAS-strip RNAi; UAS-spitz RNAi/ +
(M, N, O)	sat1Δ/2Δ (line2)/ y; en-Gal4, UAS-RFP/ UAS-strip RNAi; UAS-spitz RNAi/ +

Figure EV1	
(B, E)	en-Gal4, UAS-RFP/ +; UAS-FLP, Ubi-FRT-STOP-FRT-GFP
(C–H)	UAS-strip RNAi/ + or y; en-Gal4, UAS-RFP/ +; UAS-FLP, Ubi-FRT-STOP-FRT-GFP
(I, K)	en-Gal4, UAS-RFP/ +
(J, K)	UAS-strip RNAi/ + or y; en-Gal4, UAS-RFP/ +

Figure EV2	
(A, C)	ptc-Gal4/ UAS-strip RNAi; UAS-mCD8-RFP/ +
(B)	ptc-Gal4/ +; UAS-mCD8-RFP/ UAS-yki RNAi
(D, F)	en-Gal4, UAS-RFP/ puc-stinger
(E, F)	UAS-strip RNAi/ + or y; en-Gal4, UAS-RFP/ puc-stinger
(G, I, J, L)	en-Gal4, UAS-RFP/ +
(H, I, K–N)	UAS-strip RNAi/ + or y; en-Gal4, UAS-RFP/ +

Appendix Table S1: Genotype list (continued)

Figure EV3	
(C, F)	en-Gal4, UAS-RFP/ +
(D, F)	UAS-strip RNAi/ + or y; en-Gal4, UAS-RFP/ +
(E, F)	UAS-strip RNAi/ + or y; en-Gal4, UAS-RFP/ UAS-miRHG
(H, L)	+/ +; nub-Gal4/ UAS-mCD8-GFP
(I, L)	+/ +; UAS-strip RNAi; nub-Gal4/ UAS-mCD8-GFP
(J, L)	hs-FLP/ +; Act>y>Gal4, UAS-GFP
(K, L)	hs-FLP/ UAS-strip RNAi; Act>y>Gal4, UAS-GFP

Figure EV4	
(B–O)	en-Gal4, UAS-RFP/ UAS-strip RNAi; tub>Gal80ts/ +

Figure EV5	
(B)	OK6-Gal4/ UAS-strip RNAi
(B)	sat1Δ (line1)/ + or y; OK6-Gal4/ UAS-strip RNAi
(B)	sat1Δ (line2)/ + or y; OK6-Gal4/ UAS-strip RNAi
(C, D)	pMT-puro
(E, F)	pMT-Flag-Sat1-puro (line1)
(G, H)	pMT-Flag-Sat1-puro (line2)
(J, N)	+/ y; en-Gal4, UAS-RFP/ +
(K, N)	sat1Δ/2Δ (line1)/ y; en-Gal4, UAS-RFP/ +
(L, N)	+/ y; en-Gal4, UAS-RFP/ UAS-strip RNAi
(M, N)	sat1Δ/2Δ (line1)/ y; en-Gal4, UAS-RFP/ UAS-strip RNAi

Appendix Figure S1	
(A, C)	ci-Gal4, UAS-GFP/ +
(B, C)	ci-Gal4, UAS-GFP/ UAS-strip RNAi
(D, F)	bx-Gal4/ + or y; ; UAS-mCD8-RFP/ +
(E, F)	bx-Gal4/ + or y; UAS-strip RNAi/ +; UAS-mCD8-RFP/ +
(G, I)	284-Gal4/ + or y; ; UAS-mCD8-RFP/ +
(H, I)	284-Gal4/ + or y; UAS-strip RNAi/ +; UAS-mCD8-RFP/ +

Appendix Figure S2	
(A)	en-Gal4, UAS-RFP/ UAS-strip RNAi; UAS-myc/ +
(B)	en-Gal4, UAS-RFP/ UAS-strip RNAi; UAS-brk/ +
(C)	en-Gal4, UAS-RFP/ UAS-strip RNAi; UAS-chimo RNAi/ +
(D)	en-Gal4, UAS-RFP/ UAS-strip RNAi; UAS-fng RNAi/ +
(E, H)	en-Gal4, UAS-RFP/ UAS-strip RNAi; UAS-diap1/ +
(F, H)	en-Gal4, UAS-RFP/ UAS-strip RNAi; UAS-InR#1/ +
(G, H)	en-Gal4, UAS-RFP/ UAS-strip RNAi; UAS-InR#2/ +

Appendix Figure S3	
(B, F)	en-Gal4, UAS-RFP/ +
(C, F)	UAS-strip RNAi/ + or y; en-Gal4, UAS-RFP/ +
(D, F)	UAS-strip RNAi/ + or y; en-Gal4, UAS-RFP/ UAS-miRHG
(E, F)	UAS-strip RNAi/ + or y ;en-Gal4, UAS-RFP/ UAS-p35
