# Supplementary figures and images for: The Hippo tumor suppressor pathway triggers non-cell autonomous tumorigenesis in Drosophila
Source: EMBO Rep. 2026 May 1;27(11):2915–46. doi: 10.1038/s44319-026-00778-5 (PMC13261145; doi:10.1038/s44319-026-00778-5)

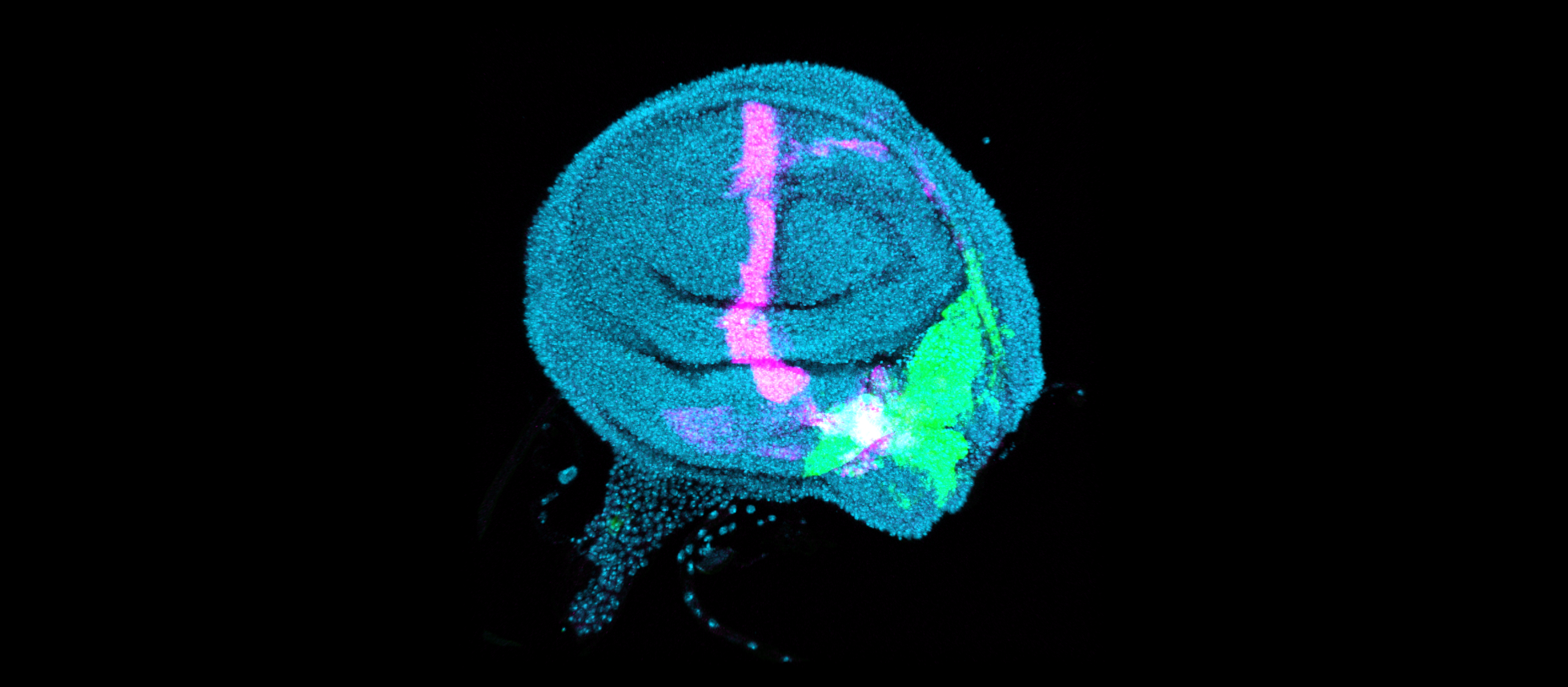

Supplement: Supplementary file 3 — Source data Fig. 1 [file 44319_2026_778_MOESM3_ESM.zip › Figure 1/1H/Fig.1H.tif]

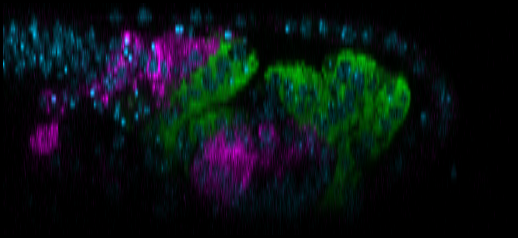

Supplement: Supplementary file 3 — Source data Fig. 1 [file 44319_2026_778_MOESM3_ESM.zip › Figure 1/1H/Fig.1H vertical section.tif]

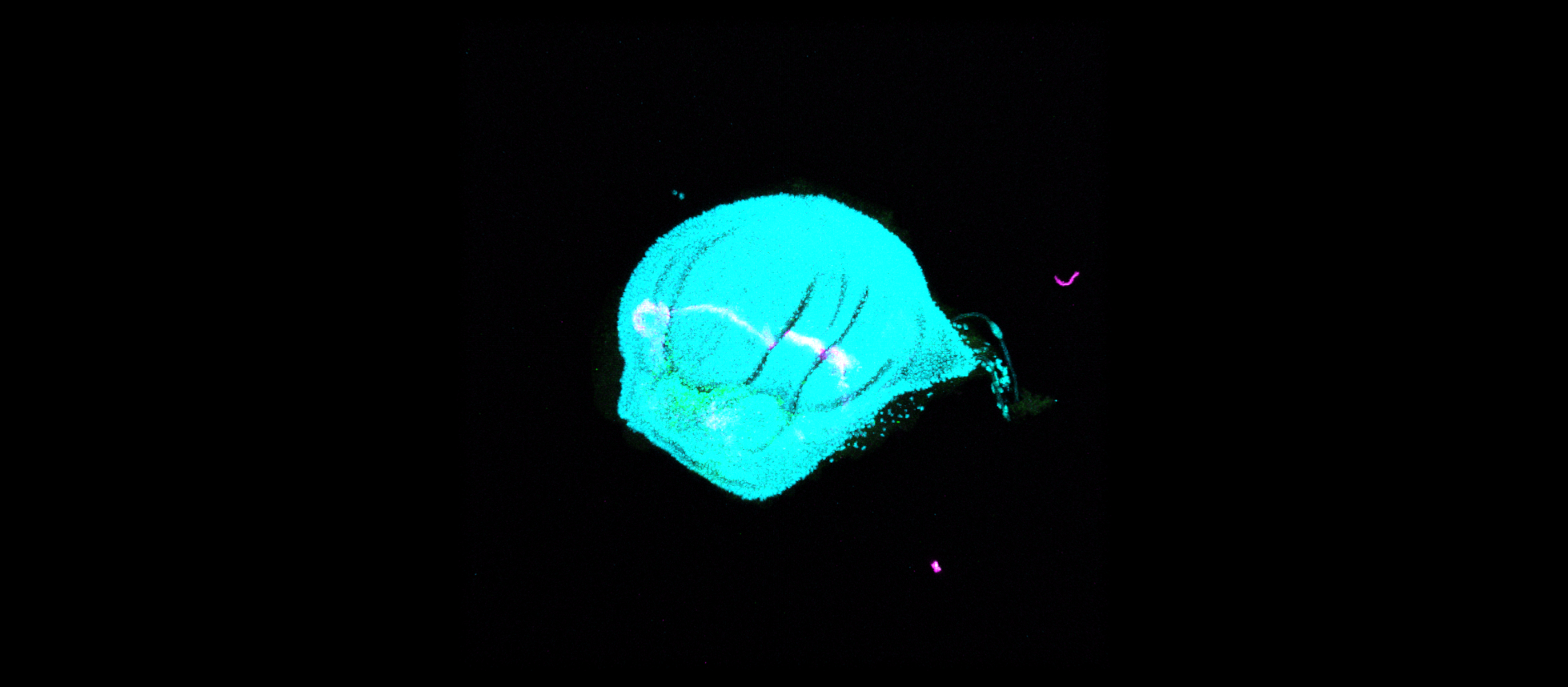

Supplement: Supplementary file 3 — Source data Fig. 1 [file 44319_2026_778_MOESM3_ESM.zip › Figure 1/1O/Fig.1O.tif]

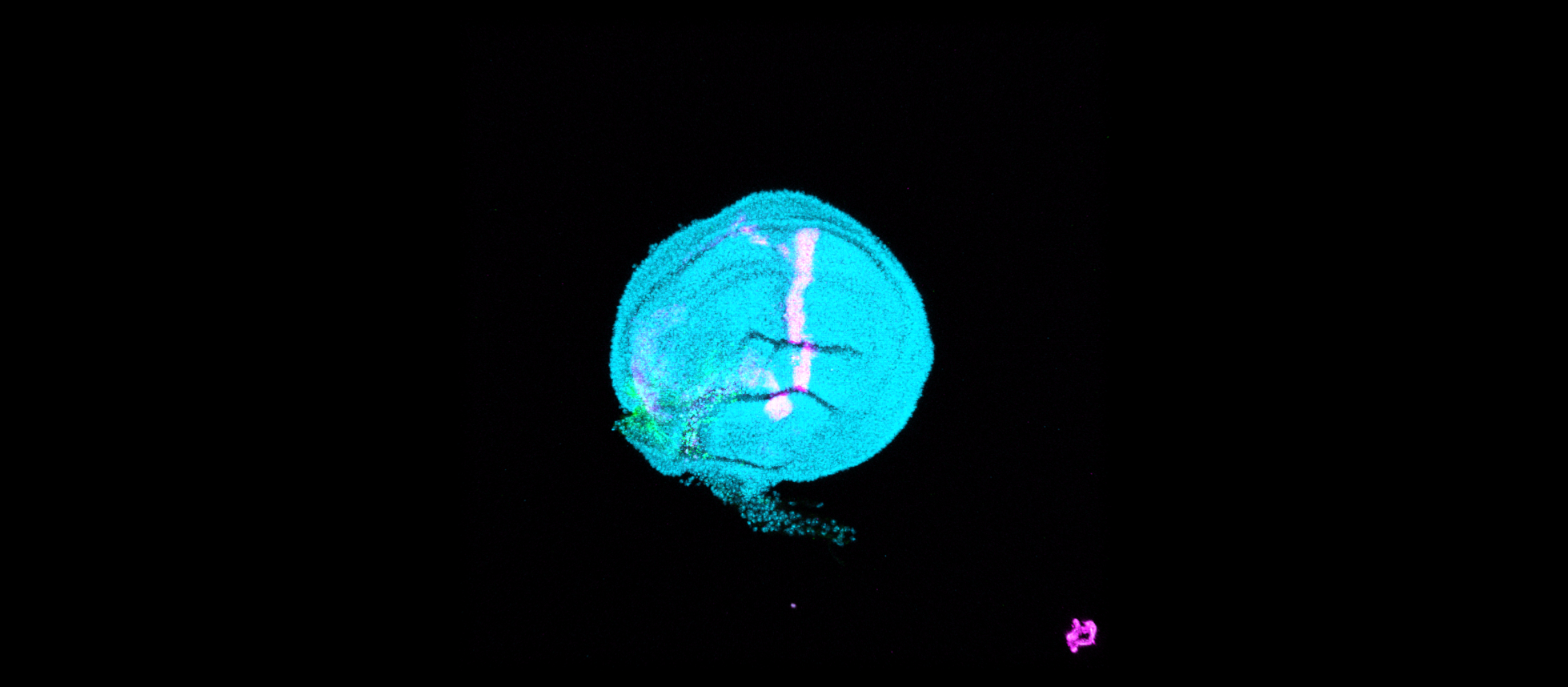

Supplement: Supplementary file 3 — Source data Fig. 1 [file 44319_2026_778_MOESM3_ESM.zip › Figure 1/1N/Fig.1N.tif]

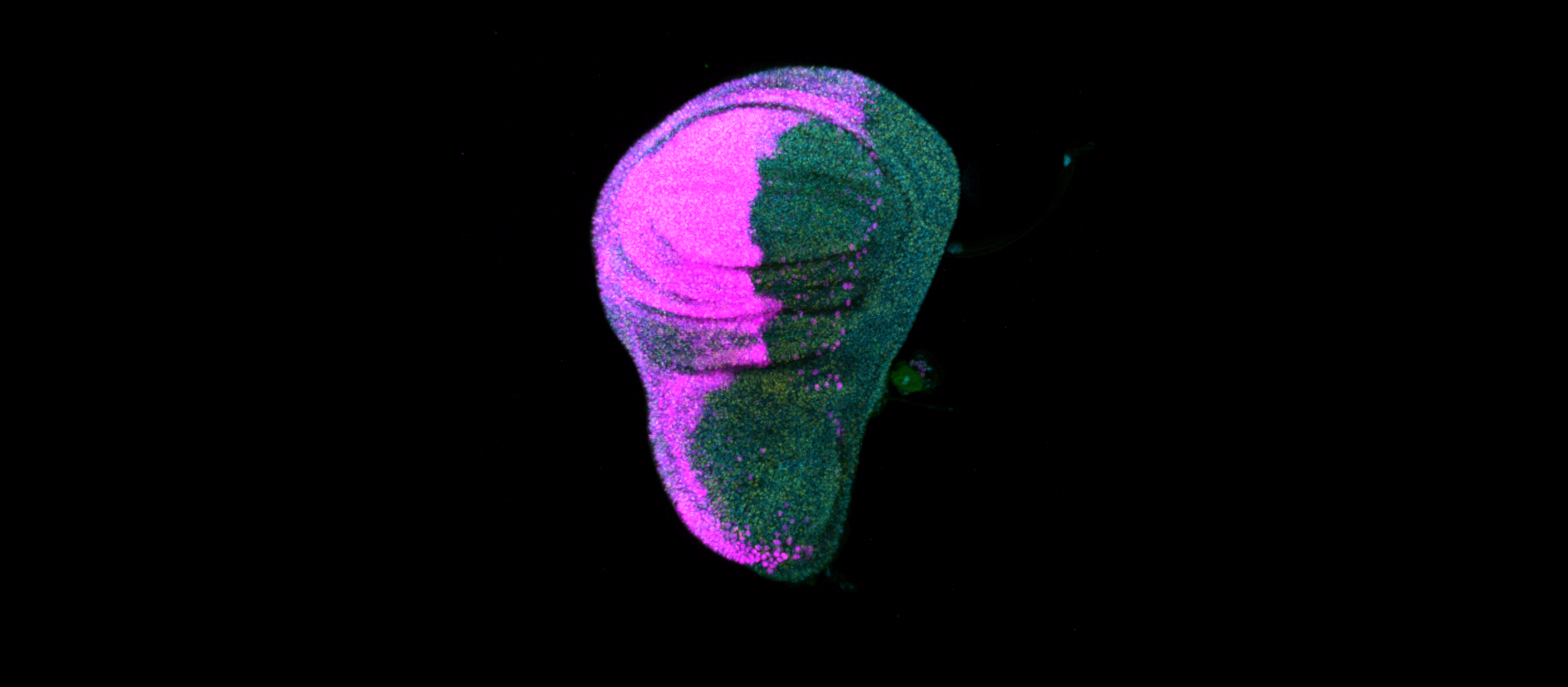

Supplement: Supplementary file 3 — Source data Fig. 1 [file 44319_2026_778_MOESM3_ESM.zip › Figure 1/1I/Fig1I.tif]

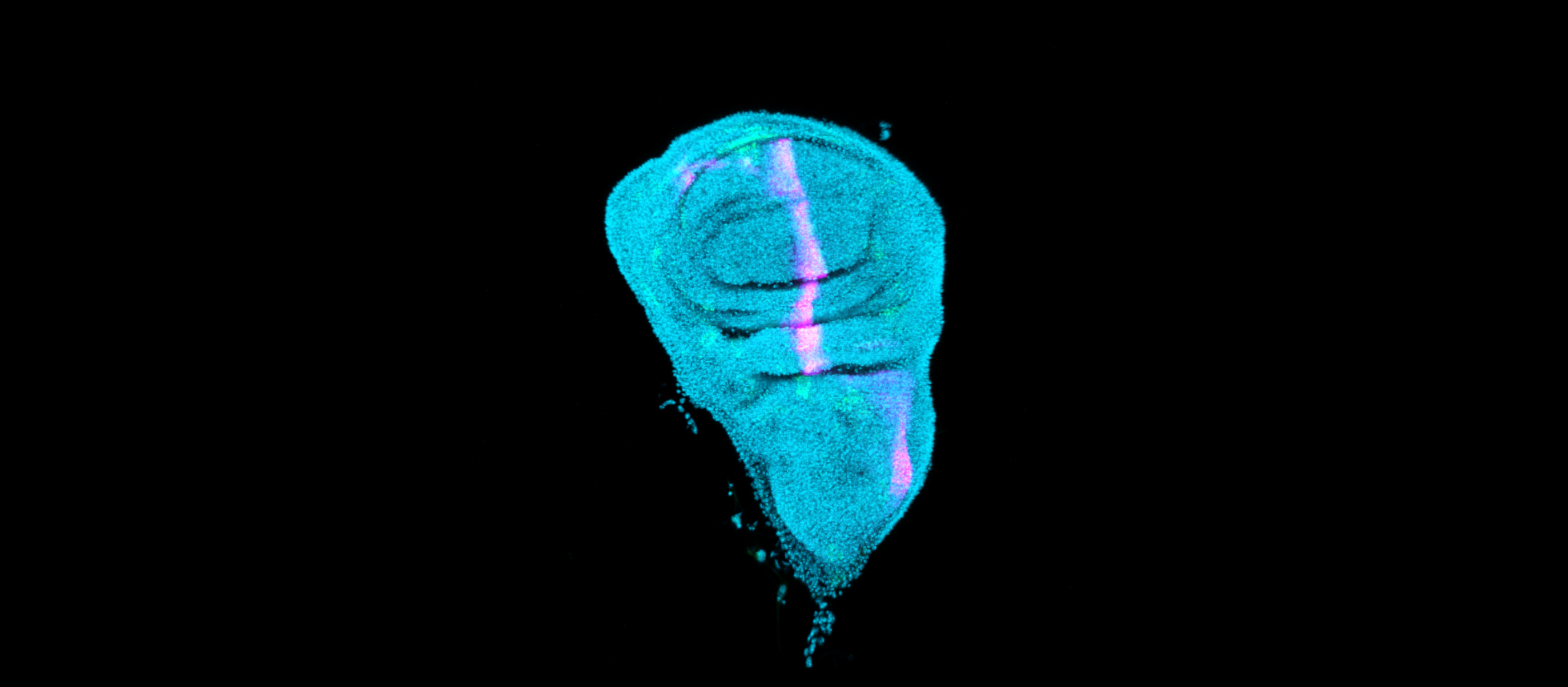

Supplement: Supplementary file 3 — Source data Fig. 1 [file 44319_2026_778_MOESM3_ESM.zip › Figure 1/1B/Fig.1B.tif]

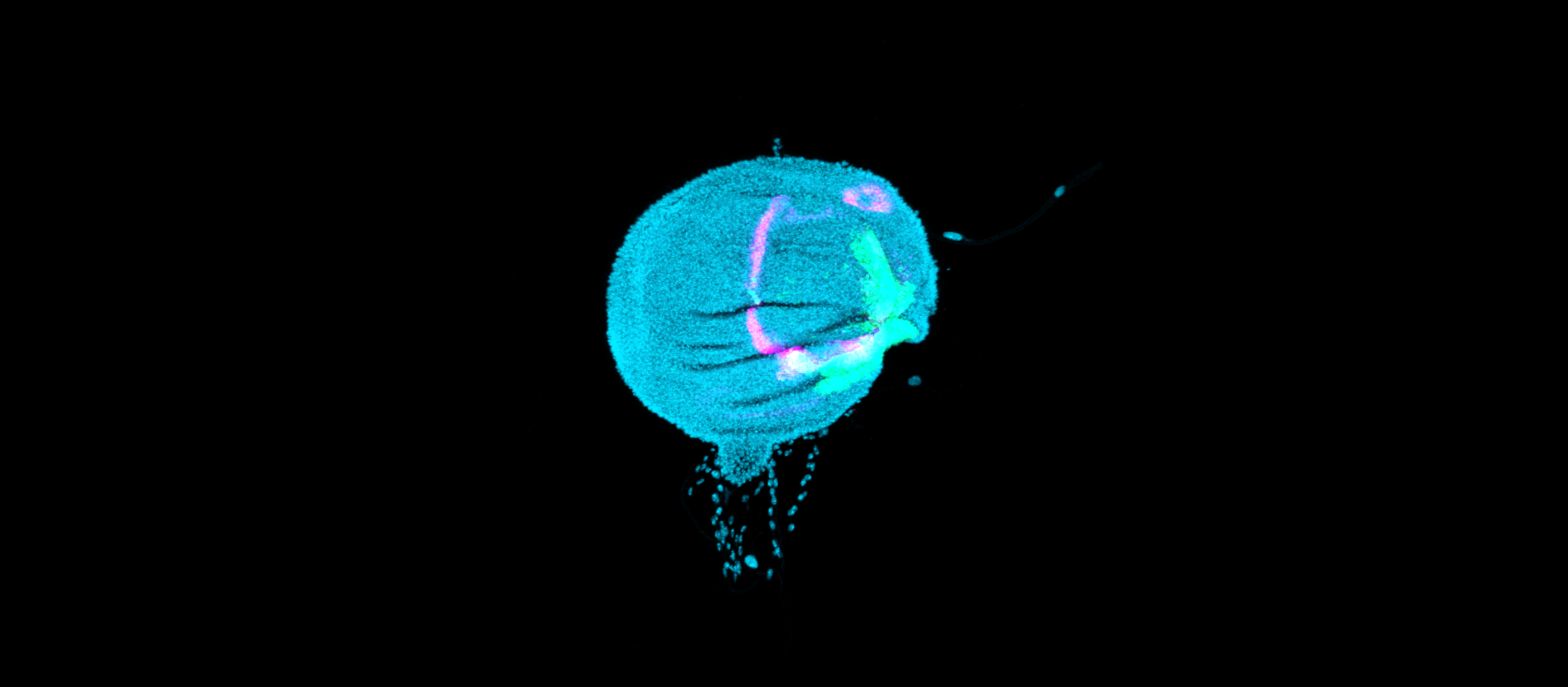

Supplement: Supplementary file 3 — Source data Fig. 1 [file 44319_2026_778_MOESM3_ESM.zip › Figure 1/1E/Fig.1E.tif]

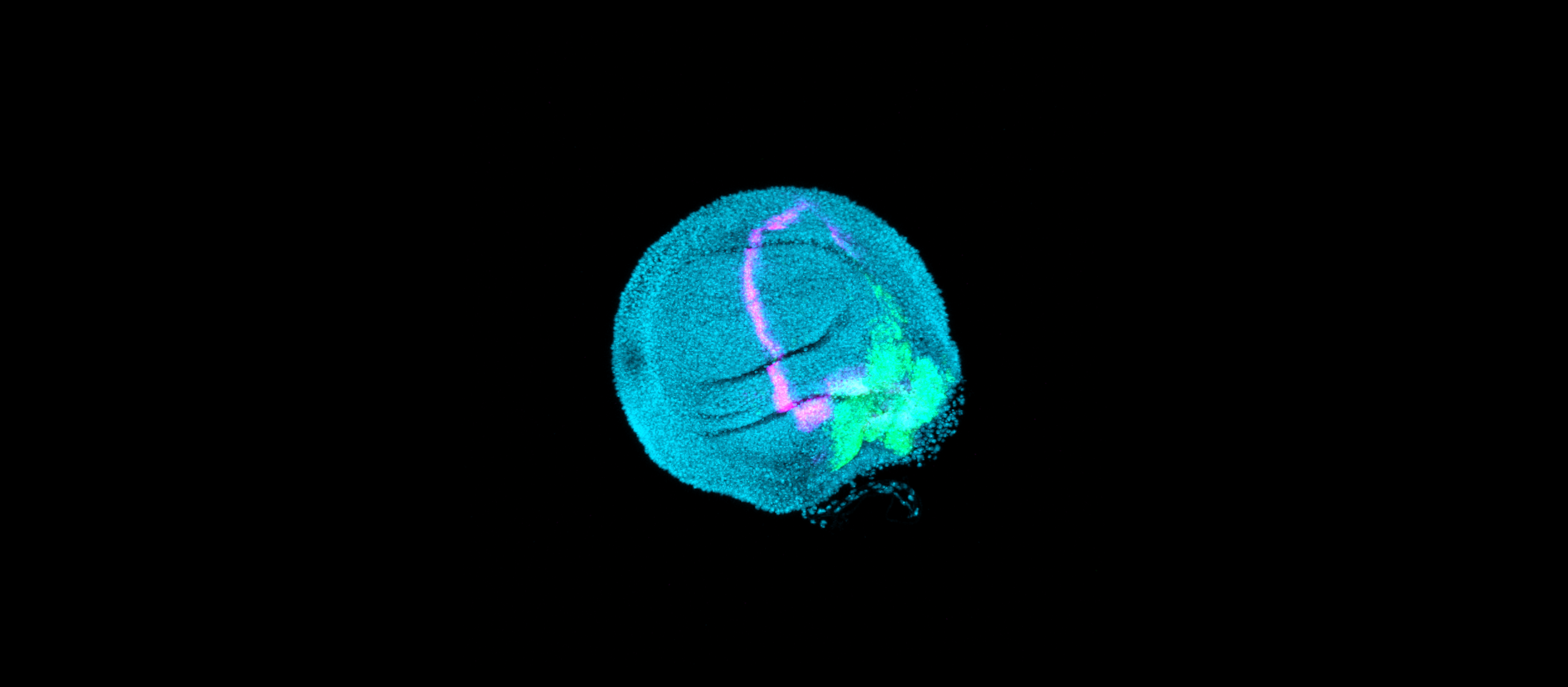

Supplement: Supplementary file 3 — Source data Fig. 1 [file 44319_2026_778_MOESM3_ESM.zip › Figure 1/1D/Fig.1D.tif]

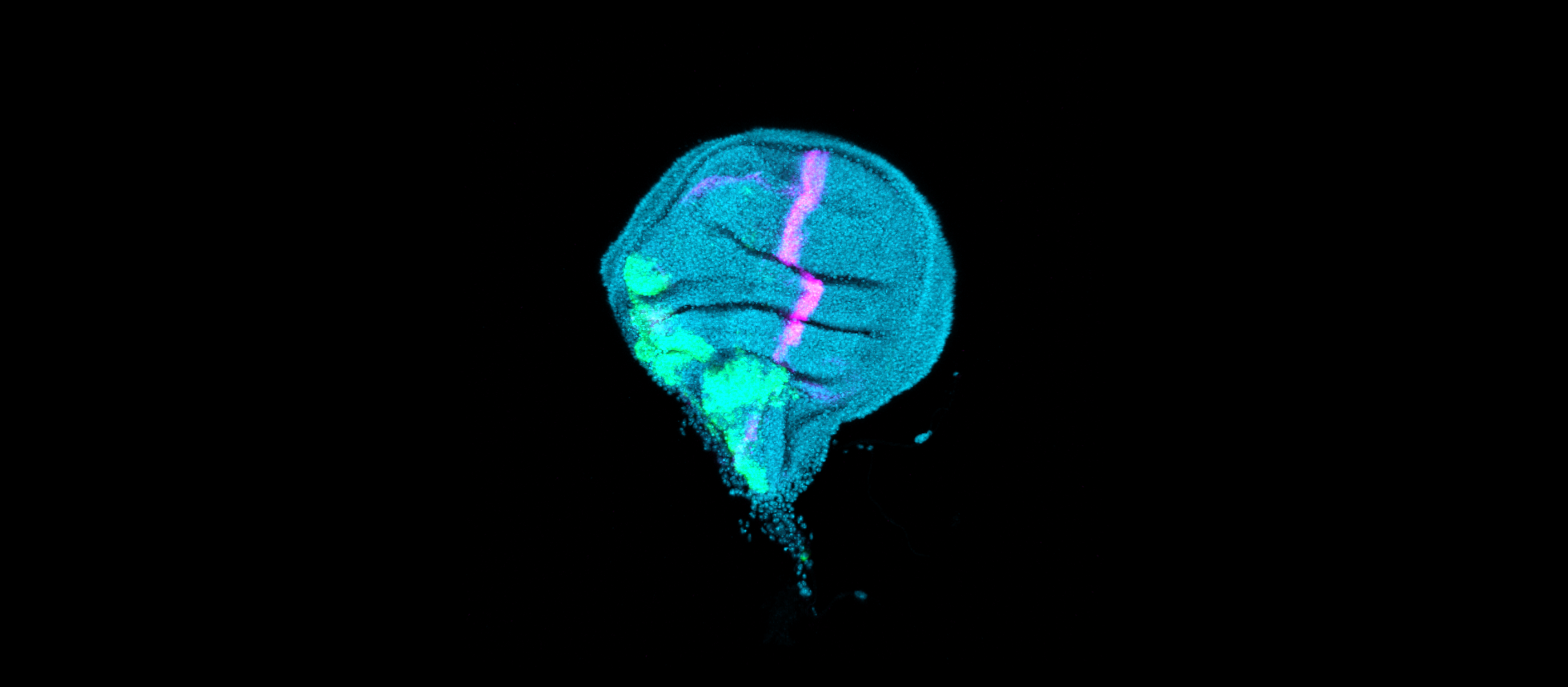

Supplement: Supplementary file 3 — Source data Fig. 1 [file 44319_2026_778_MOESM3_ESM.zip › Figure 1/1C/Fig.1C.tif]

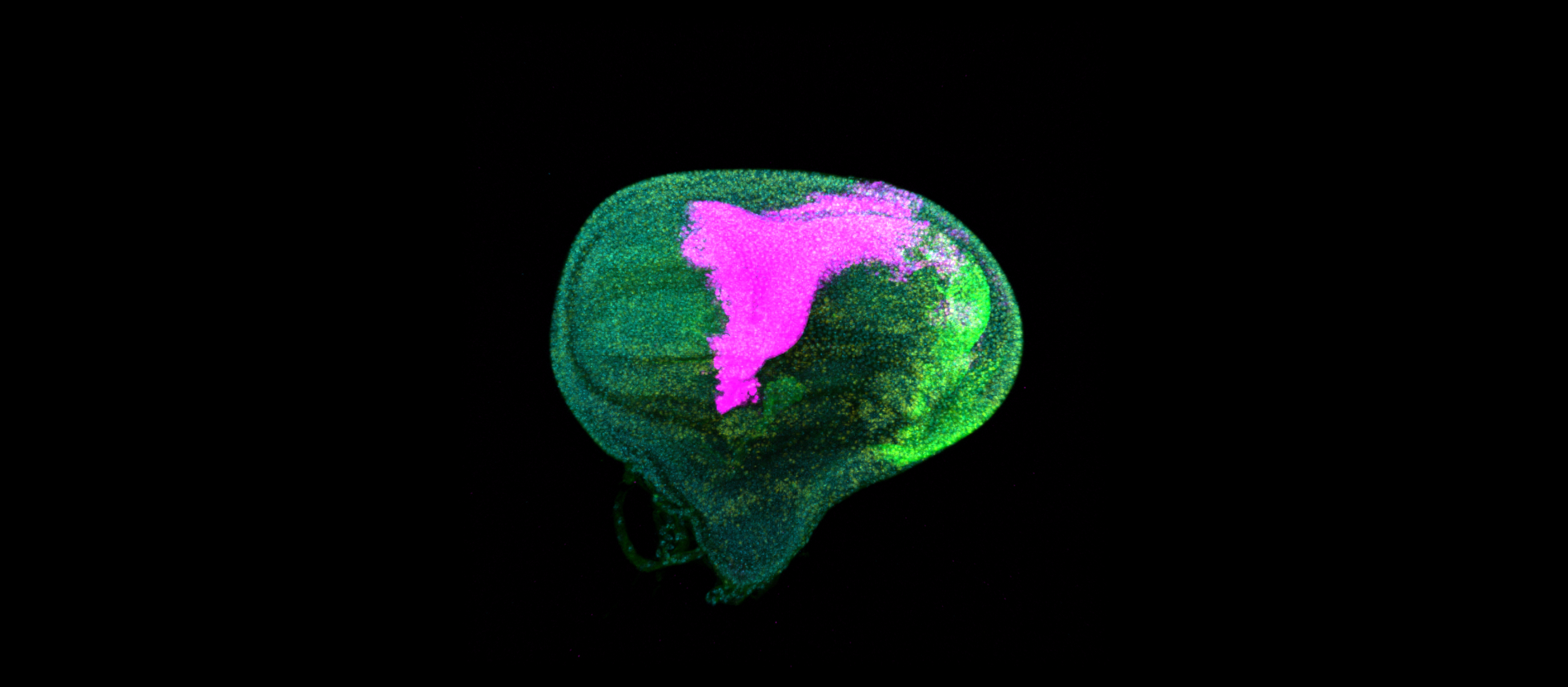

Supplement: Supplementary file 3 — Source data Fig. 1 [file 44319_2026_778_MOESM3_ESM.zip › Figure 1/1J/Fig.1J.tif]

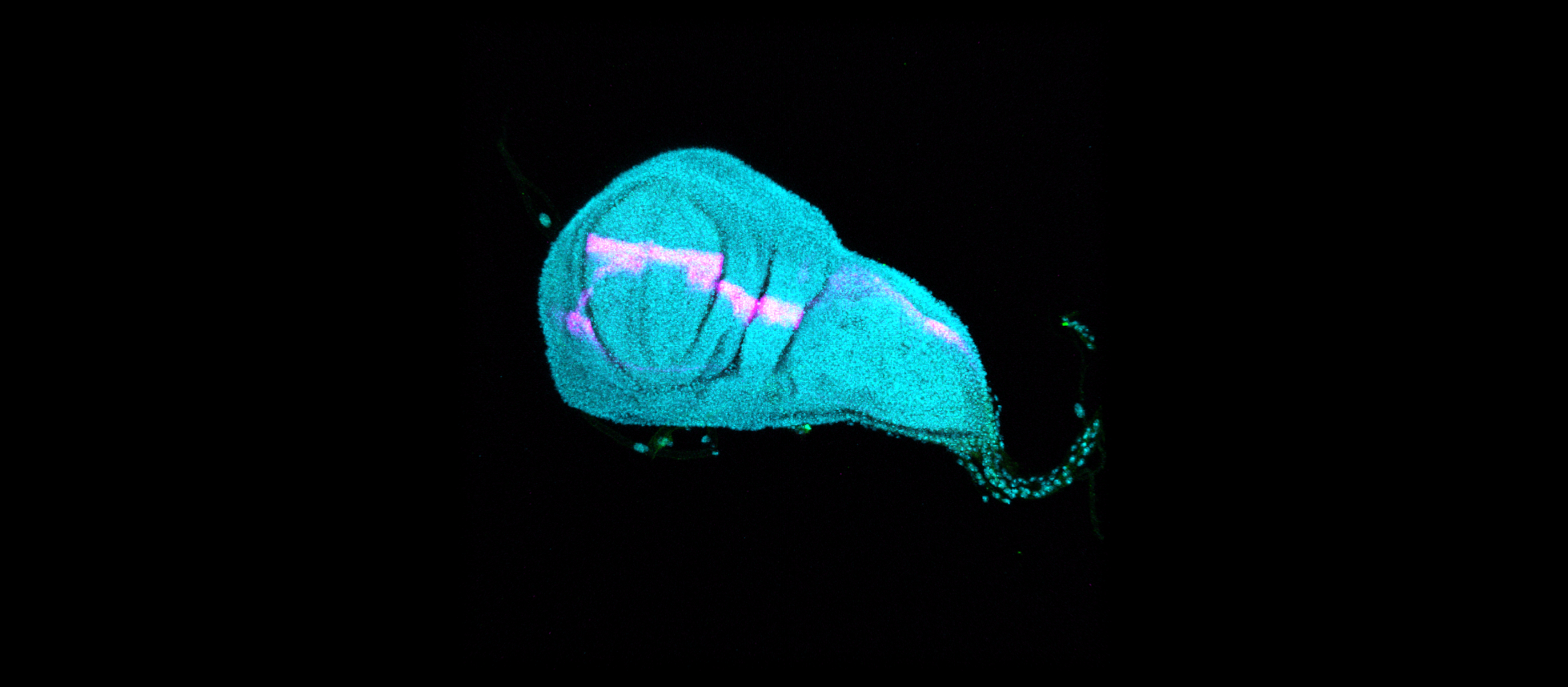

Supplement: Supplementary file 3 — Source data Fig. 1 [file 44319_2026_778_MOESM3_ESM.zip › Figure 1/1M/Fig.1M.tif]

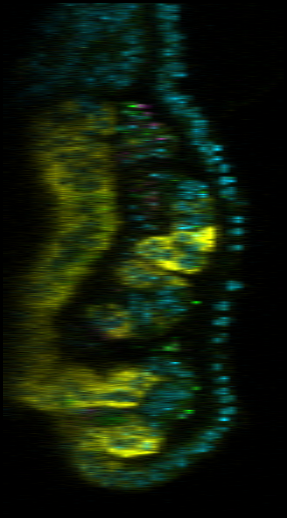

Supplement: Supplementary file 3 — Source data Fig. 1 [file 44319_2026_778_MOESM3_ESM.zip › Figure 1/1Q/Fig.1Q_c1-4.tif]

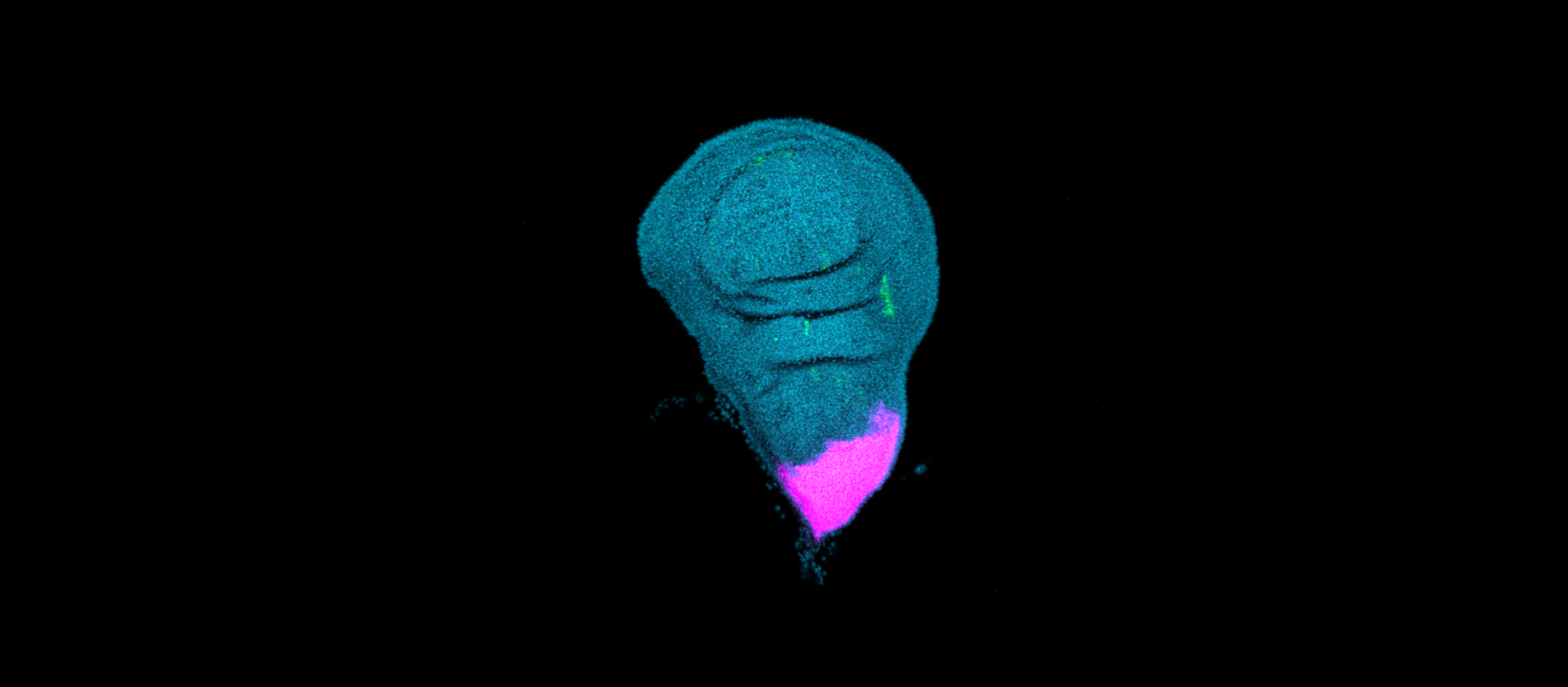

Supplement: Supplementary file 4 — Source data Fig. 2 [file 44319_2026_778_MOESM4_ESM.zip › Figure 2/2G/Fig.2G.tif]

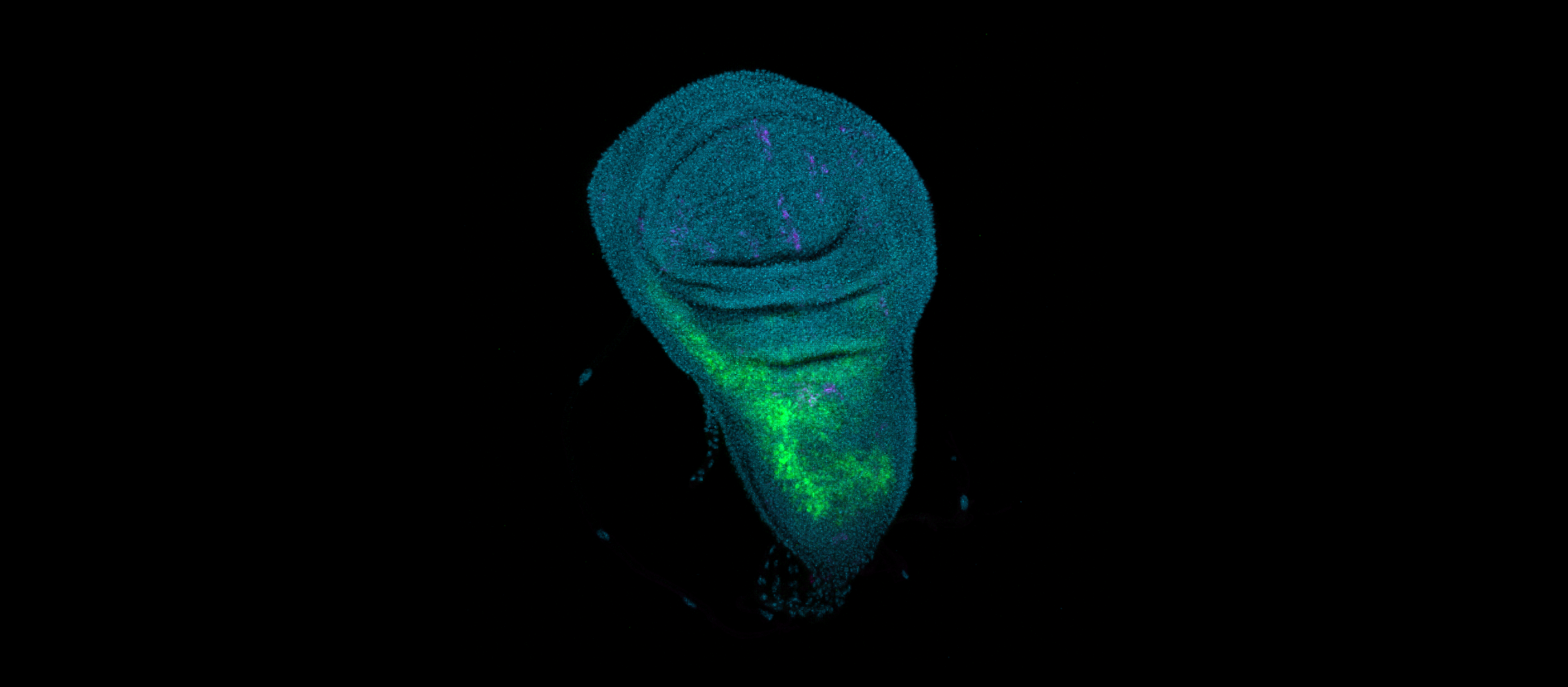

Supplement: Supplementary file 4 — Source data Fig. 2 [file 44319_2026_778_MOESM4_ESM.zip › Figure 2/2I/Fig.2I.tif]

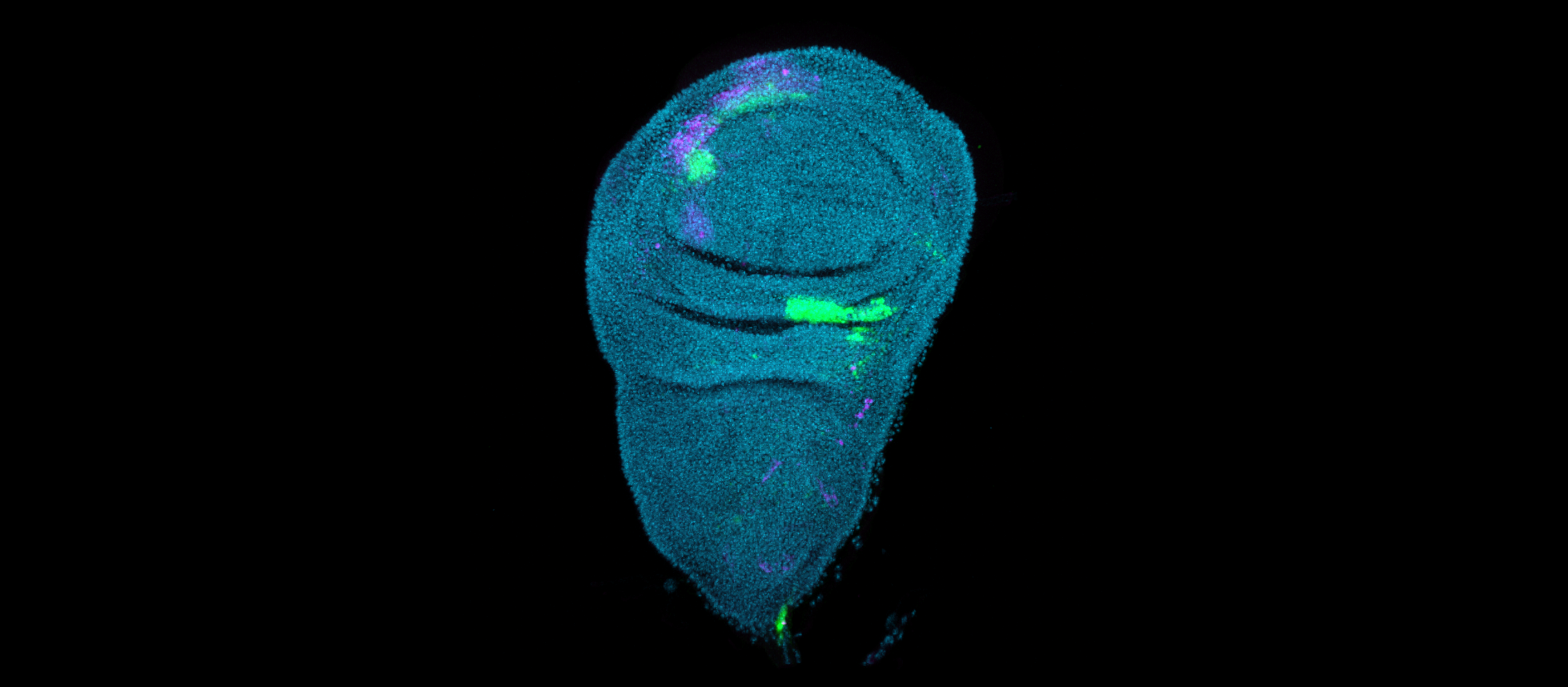

Supplement: Supplementary file 4 — Source data Fig. 2 [file 44319_2026_778_MOESM4_ESM.zip › Figure 2/2N/Fig.2N.tif]

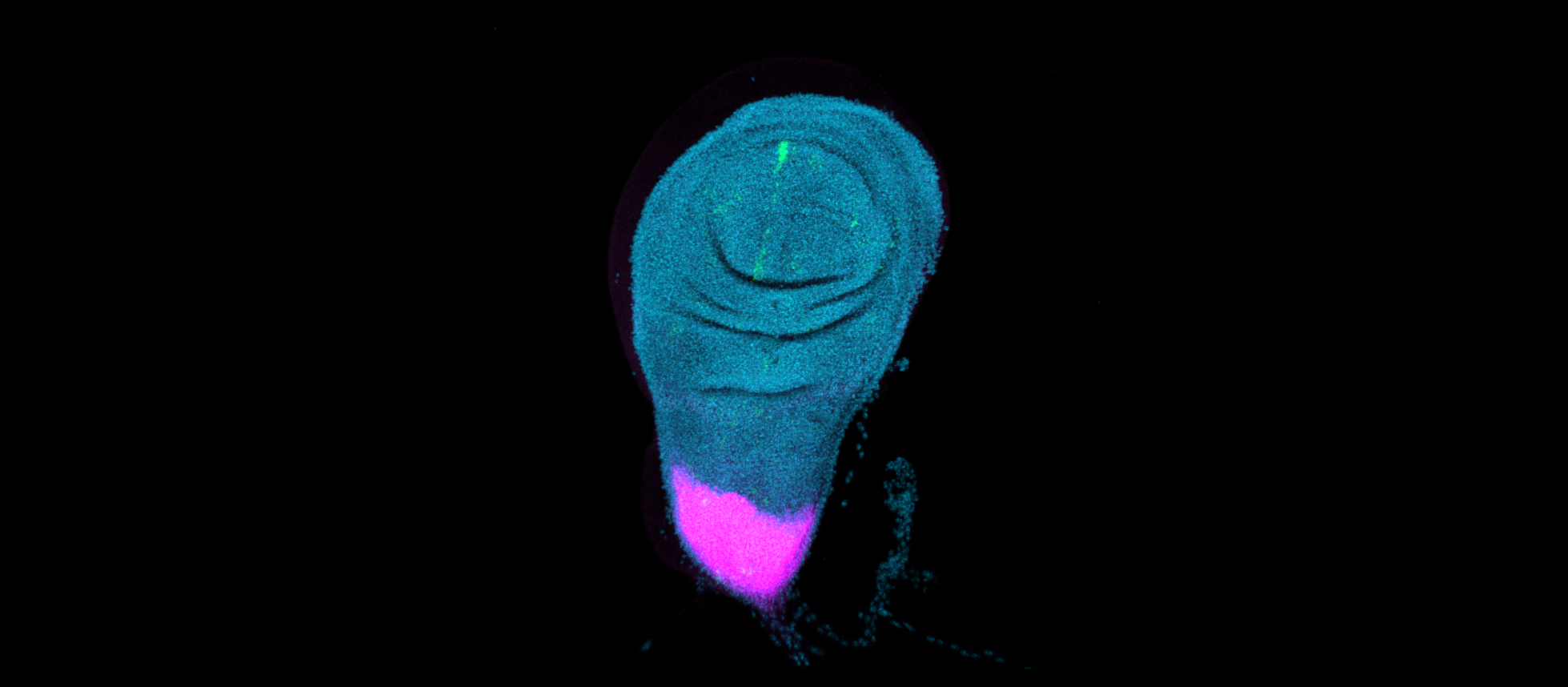

Supplement: Supplementary file 4 — Source data Fig. 2 [file 44319_2026_778_MOESM4_ESM.zip › Figure 2/2F/Fig.2F.tif]

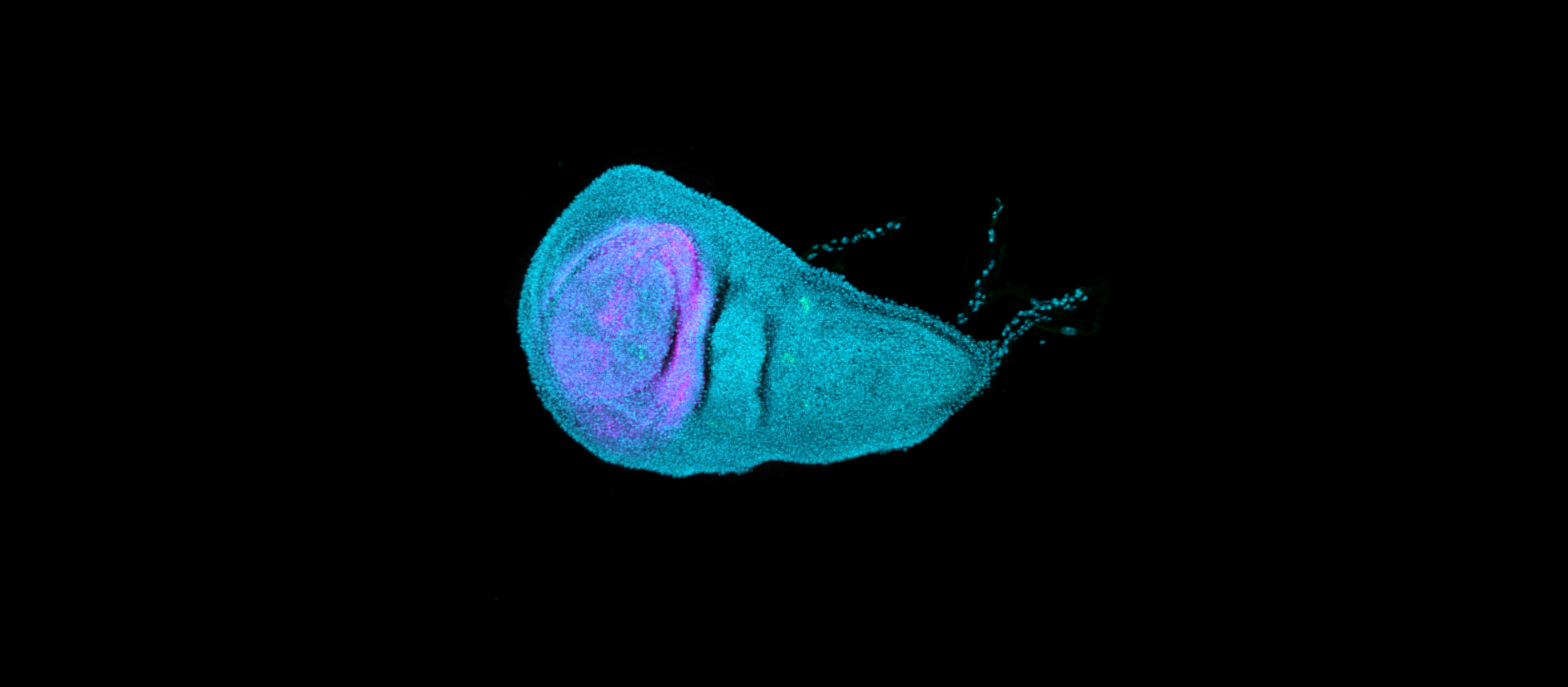

Supplement: Supplementary file 4 — Source data Fig. 2 [file 44319_2026_778_MOESM4_ESM.zip › Figure 2/2C/Fig.2C.tif]

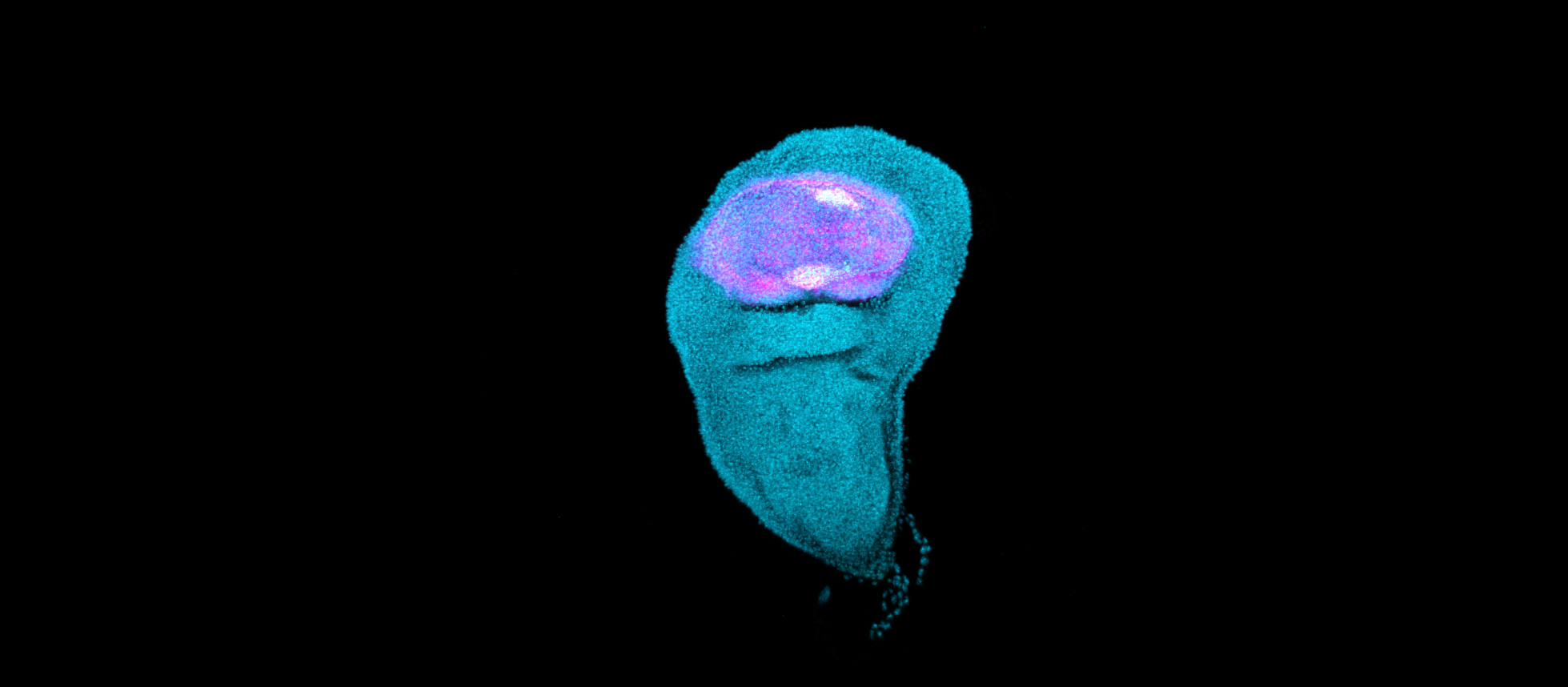

Supplement: Supplementary file 4 — Source data Fig. 2 [file 44319_2026_778_MOESM4_ESM.zip › Figure 2/2D/Fig.2D.tif]

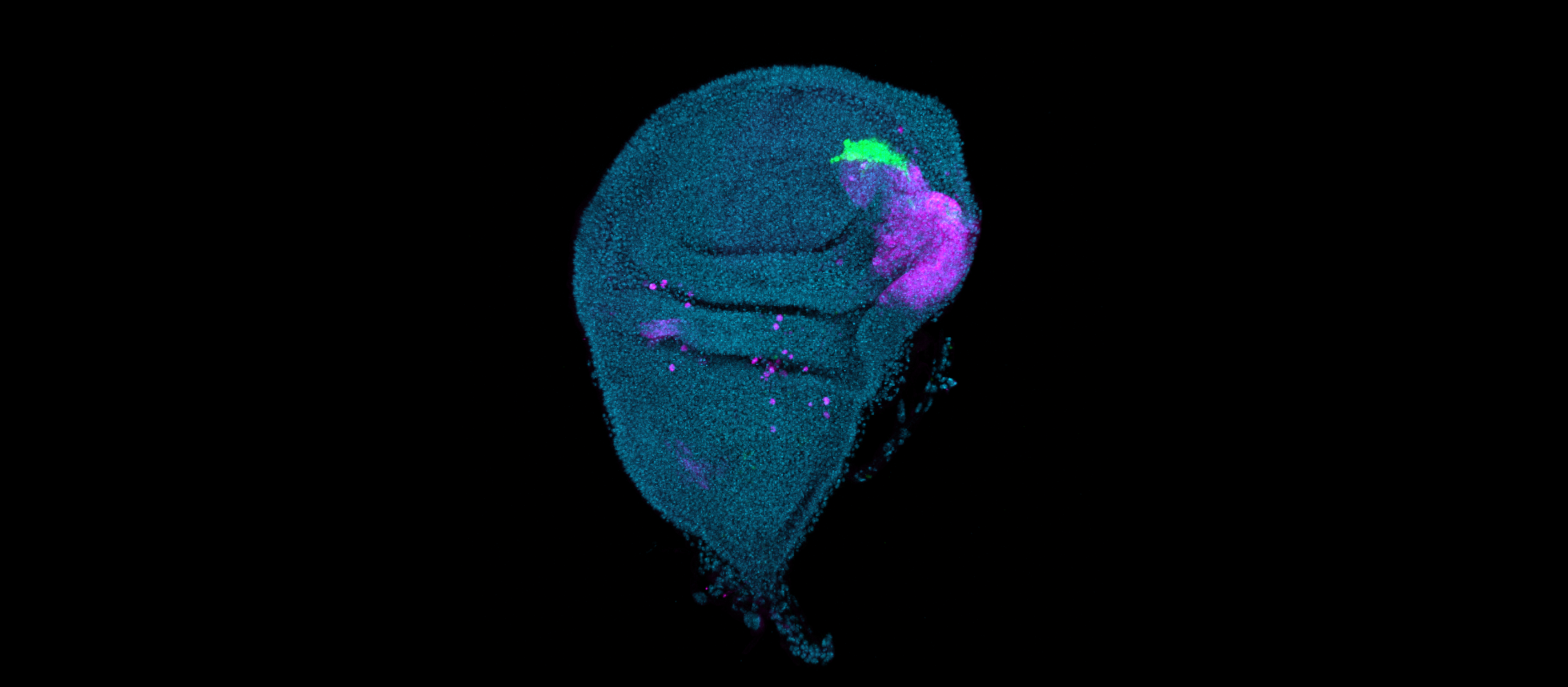

Supplement: Supplementary file 4 — Source data Fig. 2 [file 44319_2026_778_MOESM4_ESM.zip › Figure 2/2M/Fig.2M.tif]

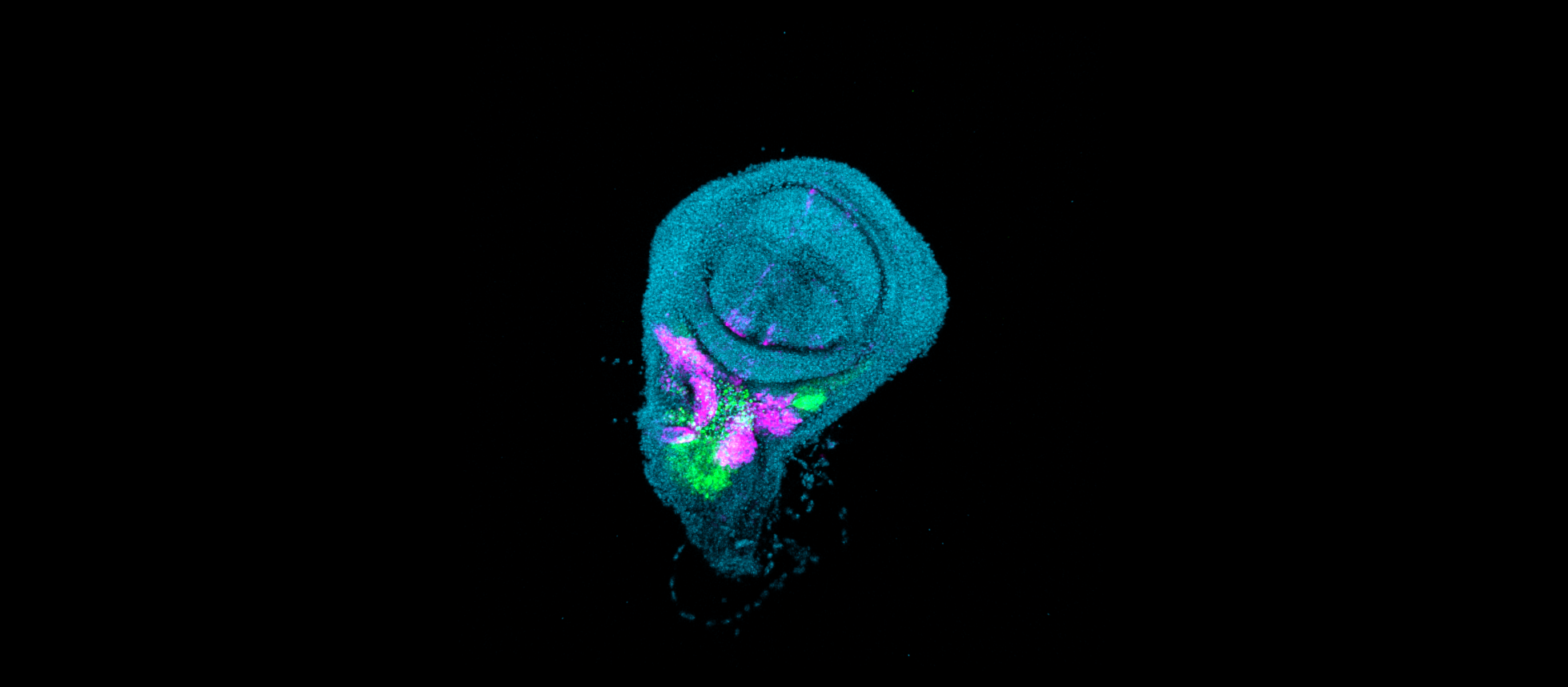

Supplement: Supplementary file 4 — Source data Fig. 2 [file 44319_2026_778_MOESM4_ESM.zip › Figure 2/2J/Fig.2J.tif]

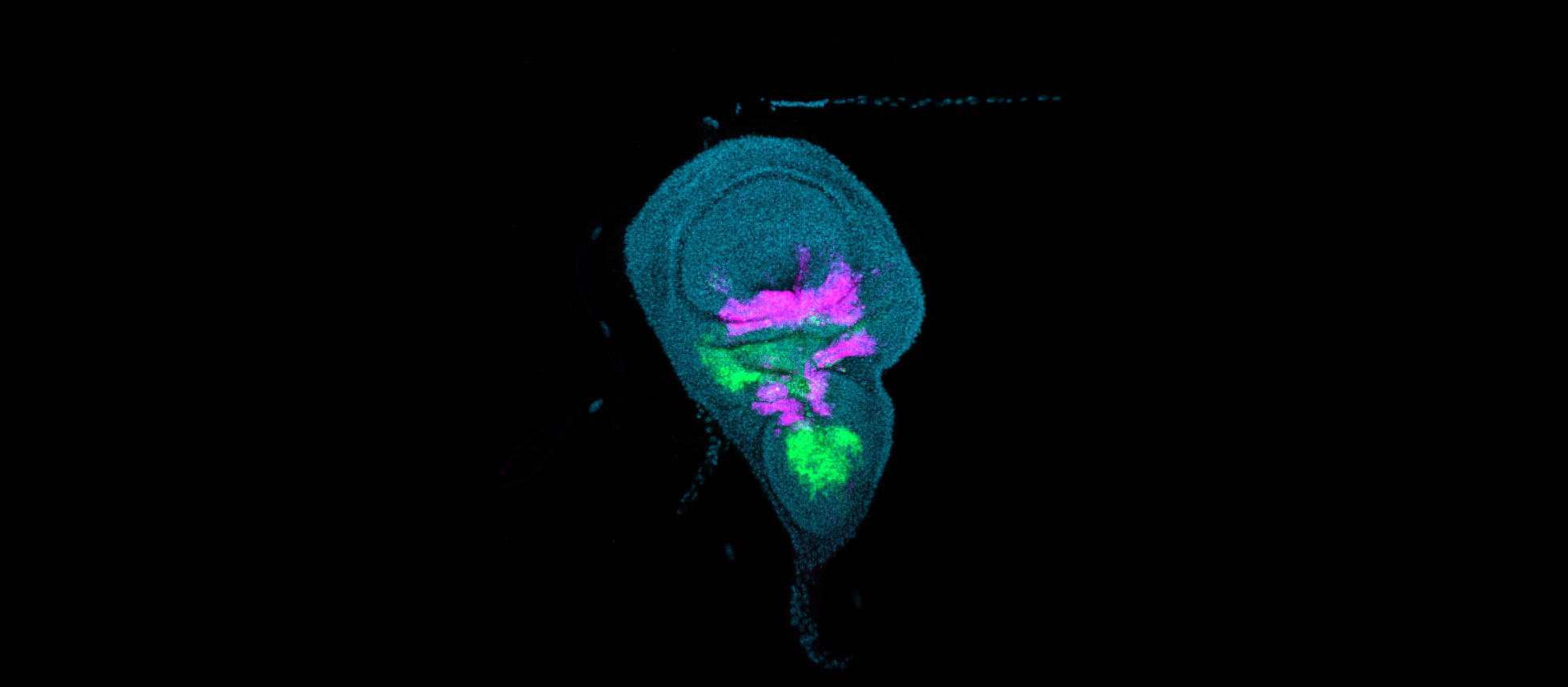

Supplement: Supplementary file 4 — Source data Fig. 2 [file 44319_2026_778_MOESM4_ESM.zip › Figure 2/2K/Fig.2K.tif]

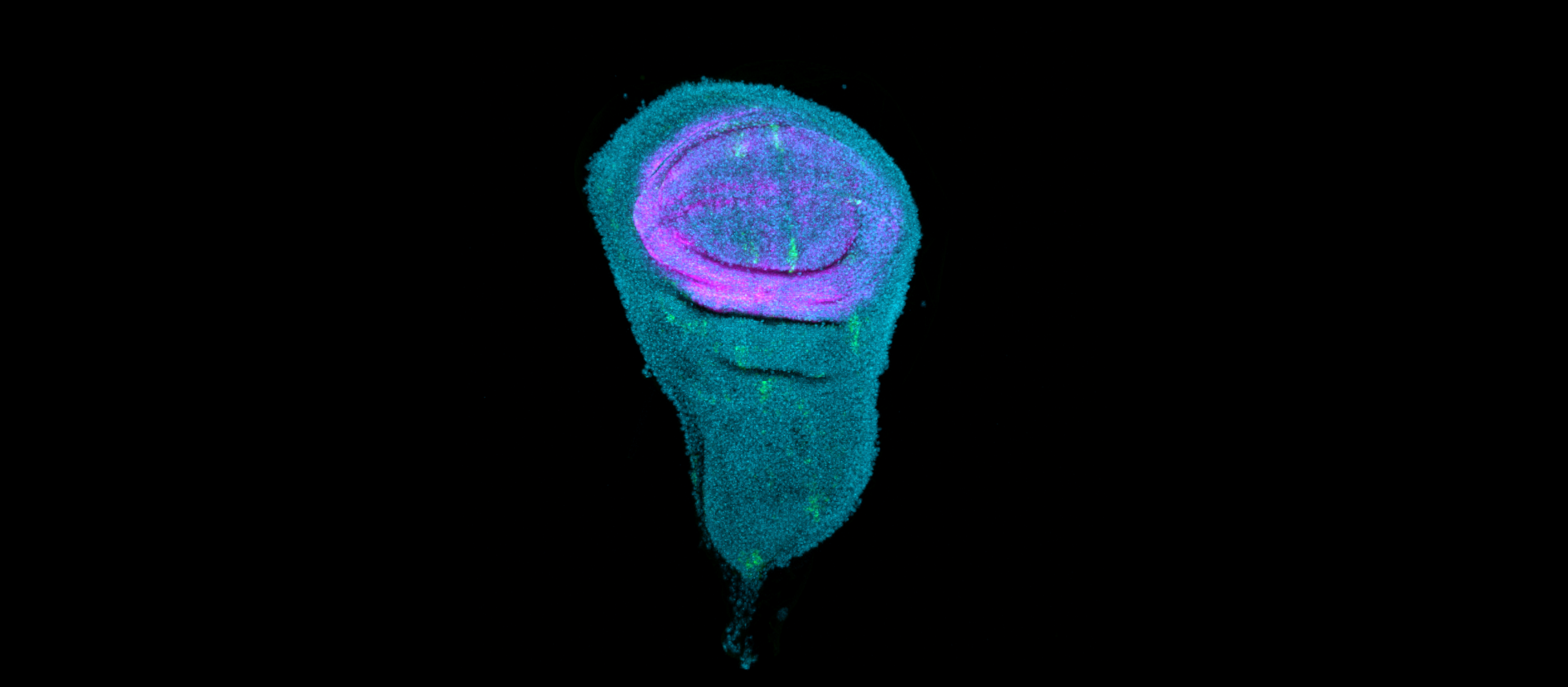

Supplement: Supplementary file 4 — Source data Fig. 2 [file 44319_2026_778_MOESM4_ESM.zip › Figure 2/2B/Fig.2B.tif]

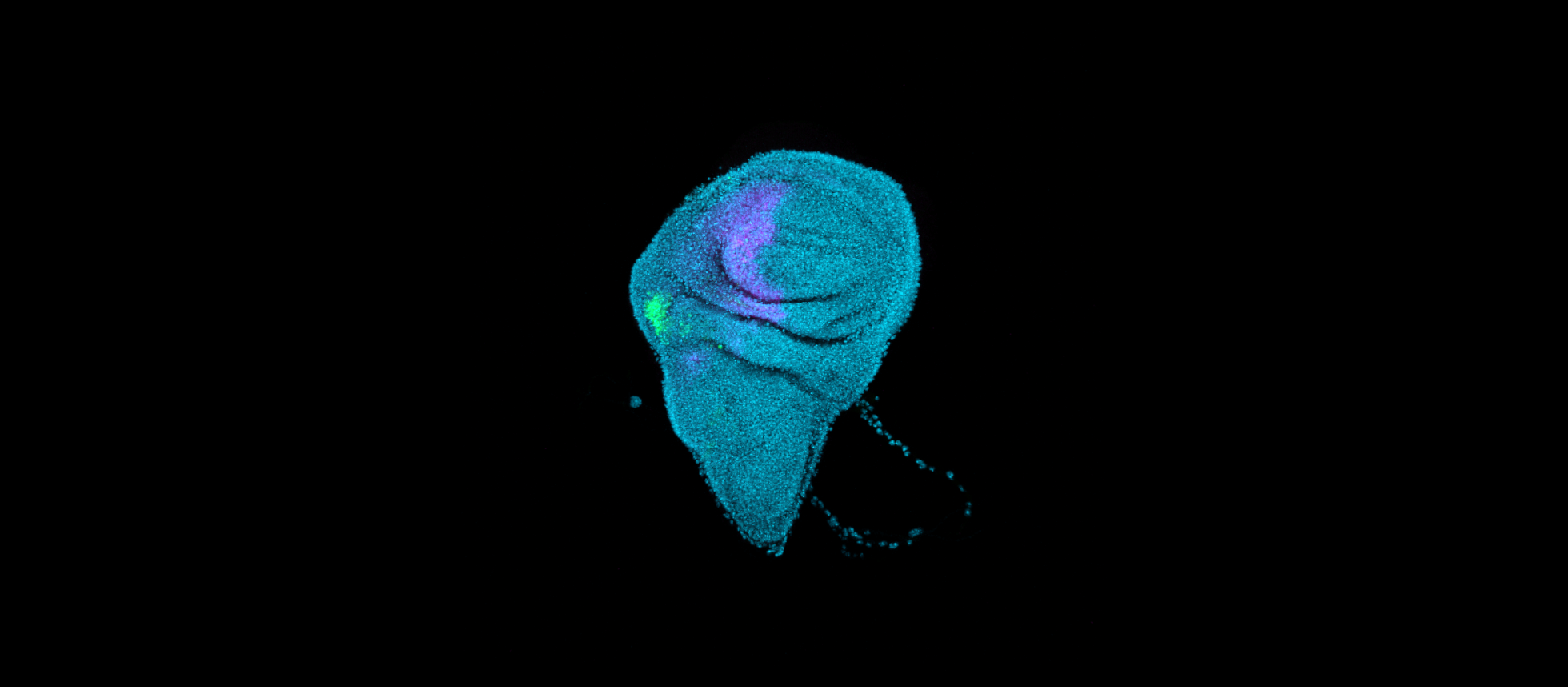

Supplement: Supplementary file 5 — Source data Fig. 3 [file 44319_2026_778_MOESM5_ESM.zip › Figure 3/3E/Fig.3E.tif]

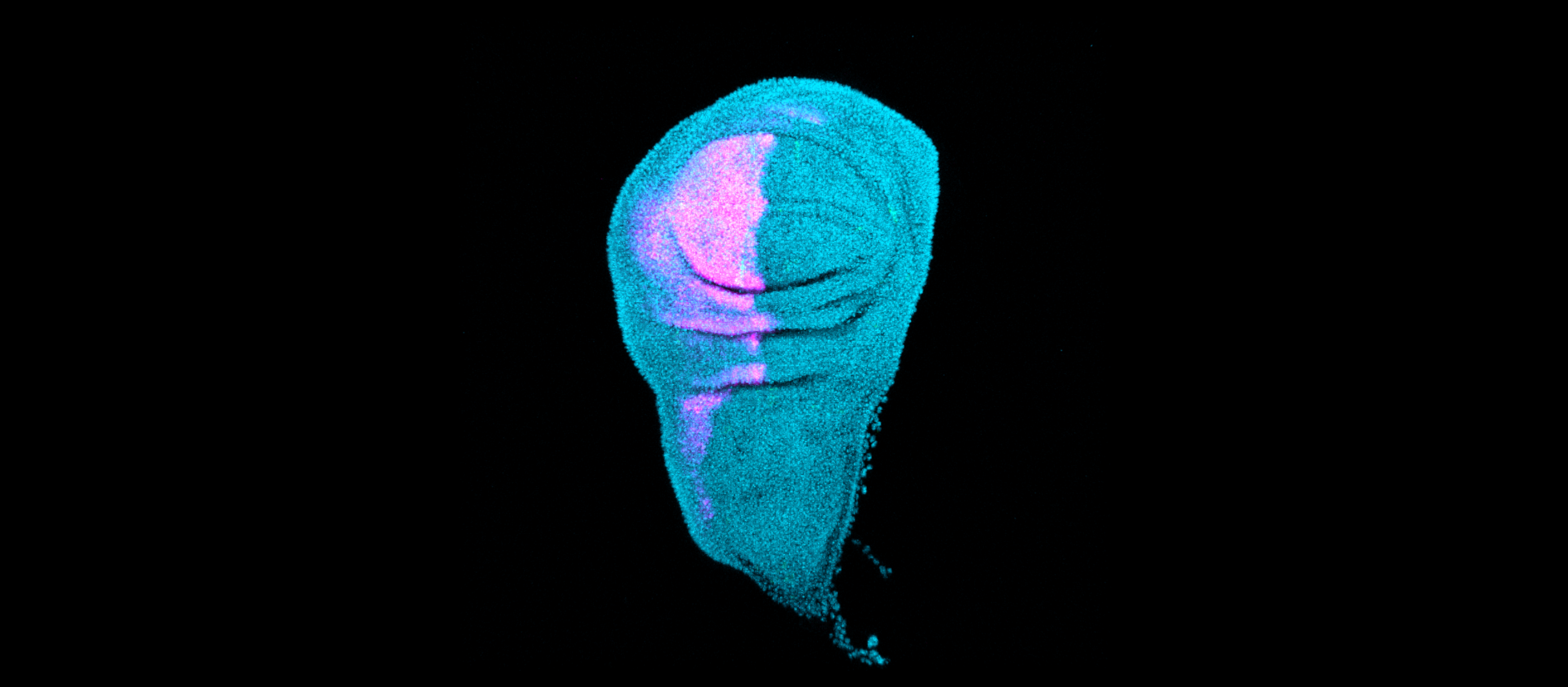

Supplement: Supplementary file 5 — Source data Fig. 3 [file 44319_2026_778_MOESM5_ESM.zip › Figure 3/3B/Fig.3B.tif]

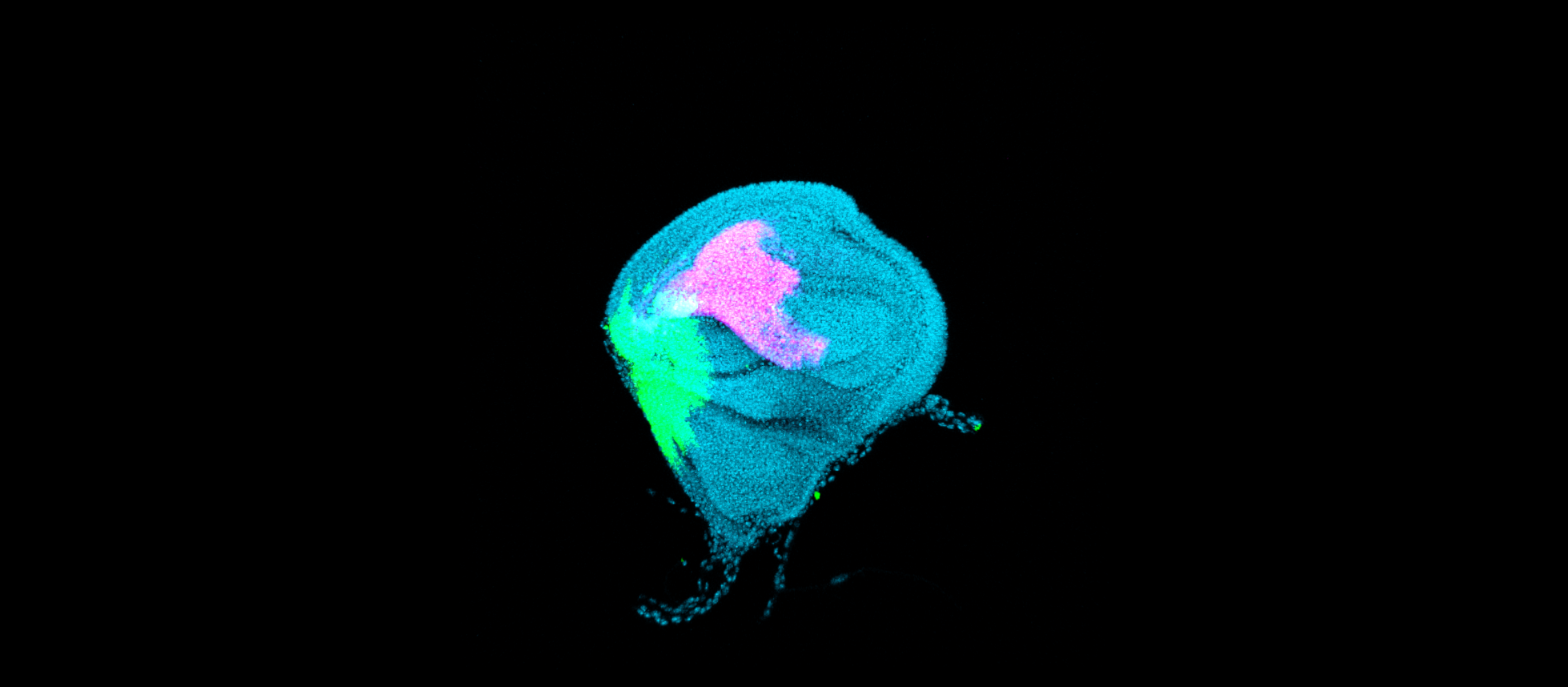

Supplement: Supplementary file 5 — Source data Fig. 3 [file 44319_2026_778_MOESM5_ESM.zip › Figure 3/3C/Fig.3C.tif]

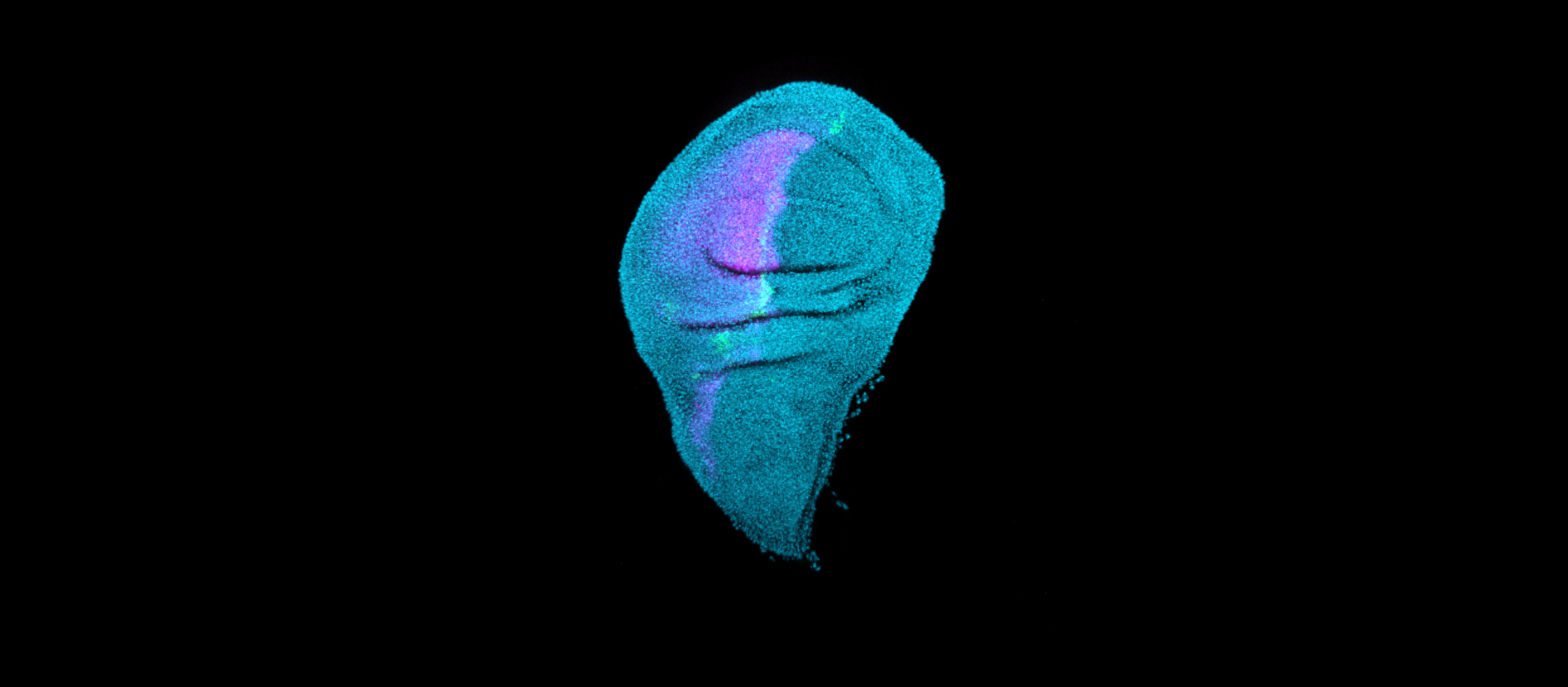

Supplement: Supplementary file 5 — Source data Fig. 3 [file 44319_2026_778_MOESM5_ESM.zip › Figure 3/3D/Fig.3D.tif]

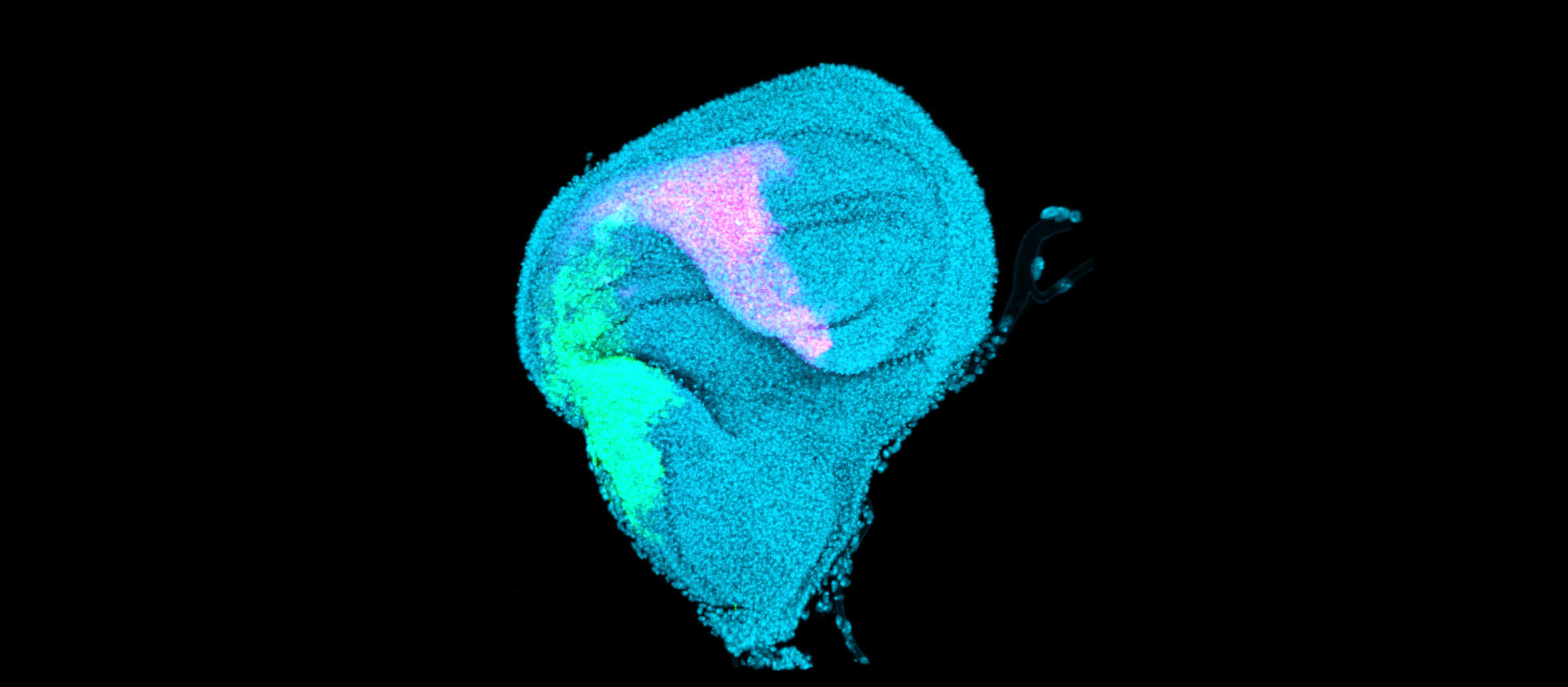

Supplement: Supplementary file 5 — Source data Fig. 3 [file 44319_2026_778_MOESM5_ESM.zip › Figure 3/3H/Fig.3H.tif]

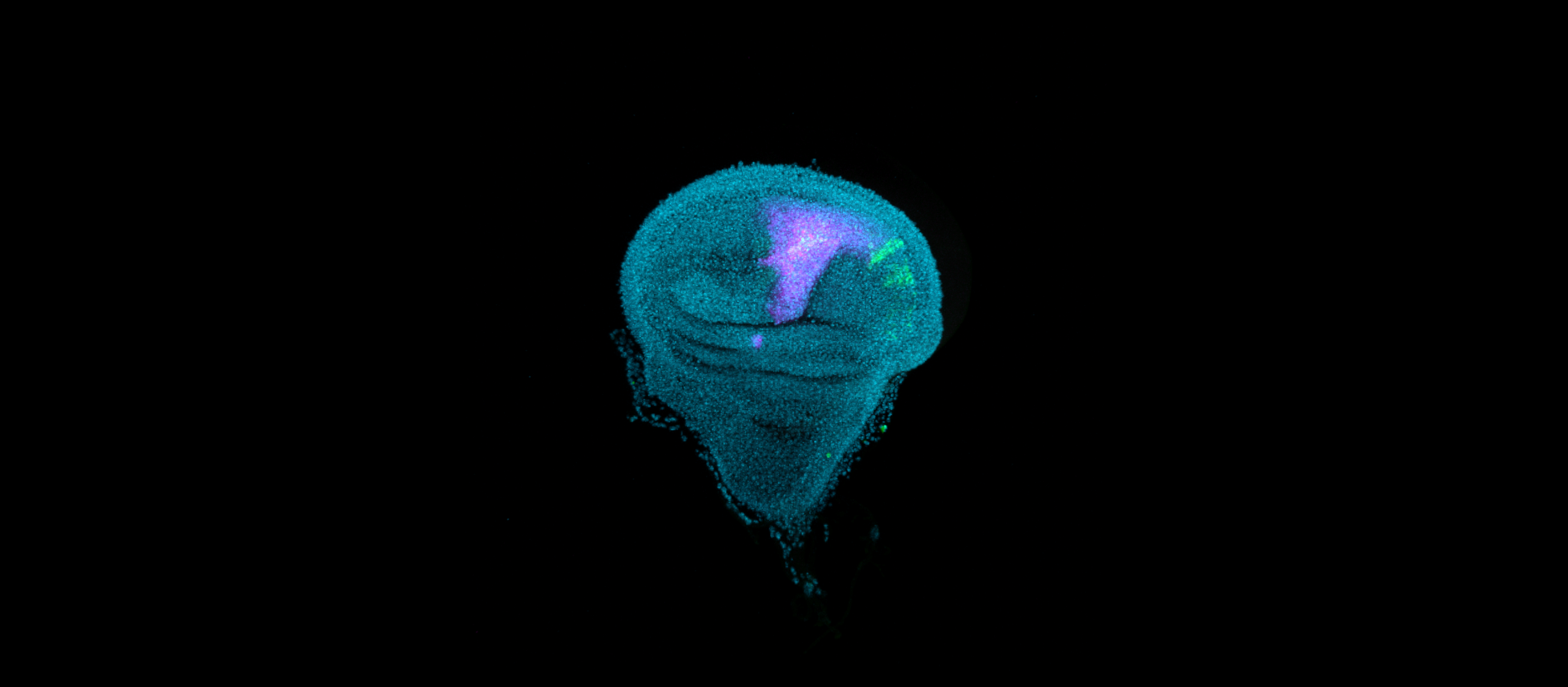

Supplement: Supplementary file 5 — Source data Fig. 3 [file 44319_2026_778_MOESM5_ESM.zip › Figure 3/3F/Fig.3F.tif]

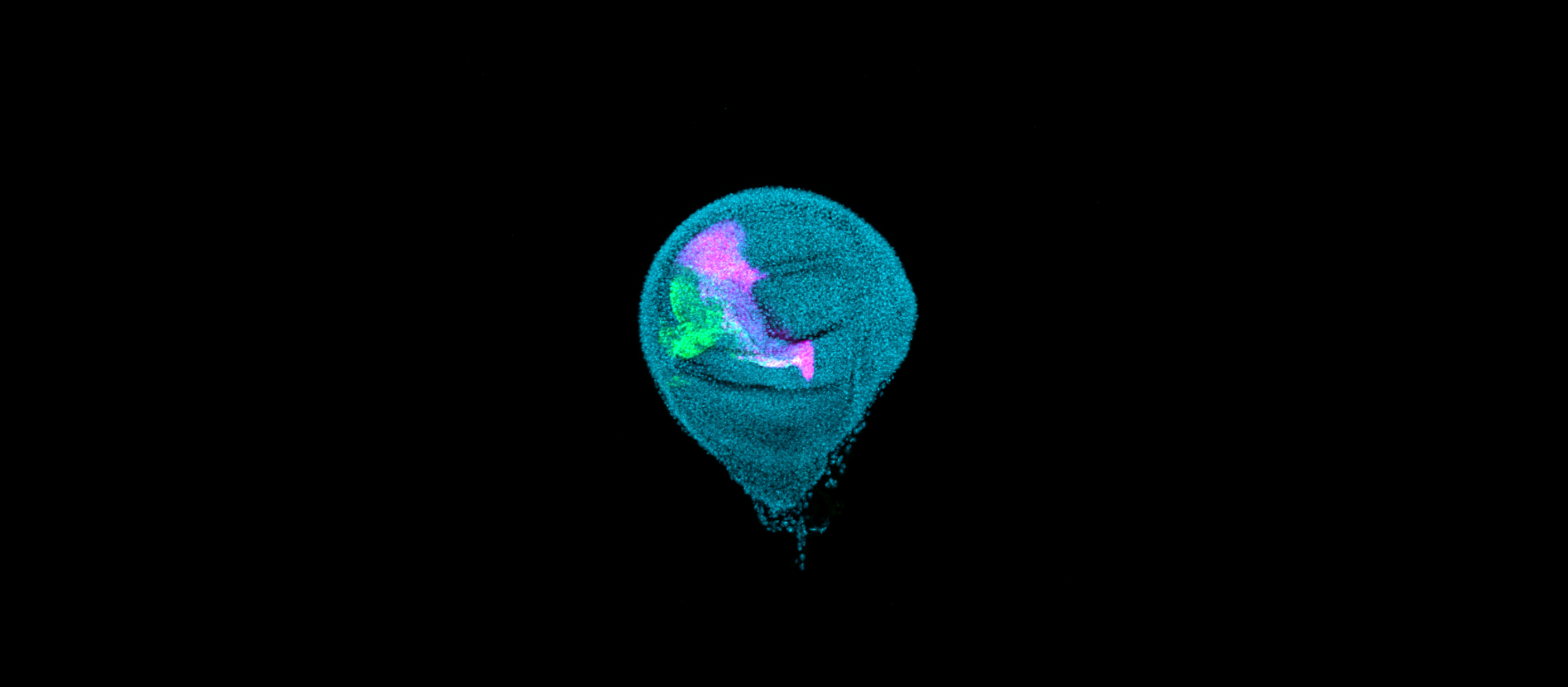

Supplement: Supplementary file 5 — Source data Fig. 3 [file 44319_2026_778_MOESM5_ESM.zip › Figure 3/3G/Fig.3G.tif]

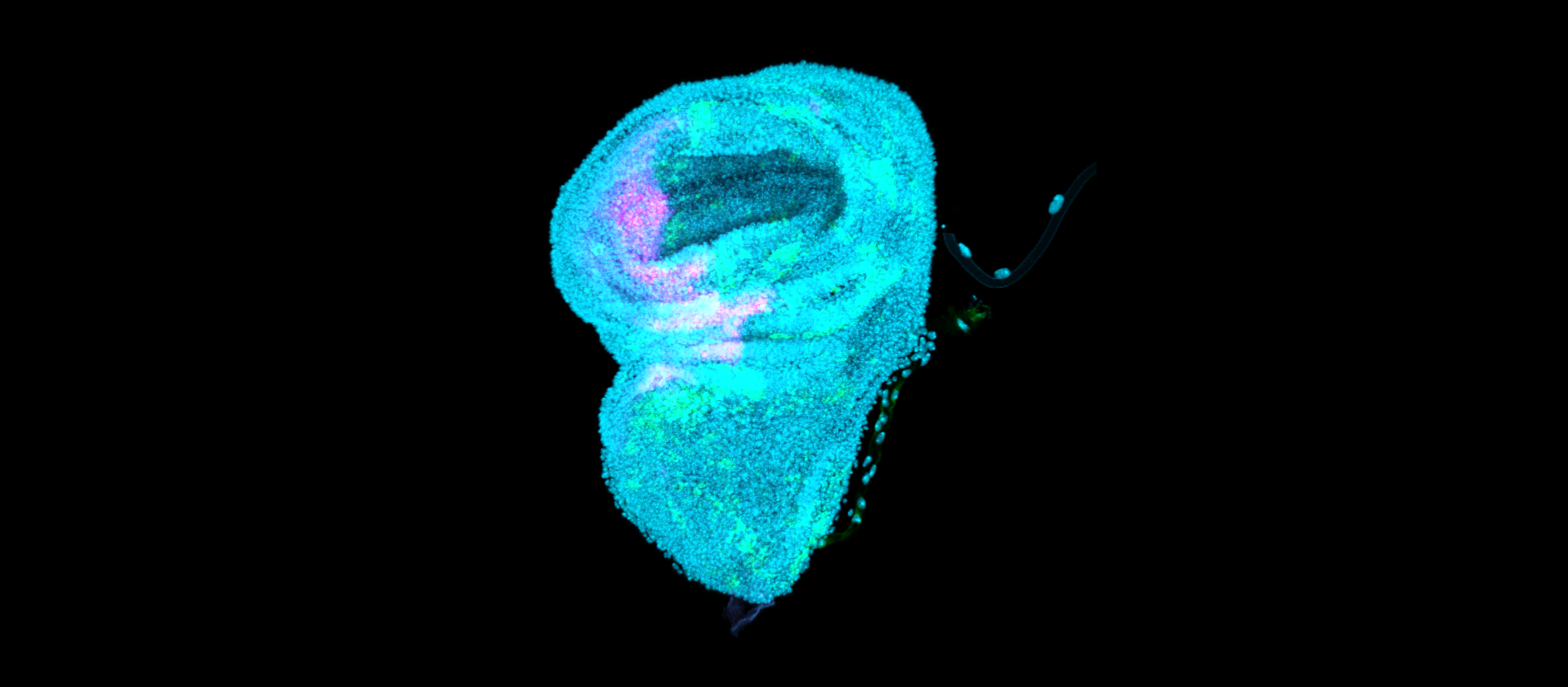

Supplement: Supplementary file 5 — Source data Fig. 3 [file 44319_2026_778_MOESM5_ESM.zip › Figure 3/3I/Fig.3I.tif]

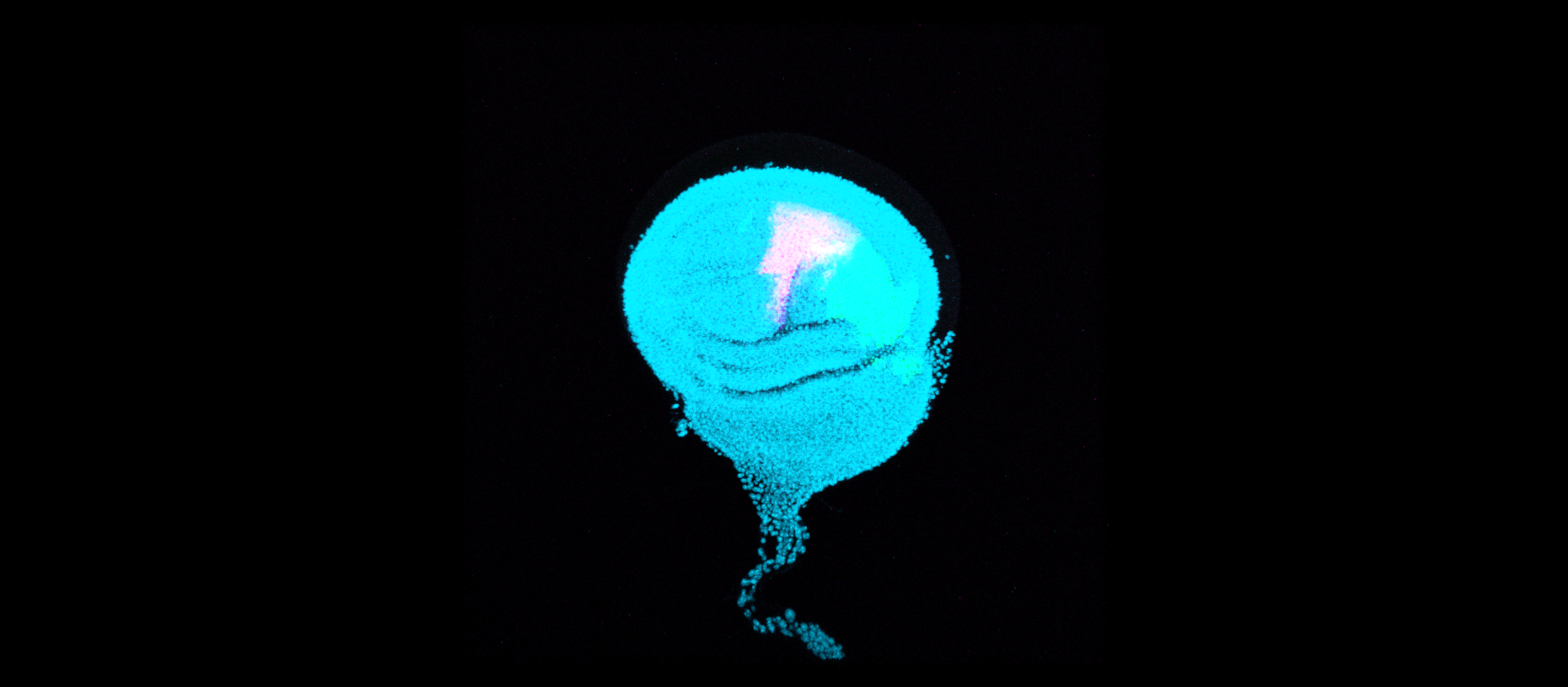

Supplement: Supplementary file 6 — Source data Fig. 4 [file 44319_2026_778_MOESM6_ESM.zip › Figure 4/4E/Fig.4E.tif]

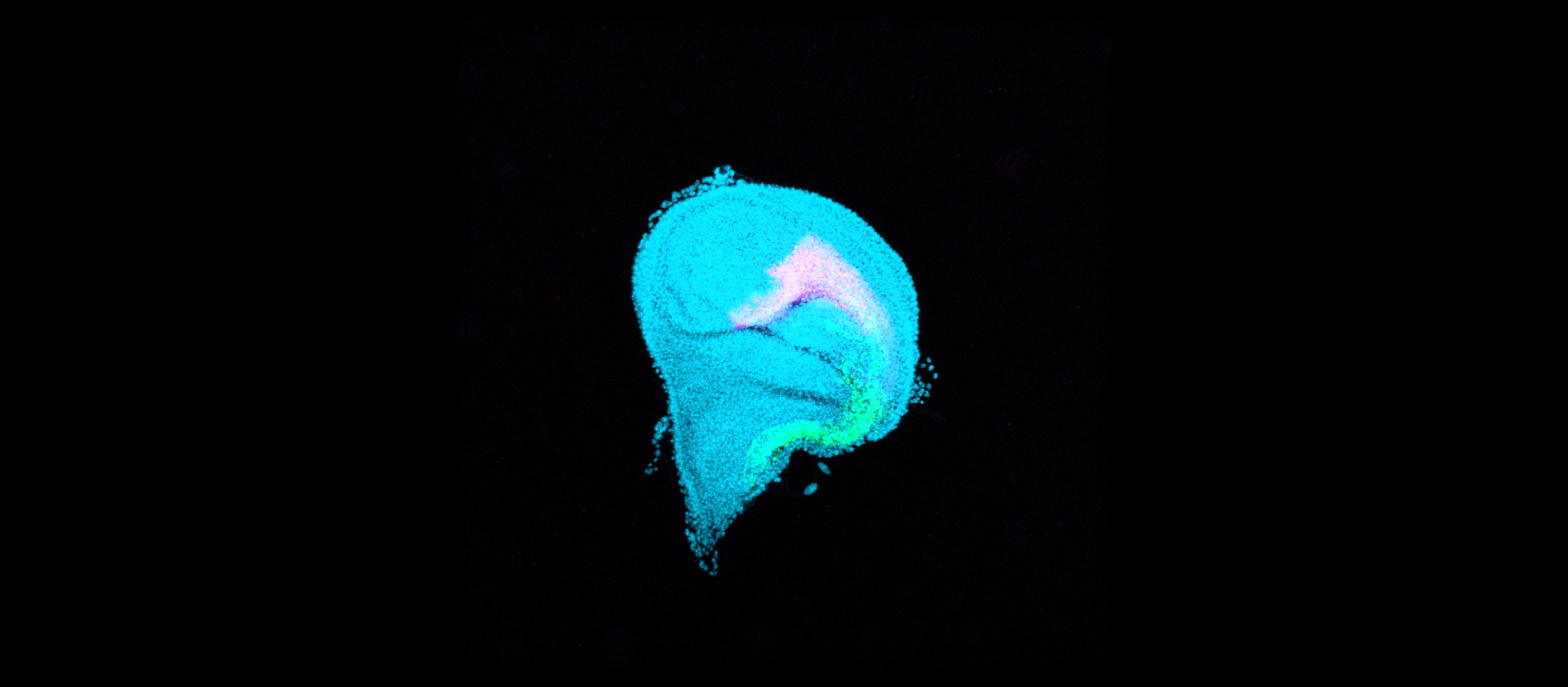

Supplement: Supplementary file 6 — Source data Fig. 4 [file 44319_2026_778_MOESM6_ESM.zip › Figure 4/4B/Fig.4B.tif]

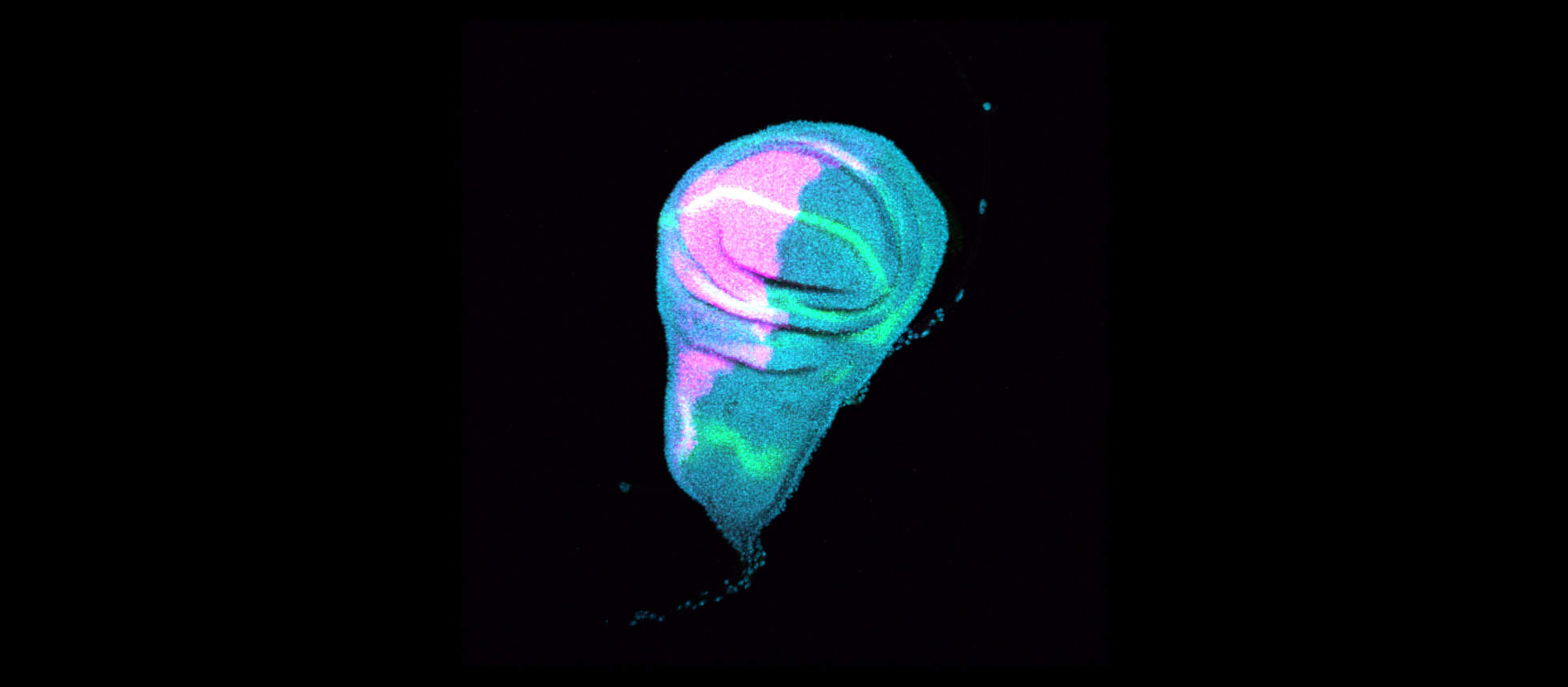

Supplement: Supplementary file 6 — Source data Fig. 4 [file 44319_2026_778_MOESM6_ESM.zip › Figure 4/4L/Fig.4L.tif]

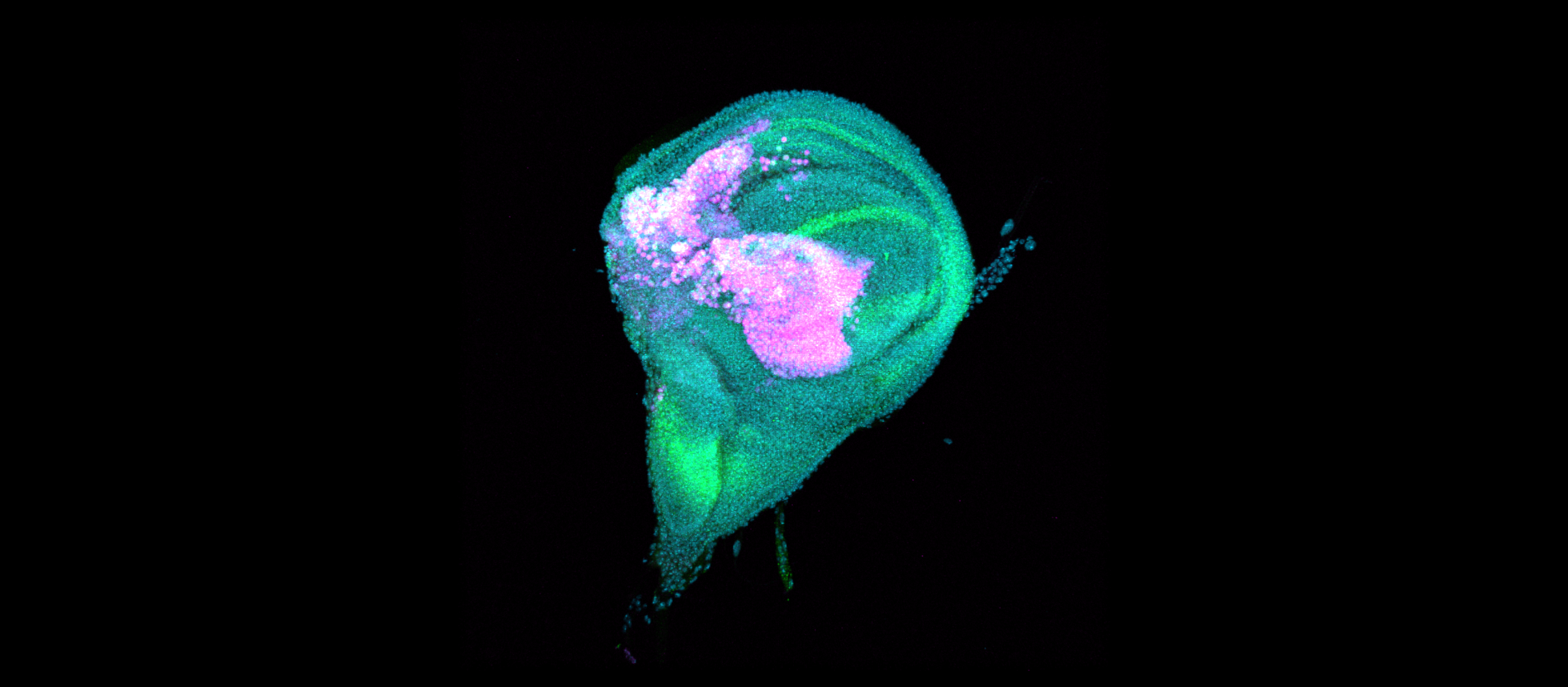

Supplement: Supplementary file 6 — Source data Fig. 4 [file 44319_2026_778_MOESM6_ESM.zip › Figure 4/4M/Fig.4M.tif]

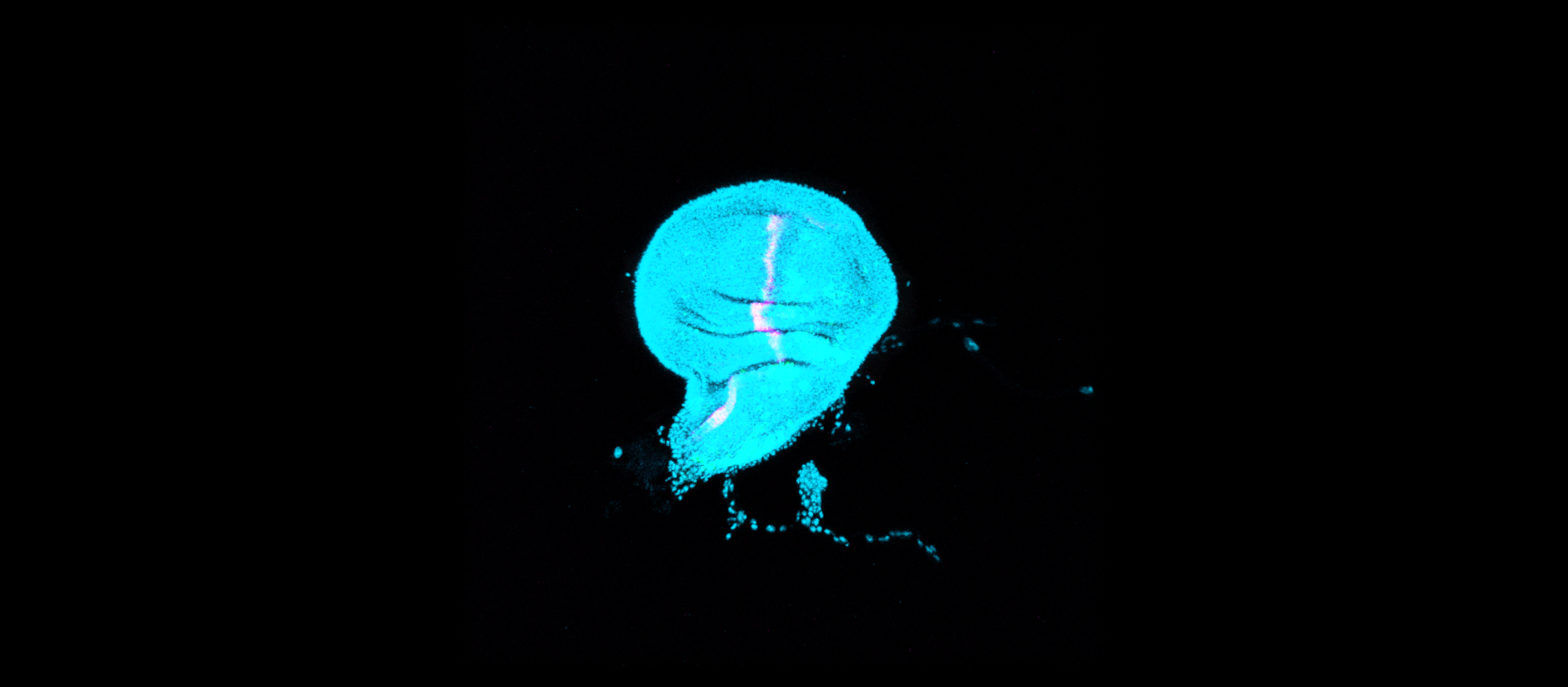

Supplement: Supplementary file 6 — Source data Fig. 4 [file 44319_2026_778_MOESM6_ESM.zip › Figure 4/4J/Fig.4J.tif]

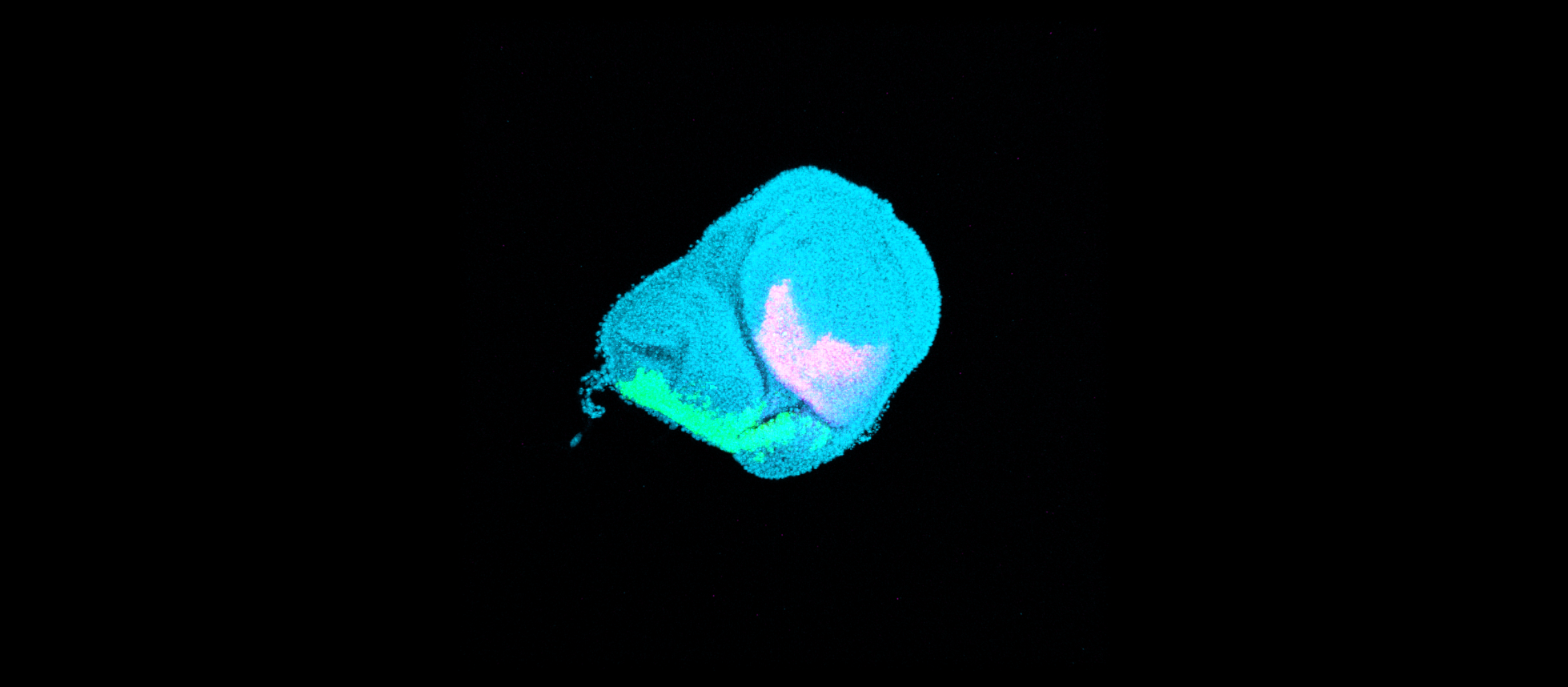

Supplement: Supplementary file 6 — Source data Fig. 4 [file 44319_2026_778_MOESM6_ESM.zip › Figure 4/4C/Fig.4C.tif]

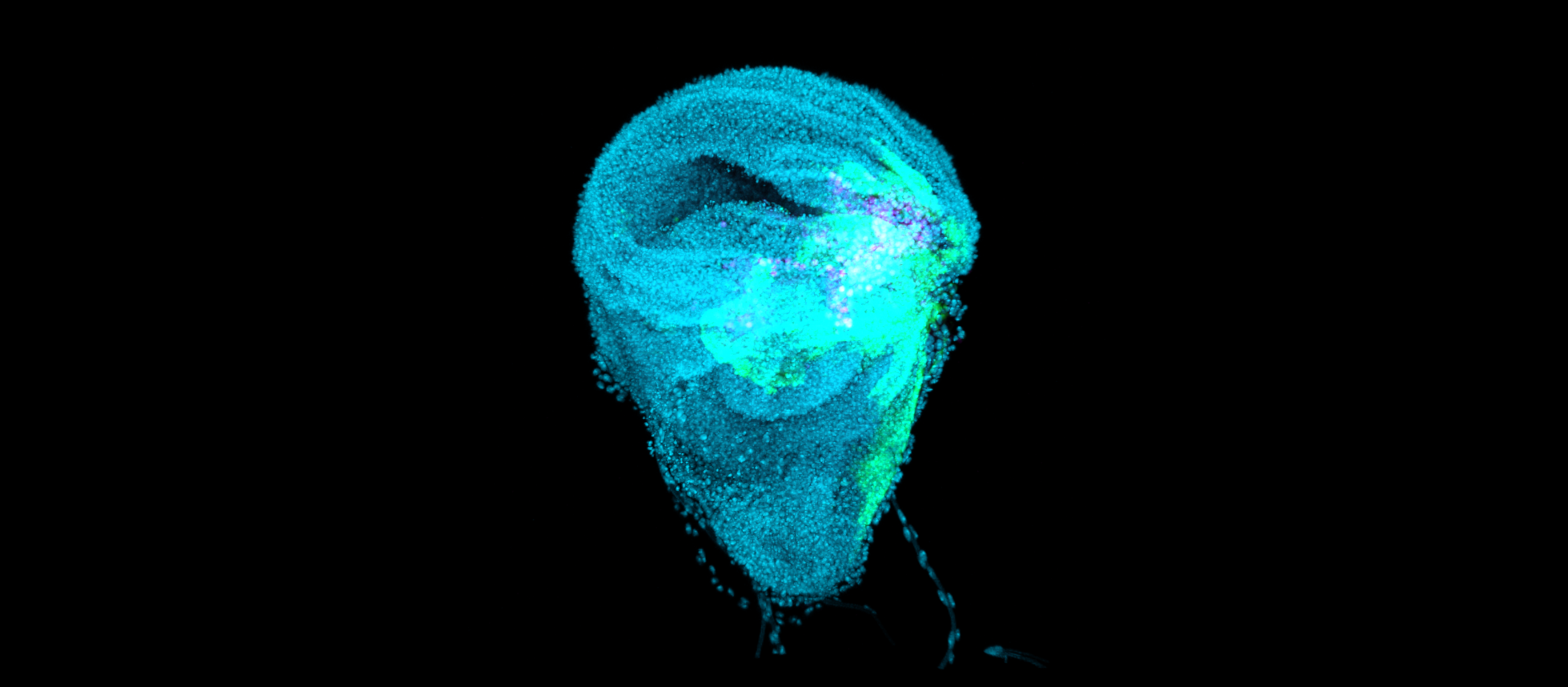

Supplement: Supplementary file 6 — Source data Fig. 4 [file 44319_2026_778_MOESM6_ESM.zip › Figure 4/4D/Fig.4D.tif]

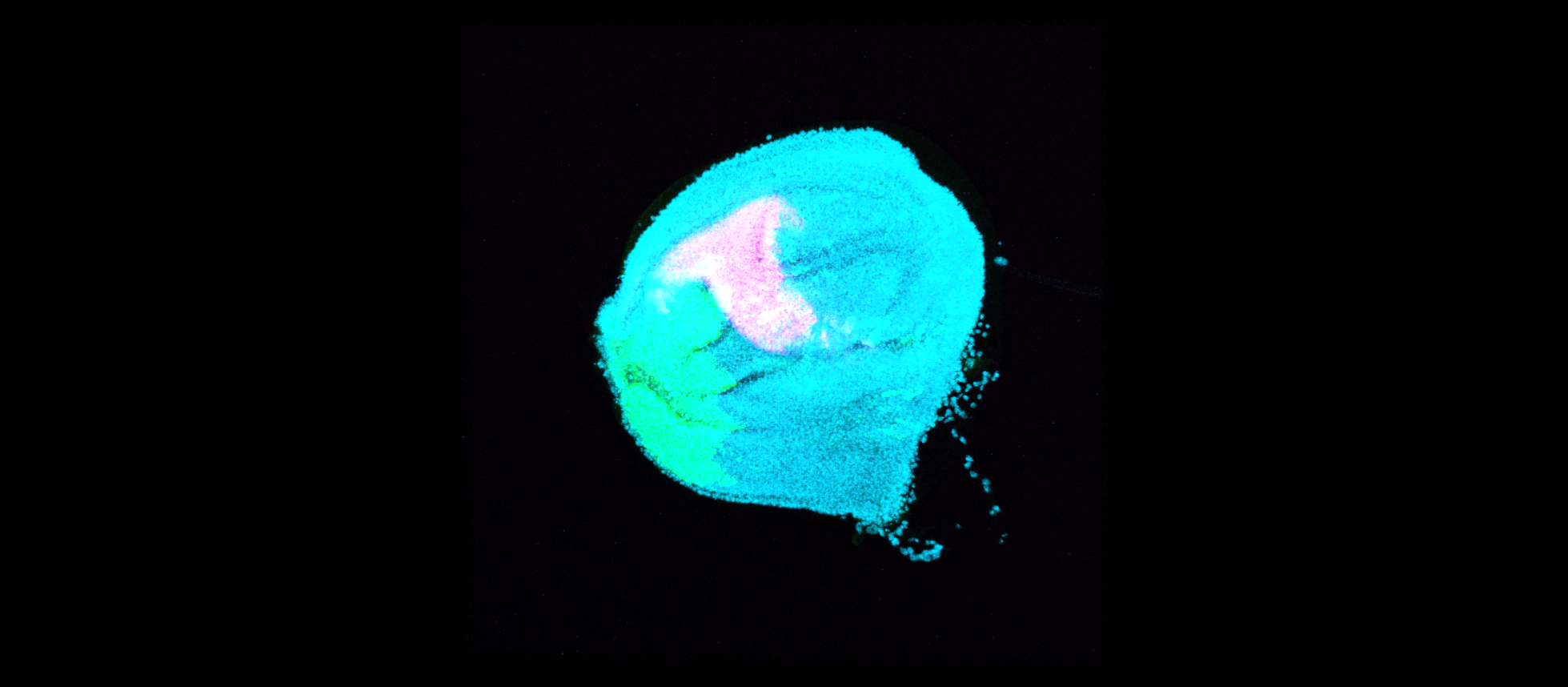

Supplement: Supplementary file 6 — Source data Fig. 4 [file 44319_2026_778_MOESM6_ESM.zip › Figure 4/4A/Fig.4A.tif]

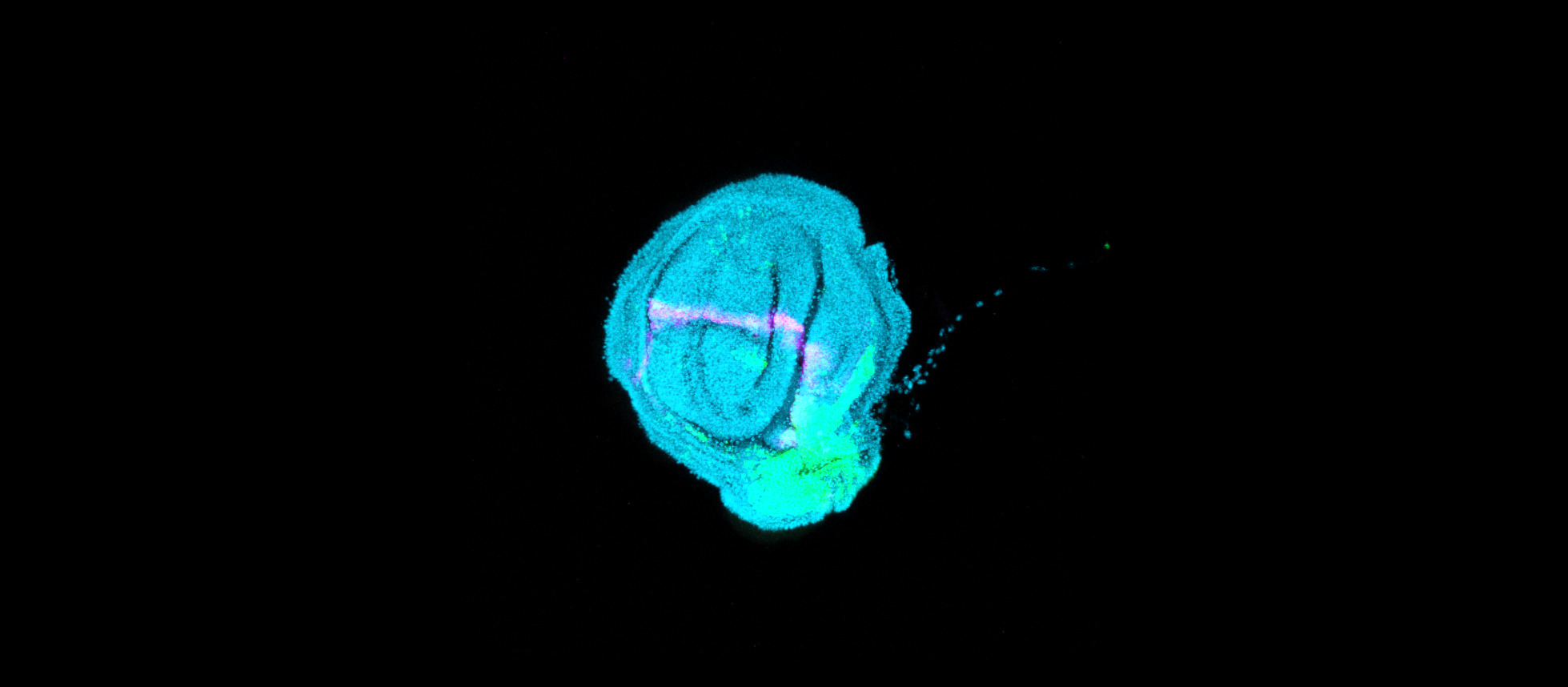

Supplement: Supplementary file 6 — Source data Fig. 4 [file 44319_2026_778_MOESM6_ESM.zip › Figure 4/4H/Fig.4H.tif]

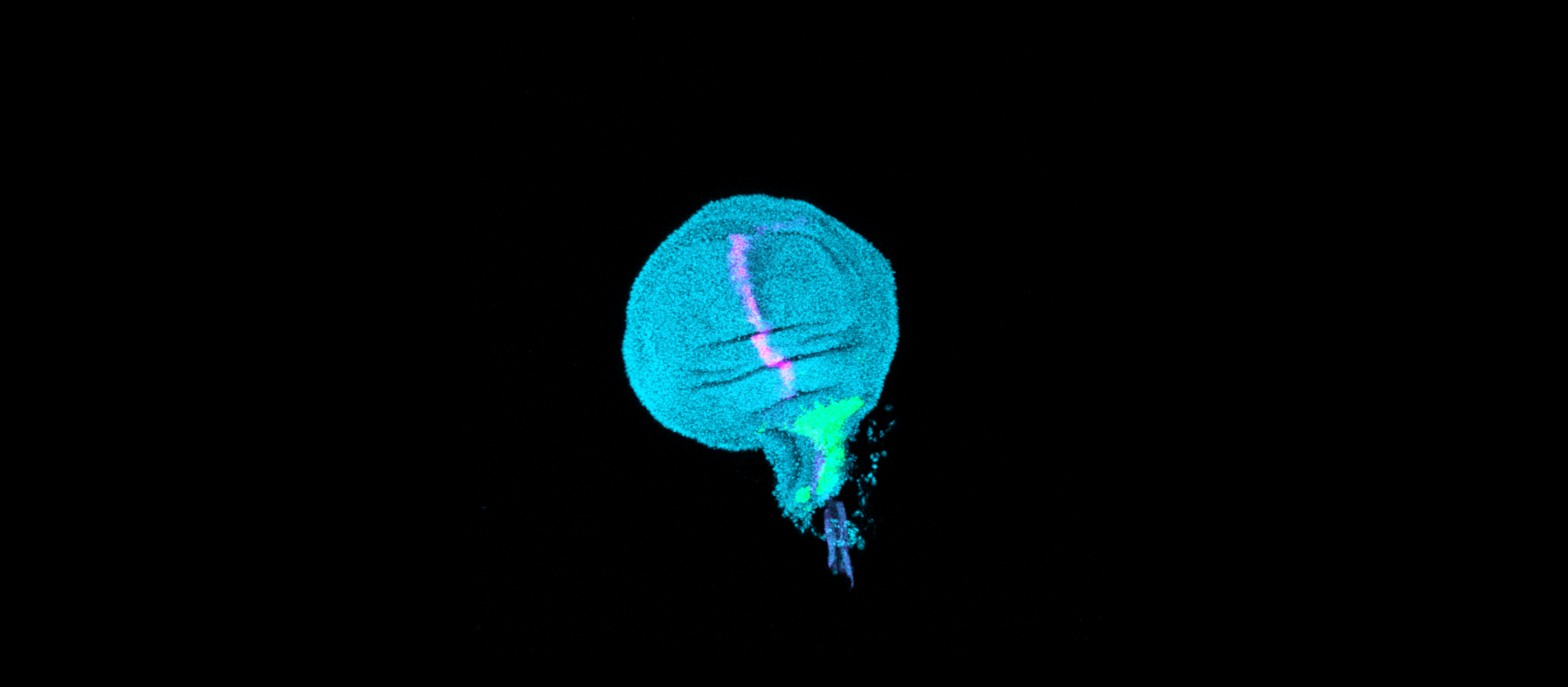

Supplement: Supplementary file 6 — Source data Fig. 4 [file 44319_2026_778_MOESM6_ESM.zip › Figure 4/4I/Fig.4I.tif]

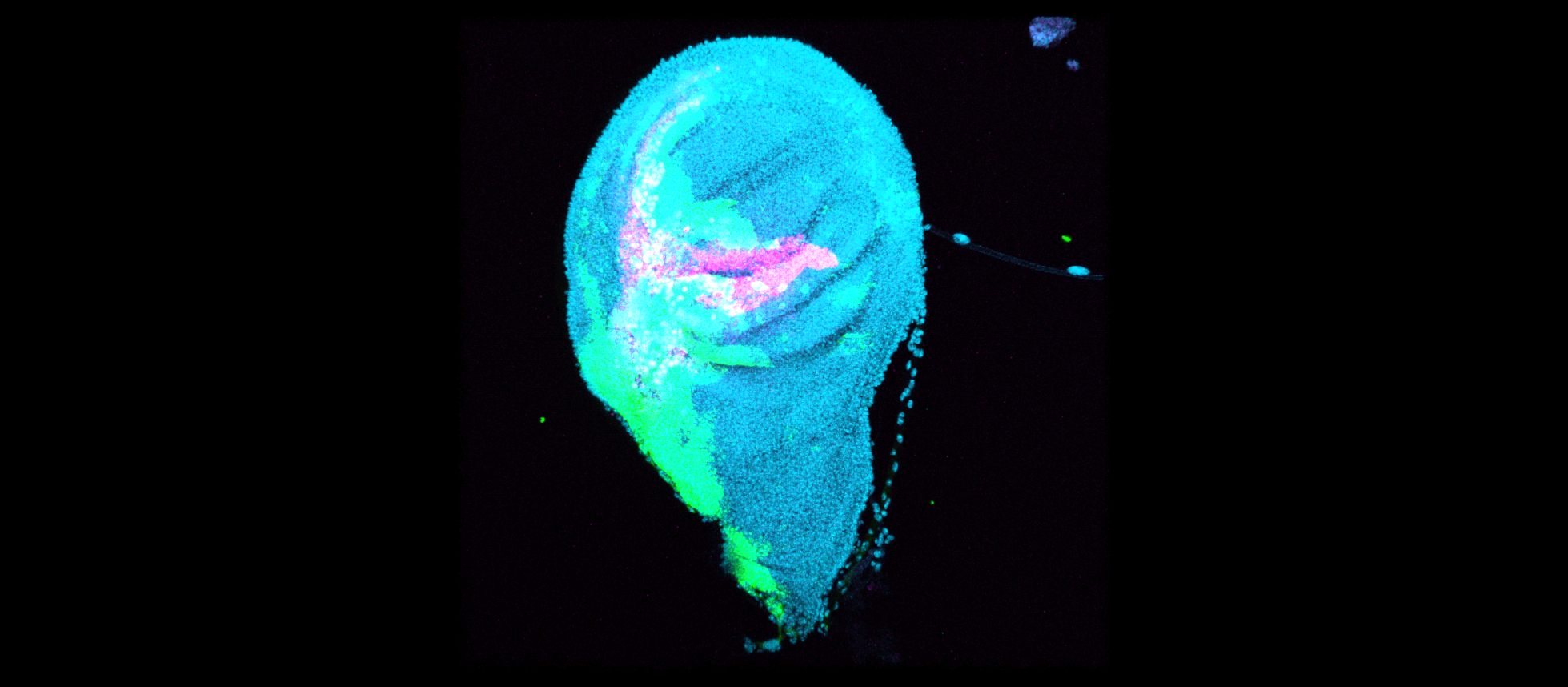

Supplement: Supplementary file 8 — Source data Fig. 6 [file 44319_2026_778_MOESM8_ESM.zip › Figure 6/6F/Fig.6F.tif]

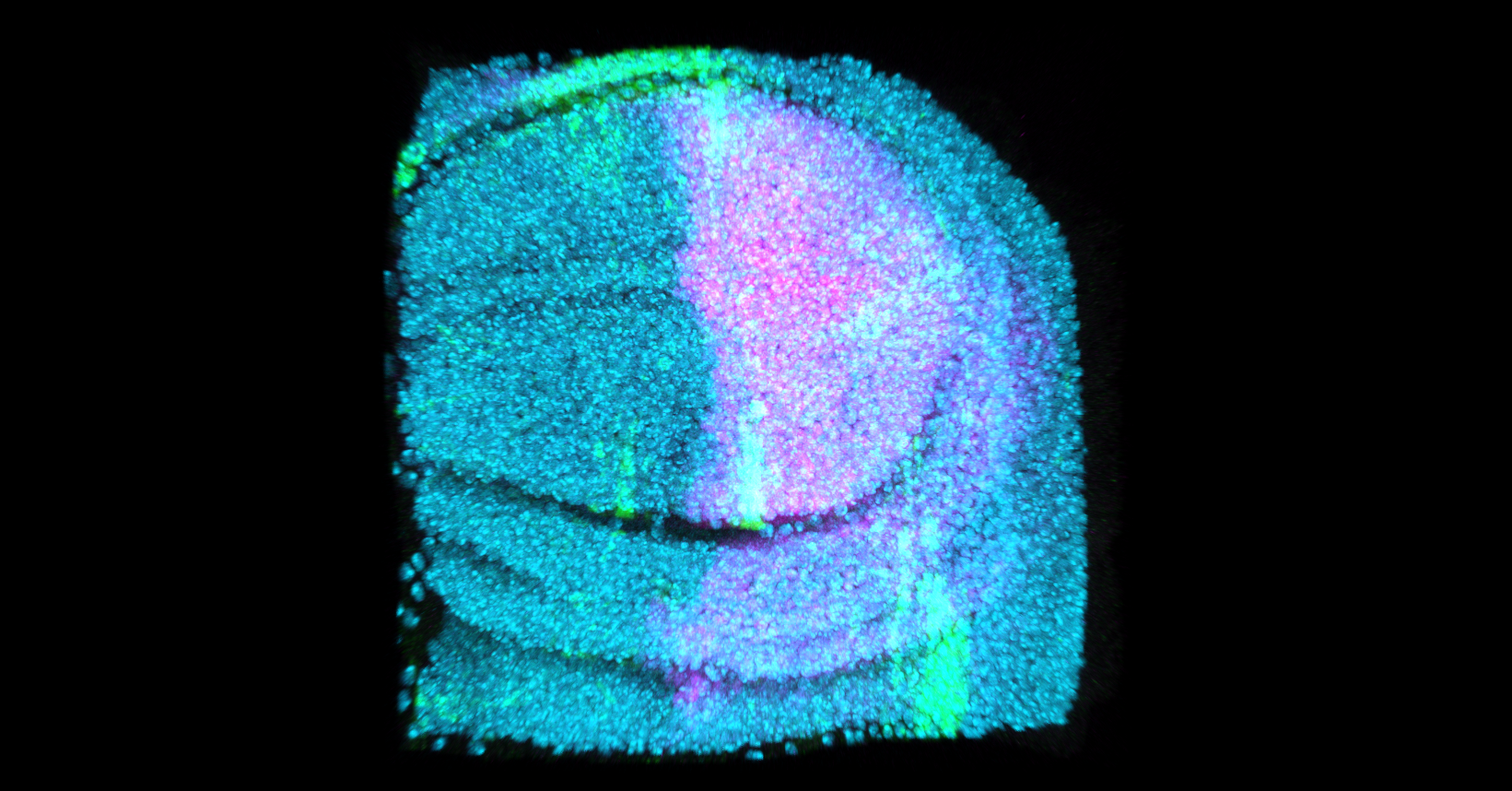

Supplement: Supplementary file 8 — Source data Fig. 6 [file 44319_2026_778_MOESM8_ESM.zip › Figure 6/6H/Fig6.H.tif]

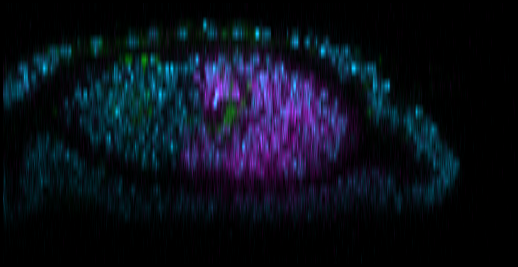

Supplement: Supplementary file 8 — Source data Fig. 6 [file 44319_2026_778_MOESM8_ESM.zip › Figure 6/6H/Fig.6H vertical section.tif]

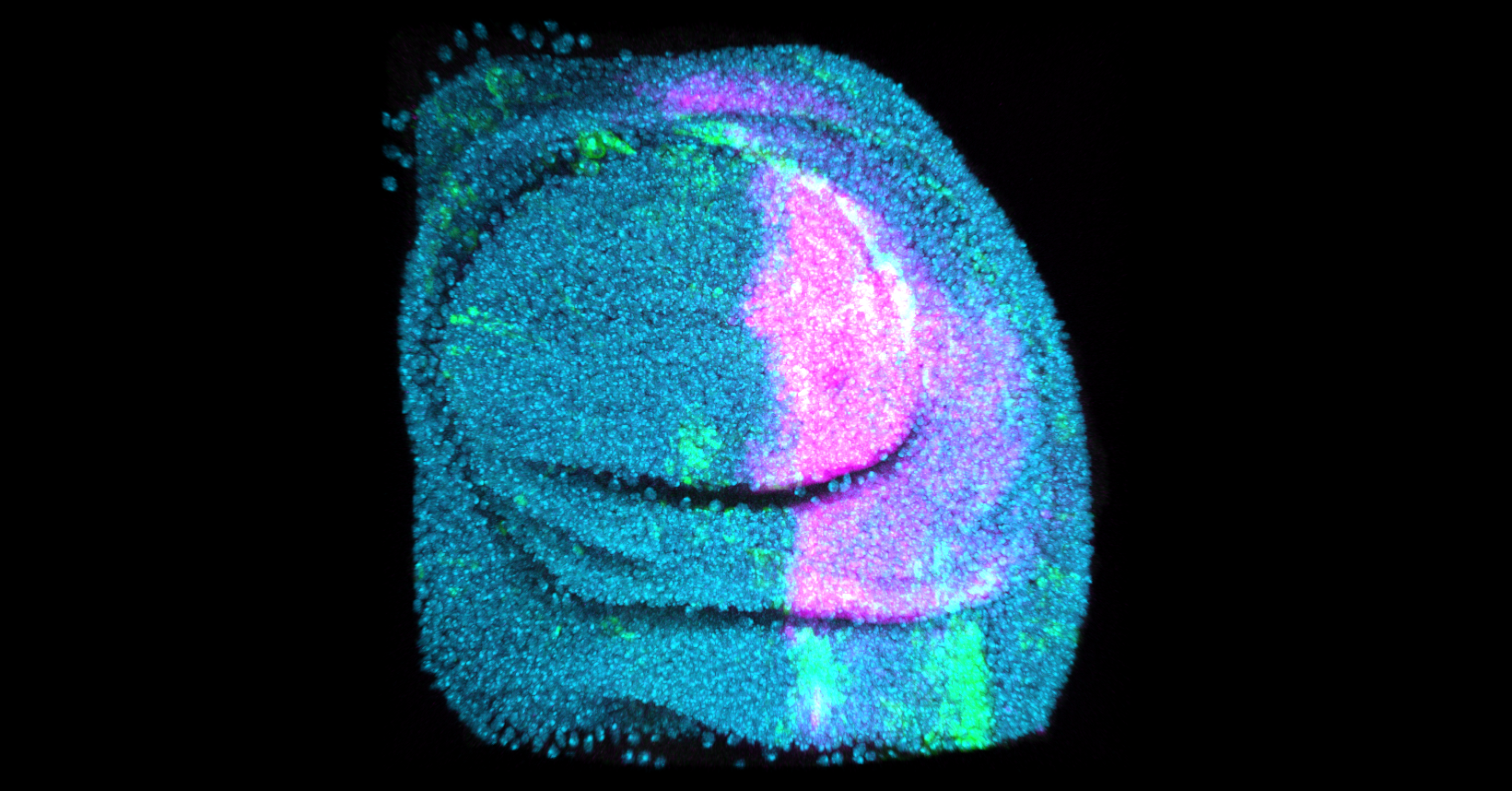

Supplement: Supplementary file 8 — Source data Fig. 6 [file 44319_2026_778_MOESM8_ESM.zip › Figure 6/6I/Fig.6I.tif]

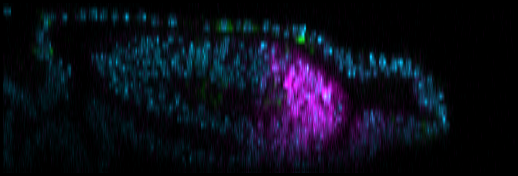

Supplement: Supplementary file 8 — Source data Fig. 6 [file 44319_2026_778_MOESM8_ESM.zip › Figure 6/6I/Fig.6I vertical section.tif]

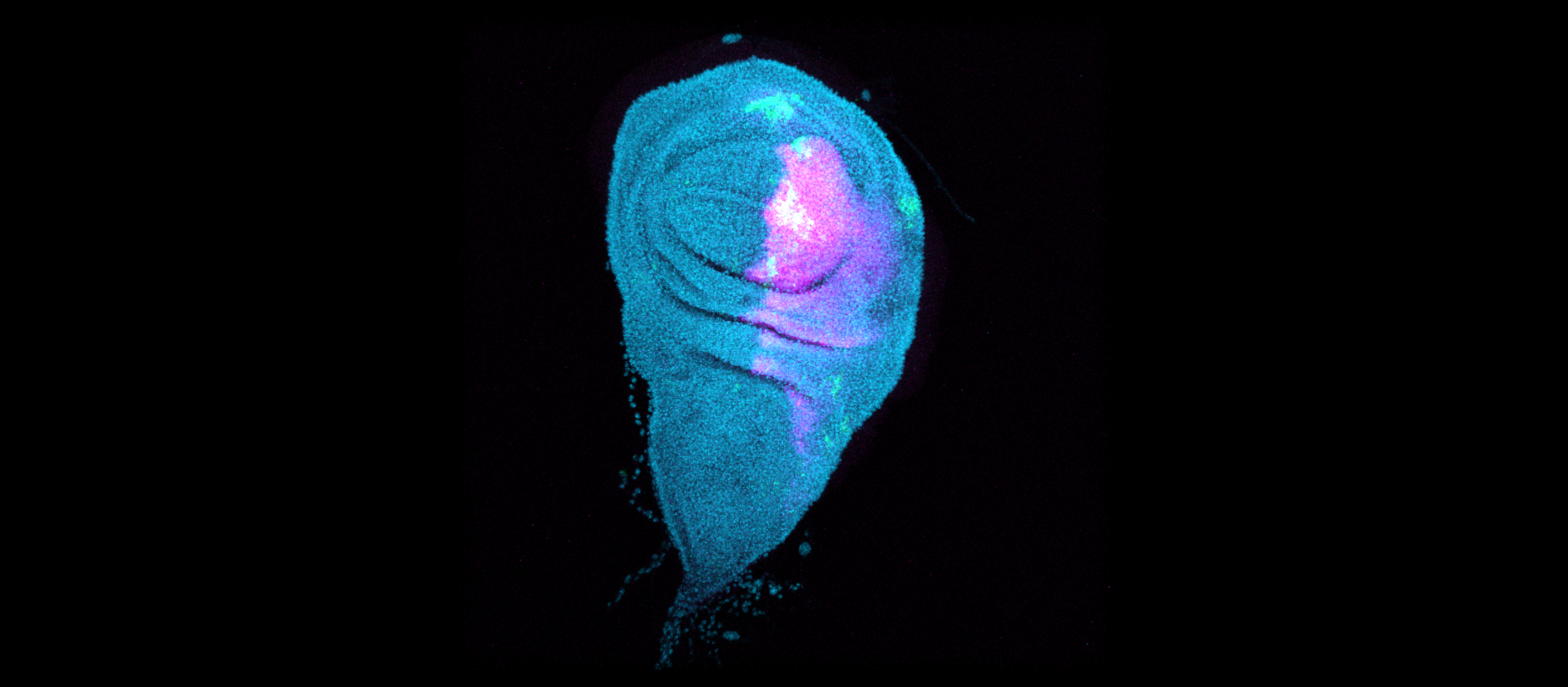

Supplement: Supplementary file 8 — Source data Fig. 6 [file 44319_2026_778_MOESM8_ESM.zip › Figure 6/6E/Fig.6E.tif]

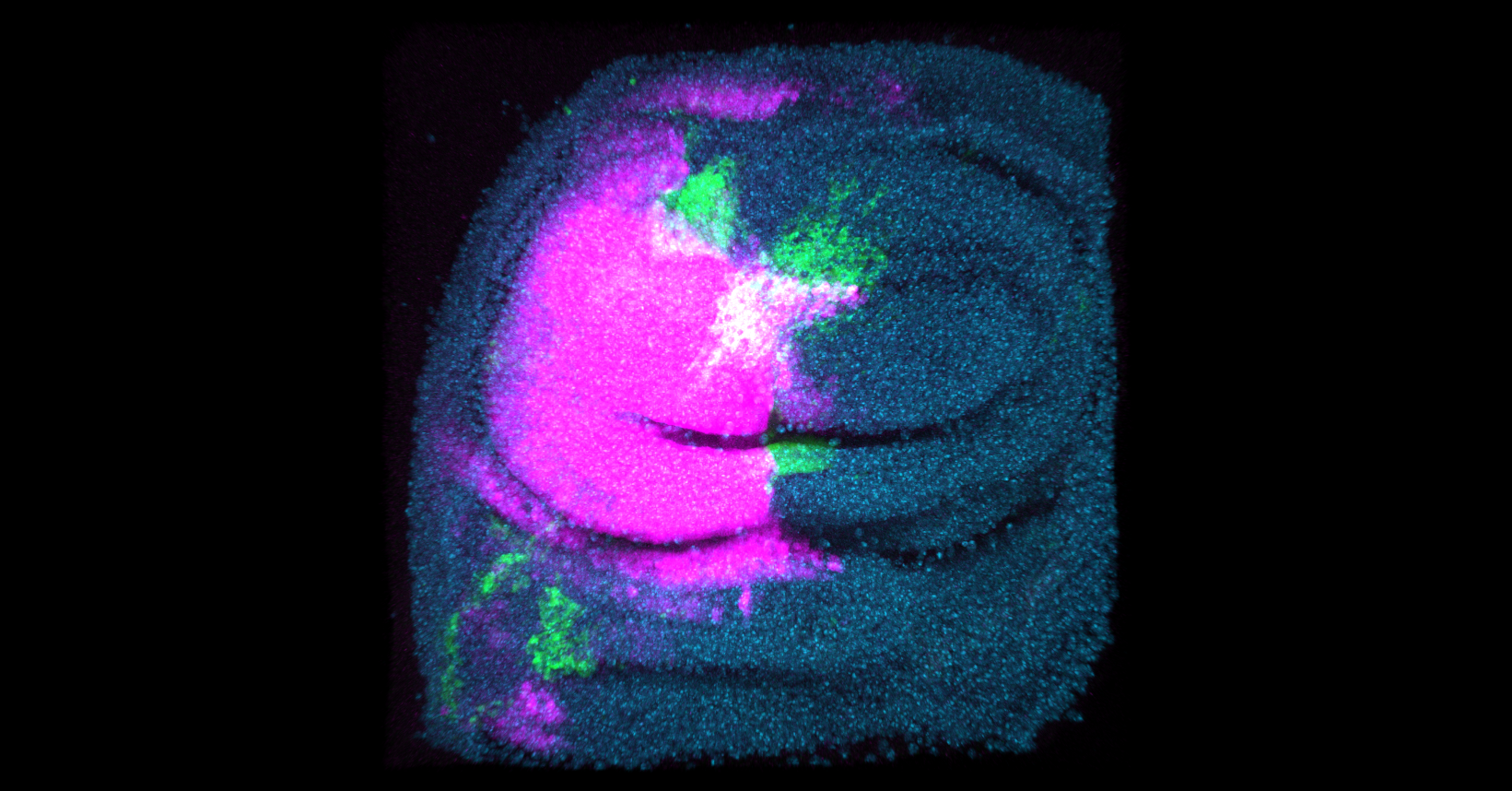

Supplement: Supplementary file 8 — Source data Fig. 6 [file 44319_2026_778_MOESM8_ESM.zip › Figure 6/6K/Fig.6K.tif]

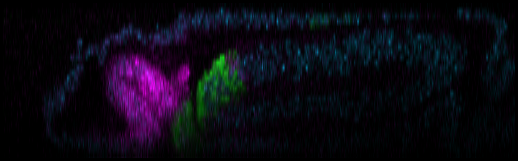

Supplement: Supplementary file 8 — Source data Fig. 6 [file 44319_2026_778_MOESM8_ESM.zip › Figure 6/6K/Fig6.H vertical section.tif]

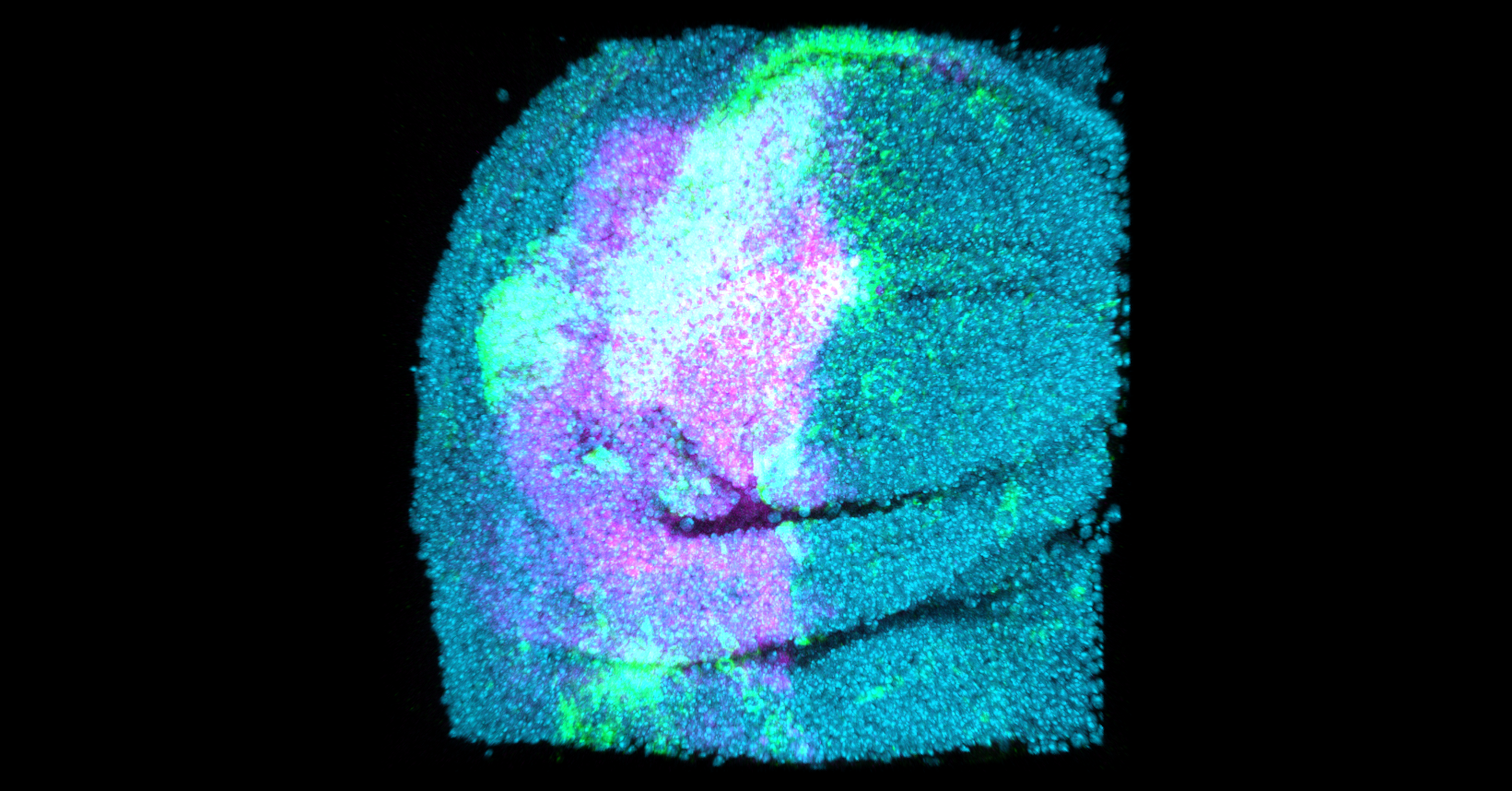

Supplement: Supplementary file 8 — Source data Fig. 6 [file 44319_2026_778_MOESM8_ESM.zip › Figure 6/6J/Fig.6J.tif]

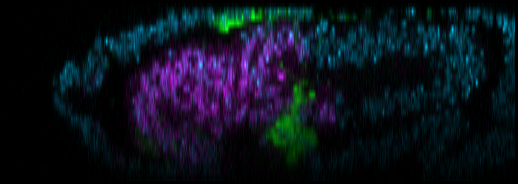

Supplement: Supplementary file 8 — Source data Fig. 6 [file 44319_2026_778_MOESM8_ESM.zip › Figure 6/6J/Fig.6J vertical section.tif]

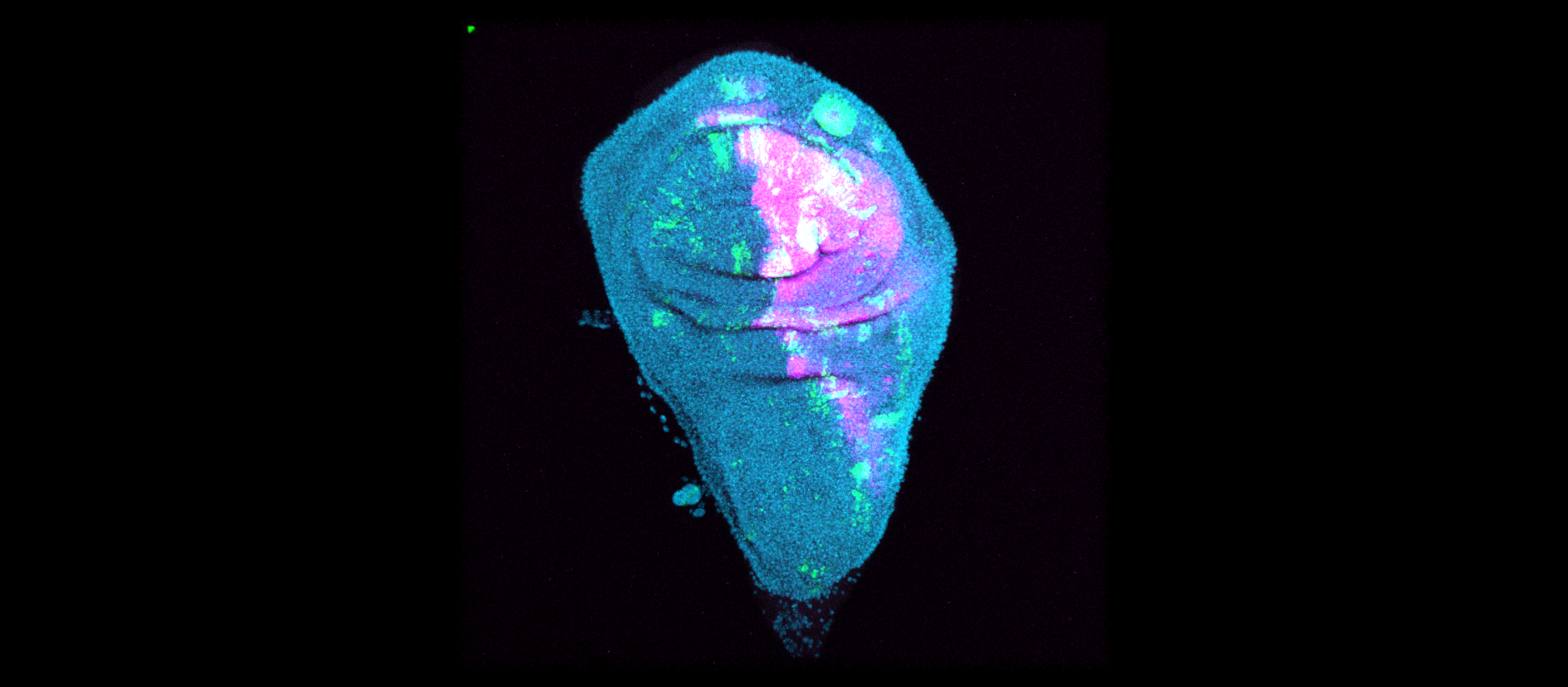

Supplement: Supplementary file 8 — Source data Fig. 6 [file 44319_2026_778_MOESM8_ESM.zip › Figure 6/6D/Fig.6D.tif]

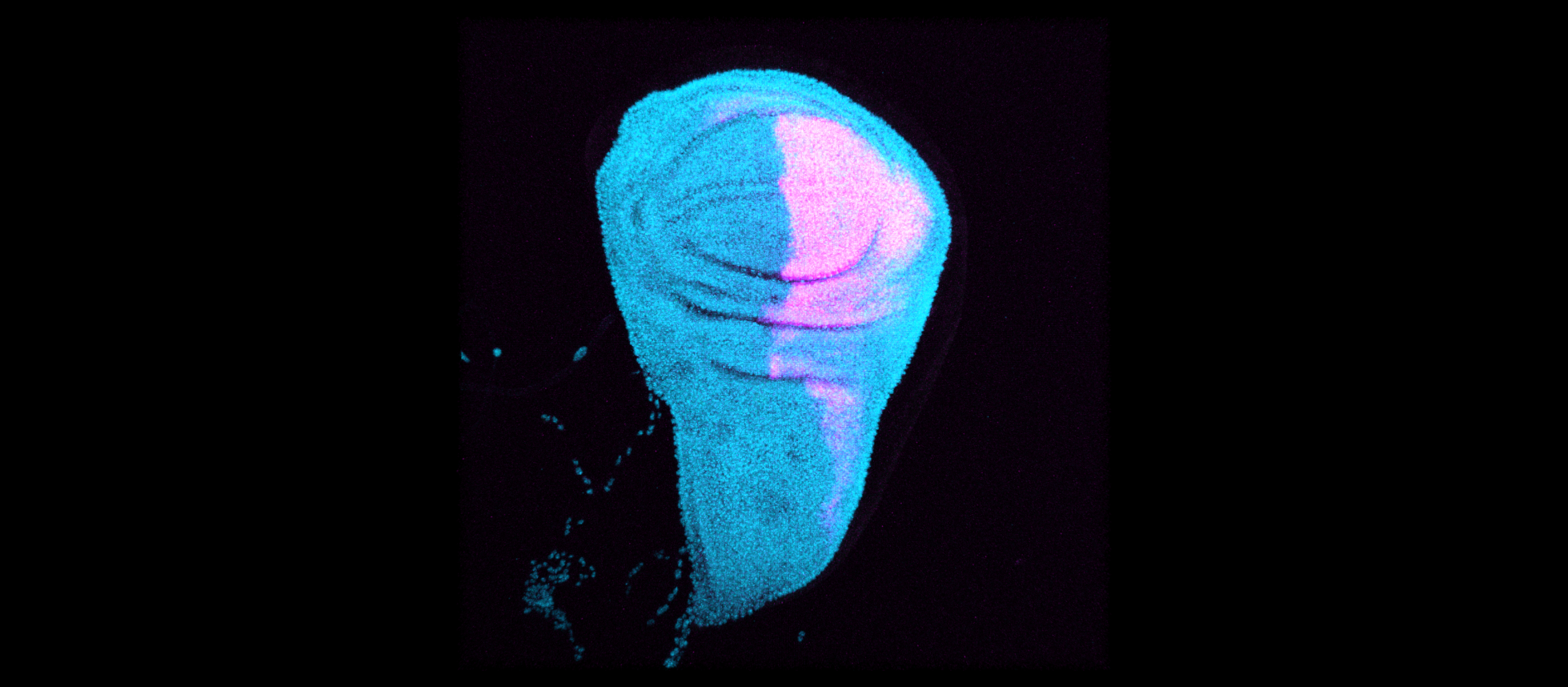

Supplement: Supplementary file 8 — Source data Fig. 6 [file 44319_2026_778_MOESM8_ESM.zip › Figure 6/6C/Fig.6C.tif]

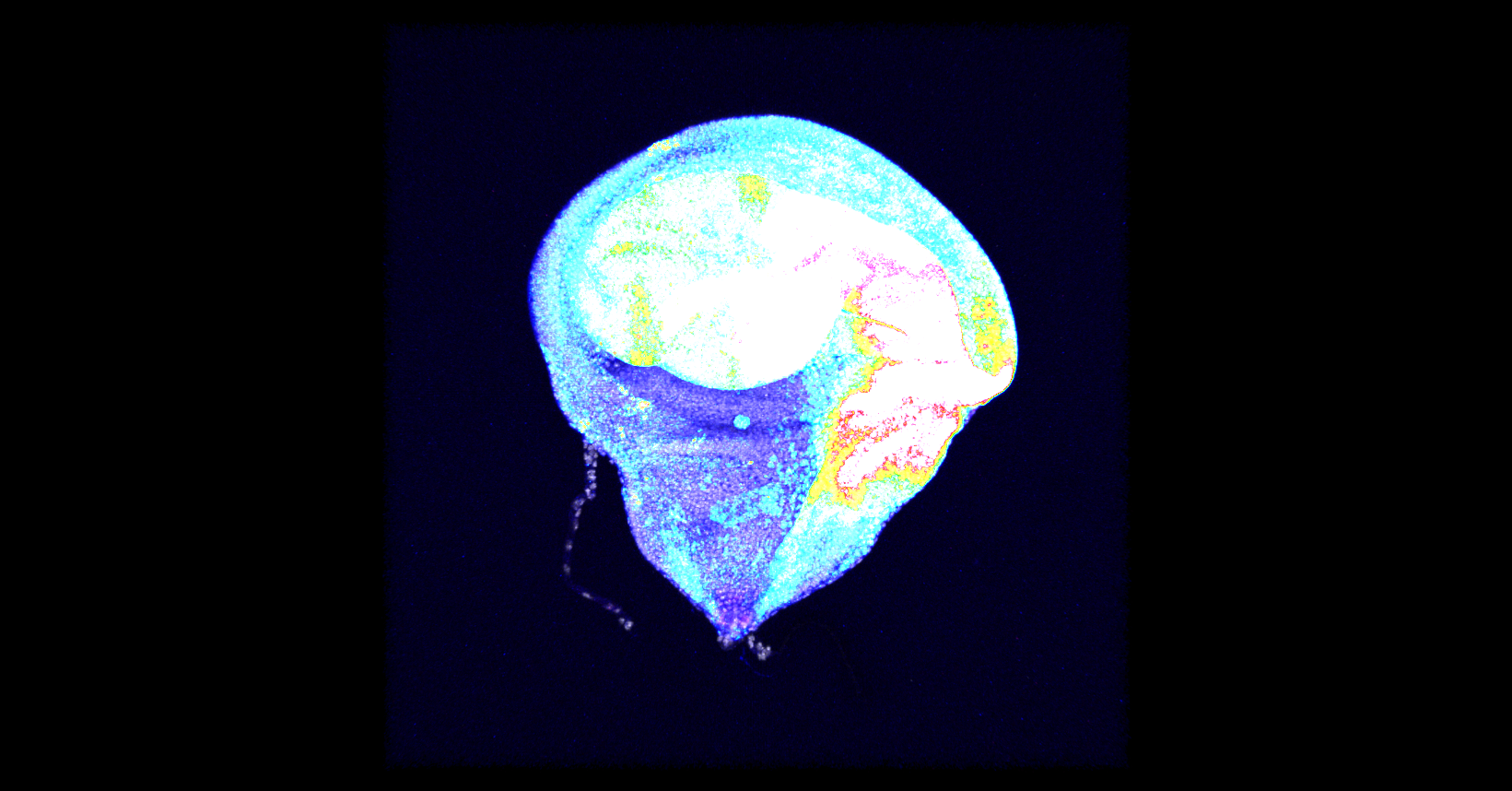

Supplement: Supplementary file 9 — Source data Fig. 7 [file 44319_2026_778_MOESM9_ESM.zip › Figure 7/7J/Fig.7J.tif]

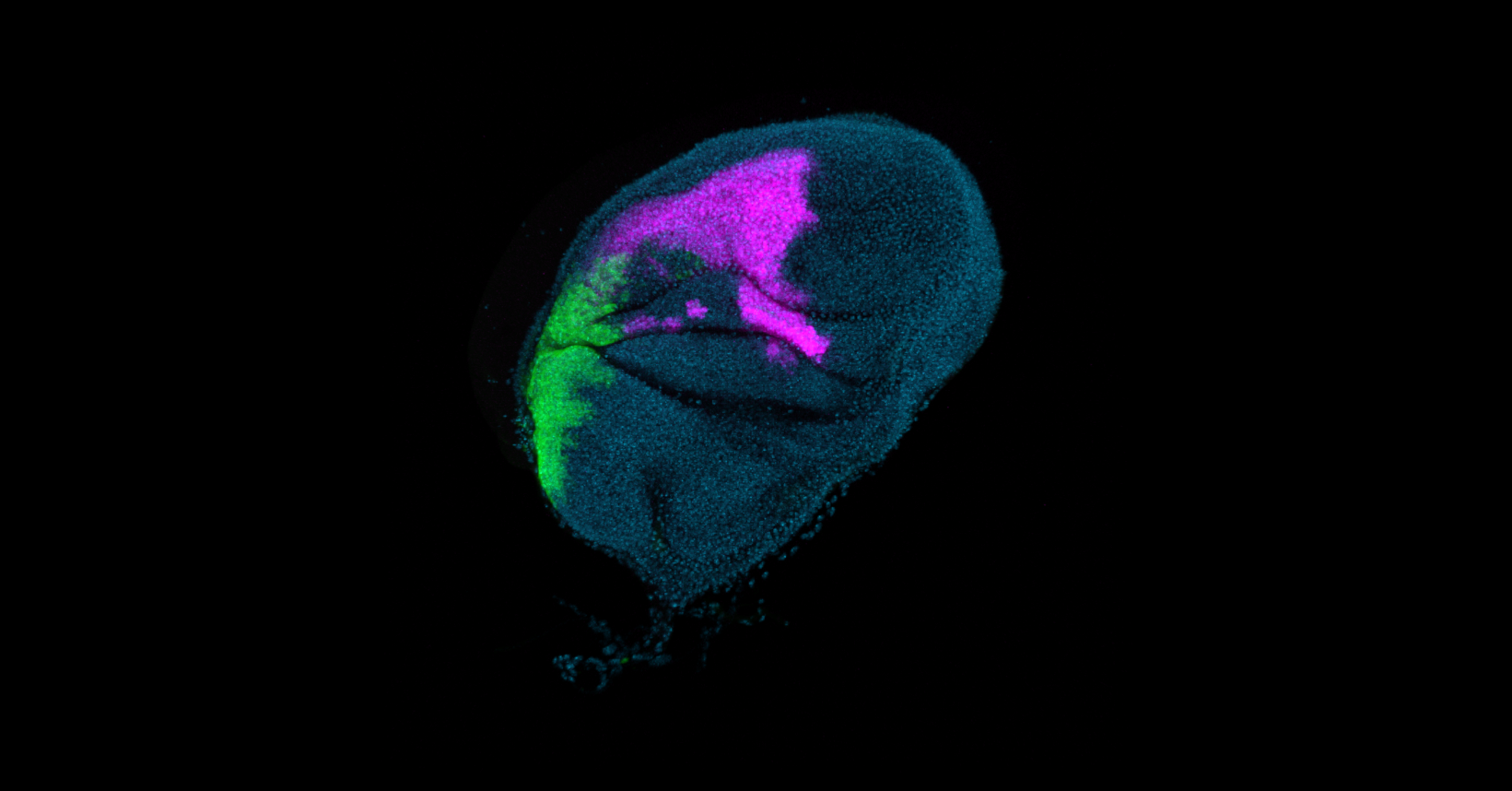

Supplement: Supplementary file 9 — Source data Fig. 7 [file 44319_2026_778_MOESM9_ESM.zip › Figure 7/7M/Fig.7M.tif]

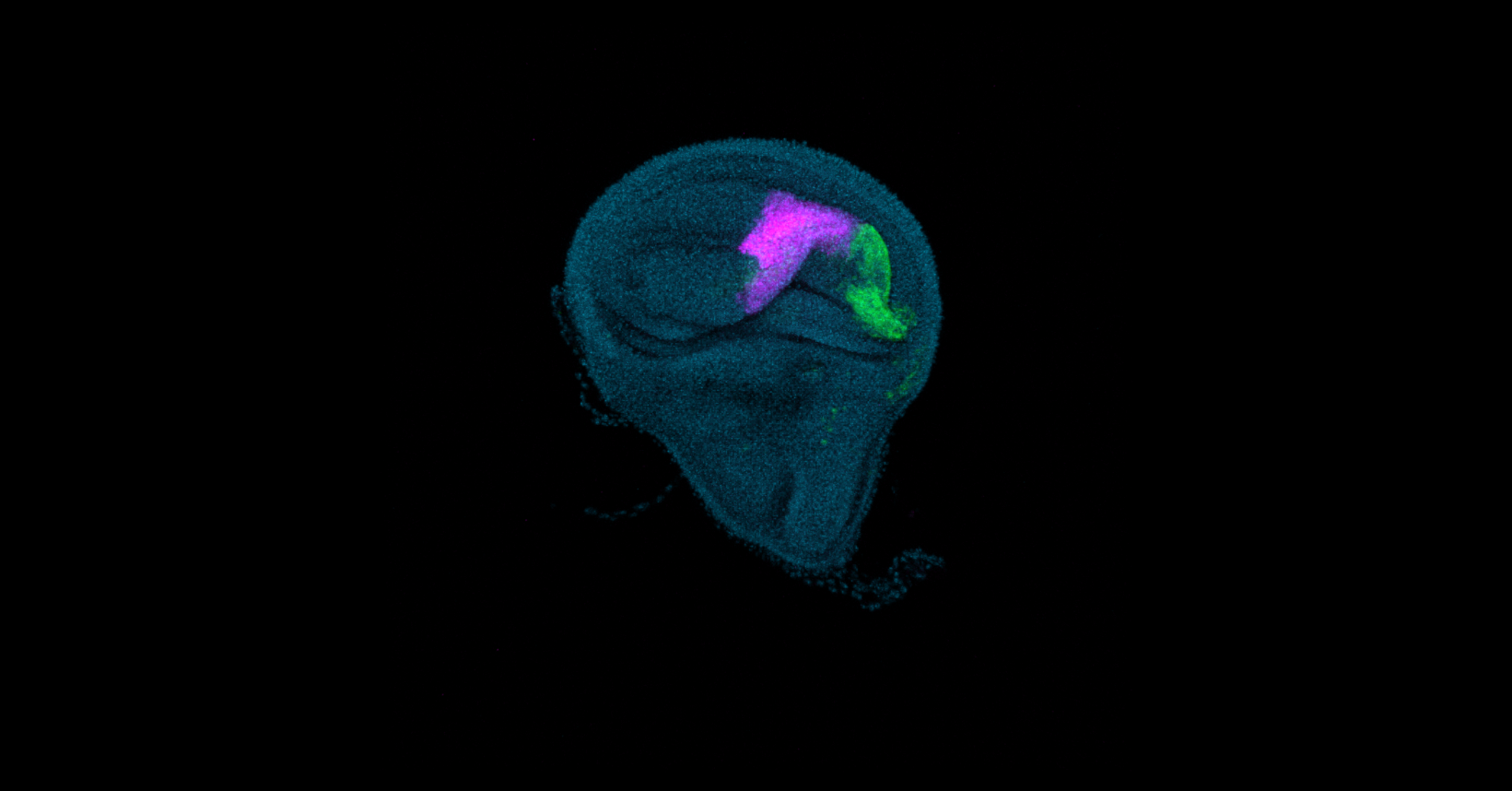

Supplement: Supplementary file 9 — Source data Fig. 7 [file 44319_2026_778_MOESM9_ESM.zip › Figure 7/7D/Fig.7D.tif]

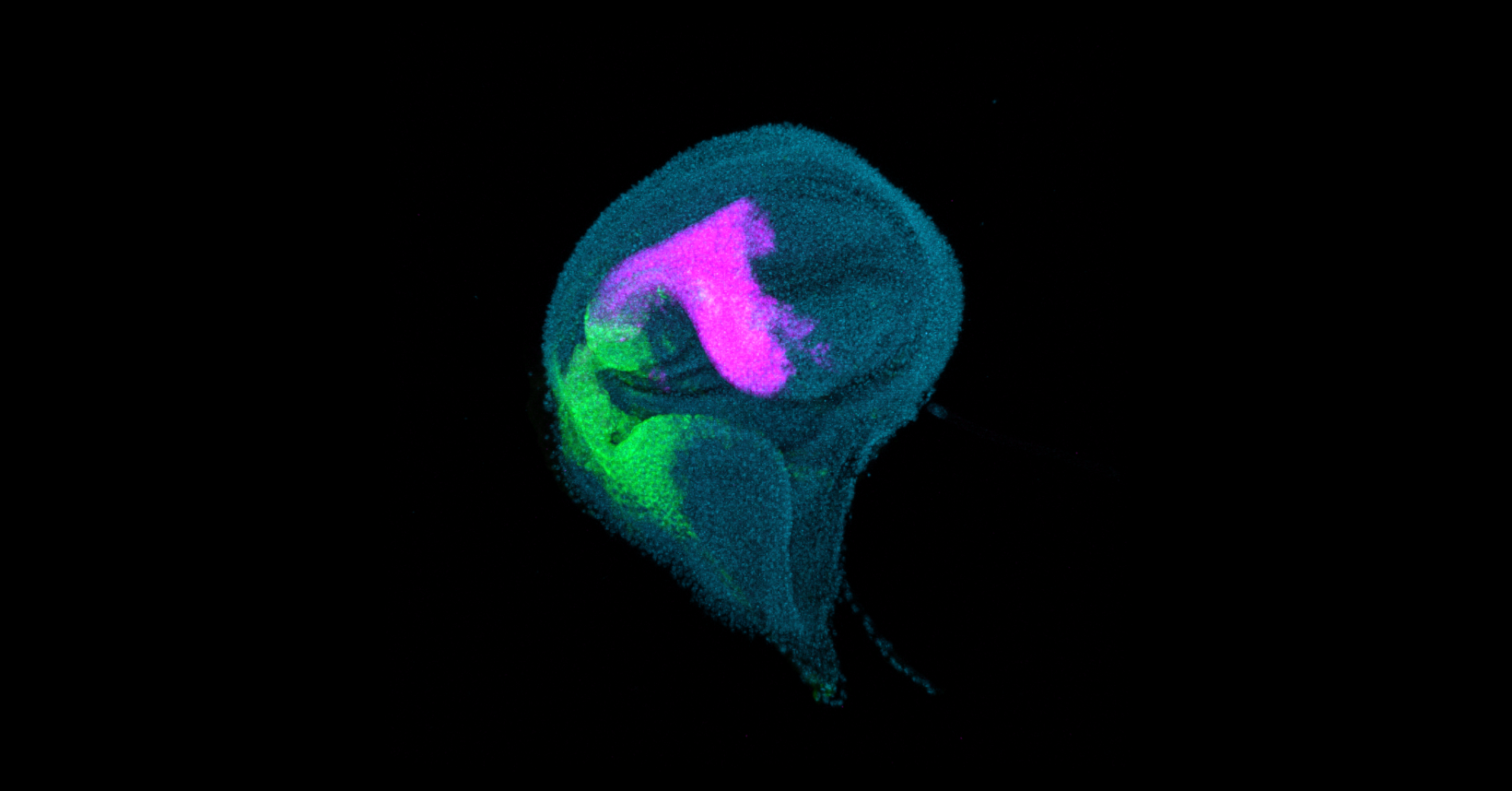

Supplement: Supplementary file 9 — Source data Fig. 7 [file 44319_2026_778_MOESM9_ESM.zip › Figure 7/7C/Fig.7C.tif]

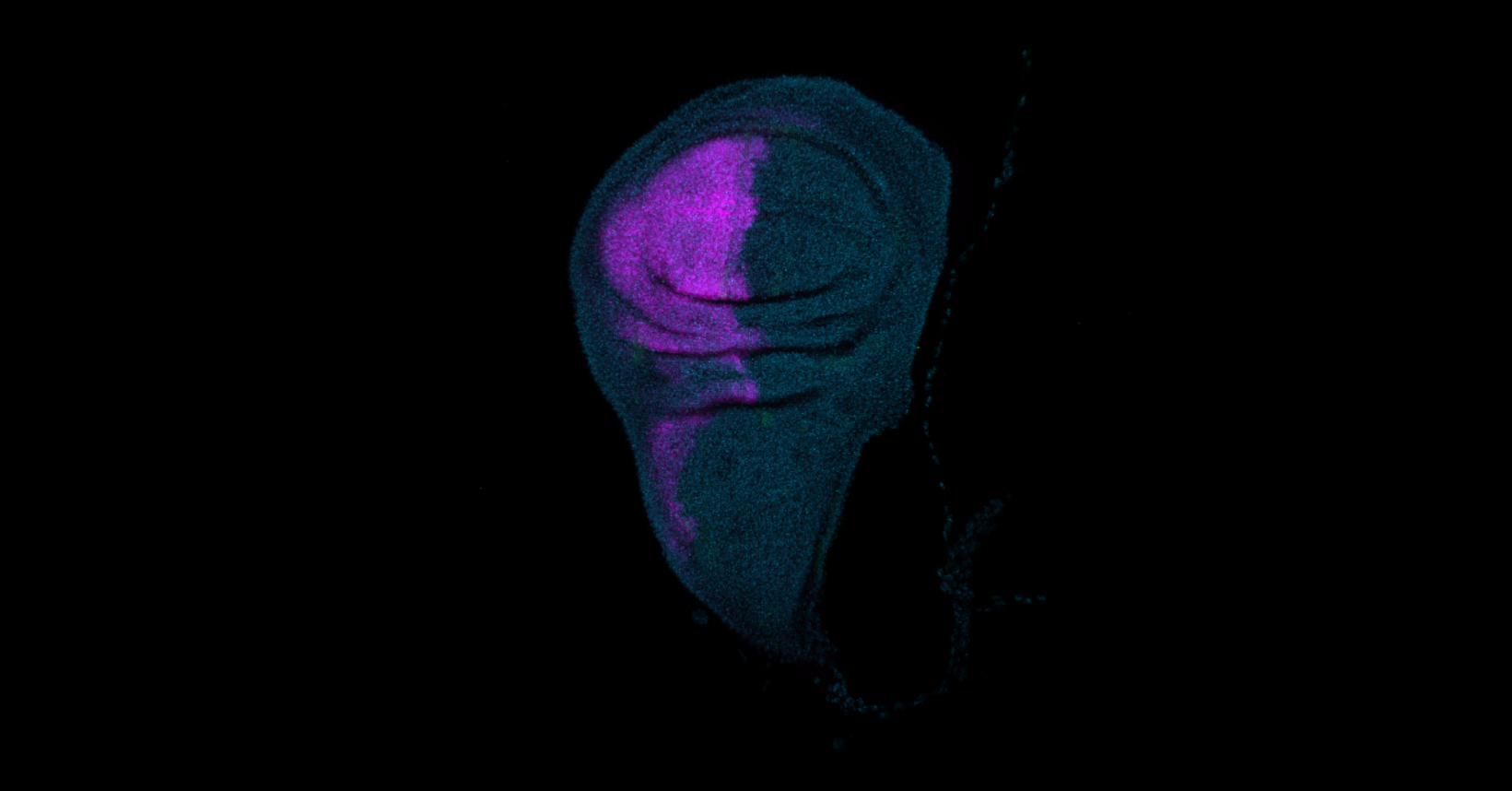

Supplement: Supplementary file 9 — Source data Fig. 7 [file 44319_2026_778_MOESM9_ESM.zip › Figure 7/7B/Fig.7B.tif]

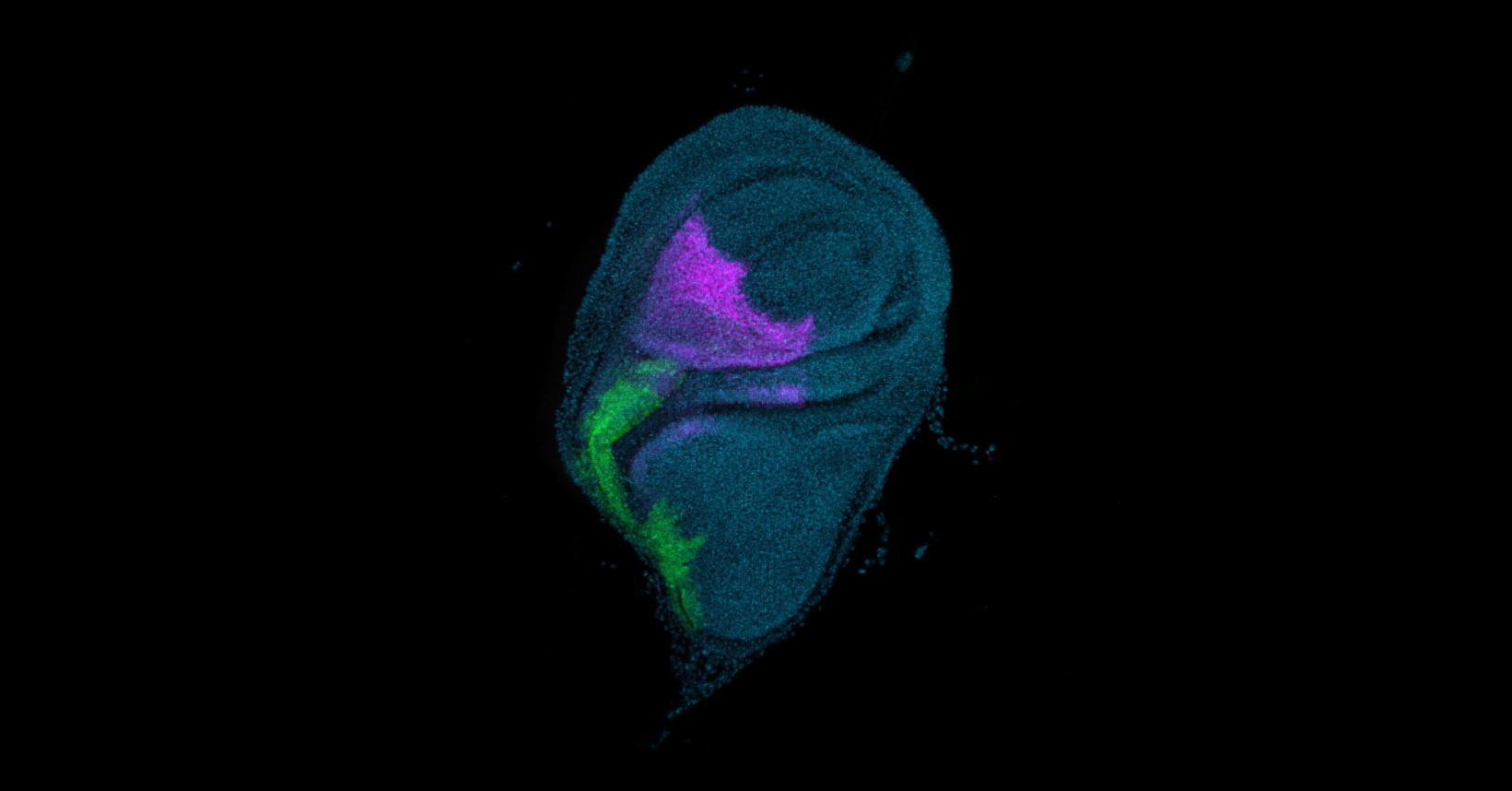

Supplement: Supplementary file 9 — Source data Fig. 7 [file 44319_2026_778_MOESM9_ESM.zip › Figure 7/7E/Fig.7E.tif]

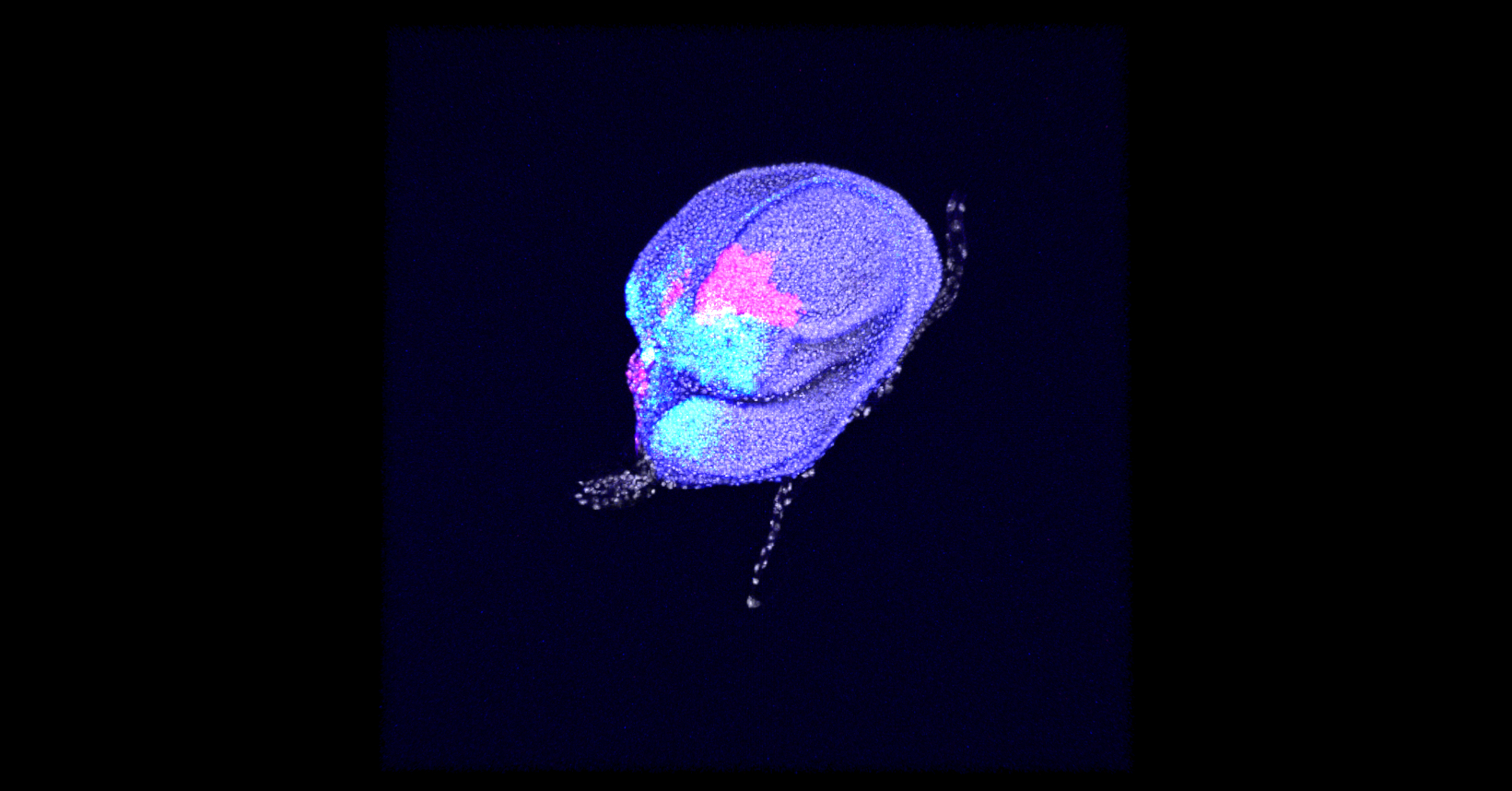

Supplement: Supplementary file 9 — Source data Fig. 7 [file 44319_2026_778_MOESM9_ESM.zip › Figure 7/7K/Fig.7K.tif]

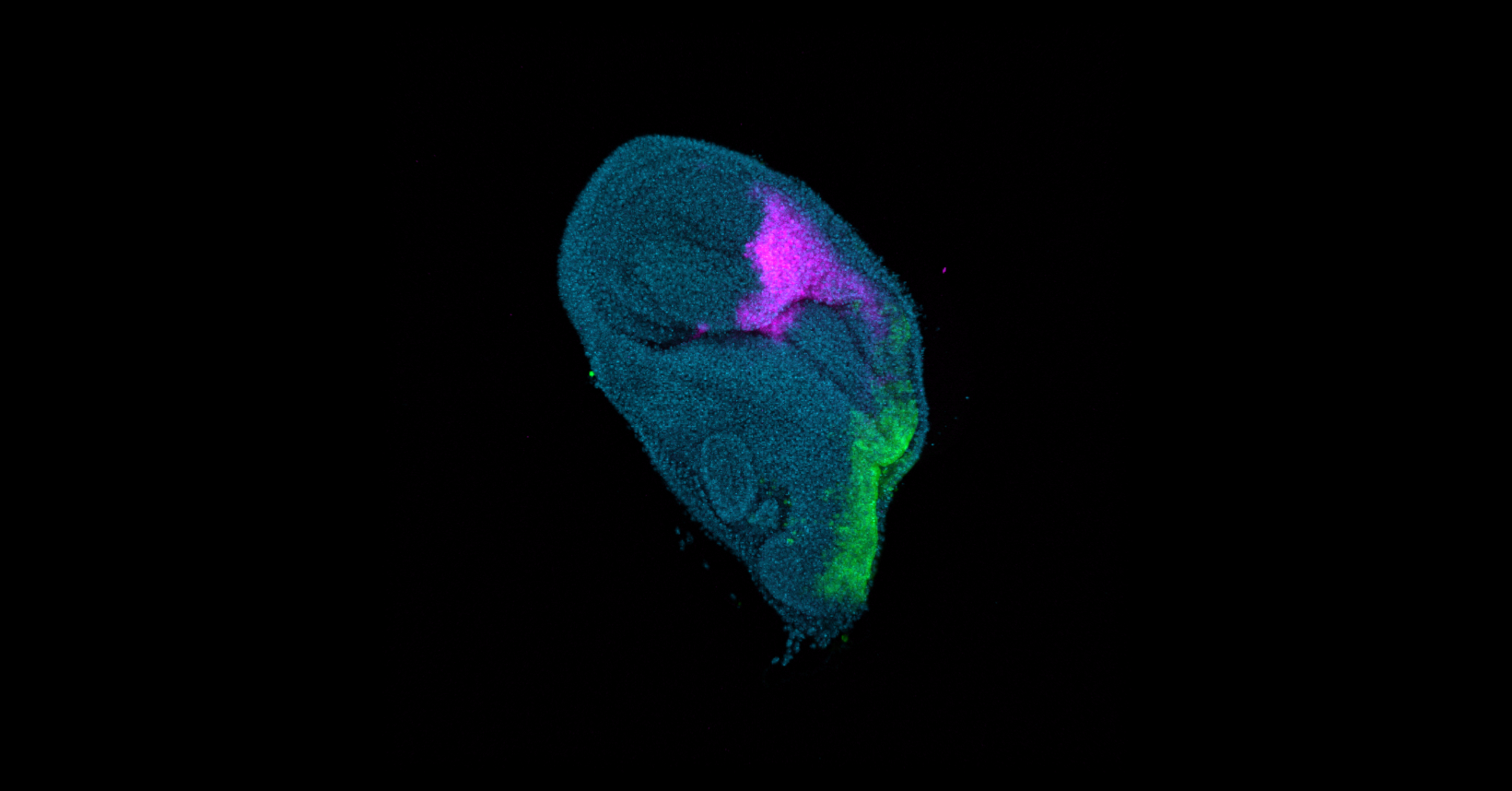

Supplement: Supplementary file 9 — Source data Fig. 7 [file 44319_2026_778_MOESM9_ESM.zip › Figure 7/7N/Fig.7N.tif]

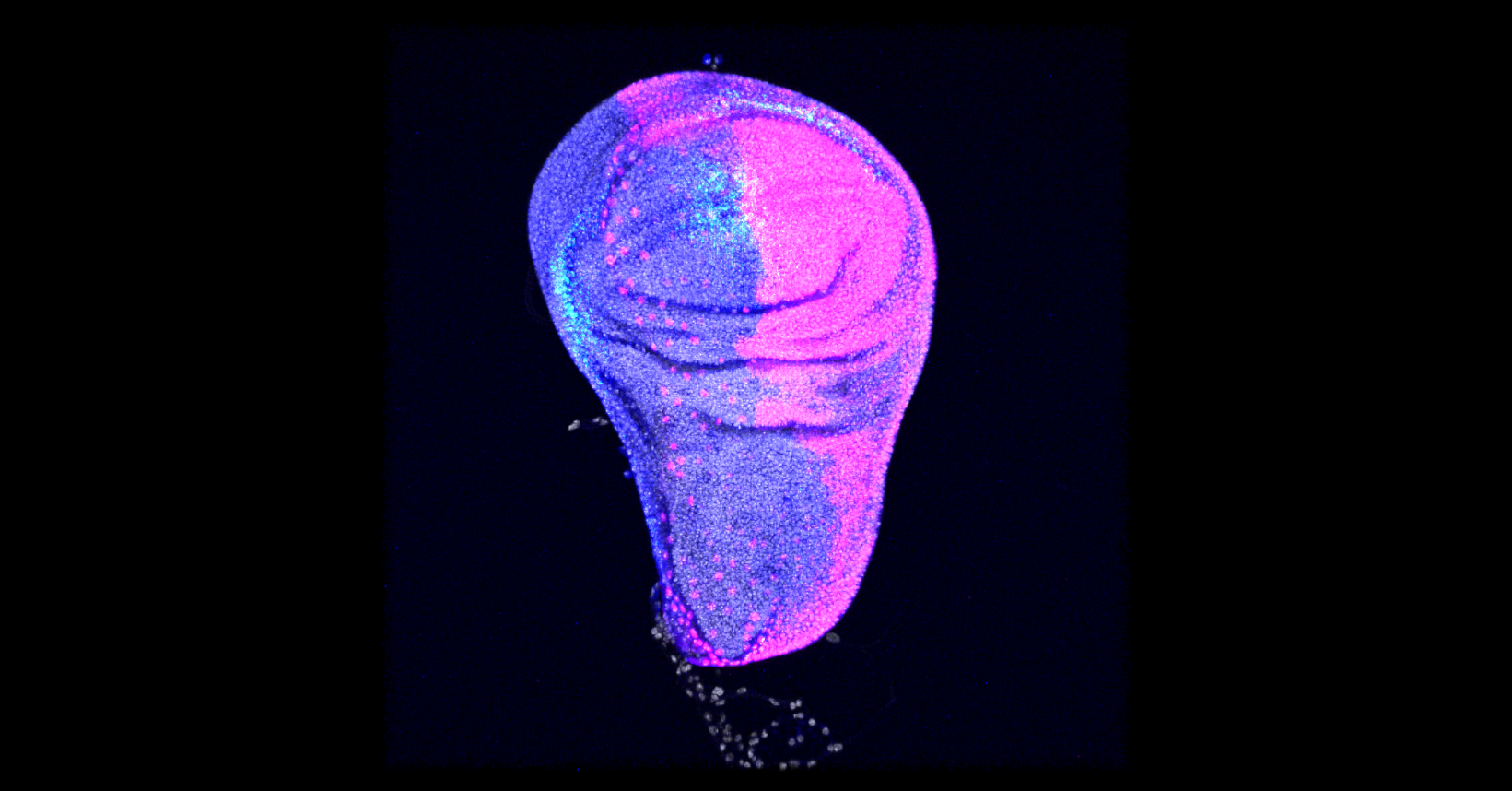

Supplement: Supplementary file 9 — Source data Fig. 7 [file 44319_2026_778_MOESM9_ESM.zip › Figure 7/7I/Fig.7I.tif]

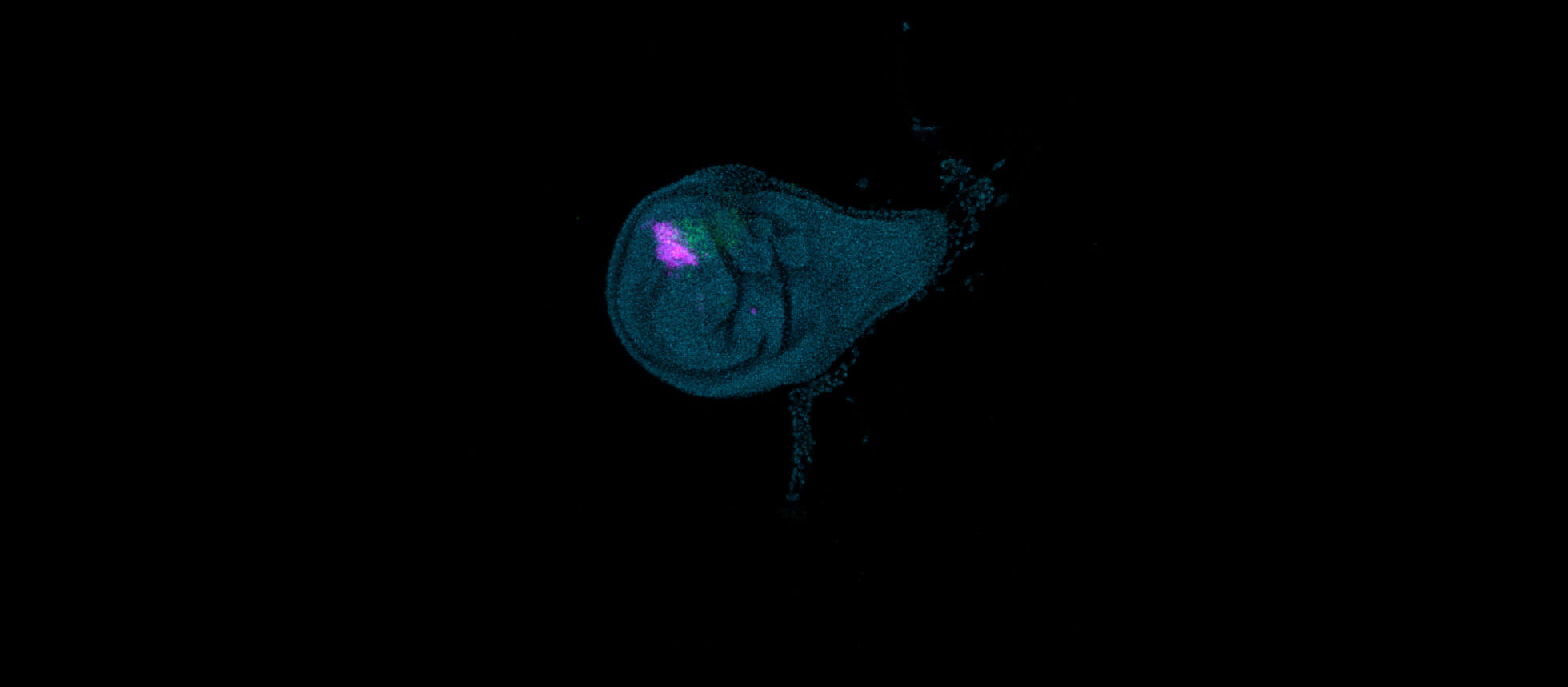

Supplement: Supplementary file 9 — Source data Fig. 7 [file 44319_2026_778_MOESM9_ESM.zip › Figure 7/7F/Fig.7F.tif]

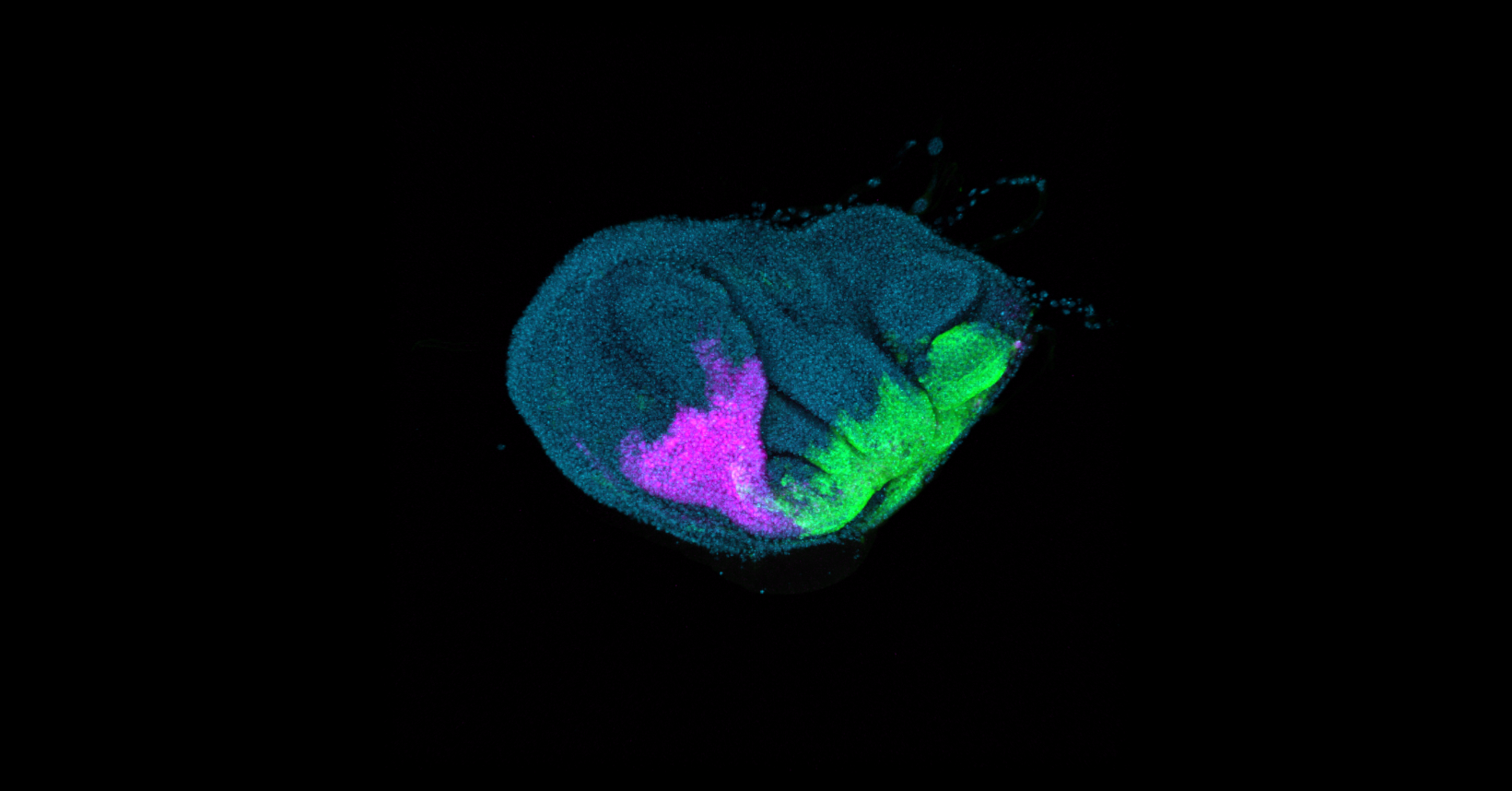

Supplement: Supplementary file 9 — Source data Fig. 7 [file 44319_2026_778_MOESM9_ESM.zip › Figure 7/7O/Fig.7O.tif]

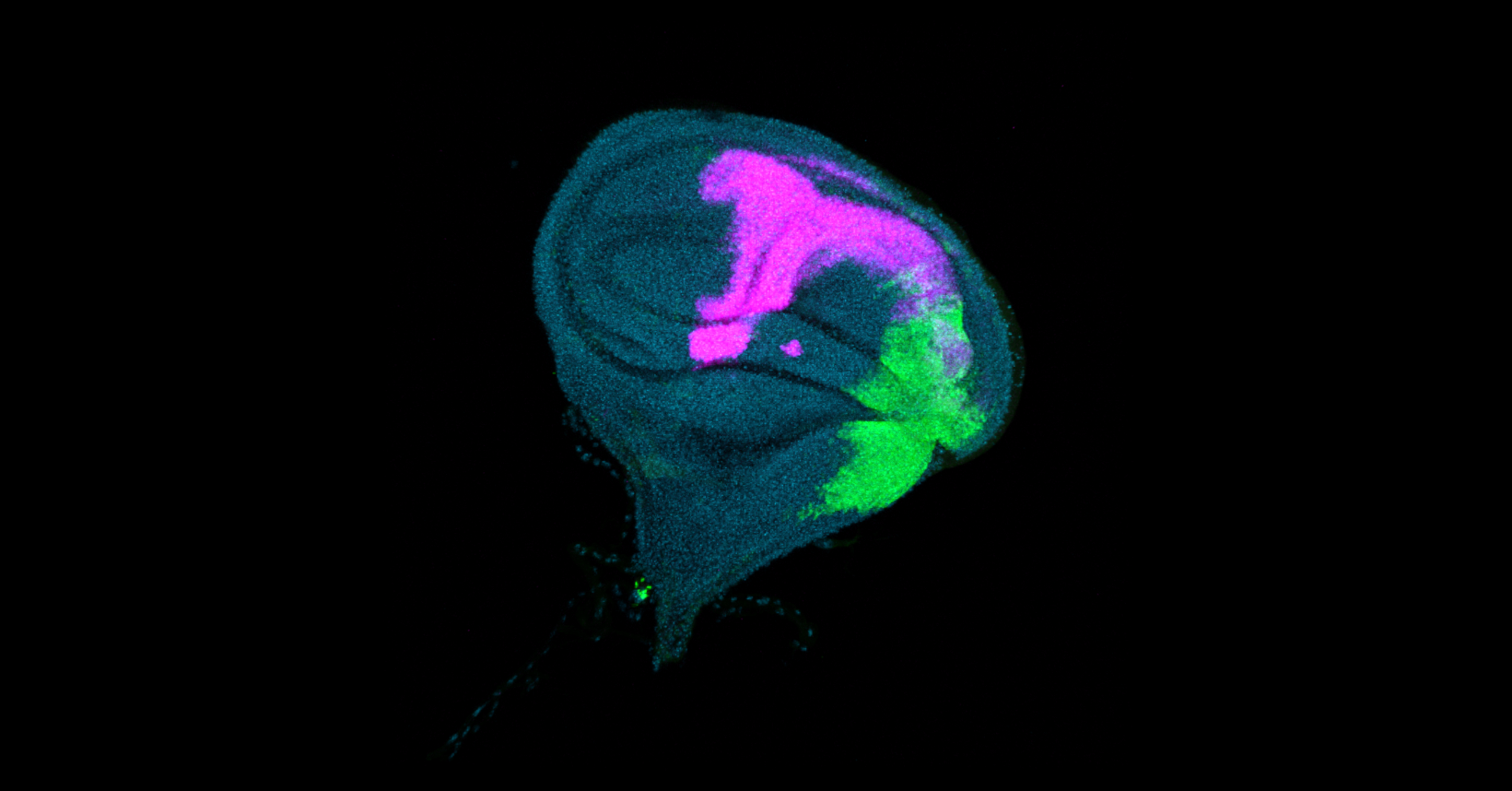

Supplement: Supplementary file 10 — Source data Fig. 8 [file 44319_2026_778_MOESM10_ESM.zip › Figure 8/8I/Fig.8I.tif]

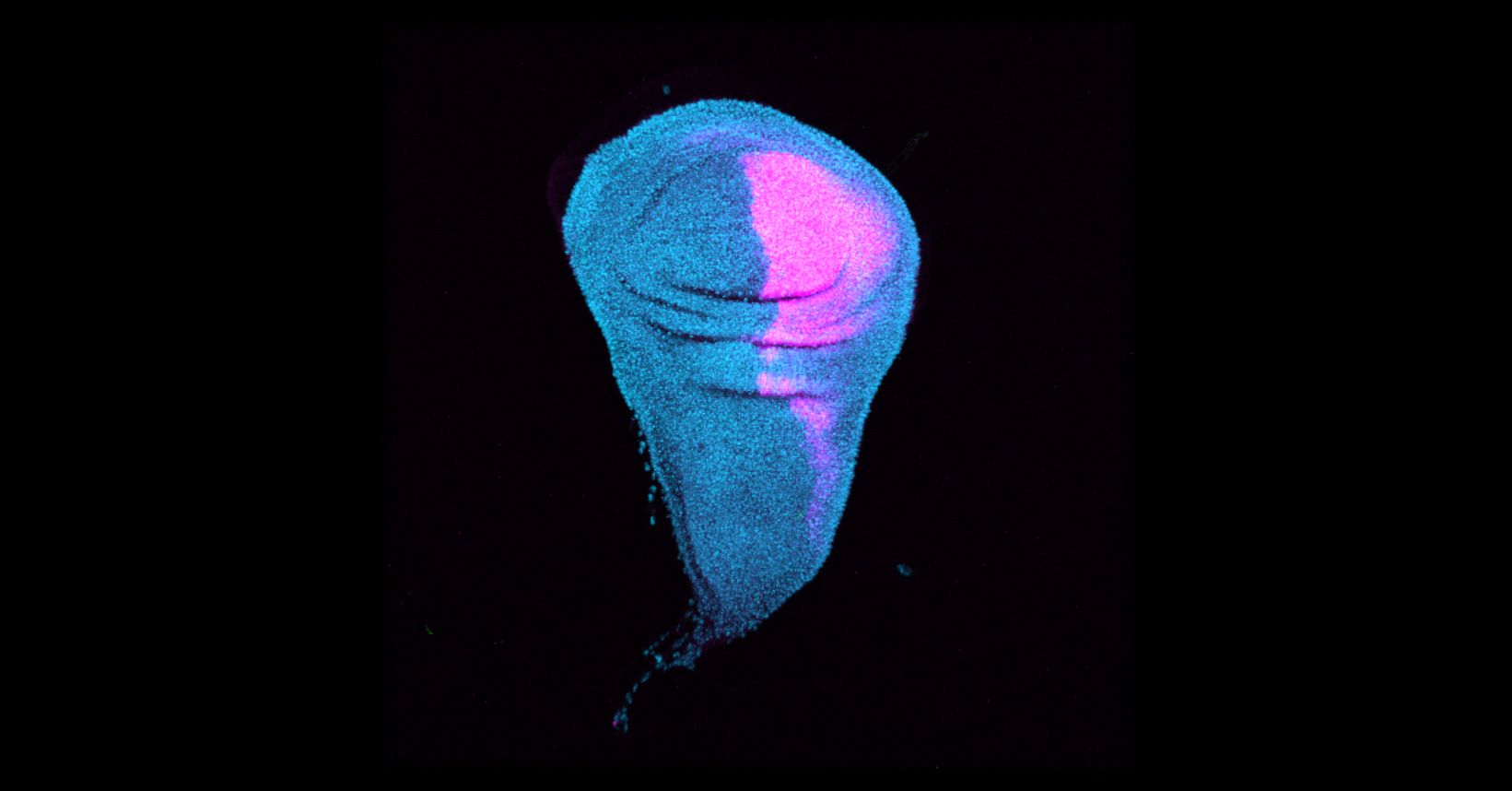

Supplement: Supplementary file 10 — Source data Fig. 8 [file 44319_2026_778_MOESM10_ESM.zip › Figure 8/8A/Fig.8A.tif]

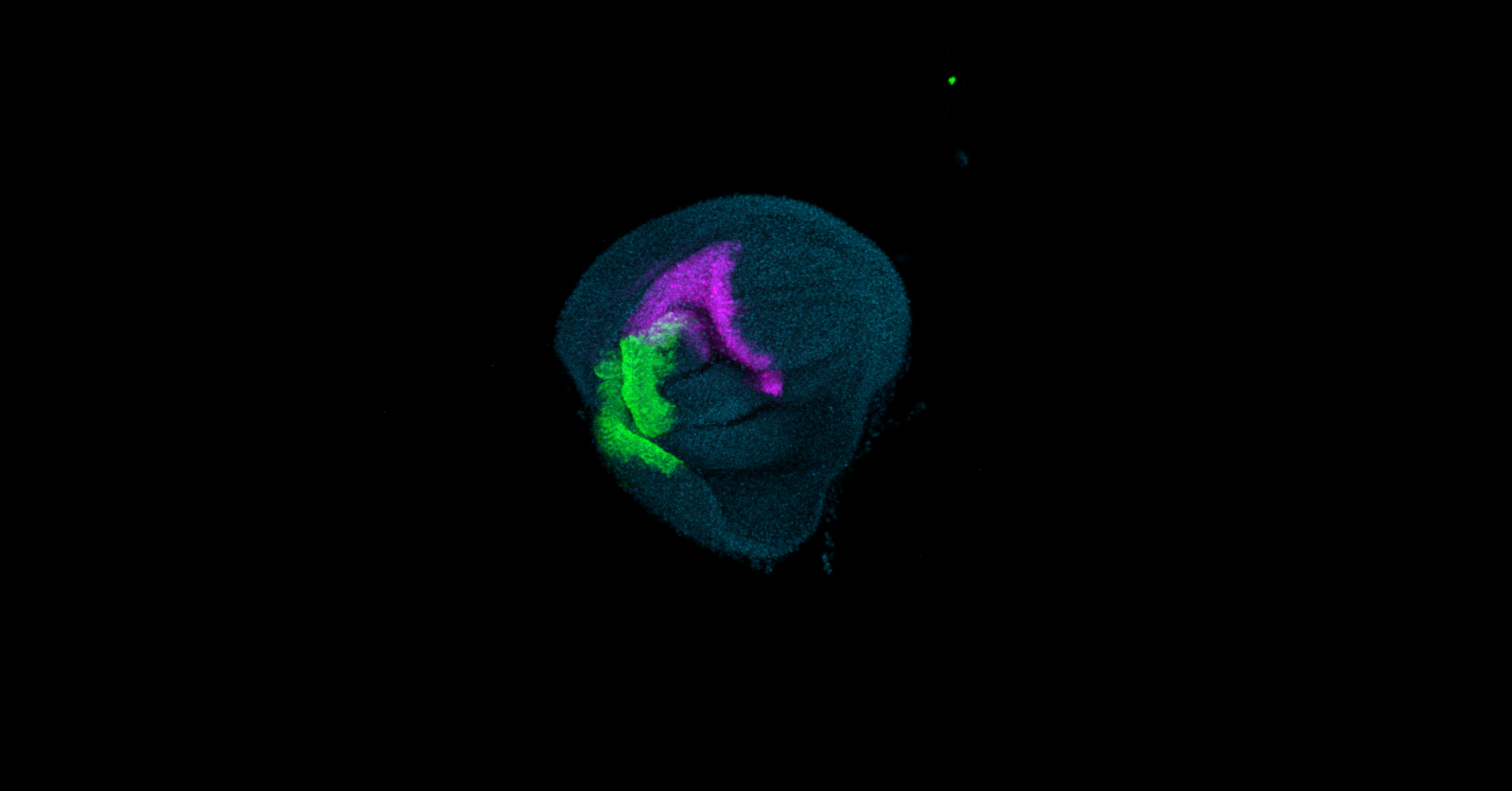

Supplement: Supplementary file 10 — Source data Fig. 8 [file 44319_2026_778_MOESM10_ESM.zip › Figure 8/8H/Fig.8H.tif]

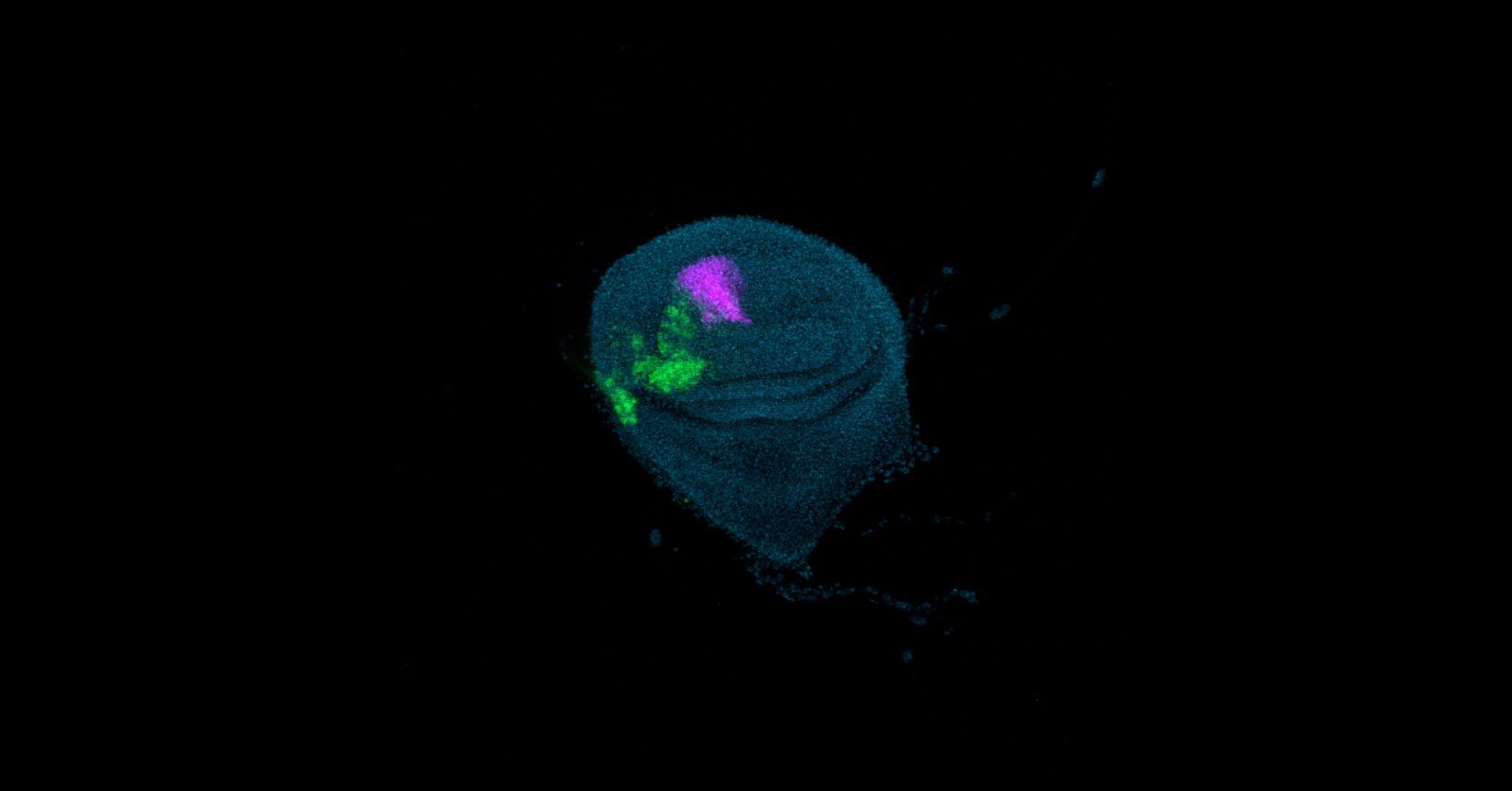

Supplement: Supplementary file 10 — Source data Fig. 8 [file 44319_2026_778_MOESM10_ESM.zip › Figure 8/8J/Fig.8J.tif]

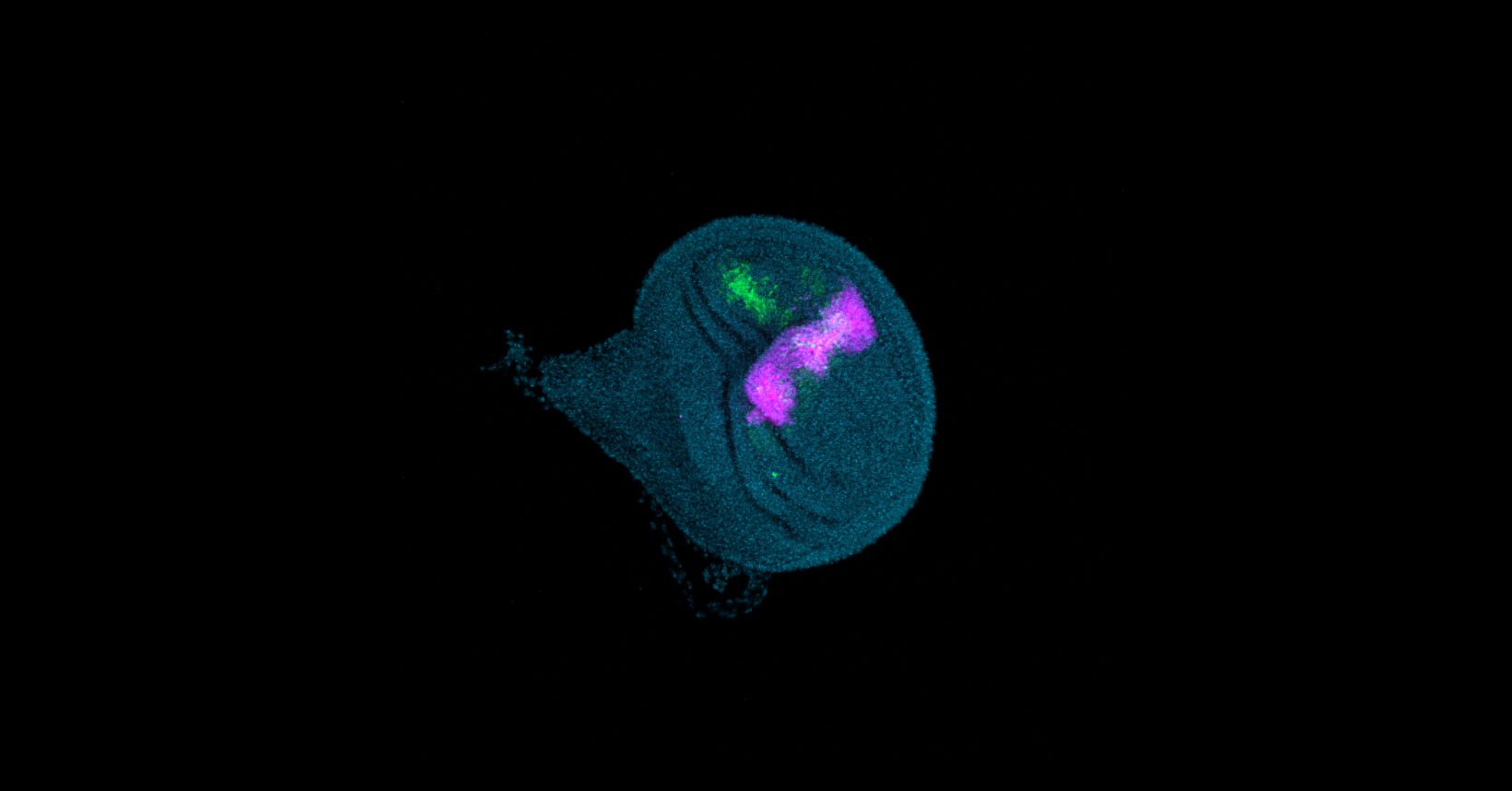

Supplement: Supplementary file 10 — Source data Fig. 8 [file 44319_2026_778_MOESM10_ESM.zip › Figure 8/8M/Fig.8M.tif]

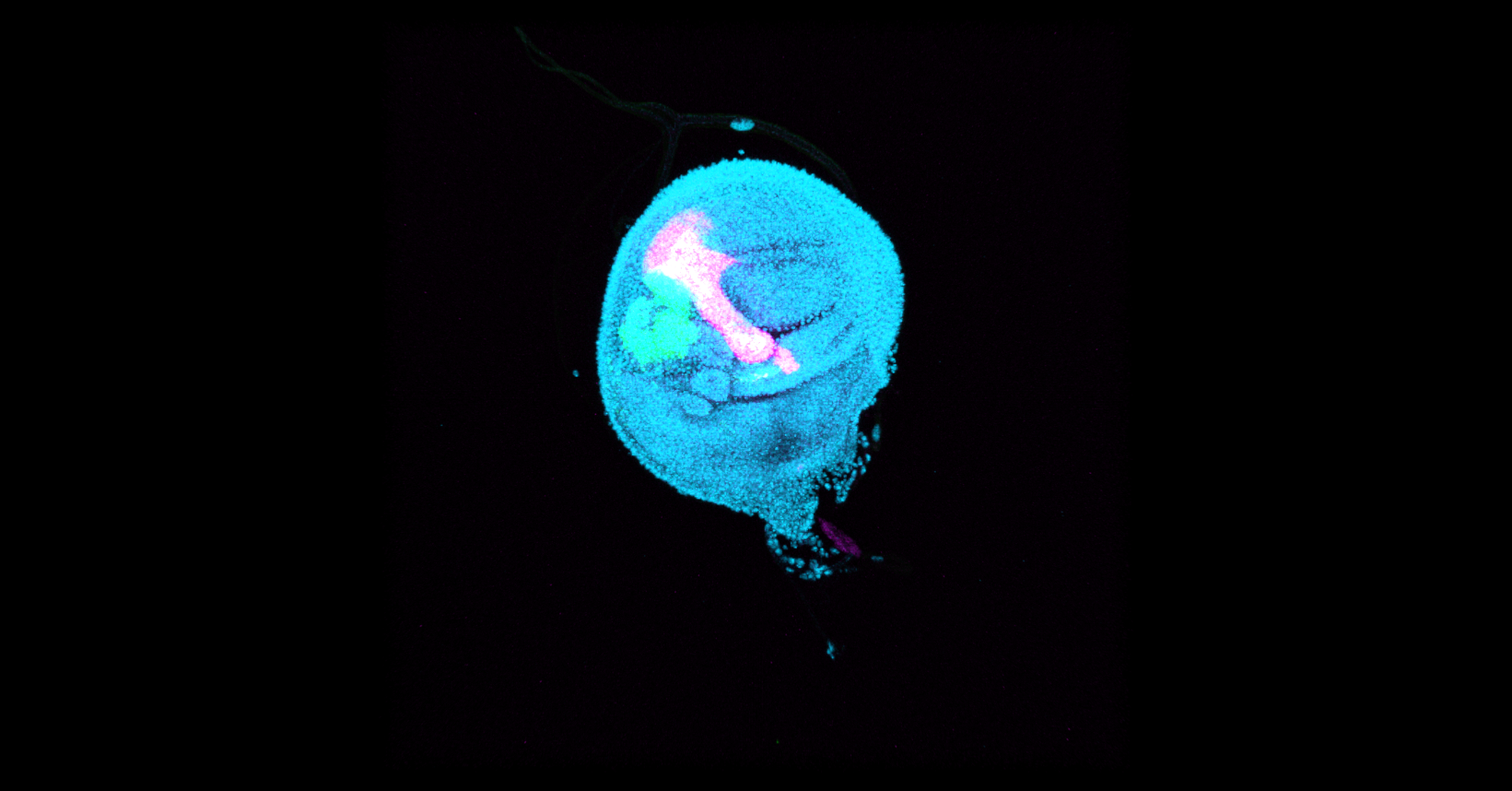

Supplement: Supplementary file 10 — Source data Fig. 8 [file 44319_2026_778_MOESM10_ESM.zip › Figure 8/8D/Fig.8D.tif]

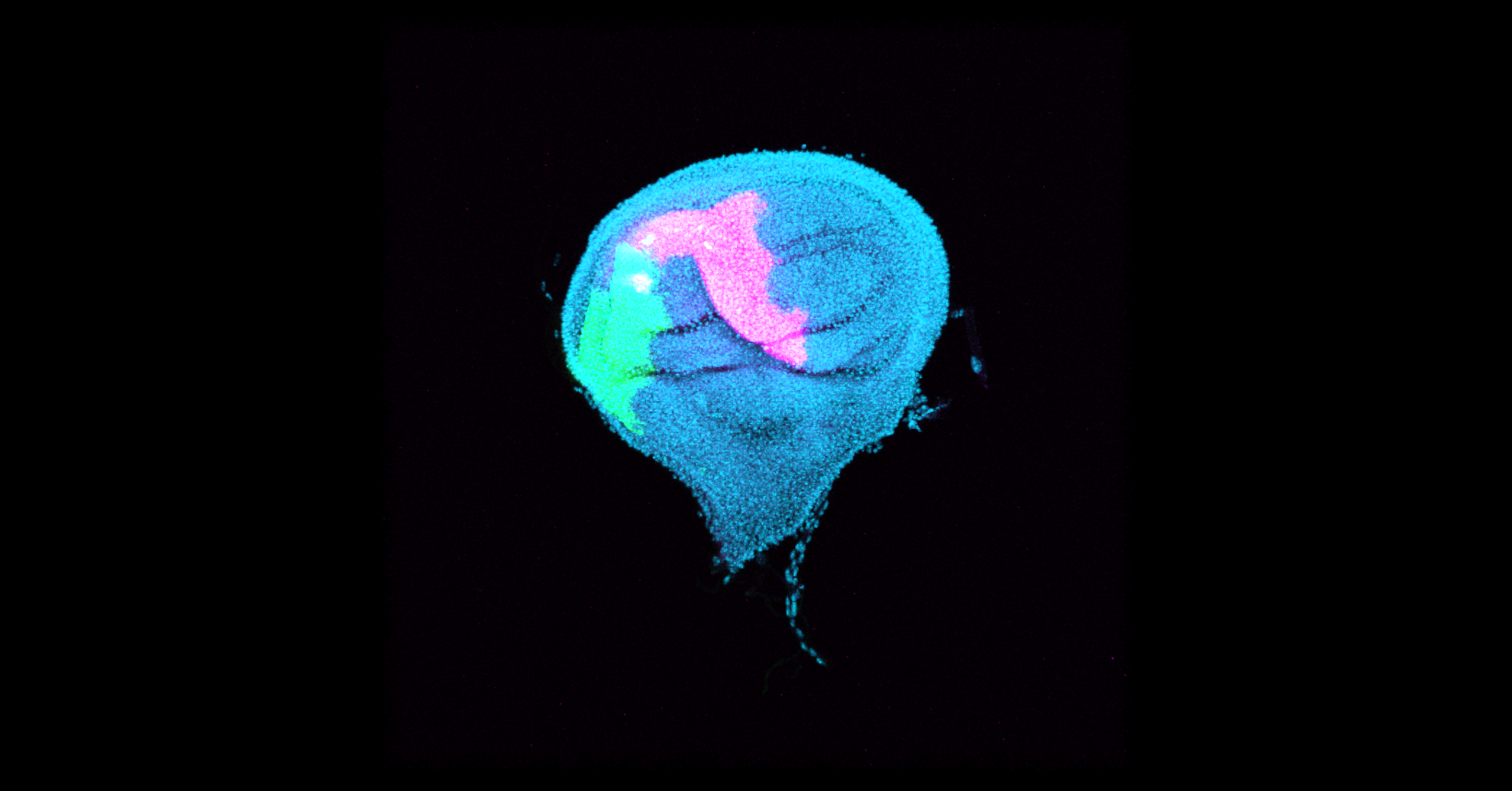

Supplement: Supplementary file 10 — Source data Fig. 8 [file 44319_2026_778_MOESM10_ESM.zip › Figure 8/8C/Fig.8C.tif]

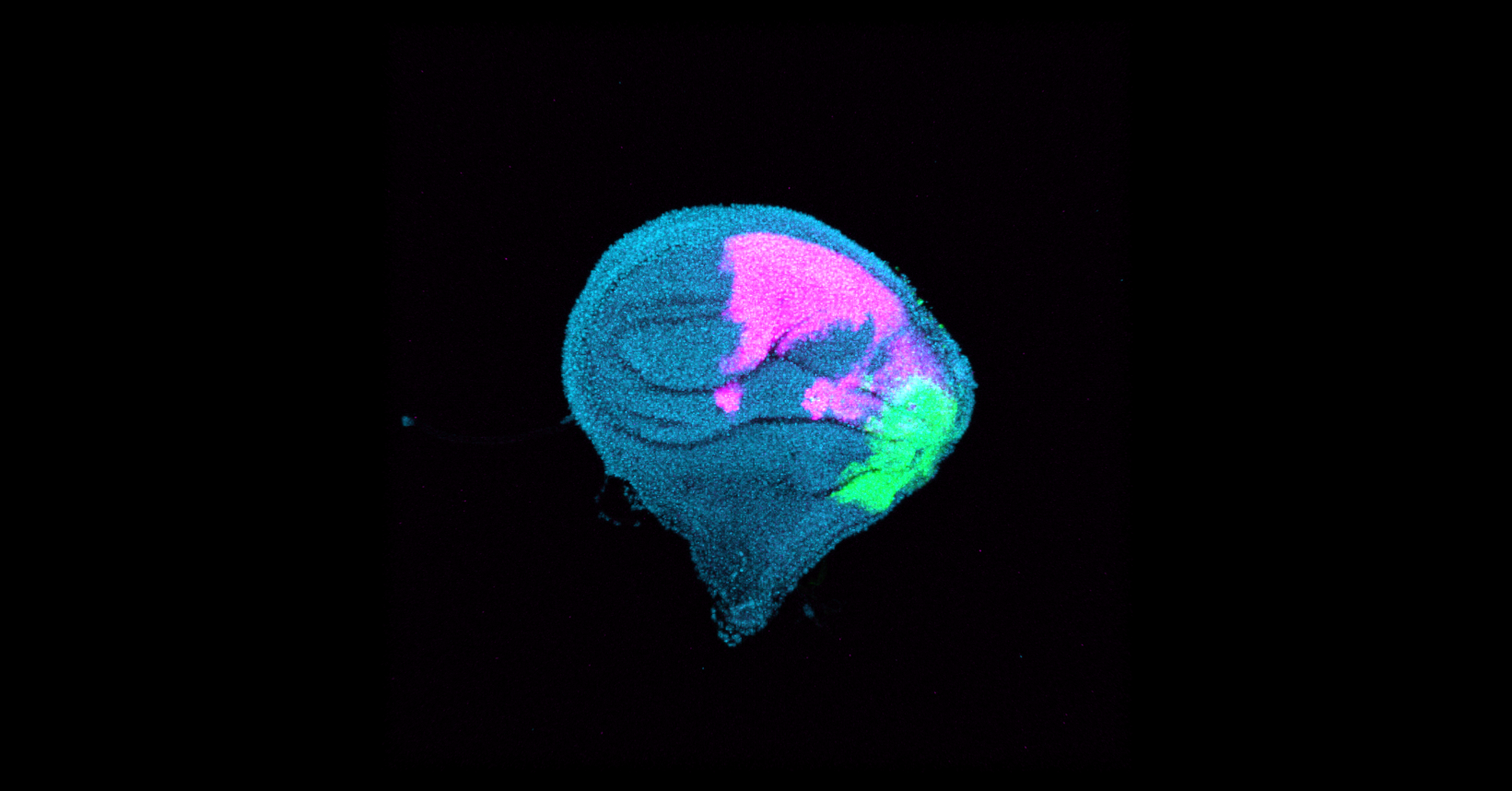

Supplement: Supplementary file 10 — Source data Fig. 8 [file 44319_2026_778_MOESM10_ESM.zip › Figure 8/8B/Fig.8B.tif]

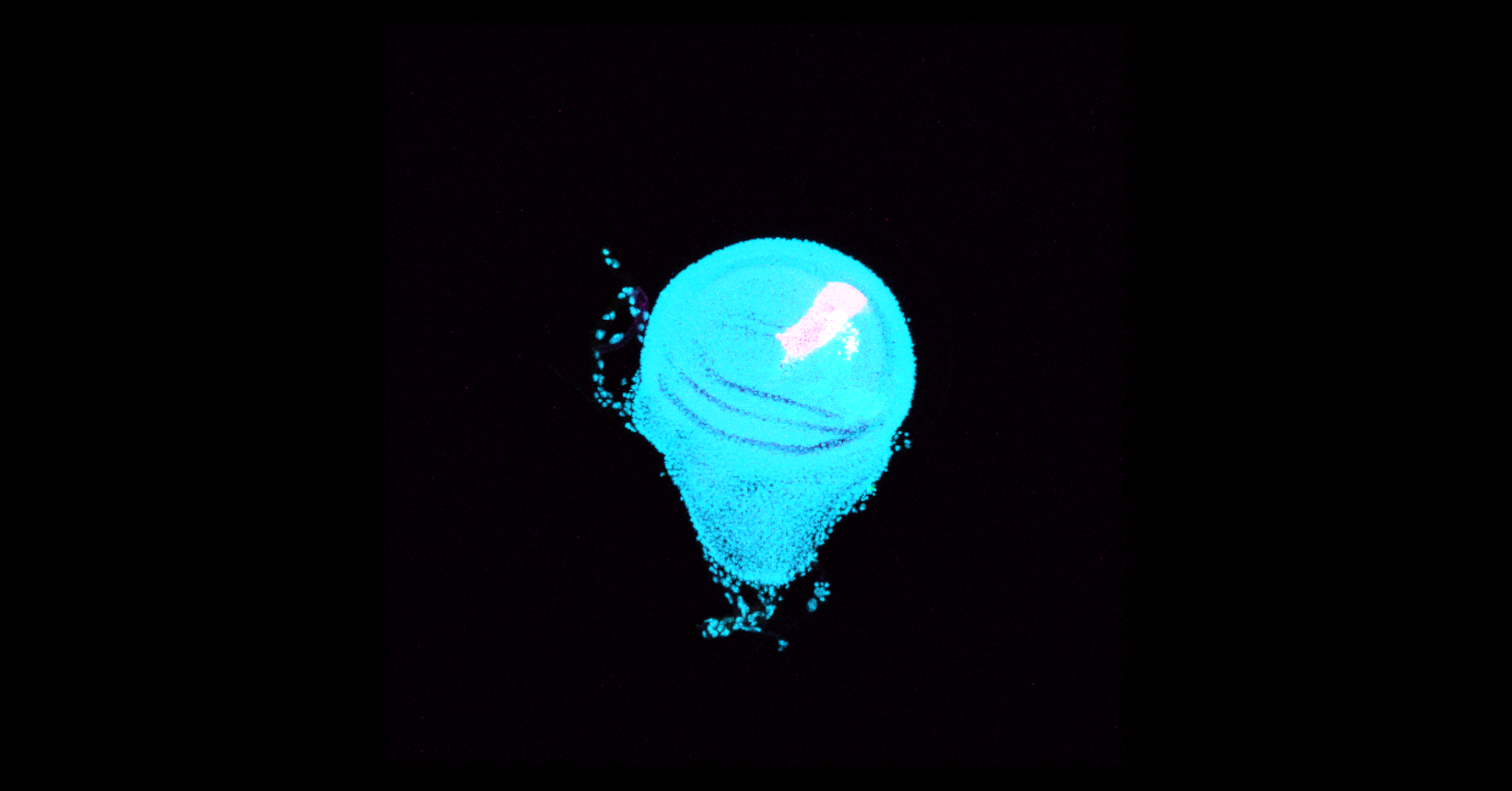

Supplement: Supplementary file 10 — Source data Fig. 8 [file 44319_2026_778_MOESM10_ESM.zip › Figure 8/8E/Fig.8E.tif]

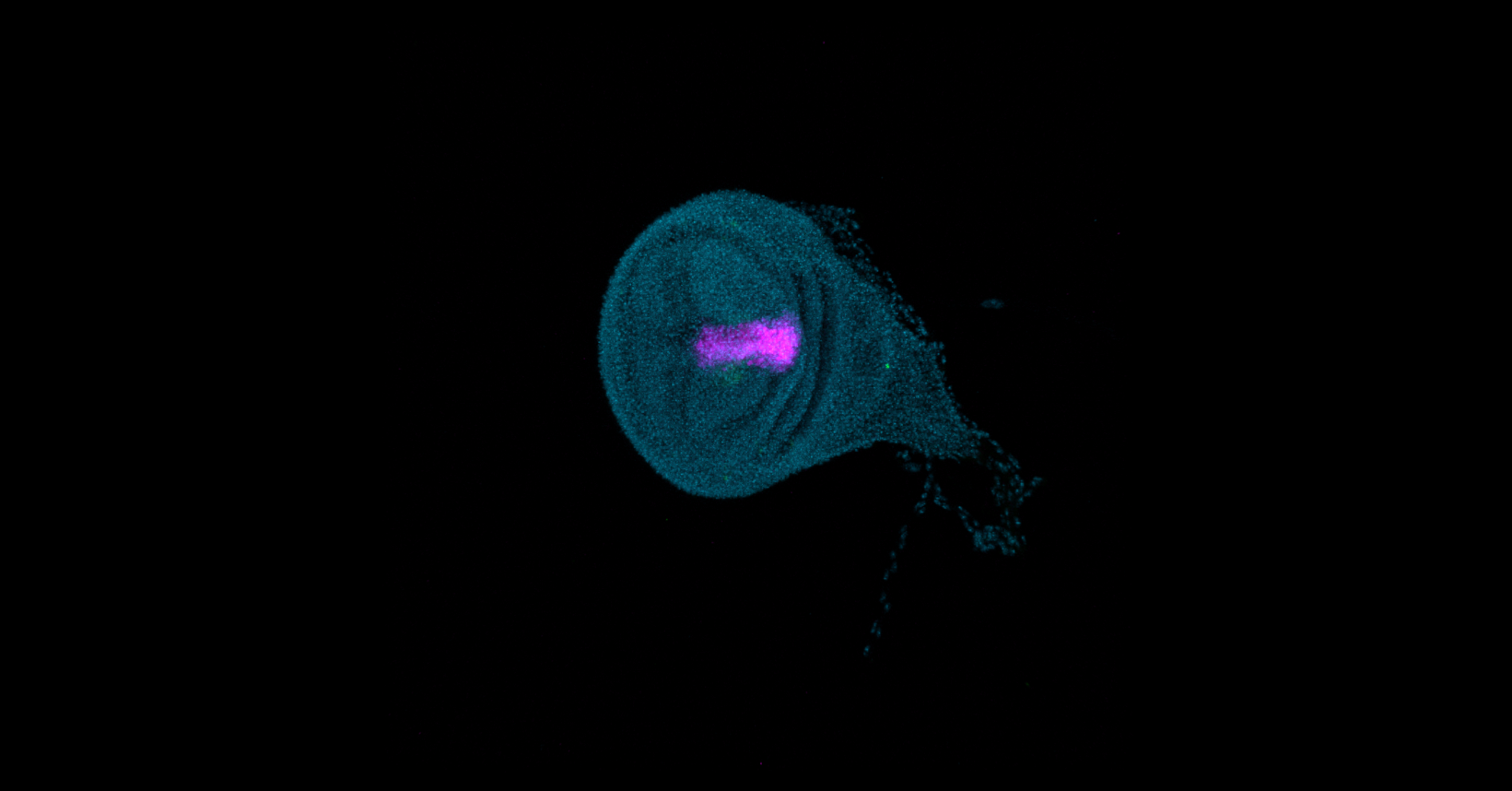

Supplement: Supplementary file 10 — Source data Fig. 8 [file 44319_2026_778_MOESM10_ESM.zip › Figure 8/8L/Fig.8L.tif]

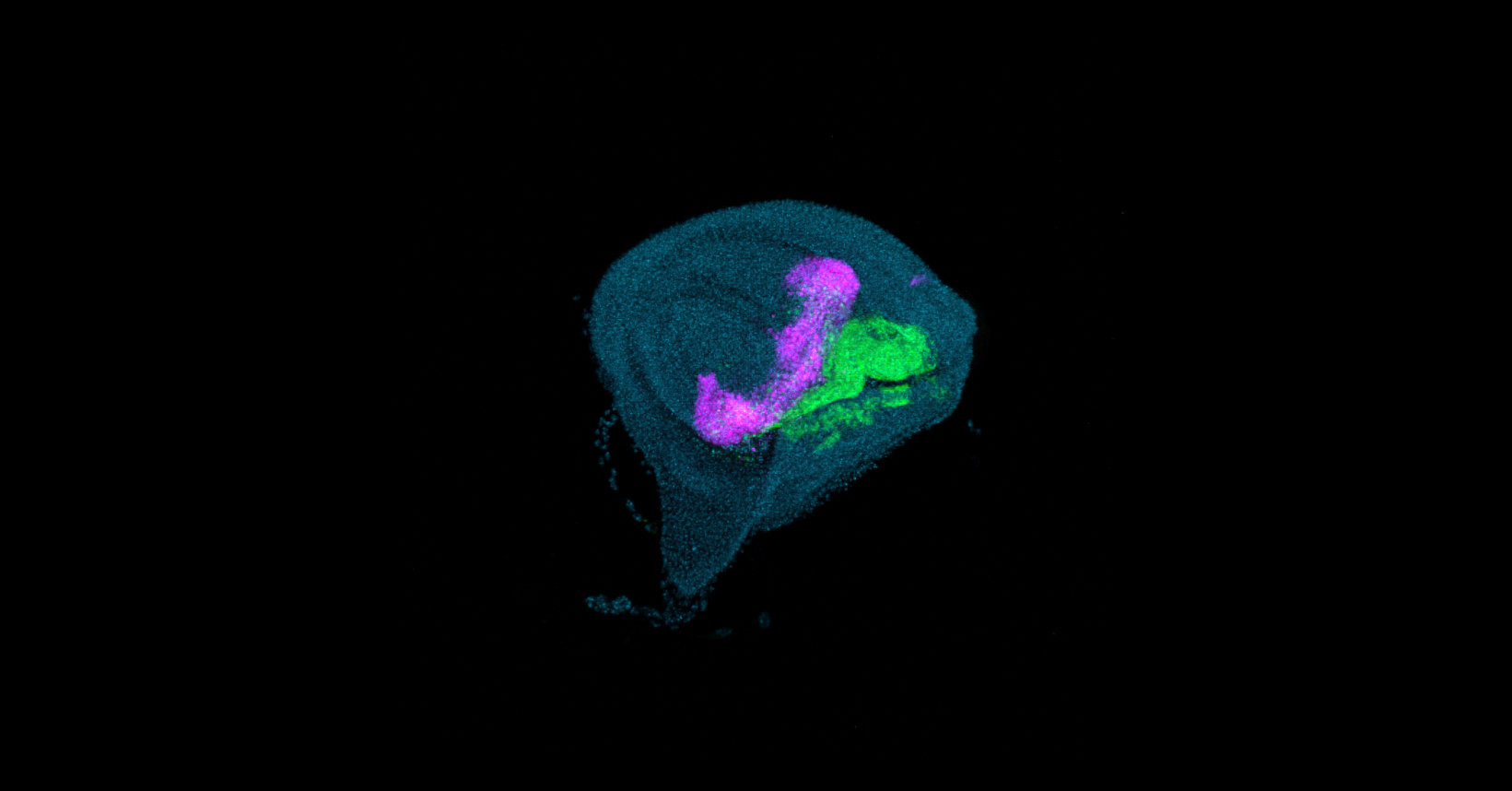

Supplement: Supplementary file 10 — Source data Fig. 8 [file 44319_2026_778_MOESM10_ESM.zip › Figure 8/8K/Fig.8K.tif]
