# Supplementary material for: The Hippo tumor suppressor pathway triggers non-cell autonomous tumorigenesis in Drosophila
Source: EMBO Rep. 2026 May 1;27(11):2915–46. doi: 10.1038/s44319-026-00778-5 (PMC13261145; doi:10.1038/s44319-026-00778-5)
Supplement: Supplementary file 11 — Expanded View Figures [file 44319_2026_778_MOESM11_ESM.pdf]

Expanded View Figures

Figure EV1. The mTOR pathway is activated in a non-cell-autonomous manner accompanied by PH3 upregulation.

(**A**) The schematic representation illustrates the G-TRACE technique. Enhancer activity, indicated by real-time expression, is visualized through RFP expression facilitated by Gal4 binding to UAS. Conversely, upon *Gal4* expression, a ubiquitous promoter persistently induces GFP (lineage expression) in these cells via flippase-dependent excision of a STOP cassette. (**B**, **C**) Confocal images depict wing discs containing either wild-type or *strip*-knockdown cells, marked with G-TRACE, demonstrating the lineage of Gal4 (green) through GFP expression and active Gal4 through RFP expression (magenta), and stained with anti-phospho-S6 (yellow). RFP (magenta) delineates the current expression pattern, whereas GFP indicates the lineage of *en-Gal4* in the wing disc. pS6 cell clusters are outlined by white dashed lines. (**D**) Incidence of predominantly GFP-negative pS6 clusters. Sample size: $n = 32$ (*en > RFP, strip RNAi, FLP, ubi-FRT-STOP-FRT-GFP*). (**E**) Quantification of the area of loss of differentiated characteristics (% of GFP^+ area without RFP/entire GFP-positive area) in wing discs containing either wild-type or *strip*-knockdown cells with G-TRACE. ****$P < 0.0001$; Welch's *t* test. Sample size and *P* value: $n = 28$ (*en > RFP, FLP, ubi-FRT-STOP-FRT-GFP*), $n = 31$, $P = 4.57 \times 10^{-9}$ (*en > RFP, strip* RNAi, *FLP, ubi-FRT-STOP-FRT-GFP*). (**F–H**) High-magnification image (**F**) indicated in the white box in (**C**). (**G**, **H**) Vertical sections at a site indicated by a dashed line in (**F**). GFP (green) is outlined by white dashed lines. (**I**, **J**) Confocal images showing wing discs containing either wild-type or *strip*-knockdown cells marked with RFP expression (magenta) and stained with anti-phospho-S6 (yellow) and anti-PH3 (green). RFP (magenta) delineates the expression pattern of *en-Gal4* in the wing disc. pS6 cell clusters are outlined by white dashed lines. (**K**) Quantification of the number of PH3 (the number of PH3/RFP-positive, RFP-negative, or pS6 cell-cluster area [pixels]) in wing discs containing either wild-type or *strip*-knockdown cells. Normalized values were scaled by a factor of 100. ****$P < 0.0001$; one-way ANOVA with Dunnett's multiple comparison test. Sample size and *P* value: $n = 22$, $P = 6.96 \times 10^{-12}$ (RFP^+ area in *en > RFP*), $n = 22$, $P < 1 \times 10^{-15}$ (RFP^- area in *en > RFP*), $n = 29$, $P = 3.93 \times 10^{-12}$ (RFP^+ area in *en>strip* RNAi), $n = 29$, $P < 1 \times 10^{-15}$ (RFP^- area in *en>strip* RNAi), $n = 29$ ($pS6^+$ area in *en>strip* RNAi). Numbers indicate the number of biological replicates (**D**); dots represent biological replicates (**E**, **K**); error bars indicate SEM.

© The Author(s)

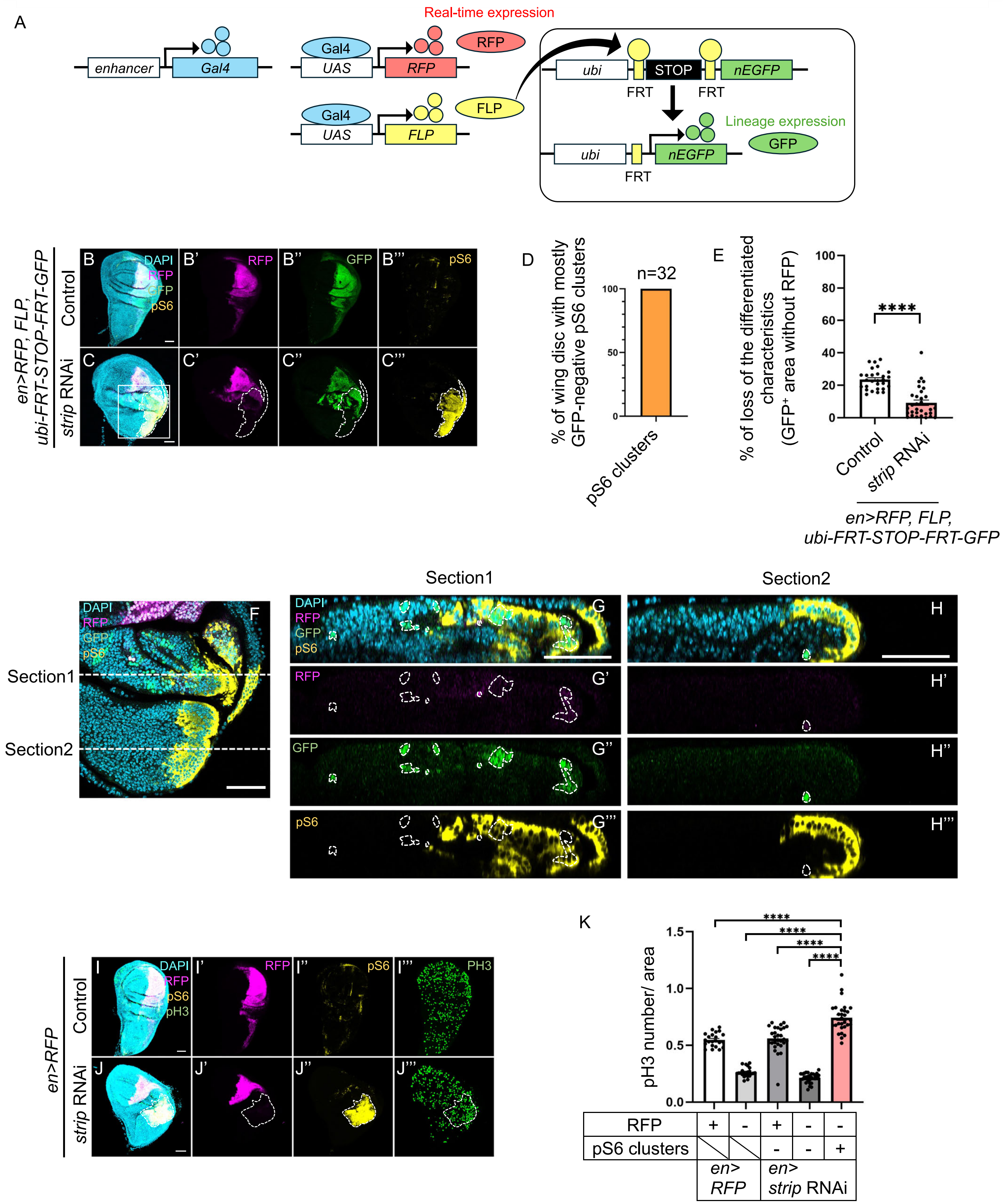
A
Real-time expression
enhancer
Gal4
Gal4
UAS
RFP
RFP
Gal4
UAS
FLP
FLP
ubi
STOP
nEGFP
FRT
FRT
Lineage expression
ubi
nEGFP
GFP
FRT
en>RFP, FLP, ubi-FRT-STOP-FRT-GFP
Control
strip RNAi
B DAPI RFP GFP pS6
B' RFP
B'' GFP
B''' pS6
C
C'
C''
C'''
D
% of wing disc with mostly GFP-negative pS6 clusters
n=32
pS6 clusters
E
% of loss of the differentiated characteristics (GFP+ area without RFP)
****
Control
strip RNAi
en>RFP, FLP, ubi-FRT-STOP-FRT-GFP
Section1
Section2
DAPI RFP GFP pS6
F
G
G'
G''
G'''
H
H'
H''
H'''
en>RFP
Control
strip RNAi
I DAPI RFP pS6 pH3
I' RFP
I'' pS6
I''' PH3
J
J'
J''
J'''
K
pH3 number/ area
RFP
pS6 clusters
en> RFP
en> strip RNAi

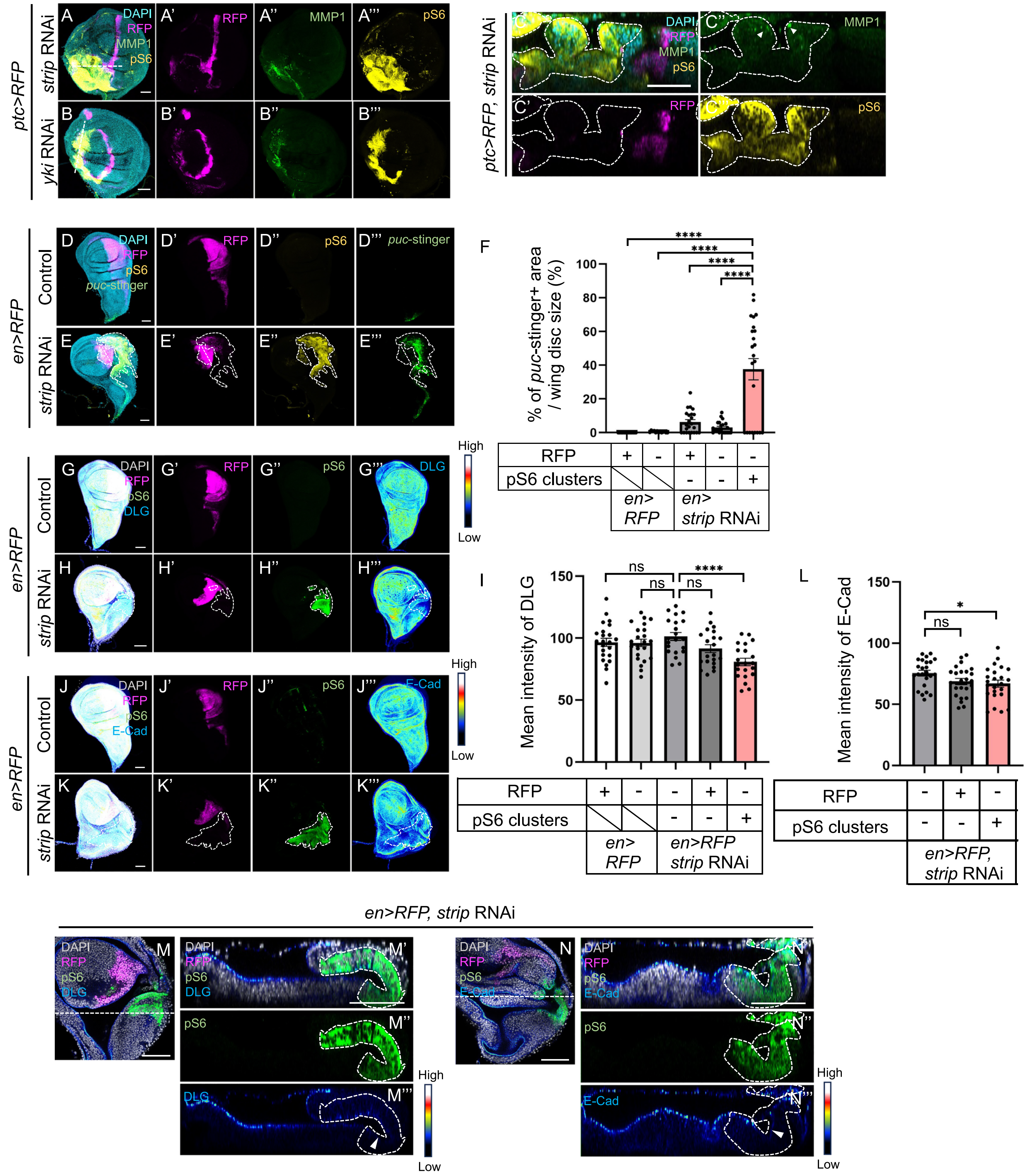
ptc>RFP
strip RNAi
yki RNAi
ptc>RFP, strip RNAi
DAPI RFP MMP1 pS6
en>RFP
Control
strip RNAi
DAPI RFP pS6 puc-stinger
% of puc-stinger+ area / wing disc size (%)
RFP
pS6 clusters
en> RFP
en> strip RNAi
DAPI RFP pS6 DLG
DAPI RFP pS6 E-Cad
High
Low
Mean intensity of DLG
Mean intensity of E-Cad
ns
****
*
en>RFP strip RNAi
en>RFP, strip RNAi

© The Author(s)

◀ **Figure EV2. MMP1 and the JNK pathway are upregulated and cell polarity proteins are downregulated in mTOR-activated cell clusters.**

(**A–C**) Confocal microscopy images depicting wing discs containing either *strip* or *yki*-knocked down cells, marked by RFP expression (magenta), and stained with anti-phospho-S6 (yellow) and anti-MMP1 (green). (**C**) Vertical sections at the site indicated by the dashed line in (**A**). The white arrowheads indicate MMP1-expressing cells within the pS6 cell clusters, which are delineated by white dashed lines. RFP (magenta) highlights the expression pattern of *ptc-Gal4* in wing discs. (**D**, **E**) Confocal images illustrating wing discs with either wild-type or *strip*-knockdown cells marked by RFP expression (magenta), stained with anti-phospho-S6 (yellow), and labeled with *puc-stinger* to indicate JNK pathway activity (green). RFP (magenta) indicates the expression pattern of *en-Gal4* on wing discs. The pS6 cell clusters are outlined by white dashed lines. (**F**) Quantification of the *puc-stinger*-labeled area (% of *puc-stinger*-labeled area/RFP-positive, RFP-negative, or pS6 cell-cluster area) in wing discs containing either wild-type or *strip*-knockdown cells. ****$P < 0.0001$; one-way ANOVA with Dunnett's multiple comparison test. Sample in and strip value: $n = 13$, $P = 1.88 \times 10^{-9}$ (RFP^+ area in *en > RFP*), $n = 13$, $P = 2.82 \times 10^{-9}$ (RFP^- area in *en > RFP*), $n = 23$, $P = 3.69 \times 10^{-9}$ (RFP^+ area in *en>strip* RNAi), $n = 23$, $P = 1.30 \times 10^{-10}$ (RFP^- area in *en>strip* RNAi), $n = 23$ ($pS6^+$ area in *en>strip* RNAi). (**G**, **H**) Confocal images showing wing discs with either wild-type or *strip*-knockdown cells, marked by RFP expression (magenta), and stained with anti-phospho-S6 (green) and anti-DLG (rainbow LUT). RFP (magenta) indicates the expression pattern of *en-Gal4* on wing discs. The pS6 cell clusters are outlined by white dashed lines. (**I**) Quantification of DLG intensity (DLG intensity/disc area [pixels]) in wing discs bearing wild-type or *strip*-knockdown cells. ns, not significant; ****$P < 0.0001$; one-way ANOVA with Dunnett's multiple comparison test. Sample size and P value: $n = 23$, $P = 6.49 \times 10^{-1}$ (RFP^+ area in *en > RFP*), $n = 23$, $P = 5.71 \times 10^{-1}$ (RFP^- area in *en > RFP*), $n = 21$ (RFP^- area in *en>strip* RNAi), $n = 21$, $P = 1.00 \times 10^{-1}$ (RFP^+ area in *en>strip* RNAi), $n = 21$, $P = 4.60 \times 10^{-5}$ ($pS6^+$ area in *en>strip* RNAi). (**J**, **K**) Confocal images showing wing discs with either wild-type or *strip*-knockdown cells, marked by RFP expression (magenta), and stained with anti-phospho-S6 (green) and anti-E-Cad (rainbow LUT). RFP (magenta) indicates the expression pattern of *en-Gal4* on wing discs. The pS6 cell clusters are outlined by white dashed lines. (**L**) Quantification of E-Cad intensity (E-Cad intensity/disc area [pixels]) in wing discs bearing wild-type or *strip*-knockdown cells. ns, not significant; *$P < 0.05$; one-way ANOVA with Dunnett's multiple comparison test. Sample size and P value: $n = 24$ (RFP^- area in *en>strip* RNAi), $n = 24$, $P = 1.13 \times 10^{-1}$ (RFP^+ area in *en>strip* RNAi), $n = 24$, $P = 3.83 \times 10^{-2}$ ($pS6^+$ area in *en>strip* RNAi). (**M**, **N**) High-magnification images showing wing discs with *strip*-knocked down cells stained with anti-phospho-S6 (green), DLG (rainbow LUT), or E-Cad (rainbow LUT). (**M′–M‴**, **N–N‴**) Vertical sections at a site indicated by a dashed line in (**M**, **N**). White arrowheads indicate DLG- or E-Cad-depleted spots. Scale bars represent 50 μm in (**A–E**, **G**, **H**, **J**, **K**, **M**, **N**). Dots represent biological replicates (**F**, **I**, **L**); error bars indicate SEM.

 © The Author(s)

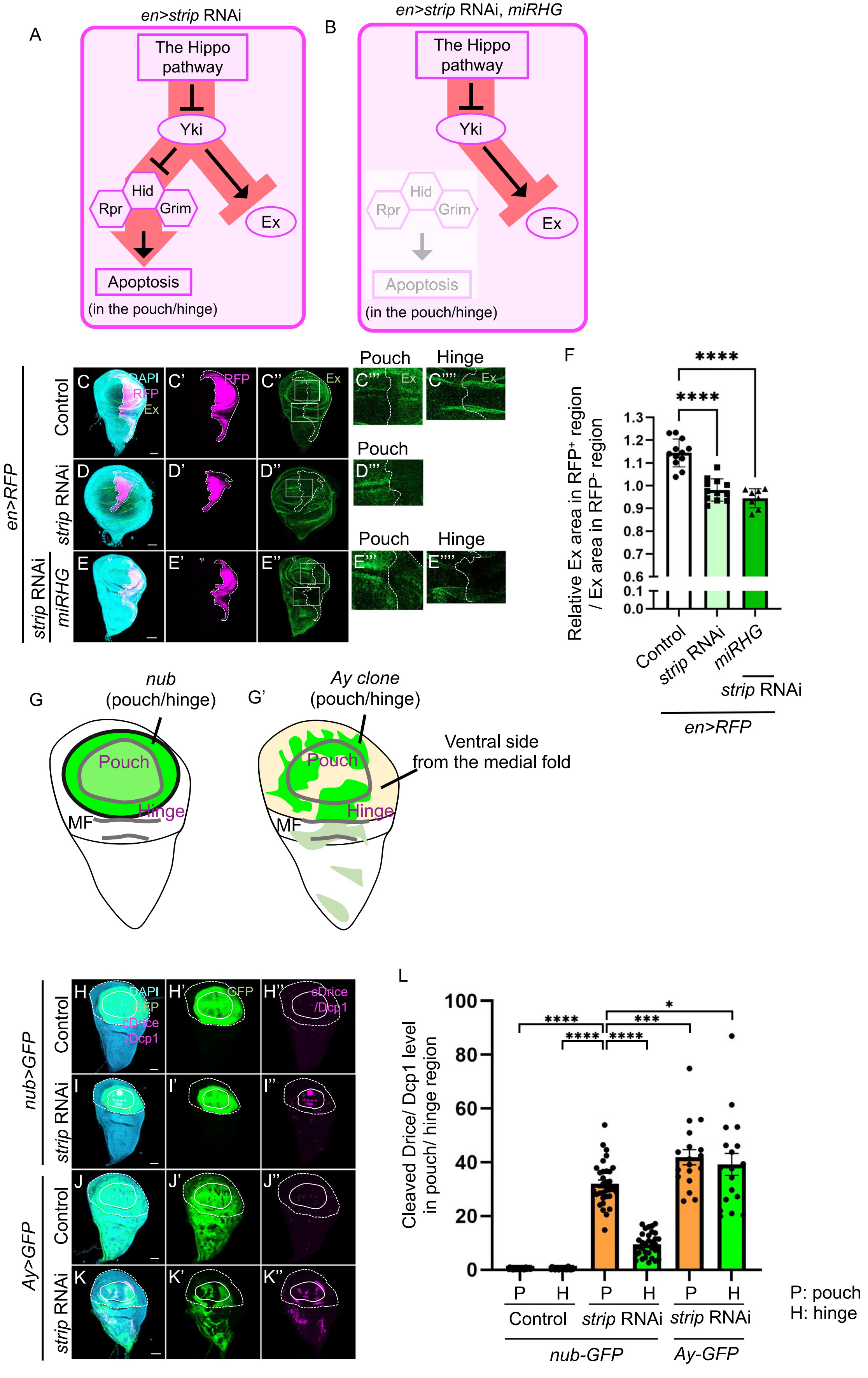
A
en>strip RNAi
The Hippo pathway
Yki
Hid
Rpr
Grim
Ex
Apoptosis
(in the pouch/hinge)
B
en>strip RNAi, miRHG
The Hippo pathway
Yki
Hid
Rpr
Grim
Ex
Apoptosis
(in the pouch/hinge)
en>RFP
Control
strip RNAi
strip RNAi miRHG
DAPI RFP Ex
Pouch
Hinge
F
Relative Ex area in RFP⁺ region / Ex area in RFP⁻ region
****
****
Control
strip RNAi
miRHG
strip RNAi
en>RFP
G
nub (pouch/hinge)
Pouch
Hinge
MF
G'
Ay clone (pouch/hinge)
Ventral side from the medial fold
nub>GFP
Ay>GFP
DAPI GFP cDrice /Dcp1
L
Cleaved Drice/ Dcp1 level in pouch/ hinge region
P H P H P H
Control strip RNAi strip RNAi
nub-GFP Ay-GFP
P: pouch
H: hinge

Figure EV3. The Hippo signaling pathway is activated by *strip* RNAi in the pouch/hinge region.

(**A**, **B**) The schematic representation illustrates a signaling pathway activated by Hippo through *strip* RNAi, with or without the knockdown of *rpr, hid, and grim* (*miRHG*). *miRHG* inhibits apoptosis in the pouch and hinge regions, facilitating the analysis of these areas under Hippo activation. (**C–E**) Confocal images displaying wing discs containing either wild-type or *strip*-knockdown cells, with or without *miRHG*, marked by RFP expression (magenta) and stained with anti-Expanded (green). RFP (magenta), delineated by white dashed lines, indicates the expression pattern of *en-Gal4* in the wing disc. High-magnification images (C‴–E‴) indicated in the white boxes in (C″–E″). (**F**) Quantification of the relative Expanded level (Expanded positive area in RFP^+ region/Expanded positive area in RFP^- region) in the wild-type or *strip*-knockdown wing disc. ****$P < 0.0001$; one-way ANOVA with Dunnett's multiple comparison test. Sample size and *P* value: $n = 12$ (*en > RFP*), $n = 12$, $P = 4.63 \times 10^{-8}$ (*en>strip* RNAi), $n = 8$, $P = 7.28 \times 10^{-9}$ (*en>strip* RNAi, *miRHG*). (**G**) The schematic depictions indicate the *nub-Gal4* active area in (**G**, green) and *Ay-Gal4* active clone in (**G′**, green). The hinge region on the ventral side from the medial fold (MF) in (**G′**, yellow). (**H–K**) Confocal images showing wing discs containing either wild-type or *strip*-knockdown cells marked with GFP expression and stained with anti-cleaved Drice/Dcp1 (magenta). GFP (green) indicates the expression pattern of *nub-Gal4* or *Ay-Gal4* in the wing disc. Solid lines and dashed lines outline the pouch region and hinge region on the ventral side of the medial fold (green clone in yellow area in **G′**), respectively. The female animals of these strains were selected for the experiment. (**L**) Quantification of the size of the cleaved Drice/Dcp1-positive region (% of anti-cleaved Drice/Dcp1-positive area/GFP-positive area) in the wing disc containing either wild-type or *strip*-knockdown cells. Quantification was conducted in the GFP-positive area in the pouch (P) or hinge region on the ventral side from the medial fold (H). *$P < 0.05$; ***$P < 0.001$; ****$P < 0.0001$; one-way ANOVA with Dunnett's multiple comparison test. Sample size and *P* value: $n = 25$, $P < 1 \times 10^{-15}$ (pouch in *nub > GFP*), $n = 25$, $P < 1 \times 10^{-15}$ (hinge in *nub > GFP*), $n = 32$ (pouch in *nub>strip* RNAi), $n = 32$, $P < 1 \times 10^{-15}$ (hinge in *nub>strip* RNAi), $n = 18$, $P = 5.26 \times 10^{-4}$ (pouch in *Ay > GFP*), $n = 18$, $P = 1.93 \times 10^{-2}$ (hinge in *Ay>strip* RNAi). Scale bars represent 50 μm in (**C–E**, **H–K**). Dots represent biological replicates (**F**, **L**); error bars indicate SEM.

 © The Author(s)

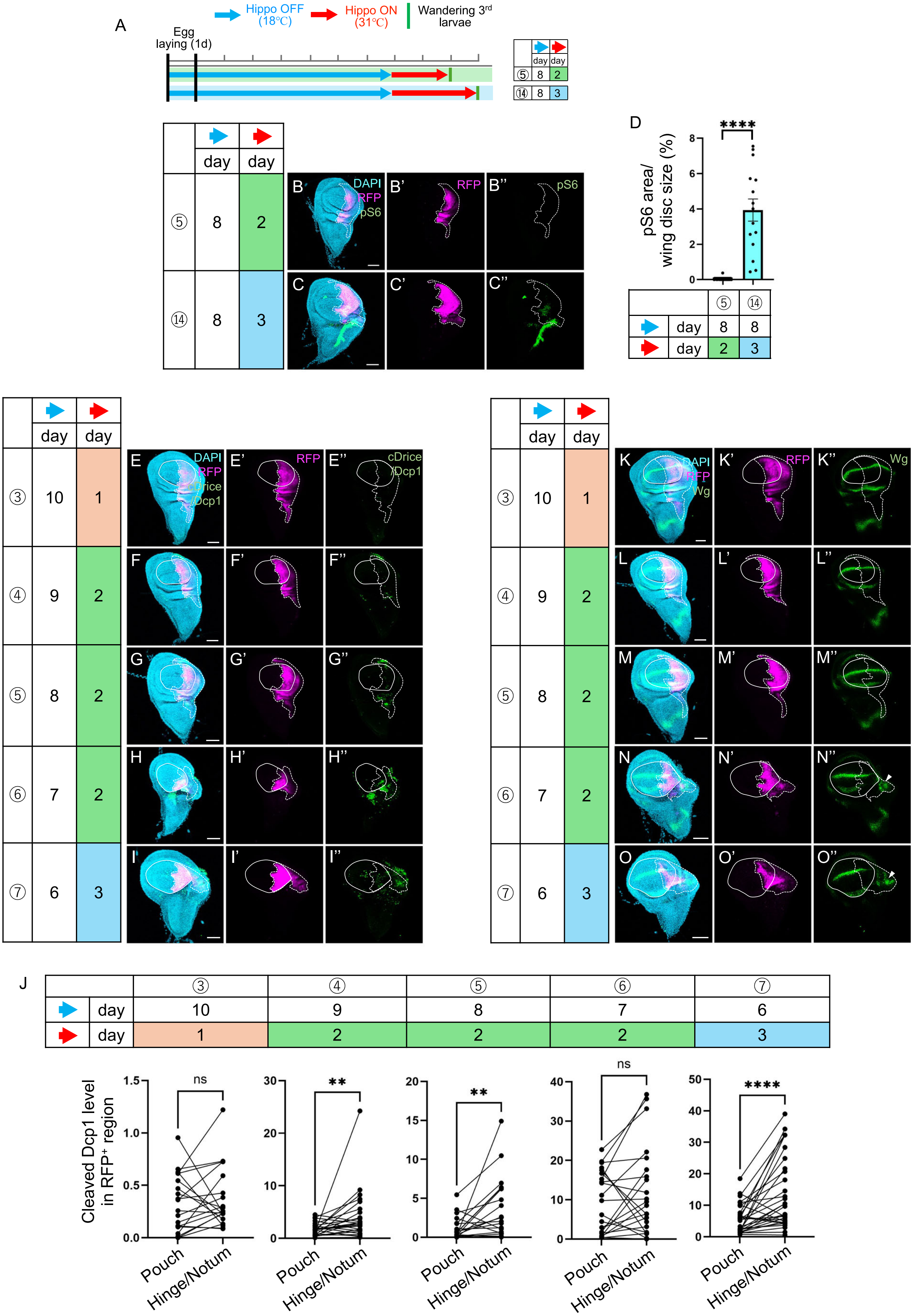
A
Hippo OFF (18℃)
Hippo ON (31℃)
Wandering 3rd larvae
Egg laying (1d)
D
pS6 area/ wing disc size (%)
****
J
Cleaved Dcp1 level in RFP⁺ region
ns
**
**
ns
****
Pouch
Hinge/Notum
DAPI
RFP
pS6
cDrice
Dcp1
Wg

Figure EV4. The activation of Dronc and ectopic expression of Wg are predominantly observed in the hinge and ventral notum regions.

(**A**) The schematic representation illustrates the temporal schedule for heat shock in the context of *strip* knockdown. Blue and red arrows denote the time post-egg laying at 18 °C (Hippo OFF) and the day of heat shock at 31 °C (Hippo ON), respectively. (**B**, **C**) Confocal images displaying wing discs containing *strip*-knockdown cells, under $Gal80^{ts}$ control, marked by RFP expression (magenta) and stained with anti-phospho-S6 (green). The RFP (magenta), delineated by white dashed lines, indicates the expression pattern of *en-Gal4* within the wing disc. (**D**) Quantification of the phospho-S6 positive region size (% of phospho-S6 positive area/disc area) in wing discs with *strip*-knockdown cells, whose induction was controlled by *Gal80ts*. ****$P < 0.0001$; Welch's *t* test. Sample size and *P* value: $n = 14$ (⑤AEL: HS = 8:2), $n = 15$, $P = 2.10 \times 10^{-5}$ (⑭AEL: HS = 8:3). (**E–I**) Confocal images depicting wing discs with *strip*-knockdown cells, regulated by $Gal80^{ts}$, marked by RFP expression (magenta) and stained with anti-cleaved Drice/Dcp1 (green). The RFP (magenta), outlined by white dashed lines, marks the *en-Gal4* expression pattern in the wing disc. Solid lines demarcate the pouch region. (**J**) A comparison of quantification data for the cleaved Drice/Dcp1-positive region (% of anti-cleaved Drice/Dcp1-positive area relative to RFP-positive area) between the pouch and hinge/ventral notum regions in wing discs containing *strip*-knockdown cells under $Gal80^{ts}$ control. **$P < 0.01$; ****$P < 0.0001$; Wilcoxon matched-pairs signed rank test. Sample size and *P* value: $n = 20$, $P = 3.49 \times 10^{-1}$ (③AEL: HS = 10:1), $n = 26$, $P = 5.1 \times 10^{-3}$ (④AEL: HS = 9:2), $n = 21$, $P = 4.88 \times 10^{-3}$ (⑤AEL: HS = 8:2), $n = 22$, $P = 1.29 \times 10^{-1}$ (⑥AEL: HS = 7:2), $n = 31$, $P = 5.61 \times 10^{-5}$ (⑦AEL: HS = 6:3). (**K–O**) Confocal images illustrating wing discs with *strip*-knockdown cells, regulated by $Gal80^{ts}$, marked by RFP expression (magenta) and stained with anti-Wg (green). The RFP (magenta), outlined by white dashed lines, indicates the *en-Gal4* expression pattern in the wing disc. Solid lines demarcate the pouch region. Scale bars represent 50 μm in (**B**, **C**, **E–I**, **K–O**). Dots represent biological replicates (**D**, **J**); error bars indicate SEM.

 © The Author(s)

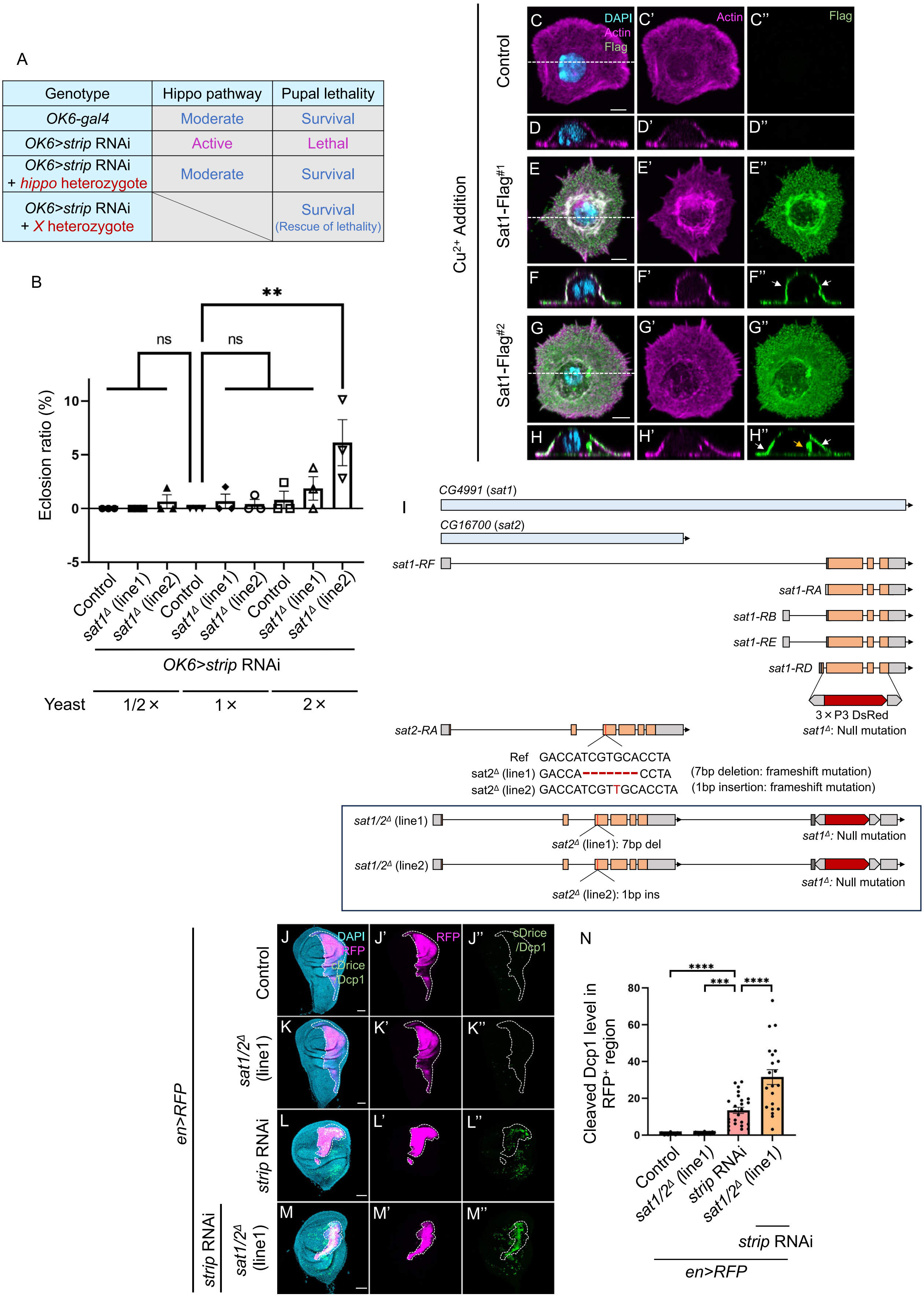
A
Genotype
Hippo pathway
Pupal lethality
OK6-gal4
Moderate
Survival
OK6>strip RNAi
Active
Lethal
OK6>strip RNAi + hippo heterozygote
Moderate
Survival
OK6>strip RNAi + X heterozygote
Survival (Rescue of lethality)
B
**
ns
ns
Eclosion ratio (%)
10
5
0
-5
Control
sat1Δ (line1)
sat1Δ (line2)
OK6>strip RNAi
Yeast
1/2 ×
1 ×
2 ×
Control
Cu2+ Addition
Sat1-Flag#1
Sat1-Flag#2
C
C'
C''
DAPI
Actin
Flag
D
D'
D''
E
E'
E''
F
F'
F''
G
G'
G''
H
H'
H''
I
CG4991 (sat1)
CG16700 (sat2)
sat1-RF
sat1-RA
sat1-RB
sat1-RE
sat1-RD
3 × P3 DsRed
sat1Δ: Null mutation
sat2-RA
Ref GACCATCGTGCACCTA
sat2Δ (line1) GACCA-------CCTA
sat2Δ (line2) GACCATCGTTGCACCTA
(7bp deletion: frameshift mutation)
(1bp insertion: frameshift mutation)
sat1/2Δ (line1)
sat2Δ (line1): 7bp del
sat1Δ: Null mutation
sat1/2Δ (line2)
sat2Δ (line2): 1bp ins
sat1Δ: Null mutation
en>RFP
Control
sat1/2Δ (line1)
strip RNAi
strip RNAi sat1/2Δ (line1)
J
J'
J''
DAPI
RFP
cDrice /Dcp1
K
K'
K''
L
L'
L''
M
M'
M''
N
Cleaved Dcp1 level in RFP+ region
80
60
40
20
0
****
***
****
Control
sat1/2Δ (line1)
strip RNAi
sat1/2Δ (line1)
strip RNAi
en>RFP

© The Author(s)

◀ **Figure EV5. Genomic deficiency screening identifies *sat1/2* and Sat1/2 functions to suppress apoptosis in cells with an activated Hippo pathway.**

(**A**) The table presents data on Hippo activity and pupal lethality in specimens possessing either wild-type or *strip*-knockdown cells, with or without different gene background mutations. The *strip* knockdown was executed using the *OK6-Gal4* driver for genomic deficiency screening. *X* genes are hypothesized to be genes whose deletion can mitigate pupal lethality induced by Hippo activation. (**B**) The eclosion ratio (percentage of adult flies relative to the number of pupae) is quantified in individuals with wild-type or *strip*-knockdown cells, with or without the *sat1* background mutation. These individuals were provided with diets containing different yeast concentrations (normal, 50%, or 200% yeast concentration). ns, not significant; $^{**}P < 0.01$; one-way ANOVA with Dunnett's multiple comparison test. Sample size and *P* value: $n = 3$, $P = 1.00$ (*OK6>strip* RNAi in 50% yeast), $n = 3$, $P = 1.00$ (*sat1* mutant line1; *OK6>strip* RNAi in 50% yeast), $n = 3$, $P = 9.97 \times 10^{-1}$ (*sat1* mutant line2; *OK6>strip* RNAi in 50% yeast), $n = 3$ (*OK6>strip* RNAi in 100% yeast), $n = 3$, $P = 9.96 \times 10^{-1}$ (*sat1* mutant line1; *OK6>strip* RNAi in 100% yeast), $n = 3$, $P = 1.00$ (*sat1* mutant line2; *OK6>strip* RNAi in 100% yeast), $n = 3$, $P = 9.89 \times 10^{-1}$ (*OK6>strip* RNAi in 200% yeast), $n = 3$, $P = 5.95 \times 10^{-1}$ (*sat1* mutant line1; *OK6>strip* RNAi in 200% yeast), $n = 3$, $P = 1.07 \times 10^{-3}$ (*sat1* mutant line2; *OK6>strip* RNAi in 200% yeast). (**C–H**) Confocal microscopy images depict S2 cells expressing a Flag-tagged Sat1 with a copper-inducible promoter. The addition of Cu^{2+} activates the copper-inducible promoter, leading to the overexpression of Flag-tagged Sat1. Sat1-Flag#1 and Sat1-Flag#2 refer to S2 cells (line1) and (line2) containing *pMT-Flag-sat1-puro*, respectively. Cell outlines are stained with TRITC-phalloidin, and Sat1 expression is indicated by anti-Flag staining (green). (**D**, **F**, **H**) Vertical sections at a site indicated by a dashed line in (**C**, **E**, **G**). The white arrows indicate Sat1 localization at the plasma membrane, while the yellow arrow indicates intracellular localization of Sat1. Scale bars represent 5 µm in (**C**, **E**, **G**). (**I**) Schematic illustrations depict the *CG4991* (*sat1*) and *CG16700* (*sat2*) genes and the mutated site of *sat1/2*. The coding region of the *sat1* gene was replaced by the marker gene *3xP3 DsRed*. The coding region of the *sat2* gene was altered with a deletion (line 1) or an insertion (line 2). *sat1/2* double mutant bears a *sat1* null mutation combined with a deletion or insertion mutation in *sat2*. (**J–M**) Confocal images displaying wing discs containing wild-type or *strip*-knockdown cells, with or without the *sat1/2* background mutations, marked with RFP expression (magenta) and stained with anti-Drice/Dcp1 (green). RFP (magenta) marks the expression pattern of *en-Gal4* in the wing disc. The male animals of these strains were selected for the experiment, and the *sat1/2* background mutations were hemizygous. (**N**) The size of the cleaved Drice/Dcp1-positive region is quantified (% of anti-cleaved Drice/Dcp1-positive area/RFP-positive area) in the wing disc containing wild-type or *strip*-knockdown cells, with or without the *sat1/2* background mutations. $^{***}P < 0.001$; $^{****}P < 0.0001$; one-way ANOVA with Dunnett's multiple comparison test. Sample size and *P* value: $n = 23$, $P = 8.43 \times 10^{-5}$ (*en > RFP*), $n = 22$, $P = 1.40 \times 10^{-4}$ (*sat1/2* mutant line1, *en > RFP*), $n = 25$ (*en>strip* RNAi), $n = 21$, $P = 6.04 \times 10^{-8}$ (*sat1/2* mutant line1, *en>strip* RNAi). Scale bars represent 50 µm in (**J–M**). Dots represent technical (**B**) or biological (**N**) replicates; error bars indicate SEM.

 © The Author(s)
