# Supplementary material for: The Hippo tumor suppressor pathway triggers non-cell autonomous tumorigenesis in Drosophila
Source: EMBO Rep. 2026 May 1;27(11):2915–46. doi: 10.1038/s44319-026-00778-5 (PMC13261145; doi:10.1038/s44319-026-00778-5)
Supplement: Supplementary file 2 — Peer Review File [file 44319_2026_778_MOESM2_ESM.pdf]

EMBO reports

The Hippo tumor suppressor pathway triggers non-cell autonomous tumorigenesis in Drosophila

Takahiro Chihara, Masayuki Miura, Chisako Sakuma, Daichi Honda, Toshinori Ando, Misako Okumura, Fumiaki Obata, and Ayano Oi

Corresponding author(s): Takahiro Chihara (tchihara@hiroshima-u.ac.jp)

Review Timeline:

Submission Date:	4th Jul 25
Editorial Decision:	4th Aug 25
Revision Received:	12th Dec 25
Editorial Decision:	3rd Feb 26
Revision Received:	2nd Mar 26
Accepted:	31st Mar 26

Editor: Achim Breiling

Transaction Report:

(Note: With the exception of the correction of typographical or spelling errors that could be a source of ambiguity, letters and reports are not edited. Depending on transfer agreements, referee reports obtained elsewhere may or may not be included in this compilation. Referee reports are anonymous unless the Referee chooses to sign their reports.)

1st Editorial Decision 4th Aug 2025

Dear Prof. Chihara,

Thank you for the transfer of your manuscript to EMBO reports. I have now received reports from the three referees that were asked to evaluate your study, which can be found at the end of this email. As you will see, the referees think that these findings are of high interest. However, they have several comments, concerns, and suggestions, indicating that a major revision of the manuscript is necessary to allow publication of the study in EMBO reports. As the reports are below, and all the referee concerns need to be addressed, I will not detail them here.

Given the constructive referee comments, I would thus like to invite you to revise your manuscript with the understanding that the concerns of the referees must be addressed in the revised manuscript and/or in a detailed point-by-point response. Acceptance of your manuscript will depend on a positive outcome of a second round of review. It is EMBO reports policy to allow a single round of revision only and acceptance of the manuscript will therefore depend on the completeness of your responses included in the next, final version of the manuscript.

Revised manuscripts should be submitted within three months of a request for revision. Please contact me to discuss the revision (also by video chat) if you have questions or comments regarding the revision, or should you need additional time.

When submitting your revised manuscript, please also carefully review the instructions that follow below.

PLEASE NOTE THAT upon resubmission revised manuscripts are subjected to an initial quality control prior to exposition to re-review. Upon failure in the initial quality control, the manuscripts are sent back to the authors, which may lead to delays. Frequent reasons for such a failure are the lack of the data availability section (please see below) and the presence of statistics based on n=2 (the authors are then asked to present scatter plots or provide more data points).

When submitting your revised manuscript, we will require:

1) a .docx formatted version of the final manuscript text (including legends for main figures, EV figures and tables), but without the figures included. Figure legends should be compiled at the end of the manuscript text.

2) individual production quality figure files as .eps, .tif, .jpg (one file per figure), of main figures and EV figures. Please upload these as separate, individual files upon re-submission.

The Expanded View format, which will be displayed in the main HTML of the paper in a collapsible format, has replaced the Supplementary information. You can submit up to 5 images as Expanded View. Please follow the nomenclature Figure EV1, Figure EV2 etc. The figure legend for these should be included in the main manuscript document file in a section called Expanded View Figure Legends after the main Figure Legends section. Additional Supplementary material should be supplied as a single pdf file labeled Appendix. The Appendix should have page numbers and needs to include a table of content on the first page (with page numbers) and legends for all content. Please follow the nomenclature Appendix Figure Sx, Appendix Table Sx etc. throughout the text, and also label the figures and tables according to this nomenclature.

For more details, please refer to our guide to authors:
http://www.embopress.org/page/journal/14693178/authorguide#manuscriptpreparation

Please consult our guide for figure preparation:
http://wol-prod-cdn.literatumonline.com/pb-assets/embo-site/EMBOPress_Figure_Guidelines_061115-1561436025777.pdf

See also the guidelines for figure legend preparation:
https://www.embopress.org/page/journal/14693178/authorguide#figureformat

3) a .docx formatted letter INCLUDING the reviewers' reports and your detailed point-by-point responses to their comments. As part of the EMBO Press transparent editorial process, the point-by-point response is part of the Review Process File (RPF), which will be published alongside your paper.

4) a complete author checklist, which you can download from our author guidelines (https://www.embopress.org/page/journal/14693178/authorguide). Please insert page numbers in the checklist to indicate where the requested information can be found in the manuscript. The completed author checklist will also be part of the RPF.

Please also follow our guidelines for the use of living organisms, and the respective reporting guidelines:
http://www.embopress.org/page/journal/14693178/authorguide#livingorganisms

5) that primary datasets produced in this study (e.g. RNA-seq, ChIP-seq, structural and array data) are deposited in an appropriate public database. If no primary datasets have been deposited, please also state this in a dedicated section (e.g. 'No

primary datasets have been generated and deposited'), see below.

See also: http://embor.embopress.org/authorguide#datadeposition

Please remember to provide a reviewer password if the datasets are not yet public.

The accession numbers and database should be listed in a formal "Data Availability" section that follows the model below. This is now mandatory (like the COI statement). Please note that the Data Availability Section is restricted to new primary data that are part of this study. This section is mandatory. As indicated above, if no primary datasets have been deposited, please state this in this section

Data availability

The datasets produced in this study are available in the following databases:

- RNA-Seq data: Gene Expression Omnibus GSE46843 (https://www.ncbi.nlm.nih.gov/geo/query/acc.cgi?acc=GSE46843)
- [data type]: [name of the resource] [accession number/identifier/doi] ([URL or identifiers.org/DATABASE:ACCESSION])

*** Note - All links should resolve to a page where the data can be accessed. ***

Moreover, I have these editorial requests:

6) We now request the publication of original source data with the aim of making primary data more accessible and transparent to the reader. You will receive a separate email with instructions for providing source data with your revised manuscript, including information how to upload and organize the files.

7) Our journal encourages inclusion of *data citations in the reference list* to directly cite datasets that were re-used and obtained from public databases. Data citations in the article text are distinct from normal bibliographical citations and should directly link to the database records from which the data can be accessed. In the main text, data citations are formatted as follows: "Data ref: Smith et al, 2001" or "Data ref: NCBI Sequence Read Archive PRJNA342805, 2017". In the Reference list, data citations must be labeled with "[DATASET]". A data reference must provide the database name, accession number/identifiers and a resolvable link to the landing page from which the data can be accessed at the end of the reference. Further instructions are available at: http://www.embopress.org/page/journal/14693178/authorguide#referencesformat

8) Regarding data quantification and statistics, please make sure that the number "n" for how many independent experiments were performed, their nature (biological versus technical replicates), the bars and error bars (e.g. SEM, SD) and the test used to calculate p-values is indicated in the respective figure legends (also for EV and Appendix figures). Please also check that all the p-values are explained in the legend, and that these fit to those shown in the figure. Please provide statistical testing where applicable. Please avoid the phrase 'independent experiment', but clearly state if these were biological or technical replicates. Please also indicate (e.g. with n.s.) if testing was performed, but the differences are not significant. In case n=2, please show the data as separate datapoints without error bars and statistics. See also: http://www.embopress.org/page/journal/14693178/authorguide#statisticalanalysis

If n<5, please show single datapoints for diagrams.

9) Please add scale bars of similar style and thickness to all microscopic images, using clearly visible black or white bars (depending on the background). Please place these in the lower right corner of the images themselves. Please do not write on or near the bars in the image but define the size in the respective figure legend.

10) Please also note our reference format:
http://www.embopress.org/page/journal/14693178/authorguide#referencesformat

11) We updated our journal's competing interests policy in January 2022 and request authors to consider both actual and perceived competing interests. Please review the policy https://www.embopress.org/competing-interests and update your competing interests if necessary. Please name this section 'Disclosure and Competing Interests Statement' and put it after the Acknowledgements section.

12) We now use CRediT to specify the contributions of each author in the journal submission system. CRediT replaces the author contribution section. Please use the free text box to provide more detailed descriptions and do NOT provide your final manuscript text file with an author contributions section. See also our guide to authors:
https://www.embopress.org/page/journal/14693178/authorguide#authorshipguidelines

13) All Materials and Methods need to be described in the main text using our 'Structured Methods' format, which is required for all research articles. According to this format, the Methods section should include a Reagents and Tools Table (listing key

reagents, experimental models, software, and relevant equipment and including their sources and relevant identifiers), uploaded as separate file, and a Methods section in which we encourage the authors to describe their methods using a step-by-step protocol format with bullet points, to facilitate the adoption of the methodologies across labs. More information on how to adhere to this format as well as downloadable templates (.doc) for the Reagents and Tools Table can be found in our author guidelines (section 'Structured Methods'):

https://www.embopress.org/page/journal/14693178/authorguide#manuscriptpreparation

14) Please order the manuscript sections like this, using only these names:
Title page - Abstract - Keywords - Introduction - Results - Discussion - Methods - Data availability section - Acknowledgements (please put here all the funding information) - Disclosure and Competing Interests Statement - References - Figure legends - Expanded View Figure legends

15) Please make sure that all the funding information is also entered into the online submission system and that it is complete and similar to the one in the acknowledgement section of the manuscript text file.

We would also welcome the submission of cover suggestions, or motifs to be used by our Graphics Illustrator in designing a cover.

As part of the EMBO publication's Transparent Editorial Process, EMBO reports publishes online a Review Process File (RPF) to accompany accepted manuscripts. This File will be published in conjunction with your paper and will include the referee reports, your point-by-point response and all pertinent correspondence relating to the manuscript.

You are able to opt out of this by letting the editorial office know (emboreports@embo.org). If you do opt out, the Review Process File link will point to the following statement: "No Review Process File is available with this article, as the authors have chosen not to make the review process public in this case."

I look forward to seeing a revised form of your manuscript when it is ready.

Yours sincerely,

Achim Breiling
Senior Editor
EMBO Reports

Referee #1:

The Hippo signaling pathway is widely known for its role in restricting tissue growth and acting as a tumor suppressor. In canonical models, loss of Hippo activity leads to autonomous overgrowth via nuclear translocation of Yorkie/YAP and activation of growth-promoting genes. However, recent studies suggest a paradoxical role where Hippo activation itself may promote tumorigenesis under certain conditions. The underlying mechanisms of such non-canonical, pro-tumorigenic functions of Hippo signaling are poorly understood. The authors aim to investigate this possibility using Drosophila wing discs as a model system.

Specifically, they authors show that activation of Hippo signaling (via strip RNAi, yki RNAi, or wts overexpression) induces mTOR activation, MMP1 expression, and EdU incorporation in adjacent cells, consistent with non-cell-autonomous tumorigenesis. These effects occur outside the Hippo-activated zone, in a signaling niche. Using multiple Gal4 drivers and clonal analysis, the authors report that the tumor-inducing capacity of Hippo-activated cells is spatially restricted to the hinge and ventral notum regions of the wing disc. Furthermore, targeted knockdown or overexpression of rpr/hid/grim, bantam, atg8a, and cyclin E are necessary for inducing mTOR activation in neighboring cells. These genes likely act downstream or independently of Yki suppression to mediate apoptotic and stress signals that promote these non-cell-autonomous effects. Interestingly, the authors demonstrate that tumorigenesis is dependent on the initiator caspase Dronc (which itself is considered a tumor suppressor) and its adaptor Dark, but not on effector caspases. This observations reveals another non-apoptotic function of Dronc for compensatory proliferation. Wg and Spitz are required for tumor formation and are upregulated in the hinge/notum region in response to Hippo activation which suggests that they are key signaling ligands in this process. A very nice temporal analysis by the authors shows that Wg upregulation and Dronc activation precede mTOR activation in neighboring cells. This temporal order supports a causal relationship where Hippo signaling initiates Dronc/Wg activation, which then promotes non-cell autonomous overgrowth. However, ectopic activation of Dronc and Wg (via hid/rpr + p35 or Wg overexpression) is not sufficient to induce non-cell-autonomous mTOR activation, suggesting that additional signals from Hippo activated cells are required. Finally, the authors perform a genetic screen and identify Sat1 and Sat2 as novel, redundant amino acid transporters involved in tumorigenesis. Knockdown of sat1/2 reduces mTOR activity and cell growth both autonomously and non-autonomously. Also, the combined sat1/2 and spitz suppression has a synergistic effect.

Altoghter, this study reveals an unexpected tumor-promoting role for the Hippo pathway via a non-cell-autonomous mechanism mediated by Dronc, Wg/Spitz, and nutrient transporters and thereby challenges the dogma that Hippo activation acts purely tumor-suppressive. However, while Hippo signaling acts as a tumor suppressor cell-autonomously, in the context described here it acts as a tumor promoter in a non-cell autonomous manner. In the latter case, Hippo-activated cells act as "oncogenic niches", and shape the local tumor microenvironment through secretion of signaling factors and nutrient exchange. These findings expand the functional scope of Hippo signaling.

Overall, this work is very well worked out, the conclusions are supported by the data and the manuscript is usually well written. I only have a few comments to futher improve it.

1. While overexpression of hid/rpr + p35 (or Wg expression) is not sufficient to mimic Hippo induced tumorigenesis, can combined overexpression of hid/rpr + p35 (or Wg) with bantam and/or atg8a in hinge/notum region induce non-autonomous tumors?

2. The authors talk about tumorigenesis in the context of these hippo-induced overgrowths. However, can they clarify more specifically the cellular outcomes in recipient mTOR+ cells e.g., tumorigenic (neoplastic) vs hyperplastic?

3. The function of Sat1 and Sat2 is inferred based on homology and localization. However, functional transport assays were not provided by the authors. Can they provide more evidence that these proteins indeed act as aa transporters?

4. Overall the writing is clear and logical. However, some sections in the results (e.g., timing experiments or transporter functions) could benefit from more concise phrasing and improved figure referencing.

5. The reference list requires improvement, as the authors have not consistently cited the most relevant papers. For example, when discussing bantam, the authors should include Brennecke et al. (2003), Cell. Several other instances suggest that key publications have been overlooked. Regarding compensatory proliferation, the authors should cite the recent defining work by Bergmann and Fan (PMID: 40640821). Additionally, I recommend discussing Waghmire et al. (2024) (PMID: 38730720), who reaches similar conclusions using a different model system.

Referee #2:

While the Hippo signaling pathway is generally recognized as a tumor suppressor, emerging evidence suggests that its activation can, in certain contexts, contribute to tumorigenesis for example by modulating tumor immunogenicity. The study by Honda et al. investigates the effects of Hippo pathway activation using the developing Drosophila wing imaginal disc as a model. The authors demonstrate that spatially restricted activation of Hippo signaling, achieved by downregulating strip/yki or overexpressing wts using primarily the engrailed (en-Gal4) or patched (ptc-Gal4) drivers, induces localized patches of phosphorylated ribosomal protein S6-positive epithelial cells, indicative of mTOR activation. Through genetic epistasis experiments, the authors show that this non-autonomous mTOR activation requires the Hippo target genes Atg8a, Cyclin E, and bantam, as well as activation of Dronc and the production of the growth factors Spitz and Wg. Furthemore, they implicate the amino acid transporters Sat1 and Sat2, which were identified as suppressors of pupal lethality caused by neuron-specific Hippo activation. Based on these findings, the authors propose that Hippo-activated cells can act as oncogenic niches, promoting non-cell-autonomous tumorigenesis via Dronc-mediated growth factor secretion and Sat1/2-dependent modulation of amino acid transport. While the study presents potentially impactful findings, significant concerns regarding data presentation, experimental design, and interpretation must be addressed to support the authors' conclusions.

Major concerns:

Definition and characterization of pS6-positive cells:
The authors define pS6-positive cells as tumors, but this conclusion is not supported by the provided data. Markers such as pS6, MMP1, and EdU incorporation does not convincingly demonstrate tumorigenesis, as they can also be activated during regenerative or stress responses, including senescence. Imporatnly, EdU labeling (shown only ones with en-Gal4) marks replicating, not necessarily dividing, cells. This weakens their claim of hyperproliferation. Additional markers such as phospho-Histone H3 (pH3) for mitosis and polarity markers would provide a more accurate characterization of these cells.

MMP1 is a secreted protease, and antibody staining alone does not clarify which cells are producing it. The use of established transgenic MMP1 transcriptional reporters would help determine the cellular source of MMP1 expression.

Non-cell-autonomous activation of mTOR signaling:
The central claim that Hippo pathway activation induces non-cell-autonomous mTOR signaling (based of pS6 staining) is not convincingly demonstrated. The stereotypical emergence of pS6-positive cells in distinct positions for ptc-Gal4 and en-Gal4 could indicate "weak points" sussceptible to transformation. Lineage tracing using G-TRACE with en-Gal4, ptc-Gal4, and a

control driver such as nub-Gal4 is needed to definitively show that pS6-positive cells do not originate from Hippo-activated lineages.

The observed morphological alterations such as the reduced or absent hinge region (e.g. Figure 4A, E, H, Figure 7I-N, Figure 1C-E, J,K) and duplications of the wing disc domains (e.g. pouch/notum domain in Figure 1O and Figure 4H) are more consistent with regenerative responses than transformation. This interpretation should be considered, particularly in the context of compartment-specific tissue loss and regrowth.

The authors should provide info on developmental staging of larvae and examine whether Hippo pathway activation leads to developmental delays correlated with the presence of pS6-positive cells.

Role of Sat1/Sat2:
The identification of Sat1/Sat2 as modulators of the Hippo-mediated phenotype is compeling. However, their cell autonomous roles remain unclear. The data suggest that inhibition of Sat1/2 affects cell survival. What is the phenotype of sat1/sat2 RNAi alone with ptc-Gal4 or en-Gal4? Is Sat1/2 required for the survival of Hippo-activated cells?

The model in Figure 8 suggests that Sat1/2 located on PM mediate amino acid efflux, but this lacks any direct evidence.

The observation that increased dietary yeast/protein partially rescues the OK6-Gal4, UAS-strip-RNAi phenotype is interesting considering that no rescue is observed without extra yeast supplementation. However, its relationship to autophagy and Sat1/2-mediated amino acid transport (efflux/import dynamics) remains unclear. Does dietary protein affect both the cell-autonomous and non-autonomous consequences of Hippo activation? Unfortunately, neither composition of the standard fly food nor the food used for the genetic screen is availbale in the manuscript.

Role of Spitz:
Spitz-RNAi suppresses pS6-positive cell formation in ptc>strip-RNAi wing discs (Figure 4J, K), but appears ineffective in en>strip-RNAi backgrounds (Figure 7J). The authors should comment on this discrepancy and the dramatic rescue of wing disc morphology seen with ptc>strip-RNAi, spitz-RNAi, which is not observed using the en-Gal4 driver.

Data presentation and Figures:
Several datasets, such as in Figure 4K, 4F, display a bimodal distribution, with distinct low and high-value groups, suggesting potential biological heterogeneity or experimental variability. This should be addressed in the M&M or Figure legends. The authors state that each experiment includes at least 10 wing discs. It would be helpful to indicate how many times they repeated the experiments.

In Figure 1F, L, overlays with four channels (E-cad, MMP1, tubulin, pS6) obscure important spatial details. Single-channel images of control and experimental wing discs are needed to assess cell morphology and polarity.

The designation of micrographs in Figure EV1 as "3D" is misleading. These are 2D projections of confocal z-stacks and should be labeled as such.

Figure 1 legend: Panel 1F is not an XZ section, as stated in the figure legend.

I would like to suggest to use "incorporation" instead of "uptake" when referring to EdU.

Figure S2 "daip1" should be "diap1".

Other comments:
The attempt to link Drosophila findings to the human cancer context appears underdeveloped. The CRC tumors are highly heterogeneous and differ significantly from the wing disc model. If the authors wish to retain this comparison, more robust rationale, and explanation of their analysis would be needed and included in the result section rather than in the discussion.

Referee #3:

In this article, submitted to EMBO Reports, Honda and collaborators analyses the oncogenic potential of Hippo signalling in the Drosophila wing disc. They show that enhancing Hippo signalling by knocking down Strip, a negative Hippo regulator, or yki or overexpressing warts, induces non-cell autonomous activation of the mTOR pathway revealed by pS6 specifically in the dorsal hinge and ventral notum region. They identified the downstream Yki target genes bantam, CyclinE and atg8A, as key mediators of this effect. They show that on one hand, Dark, downstream of DRONC induces the Rho1/Wg/Spitz pathway that triggers pS6 non-cell autonomously. On the other hand, they identified the amino acid transporters Sat1/2, as critical mediators of the non-autonomous pS6 induction downstream of Hippo activation. The observations are interesting and provide key new knowledge on the non-autonomous effect of hippo signalling. Below are some general and specific comments on the data and interpretation of

result for consideration before publication.

General comments:
• The authors refer all along the text to the formation of non-autonomous tumours by Hippo activation. I see clearly induction of pS6 and MMPI but I wonder why the authors think that these cells are tumour cells?
• Re-building the Figures would help follow the data by keeping white spaces at a minimum and having panels lettering in alphabetical order.
• In some panels, confocal images seem to show extreme examples (e.g. Figure 4A-C). It would be more appropriate to pick up examples based on quantifications with values close to the mean.
• A table in Materials and Methods indicating the genotype for every panel would be required.
Specific comments:
• The authors use the UAS-Gal4 system to induce Hippo signalling in distinct domains. UAS-RFP or UAS-GFP allows to mark targeted cells. Hippo activation could affect the expression levels of RFP in some regions (e.g. Fig. 1J weaker RFP outside the pouch). One possibility is that patch or engrailed expression goes down in cells with higher Hpo activity (" loss differentiated characteristics" ????) and consequently RFP goes down. If so, how the authors can be sure that the pS6-expressing cells where not Patch- or Engrailed-expressing cells that had turned off RFP? This could be important to demonstrate the non-autonomous effect.
• nubbin-Gal4 also drives expression in the first ring of the hinge domain (see Figure 2B) No pS6 is observed in wing discs with higher Hippo activity. However, in Figure 2N. Ay-Gal4 targets the first ring of the hinge domain and induces non-autonomous pS6. How the author explains this?
• In Figure 1I the scale bars are different in panel J and K. Would be better to use the same.
• In discs with higher Hippo, induced with ptc-Gal4 or en-Gal4, the notum is smaller. How this can be explained if Hippo induced non-autonomous "tumours" in the ventral notum?
• In Figure 1Q, the author should explain what was quantified. Why the authors did not quantify the intensity of the EdU staining?
• For all panels showing the suppression of the pS6 induction, how many UAS were used for each condition. It could be important to make sure that the effect seen is not due to titration of UAS transgenes.
• In Figure 4N-Q, the authors quantify the Wg area over the RFP area. But since the RFP area is expected to decrease in cells with higher Hippo activity, the increase observed due to the cell-autonomous effect of Hippo in reducing tissue growth. Could the author justify the rational they used to make these quantifications.
• In Figure 5, I guess the en-Gal4 was used. At least this info should be in the Figure legend.
• Figure 7F, G and O, the p-value should be shown to prove synergistical effects between sat1 and sat2 and spitz.
• The discussion is mainly related to presenting other results and summarising the data presented in the result part. To my point of view, data from tumour samples should be moved to the result section. Many interesting points could be discussed. For instance, many pS6-possitive cells are found at very long distance from RFP+ cells. How this can be? What could explain the tissue-specific behaviour? The authors propose that bantam and Cyclin E are upstream of Sat1/2. What is the reasoning behind this model and possible mechanism?

We express our gratitude to the reviewers for their insightful comments. We have addressed each of the referees' comments individually below and have revised the manuscript accordingly. The revised sections are highlighted in red in the manuscript.

Referee #1:

The Hippo signaling pathway is widely known for its role in restricting tissue growth and acting as a tumor suppressor. In canonical models, loss of Hippo activity leads to autonomous overgrowth via nuclear translocation of Yorkie/YAP and activation of growth-promoting genes. However, recent studies suggest a paradoxical role where Hippo activation itself may promote tumorigenesis under certain conditions. The underlying mechanisms of such non-canonical, pro-tumorigenic functions of Hippo signaling are poorly understood. The authors aim to investigate this possibility using Drosophila wing discs as a model system.

Specifically, they authors show that activation of Hippo signaling (via strip RNAi, yki RNAi, or wts overexpression) induces mTOR activation, MMP1 expression, and EdU incorporation in adjacent cells, consistent with non-cell-autonomous tumorigenesis. These effects occur outside the Hippo-activated zone, in a signaling niche. Using multiple Gal4 drivers and clonal analysis, the authors report that the tumor-inducing capacity of Hippo-activated cells is spatially restricted to the hinge and ventral notum regions of the wing disc. Furthermore, targeted knockdown or overexpression of rpr/hid/grim, bantam, atg8a, and cyclin E are necessary for inducing mTOR activation in neighboring cells. These genes likely act downstream or independently of Yki suppression to mediate apoptotic and stress signals that promote these non-cell-autonomous effects. Interestingly, the authors demonstrate that tumorigenesis is dependent on the initiator caspase Dronc (which itself is considered a tumor suppressor) and its adaptor Dark, but not on effector caspases. This observations reveals another non-apoptotic function of Dronc for compensatory proliferation. Wg and Spitz are required for tumor formation and are upregulated in the hinge/notum region in response to Hippo activation which suggests that they are key signaling ligands in this process. A very nice temporal analysis by the authors shows that Wg upregulation and Dronc activation precede mTOR activation in neighboring cells. This temporal order supports a causal relationship where Hippo signaling initiates Dronc/Wg activation, which then promotes non-cell autonomous overgrowth. However, ectopic activation of Dronc and Wg (via hid/rpr + p35 or Wg overexpression) is not sufficient to induce non-cell-autonomous mTOR activation, suggesting that additional signals from Hippo activated cells are required. Finally, the authors perform a genetic screen and identify Sat1 and Sat2 as novel, redundant amino acid transporters involved in tumorigenesis. Knockdown of sat1/2 reduces mTOR activity and cell growth both autonomously and non-autonomously. Also, the combined sat1/2 and spitz suppression has a synergistic effect.

Altoghter, this study reveals an unexpected tumor-promoting role for the Hippo pathway via a

non-cell-autonomous mechanism mediated by Dronc, Wg/Spitz, and nutrient transporters and thereby challenges the dogma that Hippo activation acts purely tumor-suppressive. However, while Hippo signaling acts as a tumor suppressor cell-autonomously, in the context described here it acts as a tumor promoter in a non-cell autonomous manner. In the latter case, Hippo-activated cells act as "oncogenic niches", and shape the local tumor microenvironment through secretion of signaling factors and nutrient exchange. These findings expand the functional scope of Hippo signaling.
Overall, this work is very well worked out, the conclusions are supported by the data and the manuscript is usually well written. I only have a few comments to futher improve it.

1. While overexpression of hid/rpr + p35 (or Wg expression) is not sufficient to mimic Hippo induced tumorigenesis, can combined overexpression of hid/rpr + p35 (or Wg) with bantam and/or atg8a in hinge/notum region induce non-autonomous tumors?

We thank Referee #1 for this valuable suggestion. To validate our hypothesis presented in Fig. 6B, we simultaneously overexpressed *hid*, *p35* and *bantam sponge* using *en-Gal4*. Our findings indicate a significant increase in the frequency of pS6+ clusters (mTOR+ cell clusters), which is consistent with our hypothesis. This is detailed in the Results section, and the corresponding data are presented in Fig. 6H–L.

2. The authors talk about tumorigenesis in the context of these hippo-induced overgrowths. However, can they clarify more specifically the cellular outcomes in recipient mTOR+ cells e.g., tumorigenic (neoplastic) vs hyperplastic?

We thank Referee #1 for this valuable suggestion. To elucidate the characteristics of mTOR+ cells, we investigated the expression of cell adhesion and polarity markers, specifically E-cadherin (E-Cad; adherence junction) and Discs large (Dlg; tight junction). Our findings indicate a reduction in the expression of both E-Cad and Dlg in mTOR+ cell clusters. Based on the reference (Dev Cell. 60:1036-1052, 2025), we posit that mTOR+ cells are likely dysplastic tumor cells. We have incorporated these experiments into Fig. EV2G–N and detailed these results in the relevant sections (Results and Discussion).

3. The function of Sat1 and Sat2 is inferred based on homology and localization. However, functional transport assays were not provided by the authors. Can they provide more evidence that these proteins indeed act as aa transporters?

Given the notable similarity between Sat1 and Sa12 in both amino acid sequence and genomic

location, we focused on the role of Sat1 as an amino acid transporter. We carefully measured amino acid levels in the wing disc expressing Sat1 using LC-MS/MS. Our results suggest that Sat1 overexpression is associated with increased concentrations of various amino acids within wing discs. Although the exact specificity of amino acids transported by Sat1 is yet to be fully understood, our findings indicate that Sat1 may play a significant role in facilitating the incorporation of amino acids, including methionine, into cells and facilitate protein synthesis. This assertion is further supported by our observation that HPG, a methionine analog, is incorporated into Hippo-activated and mTOR+ cell clusters, and this incorporation is reduced by *Sat1/2* knockdown. The new data on Sat1 function are presented in Fig. 7A, I–P. Since biochemical characterization, including substrate specificity, of amino acid transporters is technically challenging, it is worth a complete another paper and might not be feasible to pursue this entirely new direction at this moment.

4. Overall the writing is clear and logical. However, some sections in the results (e.g., timing experiments or transporter functions) could benefit from more concise phrasing and improved figure referencing.

We thank Referee #1 for this suggestion. We have conducted a thorough review of the entire manuscript, including the arrangement and referring of Figures.

5. The reference list requires improvement, as the authors have not consistently cited the most relevant papers. For example, when discussing bantam, the authors should include Brennecke et al. (2003), Cell. Several other instances suggest that key publications have been overlooked. Regarding compensatory proliferation, the authors should cite the recent defining work by Bergmann and Fan (PMID: 40640821). Additionally, I recommend discussing Waghmire et al. (2024) (PMID: 38730720), who reaches similar conclusions using a different model system.

We extend our gratitude to Referee #1 for supplying reference information. As recommended, we have cited Brennecke et al. (PMID: 12679032), Bergmann and Fan (PMID: 40640821), and Waghmire et al. (PMID: 38730720) in the revised manuscript. Furthermore, we have meticulously reviewed the cited references to ensure there are no omissions or inaccuracies.

Referee #2:

While the Hippo signaling pathway is generally recognized as a tumor suppressor, emerging evidence suggests that its activation can, in certain contexts, contribute to tumorigenesis for example by modulating tumor immunogenicity. The study by Honda et al. investigates the effects of Hippo

pathway activation using the developing Drosophila wing imaginal disc as a model. The authors demonstrate that spatially restricted activation of Hippo signaling, achieved by downregulating strip/yki or overexpressing wts using primarily the engrailed (en-Gal4) or patched (ptc-Gal4) drivers, induces localized patches of phosphorylated ribosomal protein S6-positive epithelial cells, indicative of mTOR activation. Through genetic epistasis experiments, the authors show that this non-autonomous mTOR activation requires the Hippo target genes Atg8a, Cyclin E, and bantam, as well as activation of Dronc and the production of the growth factors Spitz and Wg. Furthemore, they implicate the amino acid transporters Sat1 and Sat2, which were identified as suppressors of pupal lethality caused by neuron-specific Hippo activation. Based on these findings, the authors propose that Hippo-activated cells can act as oncogenic niches, promoting non-cell-autonomous tumorigenesis via Dronc-mediated growth factor secretion and Sat1/2-dependent modulation of amino acid transport. While the study presents potentially impactful findings, significant concerns regarding data presentation, experimental design, and interpretation must be addressed to support the authors' conclusions.

Major concerns:

Definition and characterization of pS6-positive cells:

The authors define pS6-positive cells as tumors, but this conclusion is not supported by the provided data. Markers such as pS6, MMP1, and EdU incorporation does not convincingly demonstrate tumorigenesis, as they can also be activated during regenerative or stress responses, including senescence. Imporatnly, EdU labeling (shown only ones with en-Gal4) marks replicating, not necessarily dividing, cells. This weakens their claim of hyperproliferation. Additional markers such as phospho-Histone H3 (pH3) for mitosis and polarity markers would provide a more accurate characterization of these cells.

We thank Referee #2 for this valuable suggestion. As suggested, we examined the level of Phospho-Histon H3 (PH3) in the wing disc possessing Hippo-activated cells. We found that the number of PH3+ cells significantly increased in the pS6+ cells (mTOR+ cell cluster), indicating that pS6-positive cells are hyperproliferative state. These results are presented in Fig. EV1I–K.

In addition, we investigated the expression of cell adhesion and polarity markers, specifically E-cadherin (E-Cad; adherence junction) and Discs large (Dlg; tight junction). Our findings indicate a reduction in the expression of both E-Cad and Dlg in mTOR+ cell clusters. Based on the reference (Dev Cell. 60:1036-1052, 2025), we posit that mTOR+ cells are likely dysplastic tumor cells. We have incorporated these experiments into Fig. EV2G–N and detailed these results in the relevant sections (Results and Discussion).

MMP1 is a secreted protease, and antibody staining alone does not clarify which cells are producing it. The use of established transgenic MMP1 transcriptional reporters would help determine the cellular source of MMP1 expression.

We sincerely appreciate Reviewer #2's insightful suggestion regarding the use of the MMP1 transcriptional reporter, *MMP1-lacZ* (BL12205). Although our initial efforts with *MMP1-lacZ* did not work, we remained committed to exploring alternative approaches. Consequently, we decided to assess JNK activity, given that MMP1 is upregulated through the JNK pathway. By utilizing *puc-stringer*, a robust JNK reporter, we observed a notable increase in the *puc-stringer* signal within mTOR-activated cell clusters. This compelling evidence suggests that mTOR-activated cell clusters may enhance MMP1 expression via JNK activation. These important findings are detailed in Fig. EV2D–F.

Non-cell-autonomous activation of mTOR signaling:
The central claim that Hippo pathway activation induces non-cell-autonomous mTOR signaling (based of pS6 staining) is not convincingly demonstrated. The stereotypical emergence of pS6-positive cells in distinct positions for ptc-Gal4 and en-Gal4 could indicate "weak points" sussceptible to transformation. Lineage tracing using G-TRACE with en-Gal4, ptc-Gal4, and a control driver such as nub-Gal4 is needed to definitively show that pS6-positive cells do not originate from Hippo-activated lineages.

We thank Referee #2 for this valuable suggestion. Given that *en-Gal4+*, as opposed to *ptc-Gal4+*, cells infrequently exhibit dedifferentiation during wing disc development (Nat. Methods 6: 603-605, 2009), we performed lineage tracing of Hippo-activated cells utilizing G-TRACE with *en-Gal4*. The G-TRACE analysis indicates that Hippo-activated cells do not facilitate dedifferentiation or transformation, and that pS6-positive cells are not predominantly derived from Hippo-activated cells. The G-TRACE data are presented in Figure EV1A–H.

The observed morphological alterations such as the reduced or absent hinge region (e.g. Figure 4A, E, H, Figure 7I-N, Figure 1C-E, J,K) and duplications of the wing disc domains (e.g. pouch/notum domain in Figure 1O and Figure 4H) are more consistent with regenerative responses than transformation. This interpretation should be considered, particularly in the context of compartment-specific tissue loss and regrowth.

Thanks Referee #2 for this insightful suggestion. We agree with their observation that the morphological changes we noted are similar to those seen in regenerative processes. In our revised

manuscript, we have elaborated on the phenotypic similarities and detailed how identical signaling pathways are involved in both non-cell autonomous tumorigenesis and regeneration. Specifically, we have highlighted that Wg/WNT are upregulated in apoptotic cells during regeneration. These insights have been integrated into the discussion section of the revised manuscript.

The authors should provide info on developmental staging of larvae and examine whether Hippo pathway activation leads to developmental delays correlated with the presence of pS6-positive cells.

We thank Referee #2 for this valuable suggestion. We assessed the developmental delays in larvae possessing Hippo-activated cells. Our findings indicate that the average developmental duration until the wandering third instar larval stage is extended by approximately one day due to Hippo activation. This developmental delay was mitigated by RNA interference of *rpr*, *hid*, and *grim* (*miRHG*), which are capable of inhibiting pS6 induction resulting from Hippo activation, suggesting that pS6-positive cells or apoptosis may be responsible for the delay (Fig. A, B below). Furthermore, we investigated whether the developmentally delayed individuals exhibited the pS6-positive region in the wing disc. The one-day developmental delay does not appear to influence the pS6-positive region, indicating a limited correlation between the developmental period and the presence of pS6-positive cells. The data illustrating this correlation are presented in Fig. C below.

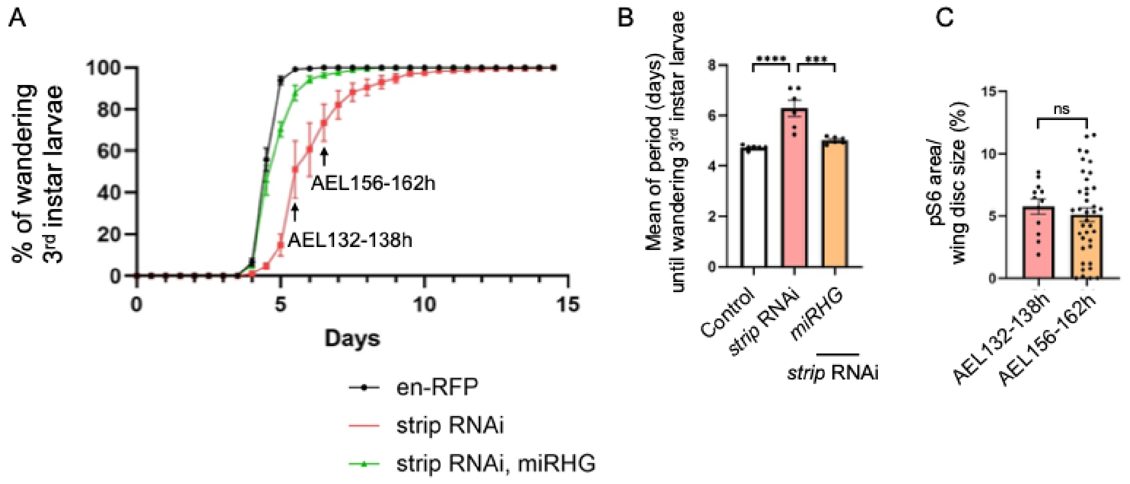

Role of Sat1/Sat2:

The identification of Sat1/Sat2 as modulators of the Hippo-mediated phenotype is competing. However, their cell autonomous roles remain unclear. The data suggest that inhibition of Sat1/2 affects cell survival. What is the phenotype of sat1/sat2 RNAi alone with ptc-Gal4 or en-Gal4? Is Sat1/2 required for the survival of Hippo-activated cells?

We thank Referee #2 for this valuable suggestion. We examined whether Sat1/2 is required for cell survival. Since apoptosis was not significantly observed in the *sat1/2* mutant, Sat1/2 would not be associated with cell survival in the usual development of the wing disc (Fig. EV5K). On the other hand, *sat1/2* mutation enhanced apoptosis in Hippo-activated cells, suggesting that Sat1/2 have the potential to promote cell survival in Hippo-activated cells. The new data on Sat1/2 function are presented in Fig. EV5J–N.

The model in Figure 8 suggests that Sat1/2 located on PM mediate amino acid efflux, but this lacks any direct evidence.

(While we have described this response to Referee #1 above, we reiterate it here for the benefit of Referee #2)

We thank Referee #2 for this valuable suggestion. Given the notable similarity between Sat1 and Sa12 in both amino acid sequence and genomic location, we focused on the role of Sat1 as an amino acid transporter. We carefully measured amino acid levels in the wing disc expressing Sat1 using LC-MS/MS. Our results suggest that *sat1* overexpression is associated with increased concentrations of various amino acids within wing discs. Although the exact specificity of amino acids transported by Sat1 is yet to be fully understood, our findings indicate that Sat1 may play a significant role in facilitating the incorporation of amino acids, including methionine, into cells. This assertion is further supported by our observation that HPG, a methionine analog, is incorporated into Hippo-activated and mTOR+ cell clusters, and this incorporation is reduced by Sat1/2 knockdown. Thus, Sat1/2 would be associated with cell-autonomous and non-cell-autonomous amino acid flux/metabolism. However, since we could not obtain any direct evidence that Sat1/2 regulate amino acid efflux, we improved the hypothesis that Sat1/2 import amino acids, promoting cell survival and subsequent growth factor secretion or affecting the amino acid level in the microenvironment. The new data on Sat1 function and hypothesis are presented in Fig. 7A, I–P and Fig. 9, respectively.

The observation that increased dietary yeast/protein partially rescues the OK6-Gal4, UAS-strip-RNAi phenotype is interesting considering that no rescue is observed without extra yeast supplementation. However, its relationship to autophagy and Sat1/2-mediated amino acid transport (efflux/import dynamics) remains unclear. Does dietary protein affect both the cell-autonomous and non-autonomous consequences of Hippo activation? Unfortunately, neither composition of the standard fly food nor the food used for the genetic screen is availbale in the manuscript.

We thank Referee #2 for this valuable suggestion. Details of the diet composition were listed in the

“method” section. We also examined whether nutritional conditions affected the emergence of pS6 cells. We used holidic medium and varied methionine levels. While the diet reducing methionine concentration had no effect, the diet increasing methionine concentration enhanced the emergence of pS6 cells. These data are presented in Fig. 7M–P.

Role of Spitz:

Spitz-RNAi suppresses pS6-positive cell formation in ptc>strip-RNAi wing discs (Figure 4J, K), but appears ineffective in en>strip-RNAi backgrounds (Figure 7J). The authors should comment on this discrepancy and the dramatic rescue of wing disc morphology seen with ptc>strip-RNAi, spitz-RNAi, which is not observed using the en-Gal4 driver.

We thank Referee #2 for this valuable suggestion. Given the differential effects of *wg* RNAi between *ptc-Gal4* and *en-Gal4*, we examined the Wg expression levels in *strip*-knocked down wing discs utilizing either *en-Gal4* or *ptc-Gal4*. The application of *strip* RNAi with *en-Gal4* effectively induced Wg expression compared to *ptc-Gal4* (Right Figure). Consequently, we hypothesize that while *wg* RNAi predominantly eliminates Wg in Hippo-activated cells using *ptc-Gal4*, it moderately reduces Wg in these cells when employing *en-Gal4*.

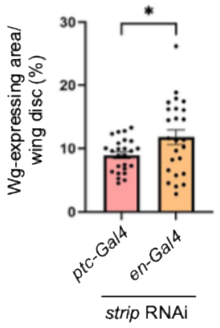

Data presentation and Figures:

Several datasets, such as in Figure 4K, 4F, display a bimodal distribution, with distinct low and high-value groups, suggesting potential biological heterogeneity or experimental variability. This should be addressed in the M&M or Figure legends. The authors state that each experiment includes at least 10 wing discs. It would be helpful to indicate how many times they repeated the experiments.

As noted by Referee #2, we also questioned whether this "bimodal distribution" is biologically reproducible. We conducted a same experiment to assess the reproducibility of data distribution. Our results confirmed that *wg* RNAi or *spitz* RNAi can suppress the non-cell autonomous induction of pS6^{+} cells, and the bimodal distribution was again observed in the different groups (Right Figure). At present, the underlying cause of this phenomenon remains unknown. We have documented the

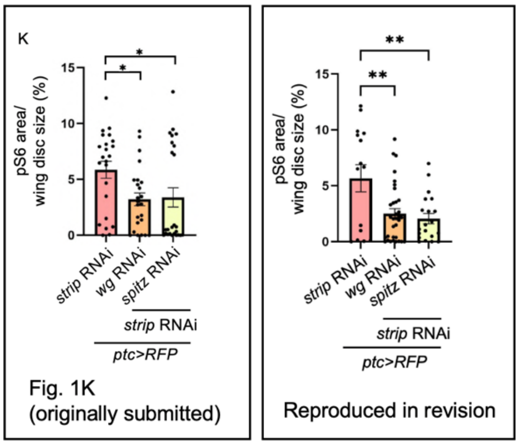

Figure
Results were reproducible with bimodal distribution

occurrence of the bimodal distribution and the number of experiments in the corresponding Figure legends or Method section.

In Figure 1F, L, overlays with four channels (E-cad, MMP1, tubulin, pS6) obscure important spatial details. Single-channel images of control and experimental wing discs are needed to assess cell morphology and polarity.

As suggested, we put single-channel images to assess the phenotype clearly in Figure 1H and Q (formerly Fig. 1F and L) to make the image clear.

The designation of micrographs in Figure EV1 as "3D" is misleading. These are 2D projections of confocal z-stacks and should be labeled as such.

The term “3D” has been removed.

Figure 1 legend: Panel 1F is not an XZ section, as stated in the figure legend.

We have replaced the “XZ section” to “vertical section”.

I would like to suggest to use "incorporation" instead of "uptake" when referring to EdU.

The term “uptake” has been changed to "incorporation".

Figure S2 "daip1" should be "diap1".

The term " daip1" has been changed to "diap1".

Other comments:
The attempt to link Drosophila findings to the human cancer context appears underdeveloped. The CRC tumors are highly heterogeneous and differ significantly from the wing disc model. If the authors wish to retain this comparison, more robust rationale, and explanation of their analysis would be needed and included in the result section rather than in the discussion.

We thank Referee #2 for this important suggestion. After reconsidering the inclusion of the original Fig. EV5, we concluded that the data analysis would be more meaningful when supported by experimental results using human cultured cells. In fact, we have already begun generating knockout

human cell lines for this purpose as part of our future research plans. As these experiments are still ongoing, we have decided to remove the original Fig EV5 from the current version of the manuscript.

Referee #3:

In this article, submitted to EMBO Reports, Honda and collaborators analyses the oncogenic potential of Hippo signalling in the Drosophila wing disc. They show that enhancing Hippo signalling by knocking down Strip, a negative Hippo regulator, or yki or overexpressing warts, induces non-cell autonomous activation of the mTOR pathway revealed by pS6 specifically in the dorsal hinge and ventral notum region. They identified the downstream Yki target genes bantam, CyclinE and atg8A, as key mediators of this effect. They show that on one hand, Dark, downstream of DRONC induces the Rho1/Wg/Spitz pathway that triggers pS6 non-cell autonomously. On the other hand, they identified the amino acid transporters Sat1/2, as critical mediators of the non-autonomous pS6 induction downstream of Hippo activation. The observations are interesting and provide key new knowledge on the non-autonomous effect of hippo signalling. Below are some general and specific comments on the data and interpretation of result for consideration before publication.

General comments:

• The authors refer all along the text to the formation of non-autonomous tumours by Hippo activation. I see clearly induction of pS6 and MMPI but I wonder why the authors think that these cells are tumour cells?

(While we have described this response to Referee #1 and #2 above, we reiterate it here for the benefit of Referee #3)

We thank Referee #3 for this valuable suggestion. To elucidate the characteristics of mTOR+ cells, we investigated proliferation marker, PH3, and the expression of cell adhesion and polarity markers, specifically E-cadherin (E-Cad; adherence junction) and Discs large (Dlg; tight junction). Our findings indicate an increase in PH3 number and a reduction in the expression of both E-Cad and Dlg in mTOR+ cell clusters. Based on the reference (Dev Cell. 60:1036-1052, 2025), we posit that mTOR+ cells are likely dysplastic tumor cells. We have incorporated these experiments into Fig. EV1I–K, Fig. EV2G–N, and detailed these results in the relevant sections (Results and Discussion).

• Re-building the Figures would help follow the data by keeping white spaces at a minimum and having panels lettering in alphabetical order.

We thank Referee #3 for this suggestion. We have checked and organized the arrangements for Figure panels as suggested.

• In some panels, confocal images seem to show extreme examples (e.g. Figure 4A-C). It would be more appropriate to pick up examples based on quantifications with values close to the mean.

In response to the reviewers' comments, we sincerely apologize for the inclusion of certain panels that featured extreme examples. We have meticulously reviewed all the panels and have ensured the inclusion of average examples in the panel displays.

• A table in Materials and Methods indicating the genotype for every panel would be required.

We have submitted the genotype list for every panels.

Specific comments:

• The authors use the UAS-Gal4 system to induce Hippo signalling in distinct domains. UAS-RFP or UAS-GFP allows to mark targeted cells. Hippo activation could affect the expression levels of RFP in some regions (e.g. Fig. 1J weaker RFP outside the pouch). One possibility is that patch or engrailed expression goes down in cells with higher Hpo activity (" loss differentiated characteristics" ????) and consequently RFP goes down. If so, how the authors can be sure that the pS6-expressing cells where not Patch- or Engrailed-expressing cells that had turned off RFP? This could be important to demonstrate the non-autonomous effect.

(While we have described this response to Referee #2 above, we reiterate it here for the benefit of Referee #3)

We thank Referee #3 for this valuable suggestion. Given that *en-Gal4*, as opposed to *ptc-Gal4*, positive cells infrequently exhibit dedifferentiation during wing disc development (Nat. Methods 6: 603-605, 2009), we performed lineage tracing of Hippo-activated cells utilizing G-TRACE with *en-Gal4*. The G-TRACE analysis indicates that Hippo-activated cells do not facilitate dedifferentiation or transformation, and that pS6-positive cells are not predominantly derived from Hippo-activated cells. The data pertaining to G-TRACE are illustrated in Fig. EV1A–H.

• nubbin-Gal4 also drives expression in the first ring of the hinge domain (see Figure 2B) No pS6 is observed in wing discs with higher Hippo activity. However, in Figure 2N. Ay-Gal4 targets the first ring of the hinge domain and induces non-autonomous pS6. How the author explains this?

nubbin-Gal4 driver exhibits activity not only in the pouch but also in the hinge-ventral region on the medial fold. Apoptosis signaling, which is associated with the induction of mTOR tumors, was significantly activated in the pouch but not in the hinge-ventral region on the medial fold when *strip RNAi* was applied. Conversely, *Ay-Gal4* robustly induced apoptosis in both the pouch and the hinge-ventral region on the medial fold through *strip RNAi*. These findings suggest that the efficiency of apoptosis induction by *nubbin-Gal4* is lower than that of *Ay-Gal4* in the hinge-ventral region on the medial fold, resulting in differential incidence of mTOR tumors. The results of these experiments are presented in Fig. EV3G–L.

• In Figure 1I the scale bars are different in panel J and K. Would be better to use the same.

We have changed the size of scale bars as suggested.

• In discs with higher Hippo, induced with ptc-Gal4 or en-Gal4, the notum is smaller. How this can be explained if Hippo induced non-autonomous "tumours" in the ventral notum?

We thank Referee #3 for this valuable suggestion. We calculated the number of PH3 in the notum region (RFP- and pS6-negative notum region, as shown by the blue crosshatching in the schematic diagram). Since the PH3 number decreased in the notum region, where pS6 is not elevated, Hippo activation would make two populations in the notum region: high PH3 (pS6-positive) and low PH3 (pS6-negative) population. Thus, Hippo activation may decrease notum size, although the pS6-positive region in the notum enlarged.

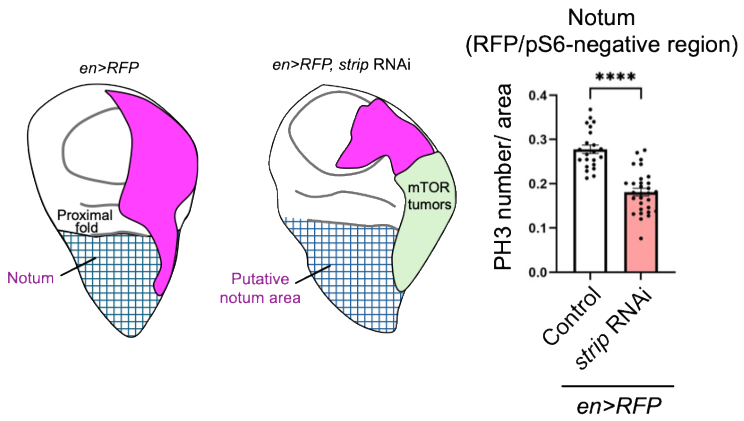

• In Figure 1Q, the author should explain what was quantified. Why the authors did not quantify the intensity of the EdU staining?

As Referee #3 suggested, we assessed the intensity of EdU staining, and our findings were consistent with the initial observations regarding the fraction of EdU staining. In the updated manuscript, we have adopted the term "the intensity of EdU staining" in Fig. 1L (formerly Fig. 1Q).

• For all panels showing the suppression of the pS6 induction, how many UAS were used for each condition. It could be important to make sure that the effect seen is not due to titration of UAS transgenes.

We thank Referee #3 for this criticism. We acknowledge that addressing the concern regarding the "titration effect of UAS transgene" presents technical challenges across all experiments. In an effort to address this as thoroughly as possible, we have investigated the induction effect of non-cell autonomous pS6 with and without *UAS-GFP*. As illustrated in the Figure on the right, no differences were observed between the two conditions. While we cannot entirely rule out the possibility of a titration effect in other experiments, providing a comprehensive genotype list for all experiments may be beneficial for readers in evaluating the results.

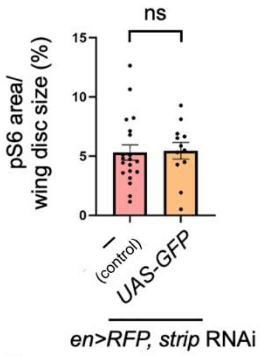

Figure
Titration effect of UAS transgene was not observed

• In Figure 4N-Q, the authors quantify the Wg area over the RFP area. But since the RFP area is expected to decrease in cells with higher Hippo activity, the increase observed due to the cell-autonomous effect of Hippo in reducing tissue growth. Could the author justify the rational they used to make these quantifications.

We thank Referee #3 for this criticism. We propose that the observed relative increase in the Wg expression area, compared to the RFP area, might be due to the induction of Wg expression by Hippo-activated cells. Nevertheless, considering Referee #3's suggestion that this increase could be a result of a reduction in the RFP area, we conducted a thorough investigation to verify whether Hippo-activated cells indeed promote Wg expression by confirming the presence of ectopic Wg expression. Our results revealed that ectopic Wg expression was exclusively present in wing discs with Hippo-activated cells, indicating that these cells may enhance Wg expression in the wing disc. The outcomes of these experiments are shown in Fig. 4N.

• In Figure 5, I guess the en-Gal4 was used. At least this info should be in the Figure legend.

As Referee #3 suggested, the information was described in Fig. 5A and the corresponding figure legend.

• Figure 7F, G and O, the p-value should be shown to prove synergistical effects between sat1 and

sat2 and spitz.

We thank Referee #3 for this constructive suggestion. In the revised manuscript, we have included the individual p-values in the figure legends of the corresponding graphs.

• The discussion is mainly related to presenting other results and summarising the data presented in the result part. To my point of view, data from tumour samples should be moved to the result section. Many interesting points could be discussed. For instance, many pS6-possitive cells are found at very long distance from RFP+ cells. How this can be? What could explain the tissue-specific behaviour? The authors propose that bantam and Cyclin E are upstream of Sat1/2. What is the reasoning behind this model and possible mechanism?

We thank Referee #3 for their insightful comments. Upon receiving feedback regarding the "data analysis of human cancer", we meticulously reevaluated the inclusion of the original Fig. EV5. We determined that the data analysis would be more robust if supported by experimental results utilizing human cultured cells. Consequently, we have initiated the generation of knockout human cell lines for this purpose as part of our future research endeavors. As these experiments are currently in progress, we have opted to exclude the original Fig. EV5 from the present version of the manuscript.

The query raised by Referee #3 concerning the emergence of numerous pS6-positive cells at a considerable distance from RFP+ cells is indeed intriguing; however, we currently lack a rational explanation. It is conceivable that the initial pS6-positive cells, induced in proximity to RFP+ cells, may effectively trigger subsequent pS6-positive cells on the opposite side of RFP+ cells. Although several possibilities exist, we do not yet have evidence to substantiate them. We intend to address this question in future studies.

Regarding the hierarchy of bantam, Cyclin E, and Sat1/2, we have revised our model to propose that bantam and Cyclin E function in coordination with Sat1/2, as reflected in the revised manuscript.

Dear Prof. Chihara,

I look forward to seeing a new revised version of your manuscript as soon as possible.

Thank you for the submission of your revised manuscript to our editorial offices. I have now received the reports from the three referees that I asked to re-evaluate the study, you will find below.

As you will see, the referees now support publication of your study in EMBO reports. Nevertheless, all three have remaining concerns, questions and suggestions to improve the manuscript, I ask you to address in a final revised manuscript. Please perform and include the requested experiment (referee #2) and address all the further minor comments and do the requested text changes. Please also provide a detailed final p-b-p-response regarding the remaining referee points and the editorial requests below.

Editorial requests:

- Please have your final manuscript carefully proofread by a native speaker. There are still typos, grammatical error or unusual phrases in the present manuscript text.

- Please provide the abstract written in present tense throughout.

- Please order the manuscript sections like this, using only these names:
Title page - Abstract - Keywords - Introduction - Results - Discussion - Methods - Data availability section - Acknowledgements - Disclosure and Competing Interests Statement - References - Figure legends - Expanded View Figure legends

- Please check again that the number "n" for how many independent experiments were performed, their nature (biological versus technical replicates), the bars and error bars (e.g. SEM, SD) and the test used to calculate p-values is indicated in the respective figure legends (main and EV figures). Please also check that all the p-values are explained in the legend, and that these fit to those shown in the figure. Please provide statistical testing where applicable. Please avoid the phrase 'independent experiment' but clearly state if these were biological or technical replicates. Please also indicate (e.g. with n.s.) if testing was performed, but the differences are not significant. In case n=2, please show the data as separate datapoints without error bars and statistics. See also:

https://link.springer.com/journal/44319/submission-guidelines#cms-Figure-and-data-presentation

If n<5, please show single datapoints for diagrams. Moreover:
Please note that the legends for figures 2, EV2, S1 is not provided in the sequential manner. This needs to be rectified.
- Please note that the exact p values are not provided in the legends of figures 1F, K, L, P; 2L, 3J, K; 4F, G, K, O, P; 5B, C, D, E; 6G, 7A, G, H, L, P; 8F, G, N, O; EV1 E, K; EV2 F, I, L; EV3 F, L; EV4 D, J; EV5 B, N; S1 C, S2 H, S3 F
- Please note that information related to n is missing in the legends of figures S1 C, F, I; S2 H, S3F

- Please name the table in the Appendix file "Appendix Table S1" and adjust the callout for the table.

- Some scale bars in the microscopic images are presently rather faint and hard to see. Please provide thicker or brighter scale bars.

- Please use our reference format (et al needs to be used after 10 author names; DOIs should only be used for preprints and datasets that have not been published yet):
https://link.springer.com/journal/44319/submission-guidelines#cms-Reference-guidelines

- Please move all the primer information presently found at several places in the Methods part to the Reagents & Tools table and add callouts to the table. Moreover, please remove the instruction text form the Reagents & Tools Table.

- Thanks for providing the source data. Please upload this as one folder per main figure, grouping together separate files for each panel of this figure (numerical or image data separated) and ZIPed together.

In addition, I would need from you uploaded separately:
- a short, two-sentence summary of the manuscript (not more than 35 words).
- two to four short (!) bullet points highlighting the key findings of your study (two lines each).
- a schematic summary figure as separate file that provides a sketch of the major findings (not a data image) in jpeg or tiff format (with the exact width of 550 pixels and a height of not more than 400 pixels) that can be used as a visual synopsis on our website.

I look forward to seeing the further revised version of your manuscript when it is ready. Please let me know if you have questions regarding the revision.

Best,

Achim Breiling
Senior Editor
EMBO Reports

Referee #1:

The authors added substantial new experimental data, including lineage-tracing controls, metabolic and dietary data, functional validation of Sat1/2, expanded tumor phenotyping, and human cancer relevance. These additions transform the manuscript from a strong genetic observation into a mechanistically and conceptually mature study, with clear implications for how Hippo-activated cells can function as metabolic and signaling hubs that drive non-cell-autonomous tumorigenesis.

The only question I still have is regarding the summary model in Figure 9. How are the amino acids transported from hippo-activated cells into the mTOR tumors? Does that also involve the Sat1/2 transporters? Or is this a different mechanism.

Other than this question I have no further suggestions for improvement.

Referee #2:

The authors have invested admirable effort into revising their manuscript, substantially strengthening both the experimental evidence and the clarity of presentation in the text and figures. The newly added data provide additional support for the role of Sat1 and Sat2 as amino acid transporters, and overall the revision reflects a significant amount of work.

That said, I remain concerned that an important conceptual issue raised during the first round of review by two reviewers has not been satisfactorily addressed. Given the importance of the central message that Hippo-activated cells function as a niche inducing non-autonomous dysplastic overgrowth, the experimental evidence on which this conclusion is based should be resolved more rigorously to ensure that the proposed model is well supported.

Two reviewers previously raised questions regarding the origin of the pS6-positive cells observed following activation of Hippo signaling using ptc-Gal4 and en-Gal4 drivers. These cells appear in stereotypical but distinct locations for each driver sometimes even distant from the "niche", while no comparable non-autonomous pS6-positive cells are observed when authors used nubbin-Gal4 or pnr-Gal4.
While the authors performed lineage tracing for en-Gal4, they did not do so for ptc-Gal4, arguing that en-Gal4-positive cells, unlike ptc-Gal4-positive cells, infrequently undergo dedifferentiation during wing disc development refering to Nat. Methods 6:603-605, 2009. However, lineage tracing serves not only to assess changes in cell fate but also to document the developmental activity pattern of the Gal4 driver itself. As demonstrated for several drivers, including ELAV-Gal4, which is generally considered pan-neuronal, driver activity can be considerably broader than expected due to transient or early expression (Berger et al., 2007, Dev Dyn., https://doi.org/10.1002/dvdy.21372, Winant et al., 2024. Genesis, https://doi.org/10.1002/dvg.23600)

For this reason, I believe it is essential that the authors perform lineage tracing experiments for ptc-Gal4 as well. Importantly, strong thresholding should be avoided, as driver activity and signal of tracer may be weak (ubi-promoter driving tracer eGFP upon FLPout) yet biologically relevant rather than entirely absent.

Minor points
The authors report that Sat1,Sat2 mutant wing discs do not exhibit increased apoptosis and conclude that these genes are not required for cell survival. However, such requirement may only become apparent in a competitive setting, in which Sat1,Sat2 mutant/deficient cells are surrounded by phenotypically wild-type neighbors, as could be assessed by inducing mutant clones or RNAi-mediated deficient compartments.

Line 126: The authors should cite the original studies that first demonstrated regulation of MMP1 by JNK, rather than only recent publication.

Figure 3A: "Cycline E" should be corrected to Cyclin E.

Referee #3:

The author of the manuscript submitted to EMBO report, untitled "The Hippo tumor suppressor pathway triggers non-cell autonomous tumorigenesis in Drosophila" significantly revised their manuscript and answered most of my queries by providing additional data.

I still have one point to highlight and suggestions regarding the text.

• The authors included new data showing that wg knockdown alone does not significantly suppress non-cell-autonomous mTOR activation (Fig. 8F). However, in Figure 4K they show that the non-cell-autonomous mTOR activation is significantly suppressed by wg knocked down. In Figure 8, en-Gal4 has been used and this driver is capable of inducing ectopic Wg (EV4). Therefore, I do not see any explanation for the discrepancy between Fig. 4K and 8F.

• Globally, the manuscript reading is a bit painful with very long sentences and sometime grammatical issues (Below are a few examples)-
Line 263: " To investigate the association with Sat1/2....." The association of what?
Line 329: "Given that Rho1, a regulator of the JNK pathway, was required in the Hippo-activated cells for non-cell-autonomous tumorigenesis (Fig. 4E, quantified in F), suggesting that.........."
Line 336: "However, unlike Hippo-activated cells, Src activation has shown to inhibit.....".
I would suggest the authors to revise the text.

• Most genetic interactions look at pS6 levels, as read out. I therefore do not think that it is correct to talk about suppression or induction of non-cell-autonomous tumorigenesis only based on this read out. Non-cell-autonomous tumorigenesis is an interpretation, but not a conclusion.

For instance, the 3 sub-titles:
rpr, hid, grim, bantam, cyclin E, and atg8a are involved in non-cell-autonomous tumorigenesis.
Hippo activation induces non-cell-autonomous tumorigenesis through Dronc-Wg/Spitz signaling.
The activation of Dronc-Wg/Spitz signaling alone is insufficient to induce non-cell-autonomous
Tumorigenesis.

Should be something like:
rpr, hid, grim, bantam, cyclin E, and atg8a are involved in non-cell-autonomous pS6 induction.
Hippo activation induces non-cell-autonomous pS6 induction through Dronc-Wg/Spitz signaling.
The activation of Dronc-Wg/Spitz signaling alone is insufficient to induce non-cell-autonomous pS6 induction.

Also, in the rest of the text, non-cell-autonomous tumorigenesis when refering to pS6 only, should be changed. The data would remain as interesting while making these changes. It is a matter of writing a scientifically correct conclusion.

Point-by-point responses for editorial requests:

We thank the Editor and the Editorial Office for handling our manuscript and for the detailed editorial guidance. We have addressed each of the requests as outlined below.

Editorial requests:

Please have your final manuscript carefully proofread by a native speaker. There are still typos, grammatical error or unusual phrases in the present manuscript text.

As requested, the entire manuscript has been professionally proofread by a native English editor (Editage).

- Please provide the abstract written in present tense throughout.

The abstract has been revised accordingly and is now written consistently in the present tense.

- Please order the manuscript sections like this, using only these names: - Title page - Abstract - Keywords - Introduction - Results - Discussion - Methods - Data availability section - Acknowledgements - Disclosure and Competing Interests Statement - References - Figure legends - Expanded View Figure legends-

The manuscript structure has been reorganized according to the journal guidelines.

- Please check again that the number "n" for how many independent experiments were performed, their nature (biological versus technical replicates), the bars and error bars (e.g. SEM, SD) and the test used to calculate p-values is indicated in the respective figure legends (main and EV figures). Please also check that all the p-values are explained in the legend, and that these fit to those shown in the figure. Please provide statistical testing where applicable. Please avoid the phrase 'independent experiment' but clearly state if these were biological or technical replicates. Please also indicate (e.g. with n.s.) if testing was performed, but the differences are not significant. In case n=2, please show the data as separate datapoints without error bars and statistics. See also:https://link.springer.com/journal/44319/submission-guidelines#cms-Figure-and-data-presentation-

All figure legends (main and EV figures) have been revised to clearly state:

- the number of biological replicates,

- the statistical tests used,
- the meaning of error bars,
- exact p-values where applicable,
- and “n.s.” where differences were not significant.

- If n<5, please show single datapoints for diagrams.

For experiments with n < 5, individual data points are now shown.

Moreover: - Please note that the legends for figures 2, EV2, S1 is not provided in the sequential manner. This needs to be rectified.

All figure legends have been corrected and reordered sequentially.

- Please note that the exact p values are not provided in the legends of figures 1F, K, L, P; 2L, 3J, K; 4F, G, K, O, P; 5B, C, D, E; 6G, 7A, G, H, L, P; 8F, G, N, O; EV1 E, K; EV2 F, I, L; EV3 F, L; EV4 D, J; EV5 B, N; S1 C, S2 H, S3 F

Exact p-values have now been included in all indicated figure legends.

- Please note that information related to n is missing in the legends of figures S1 C, F, I; S2 H, S3F

The number of replicates has now been provided for all figures.

- Please name the table in the Appendix file "Appendix Table S1" and adjust the callout for the table.

The table and its callouts have been corrected.

- Some scale bars in the microscopic images are presently rather faint and hard to see. Please provide thicker or brighter scale bars.

Scale bars have been increased in thickness and contrast for visibility.

- Please use our reference format (et al needs to be used after 10 author names; DOIs should only be used for preprints and datasets that have not been published yet):
- https://link.springer.com/journal/44319/submission-guidelines#cms-Reference-guidelines

References have been reformatted according to EMBO Reports guidelines.

- Please move all the primer information presently found at several places in the Methods part to the

Reagents & Tools table and add callouts to the table. Moreover, please remove the instruction text form the Reagents & Tools Table.

Primer information has been consolidated into the Reagents & Tools table and callouts have been added. Instruction text has been removed.

- Thanks for providing the source data. Please upload this as one folder per main figure, grouping together separate files for each panel of this figure (numerical or image data separated) and ZIPed together.

Source data have been reorganized and uploaded as one ZIP folder per main figure, with numerical and image data separated by panel.

- In addition, I would need from you uploaded separately: - a short, two-sentence summary of the manuscript (not more than 35 words).- two to four short (!) bullet points highlighting the key findings of your study (two lines each). - a schematic summary figure as separate file that provides a sketch of the major findings (not a data image) in jpeg or tiff format (with the exact width of 550 pixels and a height of not more than 400 pixels) that can be used as a visual synopsis on our website.

We have uploaded:

- a two-sentence summary,
- bullet points highlighting the key findings,
- and a schematic summary figure in the requested format.

Point-by-point responses for referees:

We express our gratitude to the referees for their helpful comments. We have addressed each of the referees' comments individually below and have revised the manuscript accordingly. In addition, the revised manuscript was proofread by a native English speaker to meet the editorial and referees' requests.

Referee #1:

The authors added substantial new experimental data, including lineage-tracing controls, metabolic and dietary data, functional validation of Sat1/2, expanded tumor phenotyping, and human cancer relevance. These additions transform the manuscript from a strong genetic observation into a mechanistically and conceptually mature study, with clear implications for how Hippo-activated cells can function as metabolic and signaling hubs that drive non-cell-autonomous tumorigenesis.

We thank Referee #1 for the positive evaluation regarding our revised manuscript.

The only question I still have is regarding the summary model in Figure 9. How are the amino acids transported from hippo-activated cells into the mTOR tumors? Does that also involve the Sat1/2 transporters? Or is this a different mechanism.

We thank the Referee #1 for raising this important point regarding the summary model in Figure 9. At present, the precise mechanism by which amino acids derived from Hippo-activated cells reach the mTOR-activated tumor cells remains unresolved. Our current data demonstrate that Sat1/2 are required in Hippo-activated cells for non-cell-autonomous mTOR activation; however, we did not directly determine whether Sat1/2 mediate amino acid export or whether additional transport mechanisms operate in Hippo-activated cells and the recipient cells.

To clarify this point, we have revised the Discussion (lines 371–385) to explicitly describe the possible scenarios. We now discuss two non-exclusive models: (i) Sat1/2-dependent amino acid release from Hippo-activated cells that promotes mTOR activation in neighboring cells, and (ii) indirect regulation of growth-factor production or microenvironmental metabolism that secondarily activates mTOR signaling. We agree that defining the directionality and transport route of amino acids represents an important future objective, and we have highlighted this in the revised manuscript.

Other than this question I have no further suggestions for improvement.

Referee #2:

The authors have invested admirable effort into revising their manuscript, substantially strengthening both the experimental evidence and the clarity of presentation in the text and figures. The newly added data provide additional support for the role of Sat1 and Sat2 as amino acid transporters, and overall the revision reflects a significant amount of work.

That said, I remain concerned that an important conceptual issue raised during the first round of review by two reviewers has not been satisfactorily addressed. Given the importance of the central message that Hippo-activated cells function as a niche inducing non-autonomous dysplastic overgrowth, the experimental evidence on which this conclusion is based should be resolved more rigorously to ensure that the proposed model is well supported.

Two reviewers previously raised questions regarding the origin of the pS6-positive cells observed following activation of Hippo signaling using ptc-Gal4 and en-Gal4 drivers. These cells appear in stereotypical but distinct locations for each driver sometimes even distant from the "niche", while no comparable non-autonomous pS6-positive cells are observed when authors used nubbin-Gal4 or pnr-Gal4.

While the authors performed lineage tracing for en-Gal4, they did not do so for ptc-Gal4, arguing that en-Gal4-positive cells, unlike ptc-Gal4-positive cells, infrequently undergo dedifferentiation during wing disc development refering to Nat. Methods 6:603-605, 2009. However, lineage tracing serves not only to assess changes in cell fate but also to document the developmental activity pattern of the Gal4 driver itself. As demonstrated for several drivers, including ELAV-Gal4, which is generally considered pan-neuronal, driver activity can be considerably broader than expected due to transient or early expression (Berger et al., 2007, Dev Dyn., https://doi.org/10.1002/dvdy.21372, Winant et al., 2024. Genesis, https://doi.org/10.1002/dvg.23600)

For this reason, I believe it is essential that the authors perform lineage tracing experiments for ptc-Gal4 as well. Importantly, strong thresholding should be avoided, as driver activity and signal of tracer may be weak (ubi-promoter driving tracer eGFP upon FLPout) yet biologically relevant rather than entirely absent.

We thank Referee #2 for this valuable suggestion. As suggested, we performed G-TRACE analysis by using *ptc-Gal4*, avoiding a strong threshold.

As a previous study has shown (Nat. Methods 6: 603-605, 2009), *ptc-Gal4* was not only expressed in the A/P boundary (as indicated by RFP), but also in a broader region of the anterior compartment in earlier stages (as indicated by GFP) (Fig. A and B below). However, Hippo activation using *ptc-Gal4* did not increase the number of dedifferentiated/transformed cells (Fig. C below), consistent with the results obtained using *en-Gal4* (Figure EV1E). This indicates that Hippo activation using *ptc-Gal4* does not actively induce dedifferentiation/transformation. Furthermore, although some GFP-positive cells that previously expressed *ptc-Gal4* partially overlapped with the mTOR-activated cells, mTOR activation was significantly driven in GFP-negative cells (Fig. D–F below). This indicates that mTOR activation by Hippo-activated cells occurs non-cell-autonomously, even when using *ptc-Gal4*.

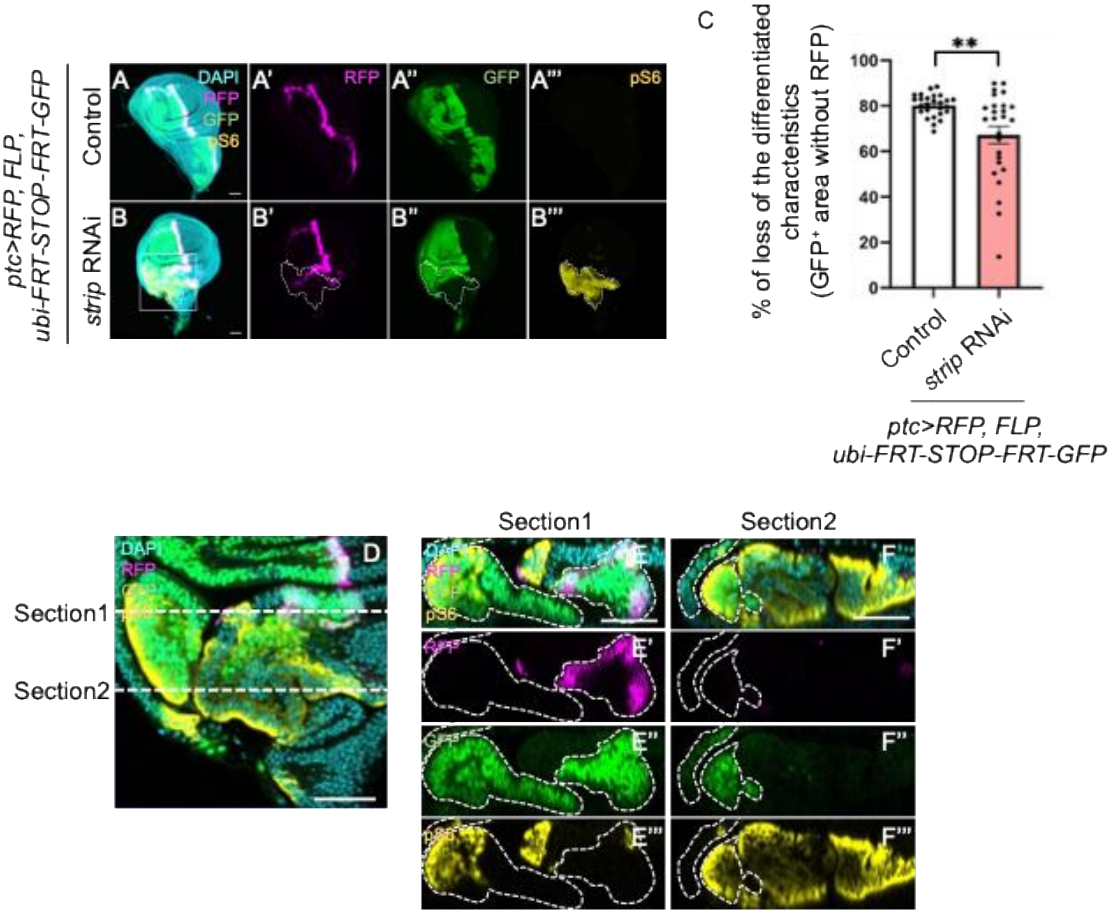

Minor points

The authors report that Sat1,Sat2 mutant wing discs do not exhibit increased apoptosis and conclude that these genes are not required for cell survival. However, such requirement may only become apparent in a competitive setting, in which Sat1,Sat2 mutant/deficient cells are surrounded by

phenotypically wild-type neighbors, as could be assessed by inducing mutant clones or RNAi-mediated deficient compartments.

We thank Referee #2 for this thoughtful comment. We agree that a requirement for Sat1/2 in cell survival could become apparent under competitive conditions, such as when mutant cells are surrounded by wild-type neighbors. In the present study, we assessed apoptosis at the tissue level and did not detect increased cell death in *Sat1/2* mutant wing discs, indicating that Sat1/2 are not generally required for basal cell survival under non-competitive conditions. However, since the potential roles of Sat1/2 in cell competition or stress-dependent survival cannot be excluded, addressing this question would require mosaic analysis in the future.

Line 126: The authors should cite the original studies that first demonstrated regulation of MMP1 by JNK, rather than only recent publication.

As Referee #2 suggested, we have added the following reference:

Uhlirova M Bohmann D (2006) JNK- and Fos-regulated Mmp1 expression cooperates with ras to induce invasive tumors in *Drosophila*. EMBO J 25:5294–304.

Figure 3A: "Cycline E" should be corrected to Cyclin E.

We have corrected this.

Referee #3:

The author of the manuscript submitted to EMBO report, untitled "The Hippo tumor suppressor pathway triggers non-cell autonomous tumorigenesis in Drosophila" significantly revised their manuscript and answered most of my queries by providing additional data.

I still have one point to highlight and suggestions regarding the text.

• The authors included new data showing that wg knockdown alone does not significantly suppress non-cell-autonomous mTOR activation (Fig. 8F). However, in Figure 4K they show that the non-cell-autonomous mTOR activation is significantly suppressed by wg knocked down. In Figure 8, en-Gal4 has been used and this driver is capable of inducing ectopic Wg (EV4). Therefore, I do not see any explanation for the discrepancy between Fig. 4K and 8F.

We thank Referee #3 for pointing out this apparent discrepancy between Fig. 4K and Fig. 8F. In these experiments, different *Gal4* drivers were used: *ptc-Gal4* in Fig. 4K and *en-Gal4* in Fig. 8F. We found that Hippo activation induces markedly different levels and spatial patterns of Wg expression depending on the driver used. Specifically, *en-Gal4*-driven Hippo activation produces broader and stronger ectopic Wg expression than *ptc-Gal4* (Right Figure). Consistent with this difference, *wg* RNAi efficiently reduces Wg in *ptc-Gal4*–expressing Hippo-activated cells, leading to a clear suppression of non-cell-autonomous mTOR activation (Fig. 4K). In contrast, under *en-Gal4* conditions, *wg* RNAi only partially reduces Wg due to the stronger and broader induction, and mTOR activation is therefore not significantly suppressed (Fig. 8F). Thus, the different outcomes reflect distinct levels of Wg depletion rather than contradictory biological effects. We have clarified this point in the Discussion (lines 360–364).

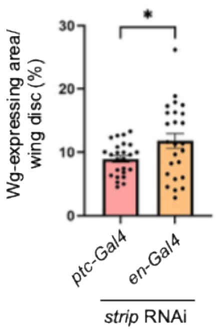

• Globally, the manuscript reading is a bit painful with very long sentences and sometime grammatical issues (Below are a few examples)-
Line 263: " To investigate the association with Sat1/2....." The association of what?
Line 329: "Given that Rho1, a regulator of the JNK pathway, was required in the Hippo-activated cells for non-cell-autonomous tumorigenesis (Fig. 4E, quantified in F), suggesting that.........."
Line 336: "However, unlike Hippo-activated cells, Src activation has shown to inhibit.....".
I would suggest the authors to revise the text.

We thank Referee #3 for these comments. This time, the entire manuscript was proofread by a native English speaker (Editage) to avoid grammatical error and enhance the readability.

• Most genetic interactions look at pS6 levels, as read out. I therefore do not think that it is correct to talk about suppression or induction of non-cell-autonomous tumorigenesis only based on this read out. Non-cell-autonomous tumorigenesis is an interpretation, but not a conclusion.

We thank the Referee #3 for this important clarification. We agree that pS6 represents a readout of mTOR activation rather than tumorigenesis per se. To ensure scientific precision, we have revised the relevant subheadings and text throughout the manuscript. Specifically, where the phenotype was assessed primarily by pS6 levels, we now refer to “non-cell-autonomous mTOR activation” instead of “non-cell-autonomous tumorigenesis”. We believe this terminology more accurately reflects the experimental readout while preserving the biological interpretation of the data.

For instance, the 3 sub-titles:

rpr, hid, grim, bantam, cyclin E, and atg8a are involved in non-cell-autonomous tumorigenesis.

Hippo activation induces non-cell-autonomous tumorigenesis through Dronc-Wg/Spitz signaling.

The activation of Dronc-Wg/Spitz signaling alone is insufficient to induce non-cell-autonomous Tumorigenesis.

Should be something like:

rpr, hid, grim, bantam, cyclin E, and atg8a are involved in non-cell-autonomous pS6 induction.

Hippo activation induces non-cell-autonomous pS6 induction through Dronc-Wg/Spitz signaling.

The activation of Dronc-Wg/Spitz signaling alone is insufficient to induce non-cell-autonomous pS6 induction.

As we described above, the sub-titles with “non-cell-autonomous tumorigenesis” were changed to non-cell-autonomous mTOR activation”.

Also, in the rest of the text, non-cell-autonomous tumorigenesis when refering to pS6 only, should be changed. The data would remain as interesting while making these changes. It is a matter of writing a scientifically correct conclusion.

As we described above, “non-cell-autonomous tumorigenesis” were changed to “non-cell-autonomous mTOR activation” in the entire manuscript.

Prof. Takahiro Chihara
Hiroshima University
Graduate School of Integrated Sciences for Life
Japan

Dear Prof. Chihara,

Thank you for the submission of your final revised manuscript to our editorial offices. I have now received the report from the referee that I asked to re-evaluate the study, you will find below. As you will see, the referees now fully supports the publication of your study in EMBO reports. Moreover, going through the manuscript, I consider the remaining editorial requests as adequately addressed.

I am thus very pleased to accept your manuscript for publication in the next available issue of EMBO reports. Thank you for your contribution to our journal.

Your manuscript will be processed for publication by EMBO Press. It will be copy edited and you will receive page proofs prior to publication. Please note that you will be contacted by Springer Nature Author Services to complete licensing and payment information.

You may qualify for financial assistance for your publication charges - either via a Springer Nature fully open access agreement or an EMBO initiative. Check your eligibility: https://link.springer.com/journal/44319/how-to-publish-with-us

Should you be planning a Press Release on your article, please get in contact with embo_production@springernature.com as early as possible in order to coordinate publication and release dates.

If you have any questions, please do not hesitate to contact the Editorial Office. Thank you for your contribution to EMBO Reports.

Yours sincerely,

Achim Breiling
Senior Editor
EMBO Reports

Referee #2:

The authors have adequately addressed the raised concerns, either by performing the necessary additional experiments or by providing reasonably satisfactory explanations. I further commend the authors for moderating their statements regarding non-cell-autonomous tumorigenesis, resulting in an a more accurate representation of their interesting findings.

>>> Please note that it is EMBO Reports policy for the transcript of the editorial process (containing referee reports and your response letter) to be published as an online supplement to each paper. If you do NOT want this, you will need to inform the Editorial Office via email immediately. More information is available here: https://link.springer.com/partners/embo-press/editorial-policies#Peer%20review

EMBO Press Author Checklist

Corresponding Author Name: Takahiro Chihara	
Journal Submitted to: EMBO Reports	
Manuscript Number: EMBOR-2025-62259V3	

USEFUL LINKS FOR COMPLETING THIS FORM
The EMBO Journal - Author Guidelines
EMBO Reports - Author Guidelines
Molecular Systems Biology - Author Guidelines
EMBO Molecular Medicine - Author Guidelines

Reporting Checklist for Life Science Articles (updated January
This checklist is adapted from Materials Design Analysis Reporting (MDAR) Checklist for Authors. MDAR establishes a minimum set of requirements in transparent reporting in the life sciences (see Statement of Task: 10.31222/osf.io/9sm4x). Please follow the journal's guidelines in preparing your
Please note that a copy of this checklist will be published alongside your article.

Abridged guidelines for figures
1. Data
The data shown in figures should satisfy the following conditions:
- the data were obtained and processed according to the field's best practice and are presented to reflect the results of the experiments in an accurate and unbiased manner.
- ideally, figure panels should include only measurements that are directly comparable to each other and obtained with the same assay.
- plots include clearly labeled error bars for independent experiments and sample sizes. Unless justified, error bars should not be shown for technical
- if n<5, the individual data points from each experiment should be plotted. Any statistical test employed should be justified.
- Source Data should be included to report the data underlying figures according to the guidelines set out in the authorship guidelines on Data

2. Captions
Each figure caption should contain the following information, for each panel where they are relevant:
- a specification of the experimental system investigated (eg cell line, species name).
- the assay(s) and method(s) used to carry out the reported observations and measurements.
- an explicit mention of the biological and chemical entity(ies) that are being measured.
- an explicit mention of the biological and chemical entity(ies) that are altered/varied/perturbed in a controlled manner.
- the exact sample size (n) for each experimental group/condition, given as a number, not a range;
- a description of the sample collection allowing the reader to understand whether the samples represent technical or biological replicates (including how many animals, litters, cultures, etc.).
- a statement of how many times the experiment shown was independently replicated in the laboratory.
- definitions of statistical methods and measures:
  - common tests, such as t-test (please specify whether paired vs. unpaired), simple χ2 tests, Wilcoxon and Mann-Whitney tests, can be unambiguously identified by name only, but more complex techniques should be described in the methods section;
  - are tests one-sided or two-sided?
  - are there adjustments for multiple comparisons?
  - exact statistical test results, e.g., P values = x but not P values < x;
  - definition of 'center values' as median or average;
  - definition of error bars as s.d. or s.e.m.

Please complete ALL of the questions below.
Select "Not Applicable" only when the requested information is not relevant for your study.

Materials

Newly Created Materials	Information included in the manuscript?	In which section is the information available? (Reagents and Tools Table, Materials and Methods, Figures, Data Availability Section)
New materials and reagents need to be available; do any restrictions apply?	Yes	Reagents and Tools Table, Methods

Antibodies	Information included in the manuscript?	In which section is the information available? (Reagents and Tools Table, Materials and Methods, Figures, Data Availability Section)
For **antibodies** provide the following information:<br>- Commercial antibodies: RRID (if possible) or supplier name, catalogue number and or/clone number<br>- Non-commercial: RRID or citation	Yes	Reagents and Tools Table, Methods

DNA and RNA sequences	Information included in the manuscript?	In which section is the information available? (Reagents and Tools Table, Materials and Methods, Figures, Data Availability Section)
**Short novel DNA or RNA including primers, probes**: provide the sequences.	Yes	Reagents and Tools Table, Methods

Cell materials	Information included in the manuscript?	In which section is the information available? (Reagents and Tools Table, Materials and Methods, Figures, Data Availability Section)
**Cell lines:** Provide species information, strain. Provide accession number in repository **OR** supplier name, catalog number, clone number, and/**OR** RRID.	Yes	Reagents and Tools Table
**Primary cultures:** Provide species, strain, sex of origin, genetic modification status.	Not Applicable	
Report if the cell lines were recently **authenticated** (e.g., by STR profiling) and tested for mycoplasma contamination.	Not Applicable	

Experimental animals	Information included in the manuscript?	In which section is the information available? (Reagents and Tools Table, Materials and Methods, Figures, Data Availability Section)
**Laboratory animals or Model organisms:** Provide species, strain, sex, age, genetic modification status. Provide accession number in repository **OR** supplier name, catalog number, clone number, **OR** RRID.	Yes	Reagents and Tools Table, Methods
**Animal observed in or captured from the field:** Provide species, sex, and age where possible.	Not Applicable	
Please detail **housing and husbandry conditions**.	Yes	Methods

Plants and microbes	Information included in the manuscript?	In which section is the information available? (Reagents and Tools Table, Materials and Methods, Figures, Data Availability Section)
**Plants:** provide species and strain, ecotype and cultivar where relevant, unique accession number if available, and source (including location for collected wild specimens).	Not Applicable	
**Microbes:** provide species and strain, unique accession number if available, and source.	Not Applicable	

Human research participants	Information included in the manuscript?	In which section is the information available? (Reagents and Tools Table, Materials and Methods, Figures, Data Availability Section)
If collected and within the bounds of privacy constraints report on age, sex and gender or ethnicity for all study participants.	Not Applicable	

Core facilities	Information included in the manuscript?	In which section is the information available? (Reagents and Tools Table, Materials and Methods, Figures, Data Availability Section)
If your work benefited from core facilities, was their service mentioned in the acknowledgments section?	Not Applicable	

Design

Study protocol	Information included in the manuscript?	In which section is the information available? (Reagents and Tools Table, Materials and Methods, Figures, Data Availability Section)
If study protocol has been **pre-registered, provide DOI in the manuscript**. For clinical trials, provide the trial registration number **OR** cite DOI.	Not Applicable	
Report the **clinical trial registration number** (at ClinicalTrials.gov or equivalent), where applicable.	Not Applicable	

Laboratory protocol	Information included in the manuscript?	In which section is the information available? (Reagents and Tools Table, Materials and Methods, Figures, Data Availability Section)
Provide DOI OR other citation details if **external detailed step-by-step protocols** are available.	Not Applicable	

Experimental study design and statistics	Information included in the manuscript?	In which section is the information available? (Reagents and Tools Table, Materials and Methods, Figures, Data Availability Section)
Include a statement about **sample size** estimate even if no statistical methods were used.	Yes	Figure lagends
Were any steps taken to minimize the effects of subjective bias when allocating animals/samples to treatment (e.g. **randomization procedure**)? If yes, have they been described?	Not Applicable	
Include a statement about **blinding** even if no blinding was done.	Not Applicable	
Describe **inclusion/exclusion criteria** if samples or animals were excluded from the analysis. Were the criteria pre-established?<br><br>If sample or data points were omitted from analysis, report if this was due to attrition or intentional exclusion and provide justification.	Not Applicable	
For every figure, are **statistical tests** justified as appropriate? Do the data meet the assumptions of the tests (e.g., normal distribution)? Describe any methods used to assess it. Is there an estimate of variation within each group of data? Is the variance similar between the groups that are being statistically compared?	Yes	Methods

Sample definition and in-laboratory replication	Information included in the manuscript?	In which section is the information available? (Reagents and Tools Table, Materials and Methods, Figures, Data Availability Section)
In the figure legends: state number of times the experiment was **replicated** in laboratory.	Yes	Figure lagends
In the figure legends: define whether data describe **technical or biological replicates**.	Yes	Figure lagends

Ethics

Ethics	Information included in the manuscript?	In which section is the information available? (Reagents and Tools Table, Materials and Methods, Figures, Data Availability Section)
Studies involving **human participants**: State details of **authority granting ethics approval** (IRB or equivalent committee(s), provide reference number for approval.	Not Applicable	
Studies involving **human participants**: Include a statement confirming that **informed consent** was obtained from all subjects and that the experiments conformed to the principles set out in the WMA Declaration of Helsinki and the Department of Health and Human Services Belmont Report.	Not Applicable	
Studies involving **human participants:** For publication of **patient photos**, include a statement confirming that consent to publish was obtained.	Not Applicable	
Studies involving experimental **animals**: State details of **authority granting ethics approval** (IRB or equivalent committee(s), provide reference number for approval. Include a statement of compliance with ethical regulations.	Not Applicable	
Studies involving **specimen and field samples:** State if relevant **permits** obtained, provide details of authority approving study; if none were required, explain why.	Not Applicable	

Dual Use Research of Concern (DURC)	Information included in the manuscript?	In which section is the information available? (Reagents and Tools Table, Materials and Methods, Figures, Data Availability Section)
Could your study fall under dual use research restrictions? Please check biosecurity documents and list of **select agents and toxins** (CDC): https://www.selectagents.gov/sat/list.htm	Not Applicable	
If you used a select agent, is the security level of the lab appropriate and reported in the manuscript?	Not Applicable	
If a study is subject to dual use research of concern regulations, is the name of the **authority granting approval and reference number** for the regulatory approval provided in the manuscript?	Not Applicable	

Reporting

The MDAR framework recommends adoption of discipline-specific guidelines, established and endorsed through community initiatives. Journals have their own policy about requiring specific guidelines and recommendations to complement MDAR.

Adherence to community standards	Information included in the manuscript?	In which section is the information available? (Reagents and Tools Table, Materials and Methods, Figures, Data Availability Section)
State if relevant guidelines or checklists (e.g., **ICMJE, MIBBI, ARRIVE, PRISMA**) have been followed or provided.	Not Applicable	
For **tumor marker prognostic studies,** we recommend that you follow the **REMARK** reporting guidelines (see link list at top right). See author guidelines, under 'Reporting Guidelines'. Please confirm you have followed these guidelines.	Not Applicable	
For **phase II and III randomized controlled trials**, please refer to the **CONSORT** flow diagram (see link list at top right) and submit the CONSORT checklist (see link list at top right) with your submission. See author guidelines, under 'Reporting Guidelines'. Please confirm you have submitted this list.	Not Applicable	

Data Availability

Data availability	Information included in the manuscript?	In which section is the information available? (Reagents and Tools Table, Materials and Methods, Figures, Data Availability Section)
Have **primary datasets** been deposited according to the journal's guidelines (see 'Data Deposition' section) and the respective accession numbers provided in the Data Availability Section?	Yes	Data Availability
Were **human clinical and genomic datasets** deposited in a public access-controlled repository in accordance to ethical obligations to the patients and to the applicable consent agreement?	Not Applicable	
Are **computational models** that are central and integral to a study available without restrictions in a machine-readable form? Were the relevant accession numbers or links provided?	Not Applicable	
If publicly available data were reused, provide the respective **data citations in the reference list**.	Not Applicable	
